# Comprehensive Analysis of Hub Genes Associated With Competing Endogenous RNA Networks in Stroke Using Bioinformatics Analysis

**DOI:** 10.3389/fgene.2021.779923

**Published:** 2022-01-12

**Authors:** Xiuqi Chen, Danhong Wu

**Affiliations:** Department of Neurology, Shanghai Fifth People’s Hospital, Fudan University, Shanghai, China

**Keywords:** ischemic stroke, lncRNA, bioinformatics analysis, ceRNA, biomarkers

## Abstract

**Background:** Acute ischemic stroke (AIS) is the second leading cause of death and the third leading cause of disability worldwide. Long noncoding RNAs (lncRNAs) are promising biomarkers for the early diagnosis of AIS and closely participate in the mechanism of stroke onset. However, studies focusing on lncRNAs functioning as microRNA (miRNA) sponges to regulate the mRNA expression are rare and superficial.

**Methods:** In this study, we systematically analyzed the expression profiles of lncRNA, mRNA (GSE58294), and miRNA (GSE110993) from the GEO database. Gene ontology (GO) analysis was performed to reveal the functions of differentially expressed genes (DEGs), and we used weighted gene co-expression network analysis (WGCNA) to investigate the relationships between clinical features and expression profiles and the co-expression of miRNA and lncRNA. Finally, we constructed a lncRNA–miRNA–mRNA competing endogenous RNA (ceRNA) network with selected DEGs using bioinformatics methods and obtained ROC curves to assess the diagnostic efficacy of differentially expressed lncRNAs (DElncRNAs) and differentially expressed mRNAs (DEmRNAs) in our network. The GSE22255 dataset was used to confirm the diagnostic value of candidate genes.

**Results:** In total, 199 DElncRNAs, 2068 DEmRNAs, and 96 differentially expressed miRNAs were detected. The GO analysis revealed that DEmRNAs primarily participate in neutrophil activation, neutrophil degranulation, vacuolar transport, and lysosomal transport. WGCNA screened out 16 lncRNAs and 195 mRNAs from DEGs, and only eight DElncRNAs maintained an area under the curve higher than 0.9. By investigating the relationships between lncRNAs and mRNAs, a ceRNA network containing three lncRNAs, three miRNAs, and seven mRNAs was constructed. GSE22255 confirmed that RP1-193H18.2 is more advantageous for diagnosing stroke, whereas no mRNA showed realistic diagnostic efficacy.

**Conclusion:** The ceRNA network may broaden our understanding of AIS pathology, and the candidate lncRNA from the ceRNA network is assumed to be a promising therapeutic target and diagnostic biomarker for AIS.

## Introduction

Stroke is the second leading cause of death and the third leading cause of disability in adults (Campbell and Khatri, 2020). Approximately 80% of all strokes are ischemic (Donnan et al., 2008); thus, rehabilitation after ischemic stroke garners much attention in stroke research. However, safe and successful rehabilitation for stroke is strictly limited by the therapeutic time window (Emberson et al., 2014), which renders early diagnosis extremely critical. At present, the diagnosis of ischemic stroke primarily depends on typical clinical symptoms and auxiliary brain imaging examinations (Campbell and Khatri, 2020). However, clinical manifestations of stroke are often confusing, and brain imaging examinations (such as CT and MRI) are time-consuming and relatively expensive, and their use may be restricted by the condition of the patient. In addition, the mechanisms underlying stroke have not been thoroughly clarified (Campbell and Khatri, 2020). Thus, it is necessary to identify novel potential biomarkers and mechanisms for the early detection of stroke onset.

Long noncoding RNAs (lncRNAs) are defined as transcripts that are longer than 200 nucleotides and are unable to encode proteins (Kopp and Mendell, 2018). They were previously considered as “junk” in the mammal genome but now are found to function as a transcriptional regulator or post-transcriptional regulator: in the former, lncRNA combines with chromosomes and modifies the gene expression (Ulitsky and Bartel, 2013), whereas in the latter, lncRNA can either directly impact the gene expression by regulating the degradation of genes or works as competing endogenous RNA (ceRNA) and sponge-specific miRNAs, thus indirectly regulating the gene expression (Bao et al., 2018; Gong et al., 2020). Recently, aberrant expression of lncRNA has been reported to correlate with the onset, progression, and prognosis of acute ischemic stroke (AIS) (Bai et al., 2014; Feng et al., 2019; Li et al., 2020a; Wang et al., 2020a; Fu et al., 2021); thus they are potential candidates for stroke diagnosis. Meanwhile, the mechanism of stroke involves a series of complicated processes, including energy depletion, ion imbalance, glutamate and free radical release, calcium channel dysfunction, inflammatory changes, and apoptosis (Dirnagl et al., 1999). lncRNAs are reported to be involved in the progression of inflammation (Feng et al., 2019; Zhang and Niu, 2020) and apoptosis (Liu et al., 2016; Chen et al., 2017; Liu et al., 2019; Zhao et al., 2019; Zhong et al., 2020) in stroke onset, indicating that lncRNAs may also be key factors in understanding the mechanism of stroke.

Generally, lncRNA research in neuroscience is still in its preliminary stages, and much has focused on lncRNAs as direct gene expression regulators; only a few have investigated the possibility that lncRNAs act as ceRNAs to indirectly regulate the gene expression, and comprehensive studies that have explored lncRNAs and possible interactive genes in stroke are also scarce. The development of microarray and high-throughput sequencing has helped in gene function interpretation (Gershon, 2002), combined with bioinformatics analysis; our knowledge in the field of lncRNA and stroke will be greatly broadened.

In this study, we constructed a lncRNA–miRNA–mRNA network by collecting differentially expressed genes (DEGs) from the GSE110993 and GSE58294 series, and then, we verified the diagnostic efficacy of this network in the GSE22255 series. We also provide a useful framework for elucidating the molecular mechanisms of ischemic stroke at the biological level.

## Materials and Methods

### Data Downloading and Data Processing

In this study, we used “stroke” and “*Homo sapiens*” as mesh terms in the GEO database. Only patients without any clinical treatment were included in this study, and the time from stroke onset to admission was less than 3 h. We found two eligible series, GSE58294 (Stamova et al., 2014) and GSE110993 (Tiedt et al., 2017). The GSE58294 series included 23 stroke patients and 23 matched healthy controls. The matching criteria involved vascular diseases related to risk factors and sex. This transcriptome series was based on the GPL570 [HG-U133_Plus_2] Affymetrix Human Genome U133 Plus 2.0 Array. The GSE110993 series included 29 stroke patients and 20 matched healthy controls, and the matching criteria involved vascular diseases related to risk factors, sex, age, and past medical history. This miRNA high-throughput sequencing series was based on GPL15456 (Illumina HiScanSQ), and all patients were included in this study. In addition, the GSE22255 series was used as a verification series, including 20 stroke patients and 20 healthy controls matched for age and sex. This transcriptome series was based on the GPL570 Affymetrix Human Genome U133 Plus 2.0 Array.

The raw data from GSE58294 were first read and normalized by affy (Gautier et al., 2004) and gcRMA R packages and then visualized by the boxplot and principal component analysis plot. Combat function in the sva R package (Leek et al., 2012) was used to adjust the batch effect in this series. The probe annotation of this series was based on Entrez Gene ID. The lncRNA and mRNA annotation files of GRCh38 (Schneider et al., 2017) were downloaded from Affymetrix using the Rsubread package. In the comment process, only the matched probe remained in the expression profile. If one Entrez Gene ID matched many probes, the average of these values was used. Differential expression analysis of GSE110993 was provided in the GEO datasets.

### Differential Expression Analysis

Differentially expressed mRNAs (DEmRNAs) and differentially expressed lncRNAs (DElncRNAs) from GSE58294 were filtered using the limma package (Ritchie et al., 2015). Differentially expressed miRNAs (DEmiRNAs) from GSE110993 were selected using the edgeR (Robinson et al., 2010) package. We defined the predictive threshold set as adj. *p* value/false discovery rate (FDR) < 0.05 and |log2 (foldchange)|>0.5 (Dalman et al., 2012; Zhang et al., 2016). ggplot2 and pheatmap packages were used to draw the volcanic maps and heat maps, respectively. In addition, the distribution of DEmRNAs and DElncRNAs on the chromosome was visualized using the OmicCircos package in this study.

### Construction of a Protein–Protein Interaction (PPI) Network

To further investigate the PPIs, we used the online Search Tool for the Retrieval of Interacting Genes (STRING, version 11.0; https://string-db.org/) database (Szklarczyk et al., 2018) to estimate the interaction between DEmRNAs. Only datasets with a combined score higher than 0.9 were involved in the PPI network, and Cytoscape was used to construct and visualize this PPI network (Shannon et al., 2003). We used cytoHubba (Chin et al., 2014) to identify 10 key genes from the PPI network using the topology MCC approach.

### Weighted Gene Co-expression Network Analysis (WGCNA)

Sample clustering was performed to verify the correlation between the expression profiles and clinical features. Vital gene modules were distinguished using the WGCNA R package (Langfelder and Horvath, 2008) after processing the raw data. First, Pearson’s correlation coefficients were computed for filtered genes in a pair-wise approach producing a gene similarity matrix. Thereafter, closely correlated gene modules were recognized by average linkage hierarchical clustering. Network internal connectivity was gauged by calculating the topological overlap, and the latter was realized using the TOM dist function with a signed TOM-Type. Average hierarchical clustering was used to classify the genes based on the topological overlap dissimilarity measure (1-TOM) of their connection strengths. The author used a dynamic tree cut algorithm with a minimum cluster size of 30 and a merging threshold of 0.05 to identify network modules. Genes that were not grouped into specific modules were classified as “colored gray”.

### Construction of the ceRNA Network

According to the admitted hypothesis, lncRNA indirectly regulates the mRNA expression by competitively interacting with miRNA (Bao et al., 2018; Gong et al., 2020). In this article, we constructed our ceRNA network as follows (Campbell and Khatri, 2020): predicting possible lncRNAs that interact with miRNA by using prediction modules of DIANA-LncBase Predicted V.2 database (Paraskevopoulou et al., 2016) and forming pairwise relations between lncRNA and miRNA; the interacting score of prediction modules higher than 0.7 considered as eligible (Donnan et al., 2008); miRWalk (Dweep et al., 2011) 3.0, miRDB (Chen and Wang, 2019), and TargetScan (Agarwal et al., 2015) (Version 7.2), were used to predict the interaction between miRNAs and mRNAs, and the interacting score higher than 0.95 was regarded as eligible (Emberson et al., 2014); lncRNAs and mRNAs predicted by previous two steps were matched with DElncRNAs and DEmRNAs, and only overlapped lncRNA–miRNA and mRNA–miRNA relationships were selected (Kopp and Mendell, 2018); arranging these paired matched relationships, the ultimate lncRNA–miRNA–mRNA network was formed and visualized through Cytoscape (Version 3.8.0).

### Functional Enrichment Analysis

Gene ontology (GO) functional enrichment analysis was performed by the clusterProfiler R package (Yu et al., 2012) and visualized using the ggplot2 R package to articulate ceRNA network-associated biological processes. The criterion was set as the adj. *p* value < 0.05.

### Diagnostic ROC Curve

To assess the diagnostic efficacy of selected DElncRNAs and DEmRNAs, we used the Proc R package to generate the ROC curve. Next, the area under the curve (Stamova et al., 2014) and 95% confidence interval were calculated to verify the reliability of the diagnostic curve. GSE22255 was used as a validation series in this study.

## Results

### Differentially Expressed lncRNA, miRNA, and mRNA

**FIGURE 1:**
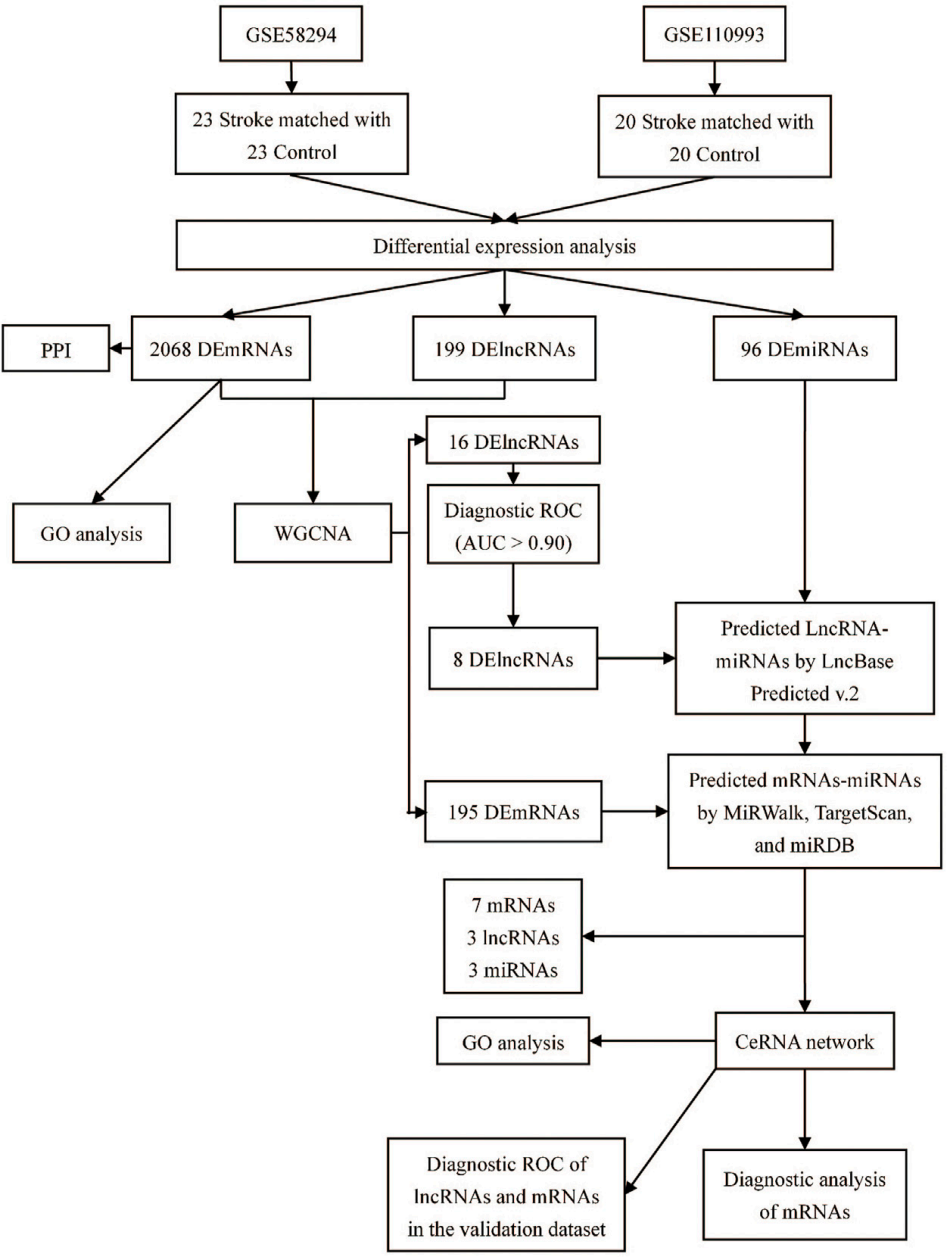
Study flow chart.

**FIGURE 2:**
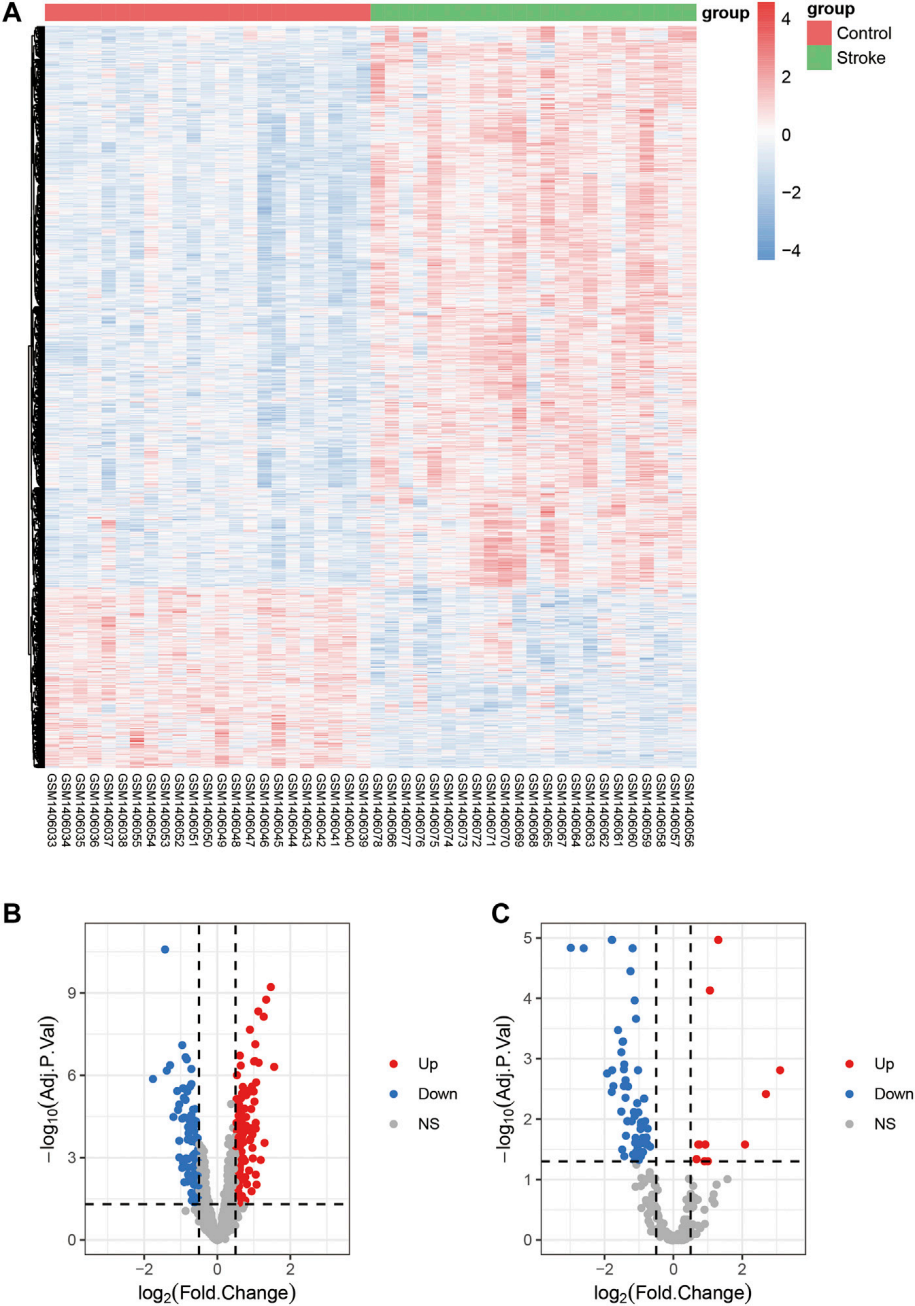
Differentially expressed mRNAs, miRNAs, and lncRNAs in stroke. **(A)**: Heatmap shows DEmRNAs from GSE58294. **(B)**: Volcanic map displaying DElncRNAs from GSE58294. **(C)**: Volcanic map exhibiting DEmiRNAs from GSE110993; *stroke* denotes the group of stroke patients, and control denotes the group with healthy controls; *Up;* upregulation, *down;* downregulation, *NS;* not significant.

**FIGURE 3:**
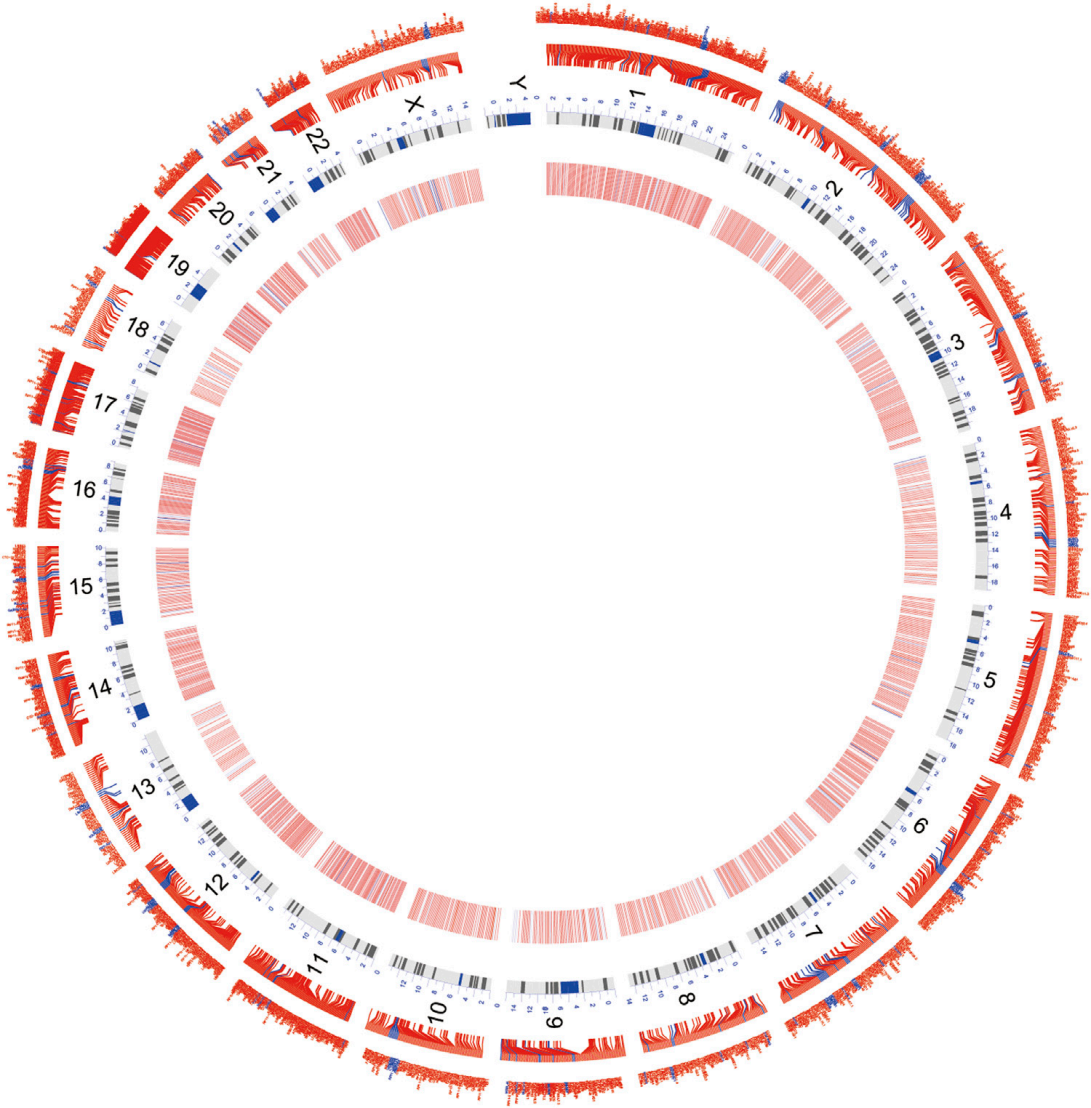
Circos map demonstrates the distribution of DEmRNAs and DElncRNAs on the chromosome. Red characters and the transverse line in the outer layer denote DEmRNAs; blue characters and the transverse line in the inner layer denote DElncRNAs; the inner circle comprising red, white, and blue stripes is the heatmap for the differential expression. Red denotes the high expression, and blue denotes the low expression.

A flow chart of this study is shown in Figure 1. In our study, 2068 DEmRNAs and 199 DElncRNAs were identified between stroke patients and healthy controls from GSE58294, including 1564 upregulated and 504 downregulated mRNAs, as well as 104 upregulated and 95 downregulated lncRNAs. We displayed DEmRNAs in the manner of the heatmap in Figure 2A and DElncRNAs in the manner of the volcanic map in Figure 2B. Red denotes that this gene is upregulated in the peripheral blood of stroke patients, whereas blue denotes that this gene is downregulated in the peripheral blood of stroke patients. According to the differentially expressed analysis of GSE110993, 96 DEmiRNAs were detected, and 16 upregulated miRNAs and 80 downregulated miRNAs were included. We exhibited the outcomes in the volcanic map, as shown in Figure 2C. The top 50 most significant DEmRNAs and DElncRNAs are listed in Tables 1, 2, respectively; the top 32 most significant DEmiRNAs are listed in Table 3. We also generated Figure 3 to display the distribution of DEmRNAs and DElncRNAs on the chromosome. It showed that DEmRNAs and DElncRNAs spread to all autosomes and the X chromosome, and none of them were found in the Y chromosome.

**TABLE 1:** Top 50 differentially expressed mRNAs in stroke samples: half upregulated and half downregulated.

Name	LogFC	Adj.*p*.Val	Name	LogFC	Adj.*p*.Val
Top 25 upregulated mRNAs			Top 25 downregulated mRNAs		
ARG1	2.0780	6.78E-07	ACSM2A	−3.2499	1.08E-16
OLAH	1.8088	1.38E-06	HLA-DQA1	−2.8088	3.96E-02
ANKRD22	1.7978	1.02E-07	TIMM8A	−2.7536	1.27E-15
CACNA1E	1.7615	2.79E-07	SH3GL3	−2.5319	1.34E-15
MAOA	1.7313	1.49E-03	SRCIN1	−2.4742	8.16E-13
MCEMP1	1.6682	1.31E-08	FAT3	−2.0960	2.32E-09
LILRA5	1.6201	1.67E-08	OVOL2	−2.0655	2.59E-10
VSIG4	1.6178	1.58E-07	BTNL3	−1.9992	5.13E-03
INSC	1.5845	2.86E-06	LPAR4	−1.9849	1.67E-07
FCAR	1.4816	4.27E-05	FAM133A	−1.9488	7.00E-11
BTNL8	1.4811	2.67E-02	PRTG	−1.9286	1.89E-11
SLC26A8	1.4386	3.55E-06	LECT2	−1.8916	5.31E-09
FGF13	1.4182	2.42E-02	THSD4	−1.8860	5.27E-10
SAP30	1.4167	8.70E-09	GAGE1	−1.8279	1.55E-08
BMX	1.4047	4.57E-06	GABRB2	−1.8149	1.39E-07
HNRNPL	1.3761	5.27E-10	ZNF536	−1.8108	7.94E-10
HTRA1	1.3550	1.26E-04	SHOX	−1.7767	2.29E-11
CLEC5A	1.3495	2.98E-07	ZNF595	−1.6665	3.48E-04
SEMG1	1.3432	2.08E-03	RNF165	−1.5800	3.62E-06
MMP9	1.3429	1.81E-05	GLYATL1	−1.5635	7.55E-10
ECHDC3	1.3402	3.53E-05	SPTLC3	−1.5420	3.34E-11
PRRG4	1.3348	4.56E-09	RBMS3	−1.5060	4.05E-08
CFD	1.3278	1.90E-05	USP43	−1.5000	1.87E-04
COX7B	1.3162	4.45E-06	PPP5D1	−1.4930	1.63E-08
CLEC4D	1.3098	6.77E-07	CXADR	−1.4875	1.08E-10

**TABLE 2:** Top 50 differentially expressed lncRNAs in stroke samples: half upregulated and half downregulated.

Name	LogFC	Adj.*p*.Val	Name	LogFC	Adj.*p*.Val
Top 25 upregulated lncRNAs			Top 25 downregulated lncRNAs		
RP11-111K18.2	1.5596	5.00E-07	LINC00883	−1.7621	1.38E-06
TOPORS-AS1	1.4688	6.02E-10	RP11-69I8.2	−1.4317	2.62E-11
DLGAP1-AS2	1.3390	1.78E-09	RP11-744D14.2	−1.3812	6.77E-07
RP11-476D10.1	1.2949	2.93E-04	DKFZP434L187	−1.2934	4.24E-07
RP11-6I2.3	1.2719	7.30E-09	LINC00540	−1.2037	3.32E-05
LINC01270	1.1892	1.22E-03	RP1-142L7.8	−1.1003	3.71E-06
LINC00282	1.1333	3.43E-07	LINC00566	−1.0718	1.80E-05
RP1-193H18.2	1.1203	4.60E-09	RP11-319G9.3	−1.0436	2.40E-04
RP3-525N10.2	1.0751	9.63E-03	LINC00624	−1.0422	1.15E-05
RP11-443B7.1	1.0611	1.80E-06	LINC01146	−1.0337	9.88E-04
CYP1B1-AS1	1.0589	4.15E-03	GAS6-AS1	−0.9631	2.37E-03
FRY-AS1	1.0538	5.39E-05	LINC00550	−0.9560	7.94E-08
RP11-44F14.8	1.0507	8.81E-05	LINC00592	−0.9546	3.74E-05
FAM13A-AS1	1.0384	7.51E-08	LINC00323	−0.9330	1.48E-03
RP11-330O11.3	1.0306	2.97E-07	LA16c-83F12.6	−0.9318	2.94E-06
RP1-228H13.5	1.0149	8.92E-06	C1QTNF1-AS1	−0.9165	6.65E-06
RP3-368A4.6	1.0042	3.09E-07	DCTN1-AS1	−0.9055	1.31E-03
RP11-305L7.3	0.9972	3.85E-06	RP4-680D5.8	−0.9005	3.38E-05
BFSP2-AS1	0.9744	1.07E-03	LINC01013	−0.8965	7.82E-03
LINC01410	0.9636	4.37E-04	RP11-749H17.2	−0.8728	7.85E-06
RP11-66N11.8	0.9576	1.42E-04	SEPSECS-AS1	−0.8684	2.18E-07
CTD-2033C11.1	0.9550	2.58E-06	RP11-138I18.2	−0.8651	1.18E-03
LINC01094	0.9405	1.18E-04	CTBP1-AS	−0.8514	1.07E-03
RP11-421F16.3	0.9352	1.67E-02	PP7080	−0.8316	2.65E-07
ST3GAL4-AS1	0.9311	1.68E-05	RP11-395I6.3	−0.8219	7.48E-03

**TABLE 3:** Top 32 differentially expressed miRNAs in stroke samples: half upregulated and half downregulated.

Name	LogFC	Adj.*p*.Val	Name	LogFC	Adj.*p*.Val
Top 16 upregulated miRNAs			Top 16 downregulated miRNAs		
hsa-miR-512-3p	3.0942	1.55E-03	hsa-mir-1-1	−2.9783	1.46E-05
hsa-miR-516b-5p	2.6903	3.79E-03	hsa-miR-1	−2.6045	1.48E-05
hsa-miR-516a-5p	2.0776	2.63E-02	hsa-mir-3158-2	−1.9255	1.75E-03
hsa-miR-125a-5p	1.3056	1.08E-05	hsa-mir-3158-1	−1.9255	1.75E-03
hsa-mir-125a	1.303	1.08E-05	hsa-miR-18a-5p	−1.7875	3.52E-03
hsa-miR-99b-5p	1.0619	7.40E-05	hsa-mir-660	−1.7857	1.08E-05
hsa-mir-99b	1.0565	7.40E-05	hsa-miR-660-5p	−1.7833	1.08E-05
hsa-mir-485	1.0066	4.94E-02	hsa-mir-193a	−1.7756	1.55E-03
hsa-miR-99a-5p	0.9271	2.63E-02	hsa-miR-193a-5p	−1.7423	2.83E-03
hsa-mir-99a	0.9271	2.63E-02	hsa-miR-532-5p	−1.6046	3.37E-04
hsa-miR-143-3p	0.9073	4.93E-02	hsa-mir-20a	−1.515	7.42E-03
hsa-mir-143	0.9071	4.93E-02	hsa-miR-20a-5p	−1.515	7.42E-03
hsa-miR-125b-5p	0.7593	2.63E-02	hsa-mir-18a	−1.5052	7.76E-04
hsa-mir-125b-2	0.7262	2.63E-02	hsa-miR-3143	−1.4845	3.16E-02
hsa-miR-10b-5p	0.6686	4.56E-02	hsa-mir-101-2	−1.4706	5.22E-04
hsa-mir-10b	0.6686	4.56E-02	hsa-miR-101-3p	−1.4615	5.12E-04

### PPI Network

**FIGURE 4:**
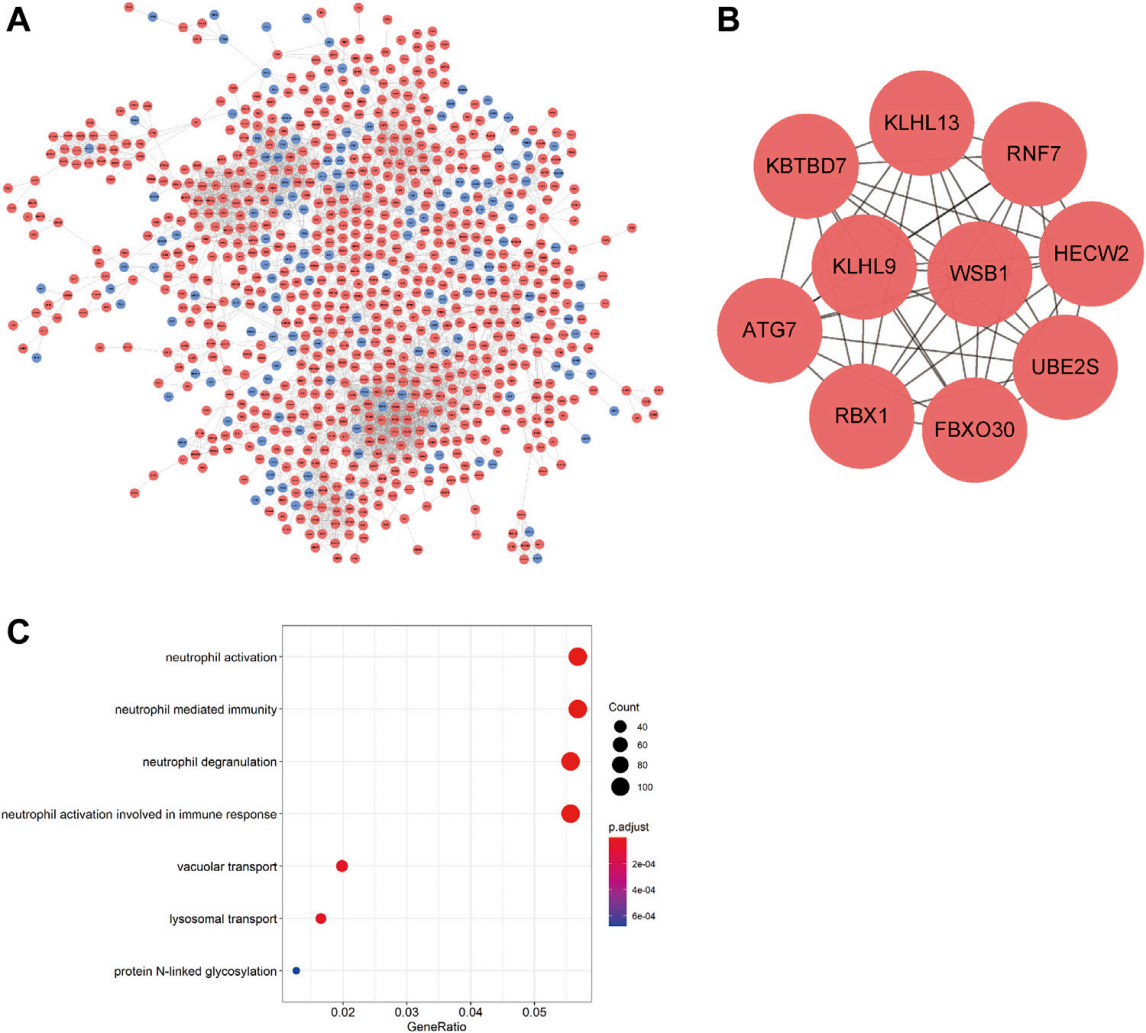
PPI network of DEmRNAs and GO analysis. **(A)** PPI network; blue denotes downregulated genes; red denotes upregulated genes. **(B)** 10 hub genes. **(C)** GO functional enrichment analysis of DEmRNAs.

The PPI network was constructed using the STRING online database and visualized by Cytoscape to detect potential interactions among DEmRNAs. This network included 853 nodes and 4243 pairs of interactive relationships (Figure 4A), and ten hub genes were selected according to the MCC function of topological analysis: *KLHL13*, *RNF7*, *KBTBD7*, *KLHL9*, *WSB1*, *HECW2*, *ATG7*, *RBX1*, *FBXO30*, and *UBE2S* (Figure 4B).

### GO Analysis

GO analysis was performed to investigate the functions of the DEmRNAs; the outcomes of GO functional enrichment analysis are displayed in Figure 4C. There were 46 terms significantly enriched in this article (Table 4). The biological process (BP) outcomes revealed that the top 10 processes that DEmRNAs were most enriched in neutrophil activation, neutrophil degranulation, neutrophil-mediated immunity, and neutrophil activation involved in immune response, lysosomal transport, vacuolar transport, protein N-linked glycosylation, response to antineoplastic agent, glycoprotein biosynthetic process, and vesicle organization, and the cellular component (Robinson et al., 2010) outcomes demonstrated that the top 10 DEmRNAs mostly related terms were specific granules, tertiary granules, specific granule lumens, secretory granule membranes, vacuolar membranes, ficolin-1-rich granules, lysosomal membranes, lytic vacuole membranes, specific granule membranes, and endosome membranes. No molecular function (MF) terms were identified.

**TABLE 4:** GO enrichment analysis of all differentially expressed mRNAs.

GO_Category	Term	Count	*P*.Adjust
BP	GO:0042119∼neutrophil activation	103	1.78E-10
BP	GO:0043312∼neutrophil degranulation	101	1.78E-10
BP	GO:0002446∼neutrophil-mediated immunity	103	1.78E-10
BP	GO:0002283∼neutrophil activation involved in immune response	101	1.97E-10
BP	GO:0007041∼lysosomal transport	30	5.48E-05
BP	GO:0007034∼vacuolar transport	36	5.48E-05
BP	GO:0006487∼protein N-linked glycosylation	23	6.78E-04
BP	GO:0097327∼response to antineoplastic agent	24	8.39E-03
BP	GO:0009101∼glycoprotein biosynthetic process	58	1.93E-02
BP	GO:0016050∼vesicle organization	55	1.93E-02
BP	GO:0007040∼lysosome organization	17	2.37E-02
BP	GO:0080171∼lytic vacuole organization	17	2.37E-02
BP	GO:0002698∼negative regulation of immune effector process	26	3.31E-02
BP	GO:0048736∼appendage development	34	4.00E-02
BP	GO:0060173∼limb development	34	4.00E-02
BP	GO:0008333∼endosome to lysosome transport	16	4.00E-02
BP	GO:0000070∼mitotic sister chromatid segregation	30	4.00E-02
CC	GO:0042581∼specific granule	46	2.47E-09
CC	GO:0070820∼tertiary granule	44	4.46E-08
CC	GO:0035580∼specific granule lumen	21	2.80E-05
CC	GO:0030667∼secretory granule membrane	57	3.51E-05
CC	GO:0005774∼vacuolar membrane	72	3.51E-05
CC	GO:0101002∼ficolin-1-rich granule	40	6.43E-05
CC	GO:0005765∼lysosomal membrane	62	1.50E-04
CC	GO:0098852∼lytic vacuole membrane	62	1.50E-04
CC	GO:0035579∼specific granule membrane	24	1.98E-04
CC	GO:0010008∼endosome membrane	77	1.98E-04
CC	GO:0008250∼oligosaccharyltransferase complex	8	3.46E-04
CC	GO:1904724∼tertiary granule lumen	17	4.57E-04
CC	GO:0005769∼early endosome	59	4.94E-04
CC	GO:0070821∼tertiary granule membrane	20	4.94E-04
CC	GO:0034774∼secretory granule lumen	54	1.10E-03
CC	GO:0005770∼late endosome	45	1.61E-03
CC	GO:0060205∼cytoplasmic vesicle lumen	55	2.00E-03
CC	GO:0005811∼lipid droplet	20	2.00E-03
CC	GO:0031983∼vesicle lumen	55	2.05E-03
CC	GO:0030496∼midbody	33	2.72E-03
CC	GO:1904813∼ficolin-1-rich granule lumen	25	7.55E-03
CC	GO:0030139∼endocytic vesicle	48	9.06E-03
CC	GO:0101003∼ficolin-1-rich granule membrane	15	1.38E-02
CC	GO:0030666∼endocytic vesicle membrane	30	1.38E-02
CC	GO:0030136∼clathrin-coated vesicle	32	2.30E-02
CC	GO:0035577∼azurophil granule membrane	14	2.30E-02
CC	GO:0045335∼phagocytic vesicle	24	3.44E-02
CC	GO:0005766∼primary lysosome	27	3.44E-02
CC	GO:0042582∼azurophil granule	27	3.44E-02

### Analysis of lncRNA and mRNA Co-expression

**FIGURE 5:**
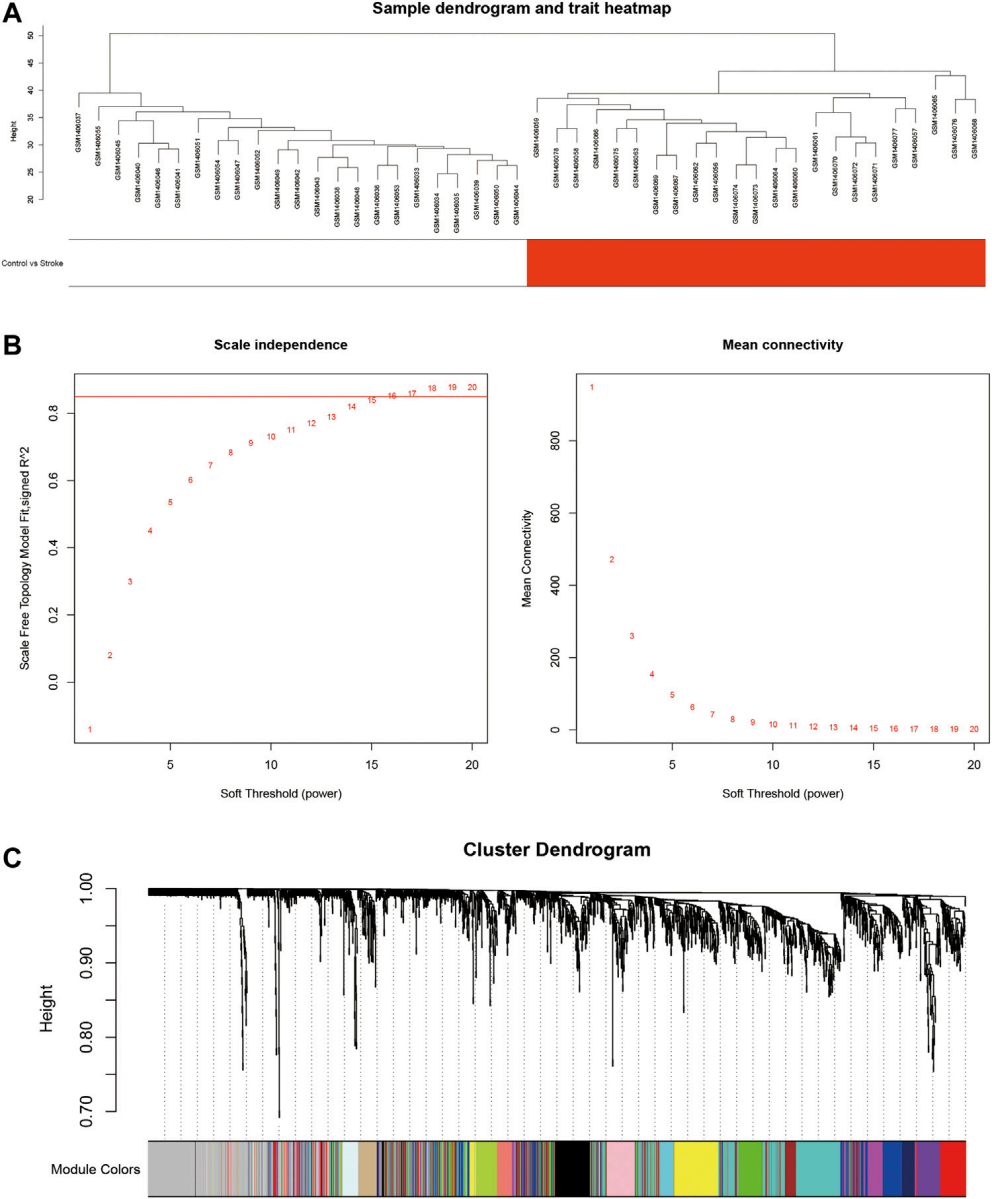
Constructing the lncRNA and mRNA co-expression module using WGCNA. **(A)**: Clustering analysis of 40 samples from GSE58794 based on the expression profiles of lncRNAs and mRNAs. White denotes the healthy control group, and red denotes the stroke group. **(B)**: Network topological analysis targeting multiple soft thresholds. The soft threshold was set at 15. The red straight line denotes the boundary of the similarity as 0.85. **(C)**: Clustering dendrogram of mRNAs and lncRNAs.

**FIGURE 6:**
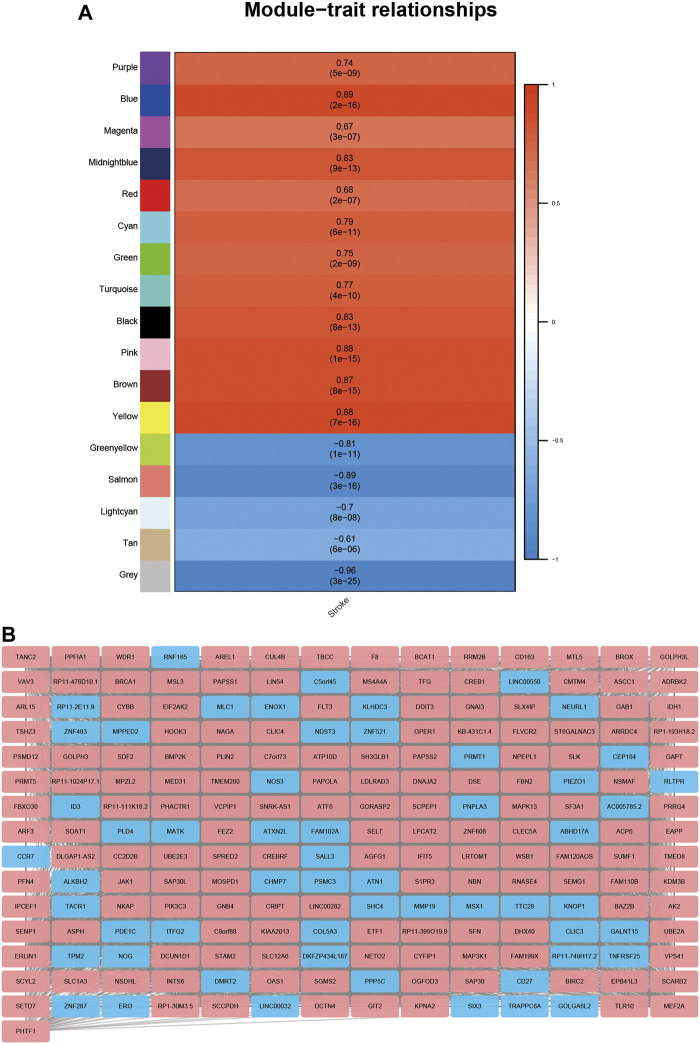
Detection of gene modules closely related to stroke using WGCNA. **(A)**: Correlation analysis on gene modules and clinical traits. Each row matches relative gene collection, and each column matched the relative clinical traits. The upper figure in the blank is the correlation coefficient, and the lower figure in the blank is the *p* value. **(B)**: Co-expression network of lncRNAs and mRNAs in blue modules; red represents the upregulation of the gene, and blue represents the downregulation of the gene.

To investigate the DElncRNAs and DEmRNAs related to biological processes, the regulation patterns of lncRNAs and mRNAs should be clearly defined. Based on the hypothesis that lncRNAs competitively combine miRNAs, a positive correlation between lncRNAs and mRNAs should be selected. We performed a co-expression analysis of lncRNA and mRNA from GSE58794 using the WGCNA R package (Figure 5). Figure 5A reveals that no outliers were detected in sample clustering; thus, there was no need to eliminate samples in subsequent WGCNA. Fifteen were chosen as the cut-off for the soft threshold. Clustering analysis on the similarity of mRNA and lncRNA expression profiles was performed, and modules were combined if the differences between them were less than 5% (Figure 5C). Eventually, we selected the blue modules that are most closely related to stroke (*R* = 0.89, *p* < 0.0001) based on the correlation coefficient between modules and clinical traits, and this is displayed in Figure 6A. After selecting the modules, we constructed a co-expression network. A weighted score higher than 0.02 was considered as the threshold, and lncRNA and mRNA should have similar expression tendencies as well. In Figure 6B, our network summarizes 7092 pairs of interactive relationships, and more detailed information is available in Supplementary Table S1. A total of 211 genes, including 16 lncRNAs and 195 mRNAs, were incorporated into this module. There were 10 upregulated and six downregulated lncRNAs, whereas among 195 mRNAs, 145 were upregulated and the rest were downregulated.

### Diagnostic ROC Curve of lncRNA

**FIGURE 7:**
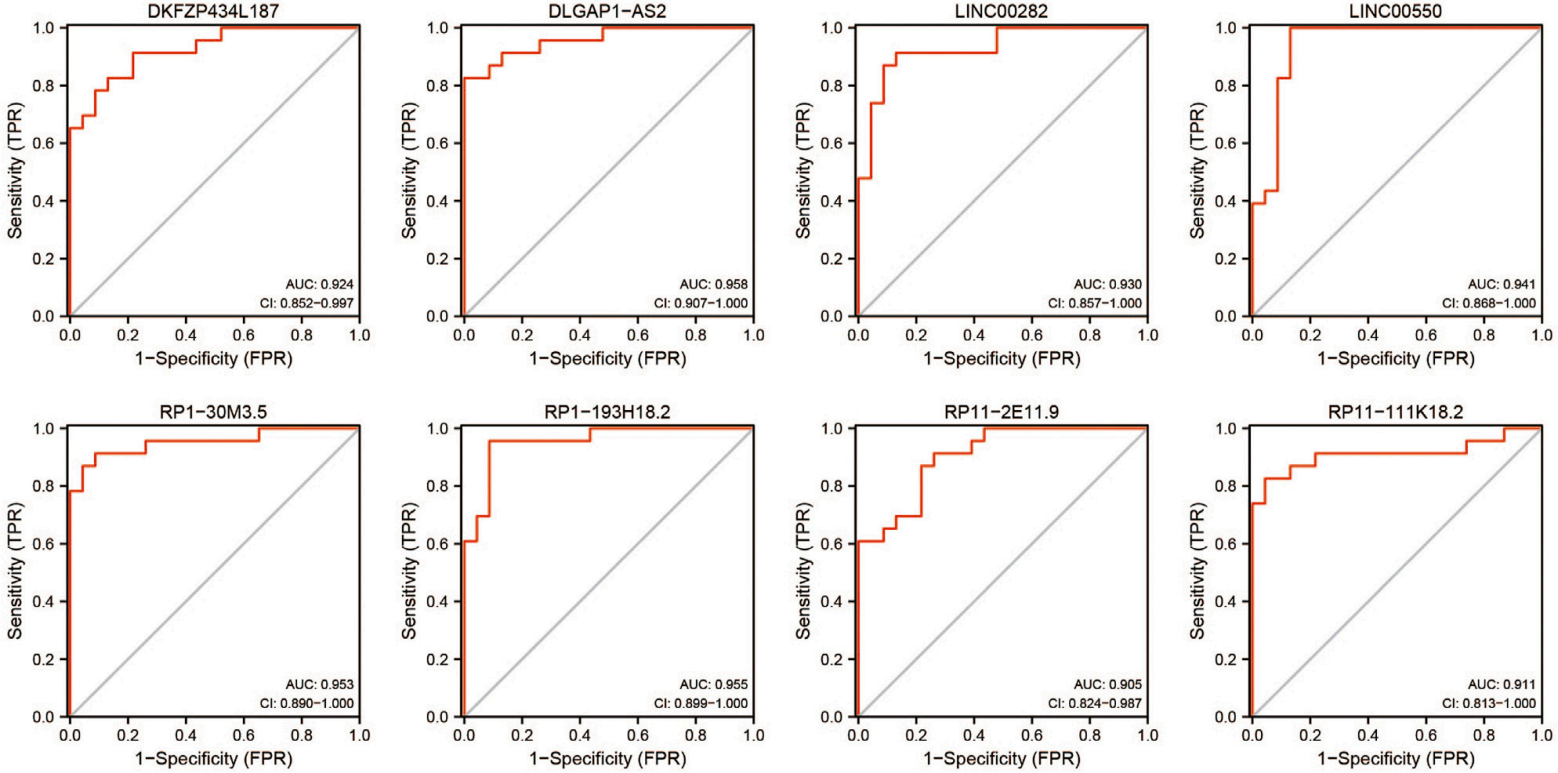
ROC diagnostic curve for lncRNAs in blue modules selected by WGCNA (AUC > 0.90).

This study performed an ROC curve for the 16 lncRNAs for selecting the proper lncRNA to conceive the ceRNA network, and the results are displayed in Figure 7. A total of eight lncRNAs presented high accuracy in diagnosing stroke, with an AUC higher than 0.9. These were DKFZP434L187, DLGAP1-AS2, LINC00282, LINC00550, RP1-30M3.5, RP1-193H18.2, RP11-2E11.9, and RP11-111K18.2, which are candidates for formulating the ceRNA network.

### lncRNA–miRNA–mRNA Network in Stroke

**FIGURE 8:**
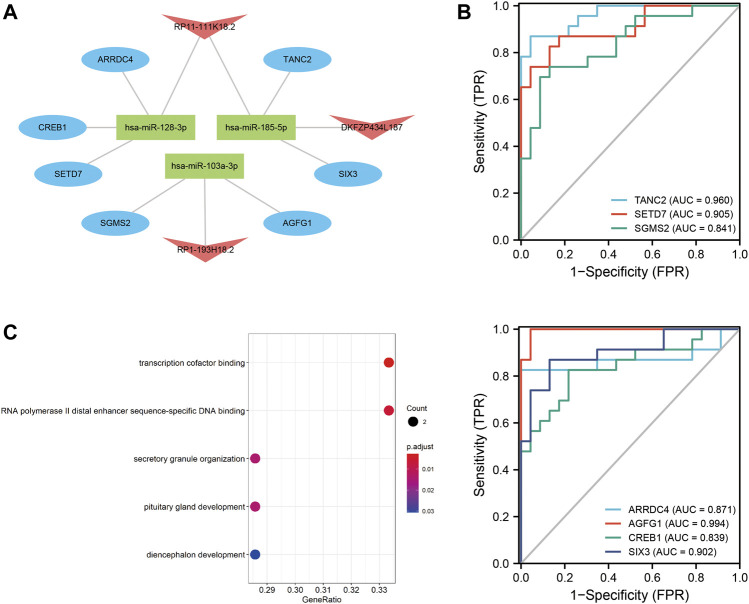
ceRNA network and diagnostic ROC curve for mRNAs and GO enrichment analysis. **(A)**: ceRNA network. Blue ovals denote mRNAs; red arrows denote lncRNAs; and green rectangles denote miRNAs. (B): Diagnostic ROC curve of seven mRNAs in the ceRNA network. **(C)**: Summary of GO functional enrichment analysis on mRNAs.

The lncRNA–miRNA–mRNA ceRNA network was constructed to further investigate the impact of the eight lncRNAs on the mRNA expression. According to Figure 7, all eight lncRNAs had high accuracy to diagnose stroke (AUCs of eight lncRNAs are higher than 0.9); then, we speculated that lncRNAs interact with miRNAs *via* the DIANA-LncBase v2.0 database and obtained four lncRNAs, including two upregulated and two downregulated lncRNAs. MiRNAs targeting mRNAs were speculated by miRWalk 3.0, TargetScan, and miRDB. Combined with mRNAs in Figure 6, a total of 13 mRNAs, including 12 upregulated and one downregulated mRNA, remained. mRNAs and lncRNAs presenting the same tendency to change were selected to form a ceRNA network. Finally, three miRNAs, seven mRNAs, and three lncRNAs constituted the final ceRNA network (Figure 8A). Except for DKFZP434, 187-SIX3 showed a low expression, whereas the remaining lncRNA–mRNA pairs showed a high expression. In the first module, RP11-111K18.2 and four mRNAs competitively combined with two miRNAs (hsa-miR-128-3p and hsa-miR-185-5p). RP1-193H18.2 and two mRNAs scrambled for hsa-miR-103a-3p in the second module, whereas in the third module, DKFZP434L187 and SIX3 struggled for hsa-miR-185-5p. More details on the miRNA–mRNA/lncRNA interactions in the ceRNA network are listed in Table 5.

**TABLE 5:** The pairwise relationships in the lncRNA–miRNA–mRNA ceRNA network.

miRNA	mRNA/lncRNA
hsa-miR-185-5p	RP11-111K18.2
hsa-miR-185-5p	TANC2
hsa-miR-128-3p	RP11-111K18.2
hsa-miR-128-3p	SETD7
hsa-miR-128-3p	CREB1
hsa-miR-128-3p	ARRDC4
hsa-miR-103a-3p	RP1-193H18.2
hsa-miR-103a-3p	SGMS2
hsa-miR-103a-3p	AGFG1

Meanwhile, the diagnostic efficacy of mRNAs in stroke has been discussed. As shown in Figure 8B, TANC2, SETD7, AGFG1, and SIX3 presented high diagnostic accuracy (AUC > 0.9). GO functional enrichment was also administered to mRNAs to understand ceRNA network-related biological processes. As shown in Figure 8C, the ceRNA network primarily participated in transcription cofactor binding, RNA polymerase II distal enhancer sequence-specific DNA binding, and secretory granule organization. More details on the GO analysis can be found in Table 6.

**TABLE 6:** GO enrichment for the mRNAs involved in the ceRNA network.

GO_Category	Terms	Count	*P*.Adjust
BP	GO:0033363∼secretory granule organization	2	1.44E-02
BP	GO:0021983∼pituitary gland development	2	1.44E-02
BP	GO:0021536∼diencephalon development	2	3.07E-02
MF	GO:0001221∼transcription cofactor binding	2	2.75E-03
MF	GO:0000980∼RNA polymerase II distal enhancer sequence-specific DNA binding	2	6.60E-03
MF	GO:0001158∼enhancer sequence-specific DNA binding	2	6.60E-03
MF	GO:0035326∼enhancer binding	2	6.60E-03
MF	GO:0035497∼cAMP response element binding	1	2.71E-02
MF	GO:0001222∼transcription co-repressor binding	1	2.71E-02
MF	GO:0016780∼phosphotransferase activity, for other substituted phosphate groups	1	2.94E-02
MF	GO:0001223∼transcription coactivator binding	1	3.06E-02
MF	GO:0001228∼DNA-binding transcription activator activity, RNA polymerase II-specific	2	3.06E-02
MF	GO:0035035∼histone acetyltransferase binding	1	3.13E-02
MF	GO:0030544∼Hsp70 protein binding	1	3.95E-02
MF	GO:0018024∼histone-lysine N-methyltransferase activity	1	3.95E-02
MF	GO:0001102∼RNA polymerase II activating transcription factor binding	1	4.10E-02
MF	GO:0042054∼histone methyltransferase activity	1	4.10E-02
MF	GO:0016279∼protein-lysine N-methyltransferase activity	1	4.10E-02
MF	GO:0016278∼lysine N-methyltransferase activity	1	4.10E-02
MF	GO:0002039∼p53 binding	1	4.17E-02
MF	GO:0033613∼activating transcription factor binding	1	4.91E-02
MF	GO:0008276∼protein methyltransferase activity	1	4.91E-02

### Verification of the Diagnostic Efficacy of mRNAs and lncRNAs in the ceRNA Network

**FIGURE 9:**
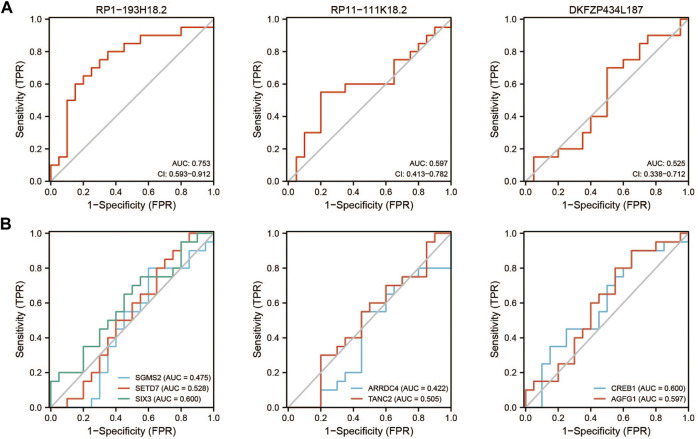
Diagnostic efficacy of GSE22255 for lncRNAs and mRNAs. **(A)**: ROC curve for lncRNAs. **(B)**: ROC curve for mRNAs.

GSE22255 was included in this study as the verification series to validate the diagnostic efficacy of mRNAs and lncRNAs in the ceRNA network. RP1-193H18.2 was more advantageous for diagnosing stroke than the other two lncRNAs, as shown in Figure 9A. No mRNA showed realistic diagnostic efficacy (Figure 9B).

## Discussion

Stroke causes great burden on human health and the economy. Early diagnosis of stroke will greatly increase the opportunity for patients to receive effective post-stroke rehabilitation; therefore, identifying biomarkers that can detect stroke onset early is vital. However, current diagnostic approaches, such as detection of clinical features and neuroimaging, are not timely. Some researchers have demonstrated that the abnormal expression of lncRNAs can be used as a diagnostic or prognostic biomarker for ischemic stroke. MALAT1 and ANRIL (Yang et al., 2018; Tan et al., 2019; Fathy et al., 2021) were previously demonstrated to serve as protective or detrimental predicting factors for stroke under certain circumstances. However, even though these studies have investigated the relationships between stroke and lncRNAs, only one or two lncRNAs were focused on. There is a lack of systematic analysis, and few researchers have paid attention to the interaction between these DElncRNAs. For the first time, we constructed a ceRNA network to comprehensively explore how lncRNAs work in a stroke onset and to ascertain the interactions between DEGs, and we obtained ROC curves to predict the diagnostic functions of DEGs in our network.

Although lncRNAs seem to be promising biomarkers for stroke, the underlying mechanism of how they impact stroke is still unclear. Currently, an increasing number of studies have been conducted on lncRNAs that interact directly with genes as transcriptional regulators. The ANRIL overexpression has been reported to elevate VEGF levels and promote angiogenesis by activating the NF-κB signaling pathway in diabetes mellitus (DM) + cerebral infarction (CI) rats (Zhang et al., 2017), whereas ANRIL knockdown alleviates neuronal apoptosis in CI rats by inhibiting the NF-κB signaling pathway (Zhao et al., 2019). lncRNAs also participate in stroke mechanisms as ceRNAs. One study has demonstrated that inhibition of MEG3 after stroke attenuates hypoxia-induced apoptosis by decreasing its combination with miR-181b and consequently reducing the expression of 12/15-LOX expression (Liu et al., 2016); another study verifies that knockdown of TUG1 promotes cell survival after oxygen-glucose deprivation insults by serving as an miR-9 sponge (Chen et al., 2017). The overexpression of ANRIL can also improve cell survival by downregulating miR-127 and rescuing the expression of Mcl-1 (Liu et al., 2019) or repressing miR-199a-5p and elevating CAV-1 (Zhong et al., 2020). There is great scope for further investigations into the role of lncRNA as ceRNA in stroke.

In this study, we performed bioinformatics analysis to identify DElncRNAs, DEmiRNAs, and DEmRNAs in stroke from the GEO database and successfully constructed a lncRNA–miRNA–mRNA ceRNA network. A total of three lncRNAs (DKFZP434L187, RP1-193H18.2, and RP11-111K18.2), three miRNAs (miR-128-3p, miR-185-5p, and miR-103a-3p), and seven mRNAs (ARRDC4, CREB1, SETD7, SGMS2, TANC2, SIX3, and AGFG1) were involved in this network. RP1-193H18.2 had greater diagnostic efficacy for stroke than the other two lncRNAs, whereas no selected mRNA presented realistic diagnostic efficacy. Among the three selected lncRNAs, DKFZP434L187 has been detected as a promising biomarker for hepatocellular carcinoma (Sun et al., 2019), and one patent has reported that it may associate with stroke (Sharp et al., 2017). As for miRNAs, miR-185-5p is primarily involved in the process of proliferation and metastasis of tumors (Zhang et al., 2020; Bagheri et al., 2021; Sun et al., 2021; Sur et al., 2021; Zhang et al., 2021); one research has reported that it may participate in ischemic-reperfusion (I/R) injury by regulating nitric oxide synthase 2 (NOS2) (Wang et al., 2016). One study suggested that miR-103a-3p levels decreased after I/R injury, and the overexpression of miR-103a-3p alleviated apoptosis and inflammation after I/R by targeting HMGB1, indicating a possible protective function of miR-103a-3p in IS (Li et al., 2020b). MiR-128-3p is significantly related to I/R injury and IS, and one study found that miR-128-3p is elevated in the cerebrospinal fluid 3 days after AIS. Furthermore, its expression may reflect brain damage (Sørensen et al., 2017). However, whether this elevation is causative or correlative is unknown. Another study demonstrated that inhibition of miR-128-3p alleviates apoptosis after I/R both *in vitro* and *in vivo* (Yan et al., 2020). However, Mao et al. (Mao et al., 2017) found that miR-128-3p contributes to neuronal survival after ischemia-induced brain injury by downregulating the expression of the pro-apoptotic protein, p38α; thus, the relationship between miR-128-3p and apoptosis in stroke remains unclear. In addition to I/R injury in stroke, miR-128-3p is also involved in I/R injury in the heart (Ma et al., 2018), liver (Mou et al., 2020), and spinal cord (Wang et al., 2020b). Considering that I/R injury in different organs may share some similar characteristics, miR-128-3p may have a more profound relationship with stroke injury.

Among the seven selected mRNAs, CREB1 and SIX3 were of the greatest importance. CREB1 has been reported to interact with the brain-derived neurotrophic factor (BDNF) and is involved in IS (Shi et al., 2020). One study has demonstrated that CREB1 is the target of miR-128 after stroke, and inhibition of miR-128 by ARPP21 leads to decreased neuronal apoptosis and promoted neurological function repair with the upregulation of CREB1 and BDNF (Chai et al., 2021). Combined with our findings, RP11-111K18.2 may serve as a promising sponge for miR-128-3p and protect against stroke by elevating the CREB1 expression. SIX3 is primarily involved in neurogenesis during embryonic development. SIX3 can participate in the process of normal vertebrate forebrain formation by repressing Wnt1 within the anterior neuroectoderm (Lagutin et al., 2003) or by facilitating cellular proliferation by sequestration of geminin from Cdt1 (Ando et al., 2005). SIX3 is a key component that balances the equilibrium between proliferation and differentiation during neurogenesis as well (Appolloni et al., 2008). In the postnatal stages of brain development, SIX3 is necessary for ependymal cell maturation and consequently ensures the normal process of neurogenesis and neuroblast migration (Lavado and Oliver, 2011). SIX3 is also vital for neuroretinal development (Samuel et al., 2016; Liu and Cvekl, 2017; Takata et al., 2017; Diacou et al., 2018) and striatum neuron formation (Song et al., 2021; Yang et al., 2021). However, it is unclear whether SIX3 functions in neuronal repair after stroke; further research should, therefore, be conducted to confirm this.

In this study, we identified AIS-related lncRNAs, miRNAs, and mRNAs in the ceRNA network. However, this was a pure bioinformatics study without any experimental demonstrations, and the relationship between the ceRNA network and AIS remains unclear. Thus, further experiments and clinical practice are needed to explore how the ceRNA network functions in AIS. First, the basic expression of these predicted DEGs after the onset of stroke, both at the transcriptional and translational levels, should be further examined quantitatively, and their interaction should be verified *via* immunohistochemistry or immunofluorescence assays directly. Second, the relationship between the DEGs and phenotypes should be demonstrated by loss- and gain-of-function studies; possible pathways need to be identified to elucidate the mechanism more deeply. Meanwhile, the co-expression and interaction of DEGs are of great interest and await investigation.

In conclusion, our research provided a reliable comprehensive analysis by analyzing datasets GSE58294 and GSE110993 (further confirmed with GSE22255) to investigate the DEGs related to stroke onset. In total, 2068 DEmRNAs, 199 DElncRNAs, and 96 DEmiRNAs were identified, and we constructed a ceRNA network with 3 DElncRNAs, 3 DEmiRNAs, and 7 DEmRNAs. GSE22255 verified that RP1-193H18.2 may serve as a novel potential target for stroke diagnosis. In the future, the AIS-related ceRNA network should be completed with improved databases, optimization of algorithms, and increased experimental verification. These results may facilitate the development of novel diagnostic and treatment strategies for AIS.

## Conclusion

In this study, we identified DEGs and constructed a lncRNA–miRNA–mRNA ceRNA network by analyzing the interactions and biological functions of these DEGs. Our network identified three lncRNAs, seven mRNAs, and three miRNAs that are closely related to stroke and might serve as promising diagnostic biomarkers. However, the specific pathogenesis and molecular targets still need to be further confirmed through molecular experiments.

## Data Availability

The datasets presented in this study can be found in online repositories. The names of the repository/repositories and accession number(s) can be found in the article/Supplementary Material.
