# Supplementary figures and images for: Comprehensive Analysis of Hub Genes Associated With Competing Endogenous RNA Networks in Stroke Using Bioinformatics Analysis
Source: Front Genet. 2022 Jan 12;12:779923. doi: 10.3389/fgene.2021.779923 (PMC8790239; doi:10.3389/fgene.2021.779923)

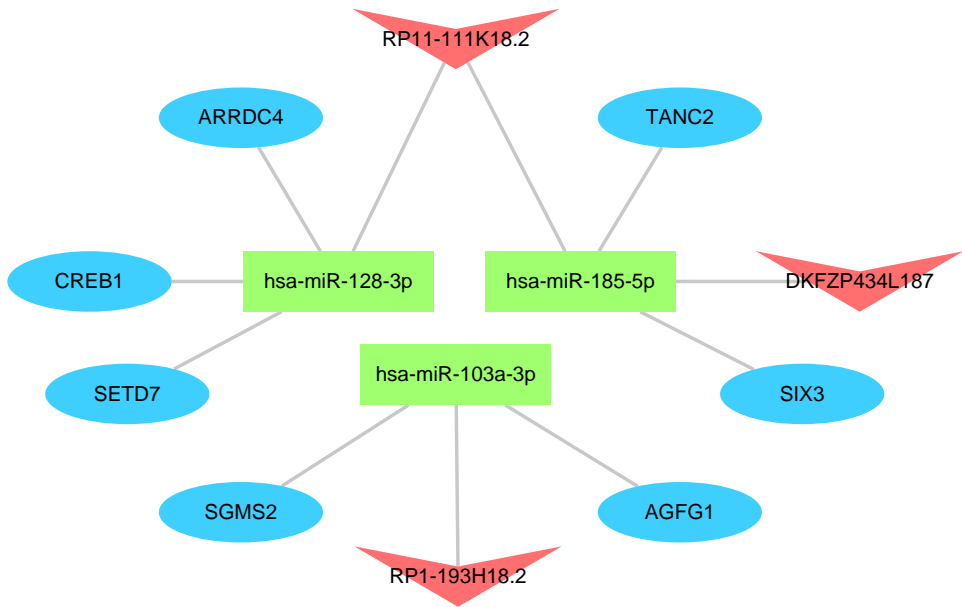

Supplement: Supplementary file 2 [file DataSheet8.ZIP › Figure 8--raw data/ceRNA_network/ceRNA network.pdf]

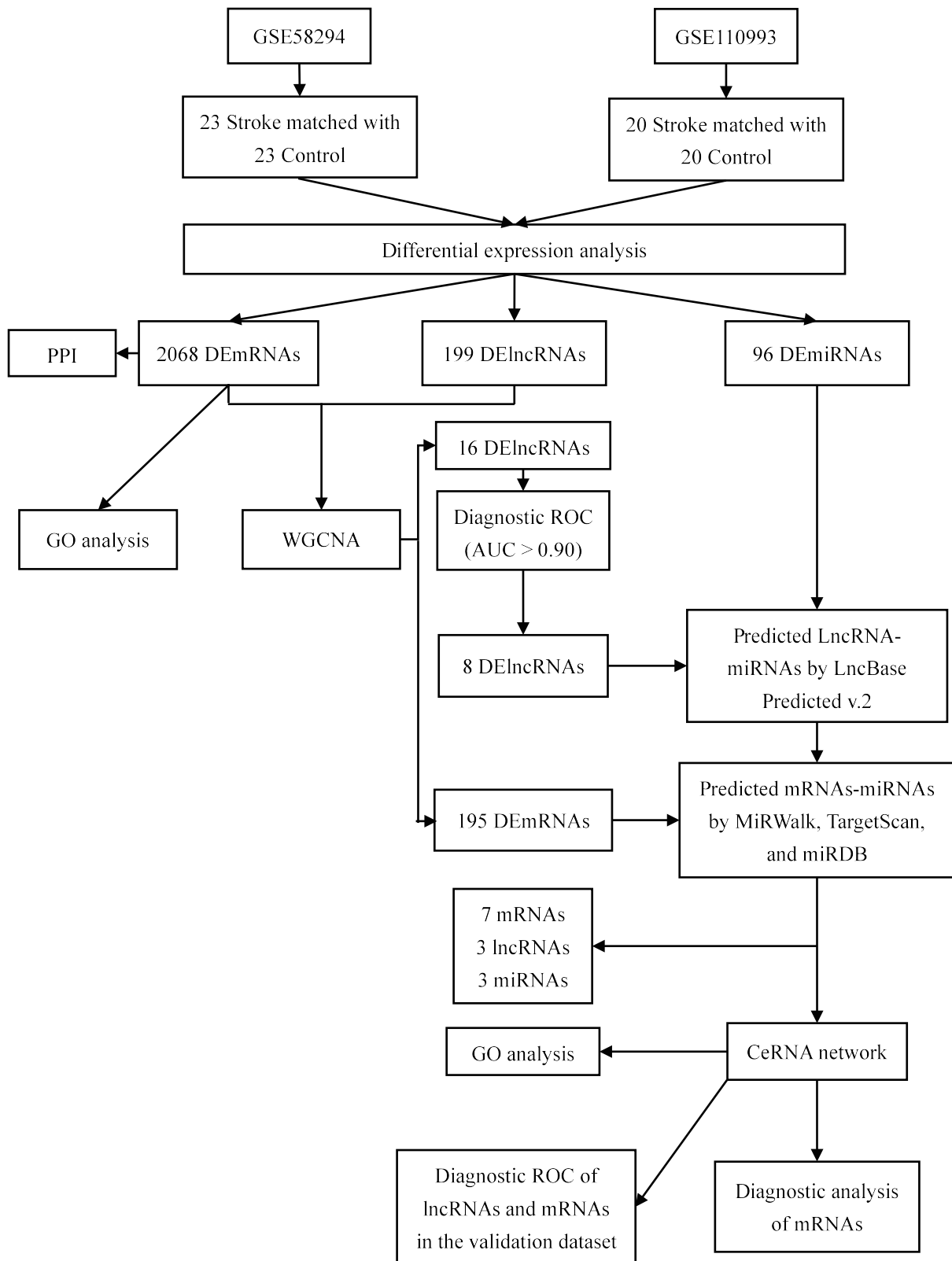

Supplement: Supplementary file 6 [file DataSheet1.ZIP › Supplementary Material Presentation-figures/Figure 1.pdf]

A

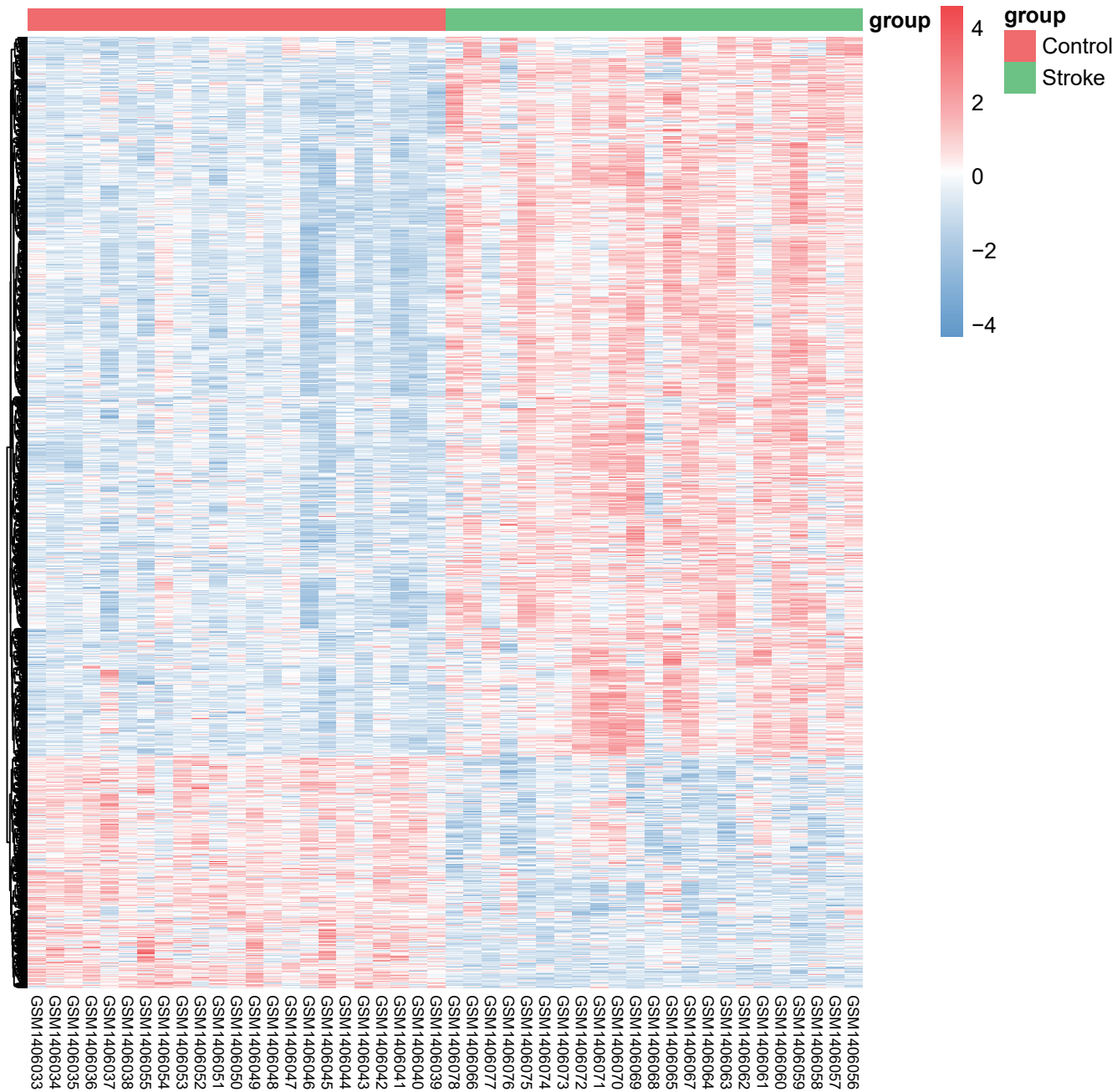

B

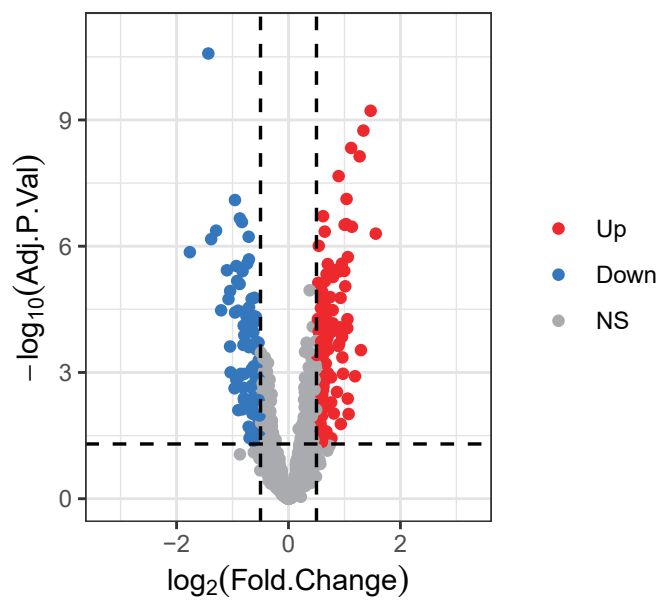

C

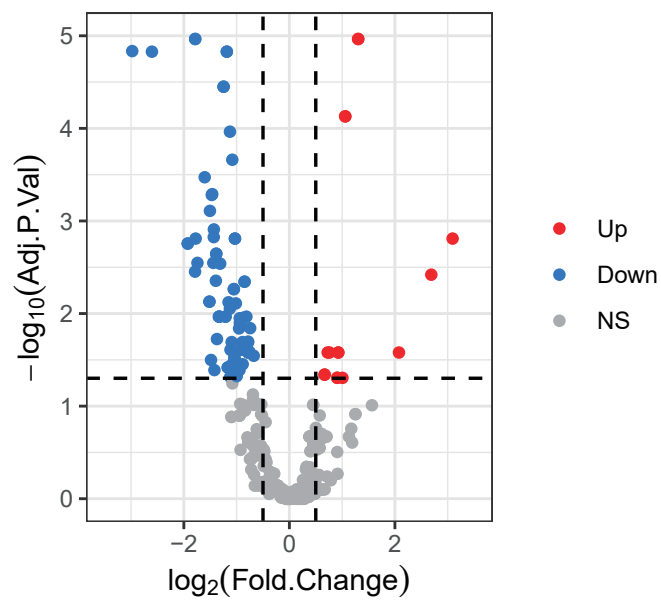

Supplement: Supplementary file 6 [file DataSheet1.ZIP › Supplementary Material Presentation-figures/Figure 2.pdf]

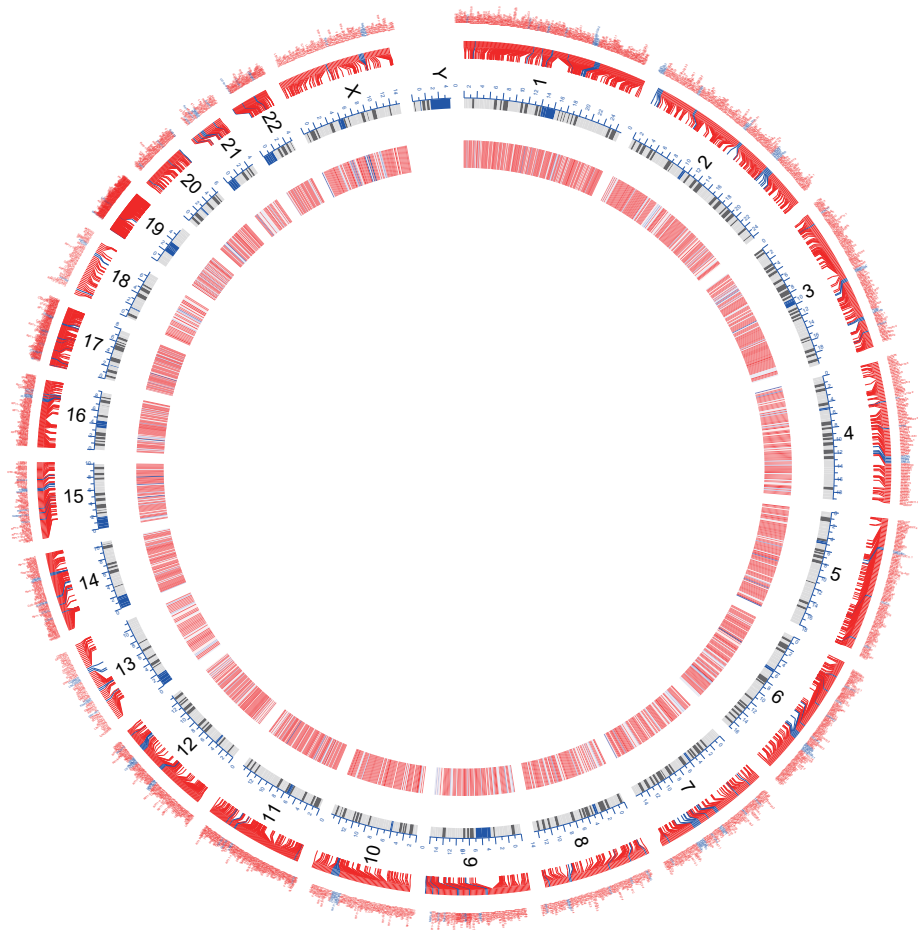

Supplement: Supplementary file 6 [file DataSheet1.ZIP › Supplementary Material Presentation-figures/Figure 3.pdf]

A

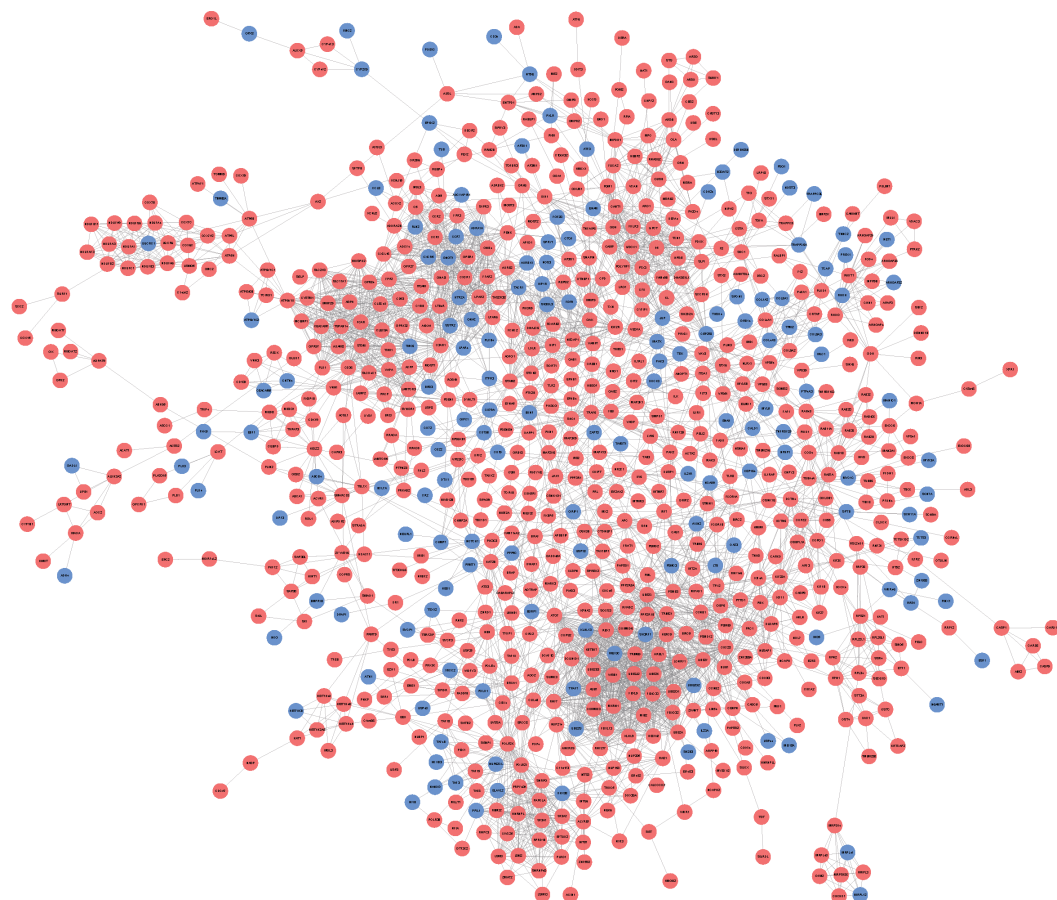

B

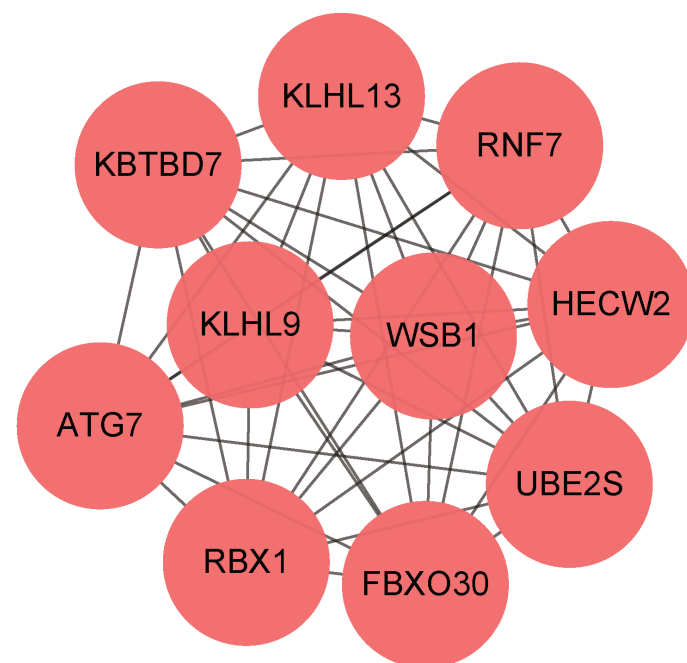

C

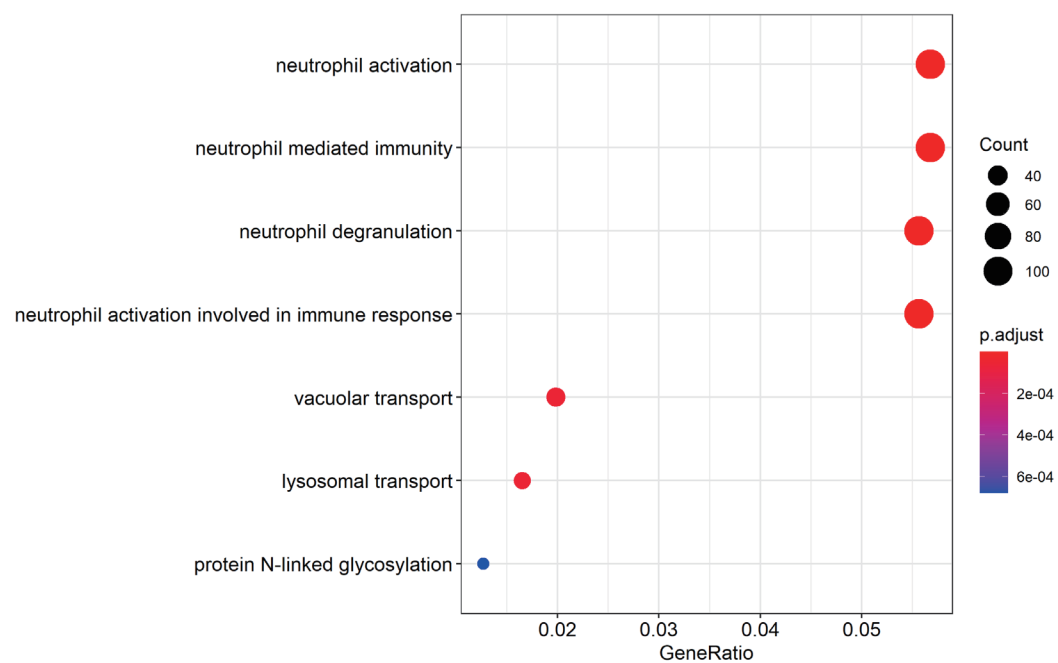

Supplement: Supplementary file 6 [file DataSheet1.ZIP › Supplementary Material Presentation-figures/Figure 4.pdf]

B

## Module-trait relationships

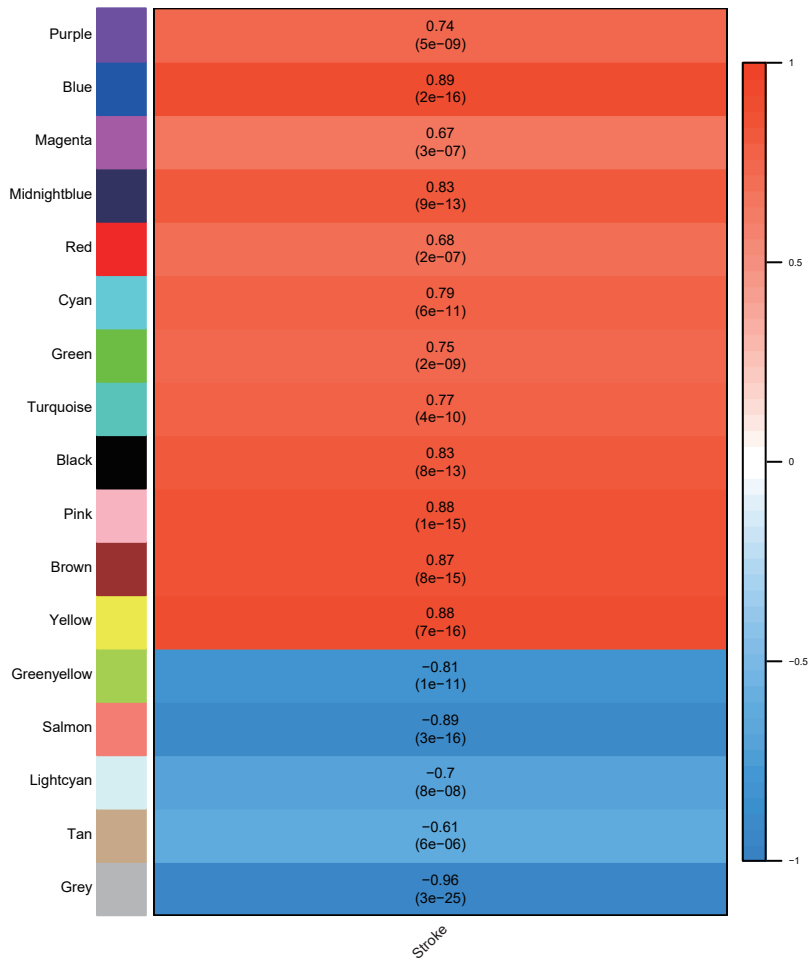

B

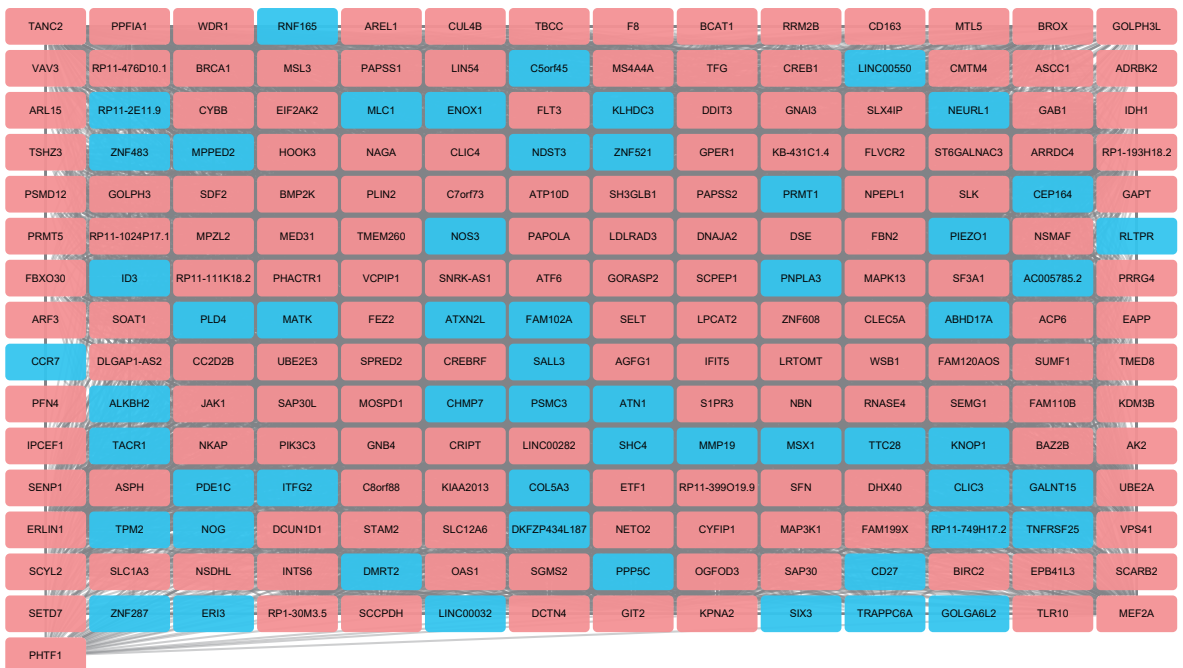

Supplement: Supplementary file 6 [file DataSheet1.ZIP › Supplementary Material Presentation-figures/Figure 6.pdf]

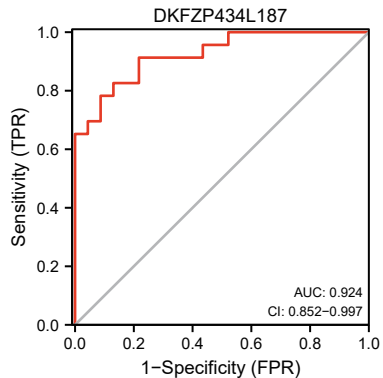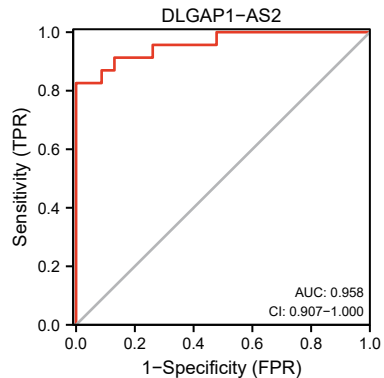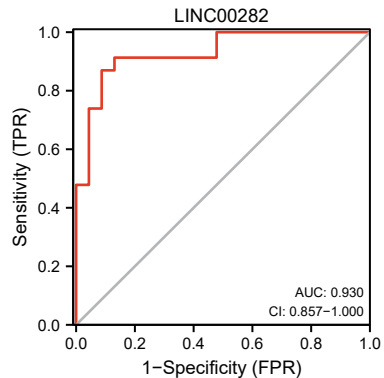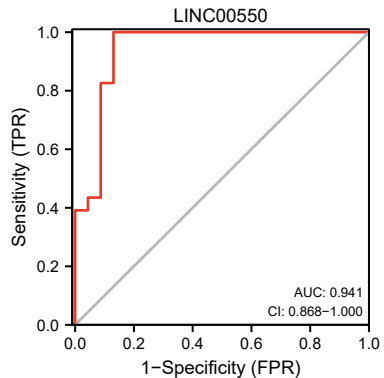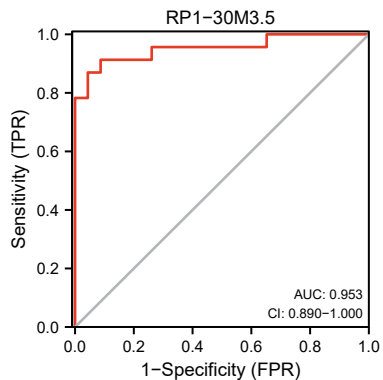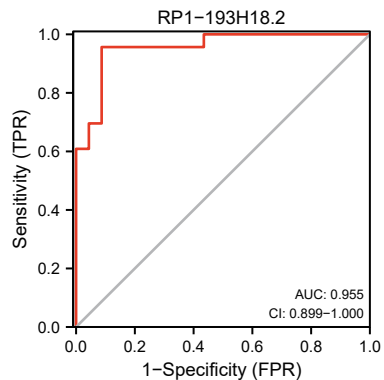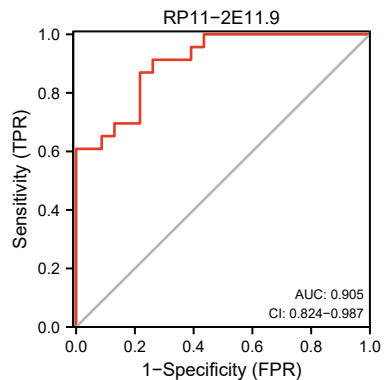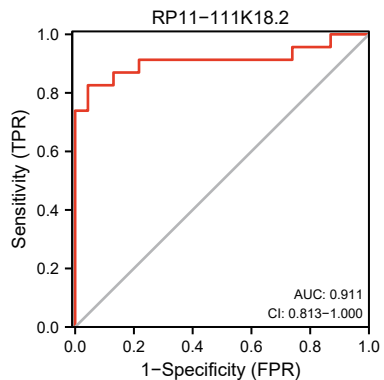

Supplement: Supplementary file 6 [file DataSheet1.ZIP › Supplementary Material Presentation-figures/Figure 7.pdf]

A

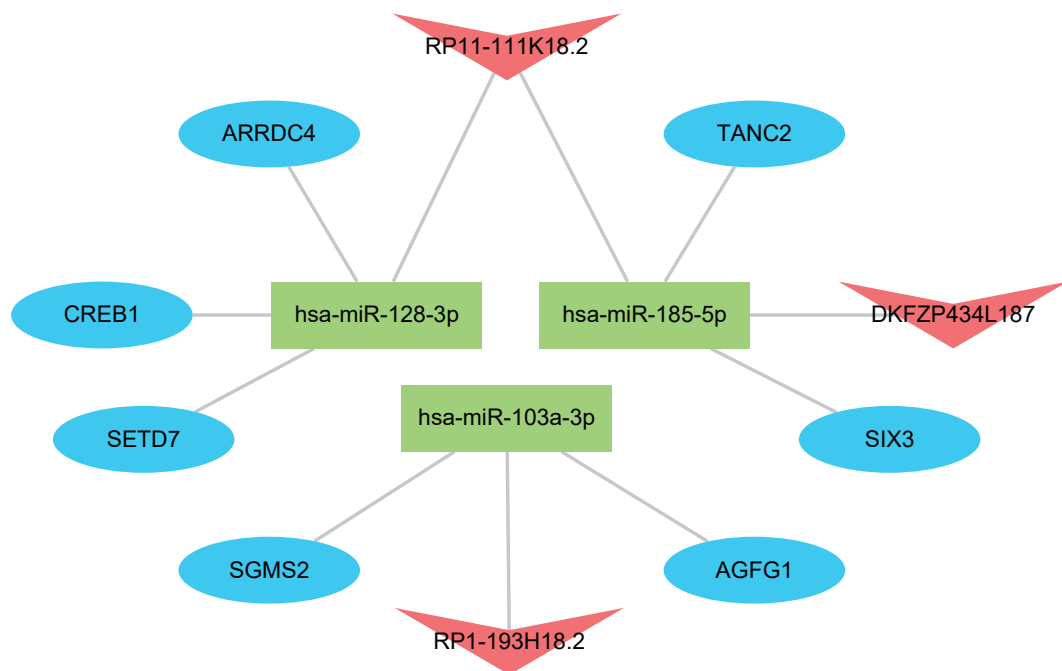

B

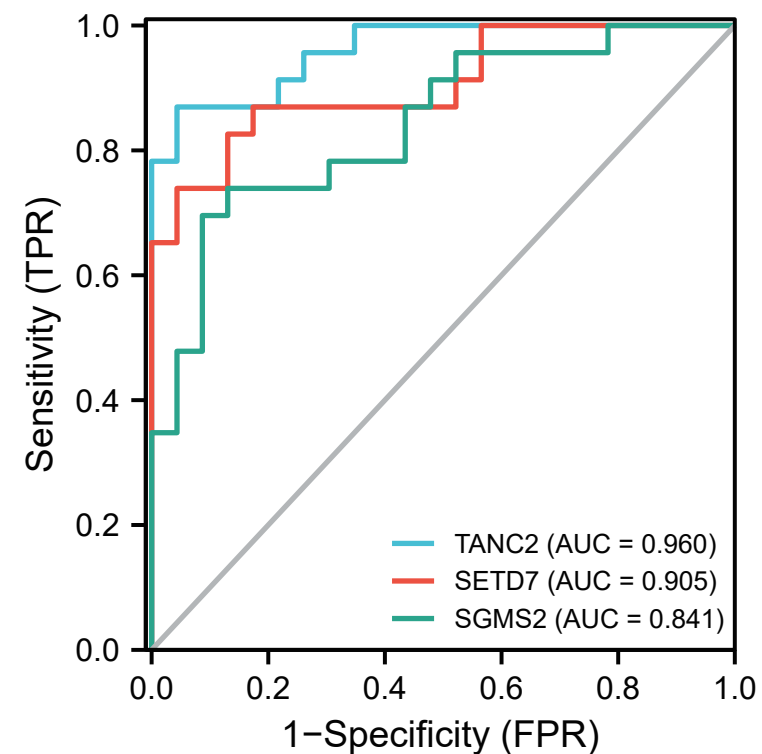

C

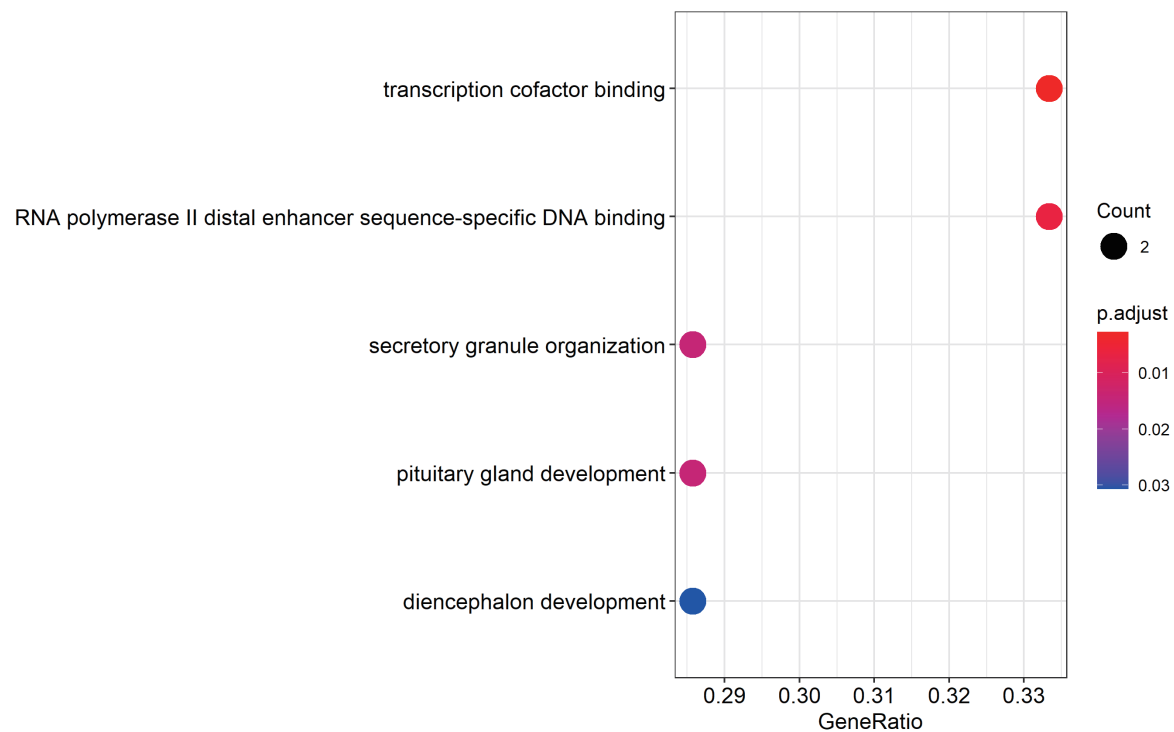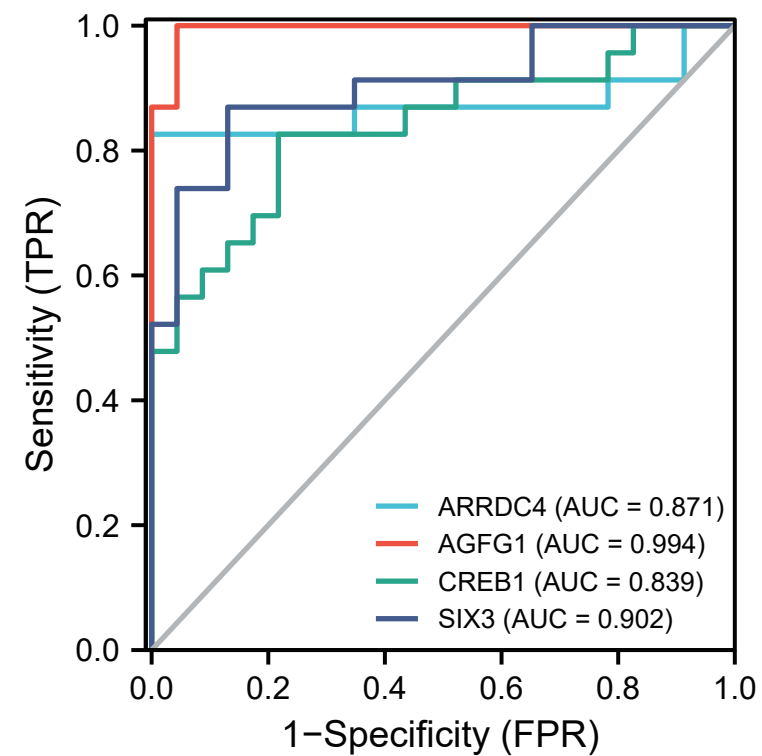

Supplement: Supplementary file 6 [file DataSheet1.ZIP › Supplementary Material Presentation-figures/Figure 8.pdf]

A

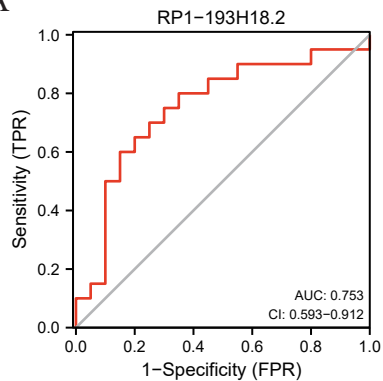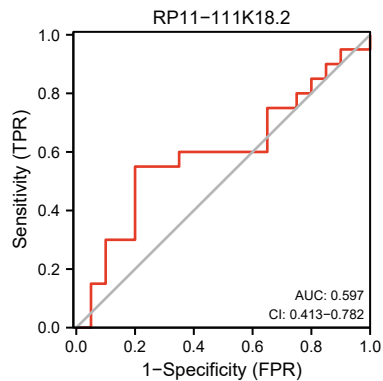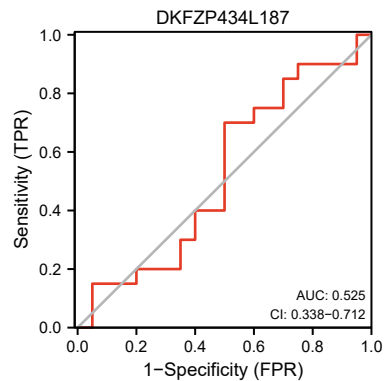

B

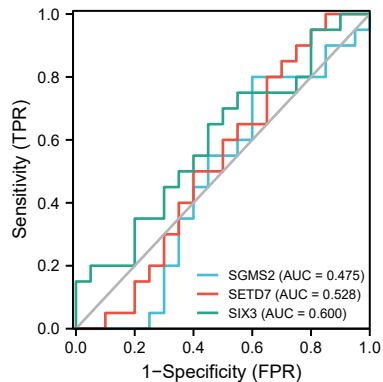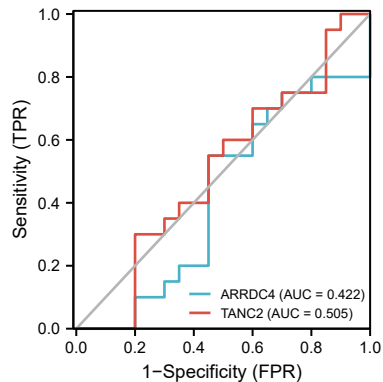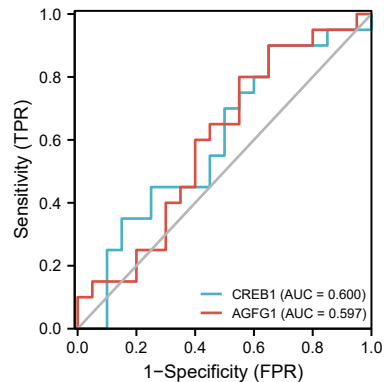

Supplement: Supplementary file 6 [file DataSheet1.ZIP › Supplementary Material Presentation-figures/Figure 9.pdf]

# Gene dendrogram and module colors

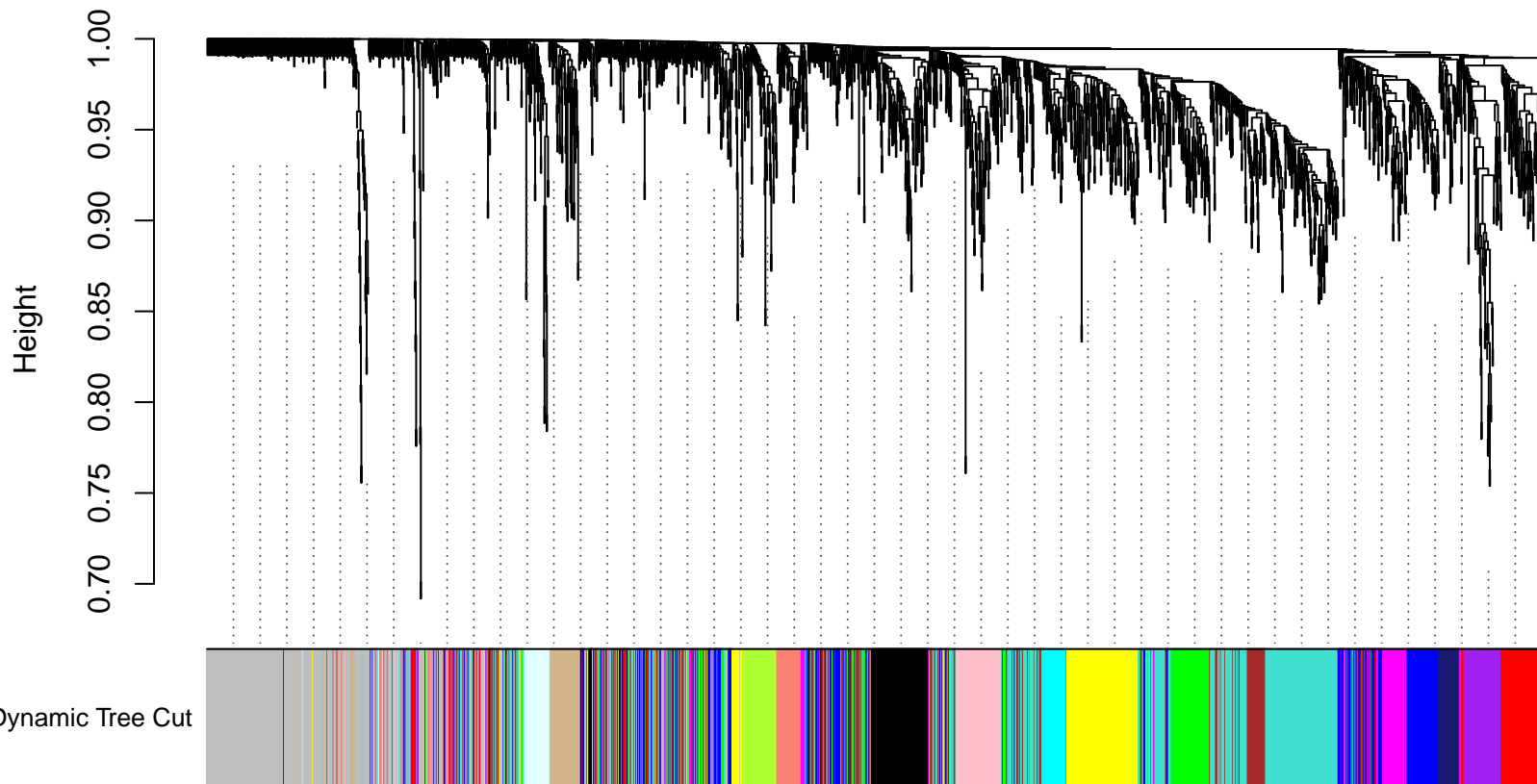

Supplement: Supplementary file 7 [file DataSheet6.ZIP › Figure 5-6--raw data/WGCNA/figure/DynamicTreeCut.pdf]

# Gene clustering on TOM-based

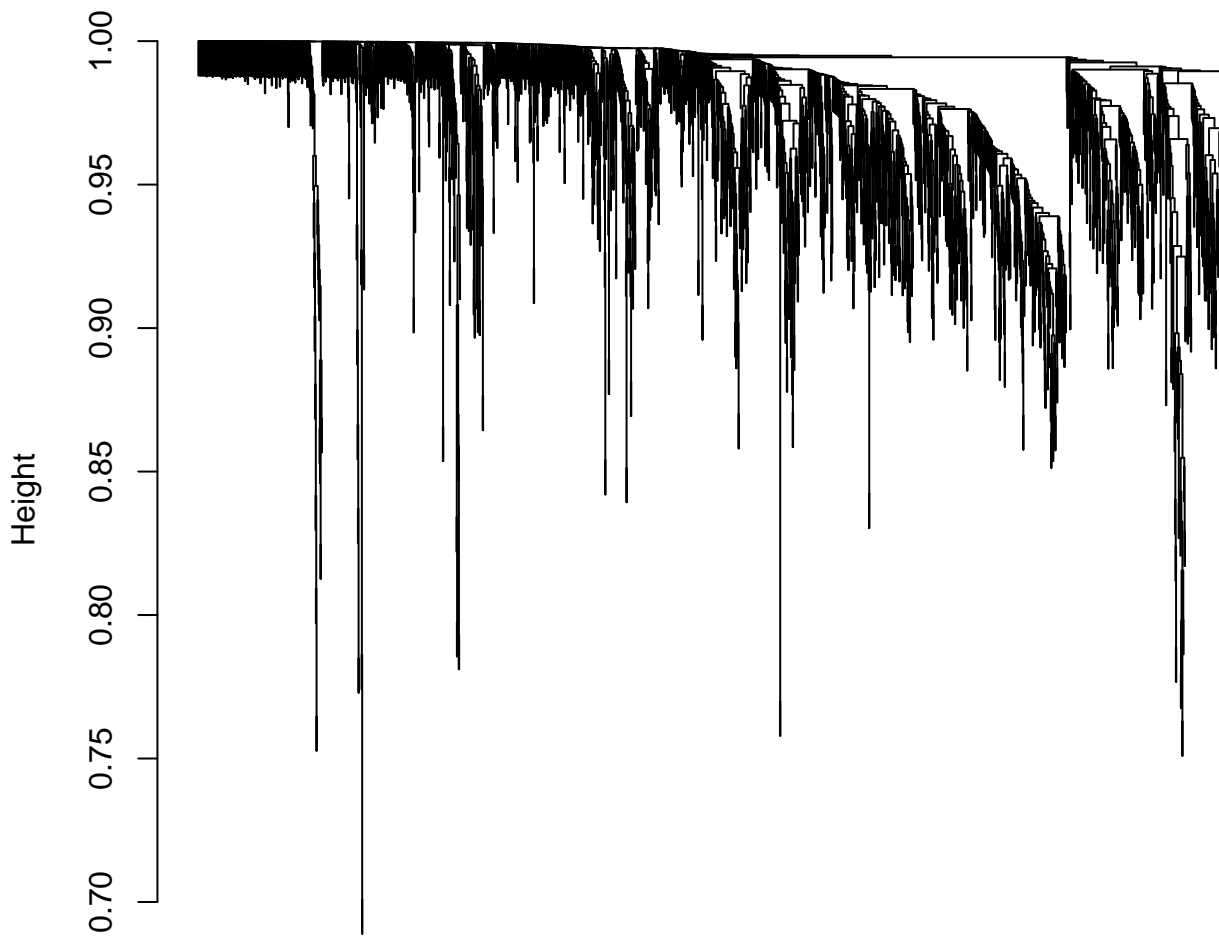

Supplement: Supplementary file 7 [file DataSheet6.ZIP › Figure 5-6--raw data/WGCNA/figure/GeneClusterTOM-based.pdf]

**Clustering of ME before combined**

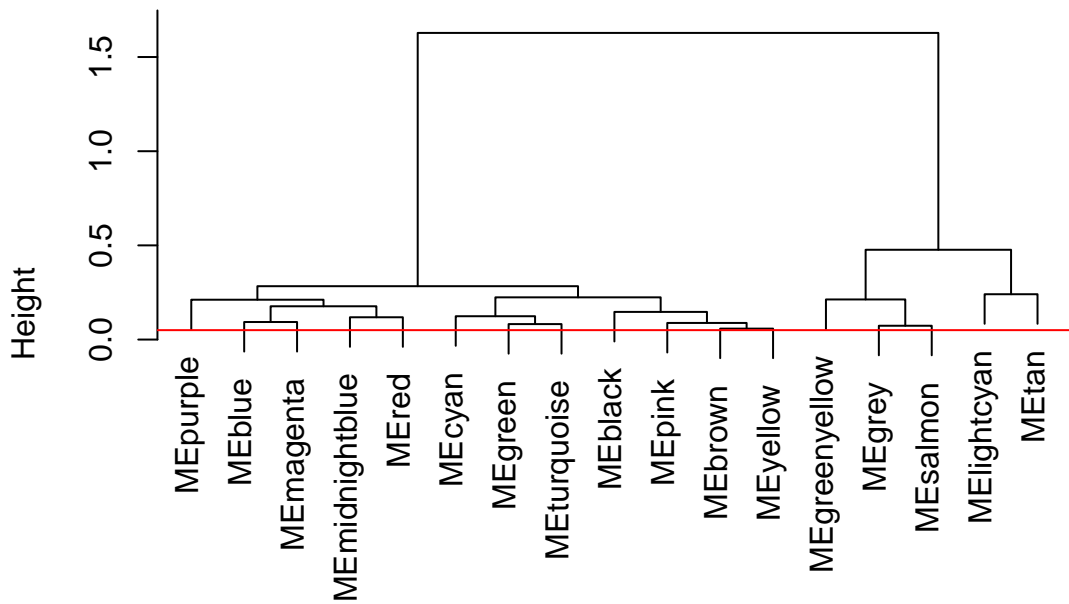

**Clustering of ME after combined**

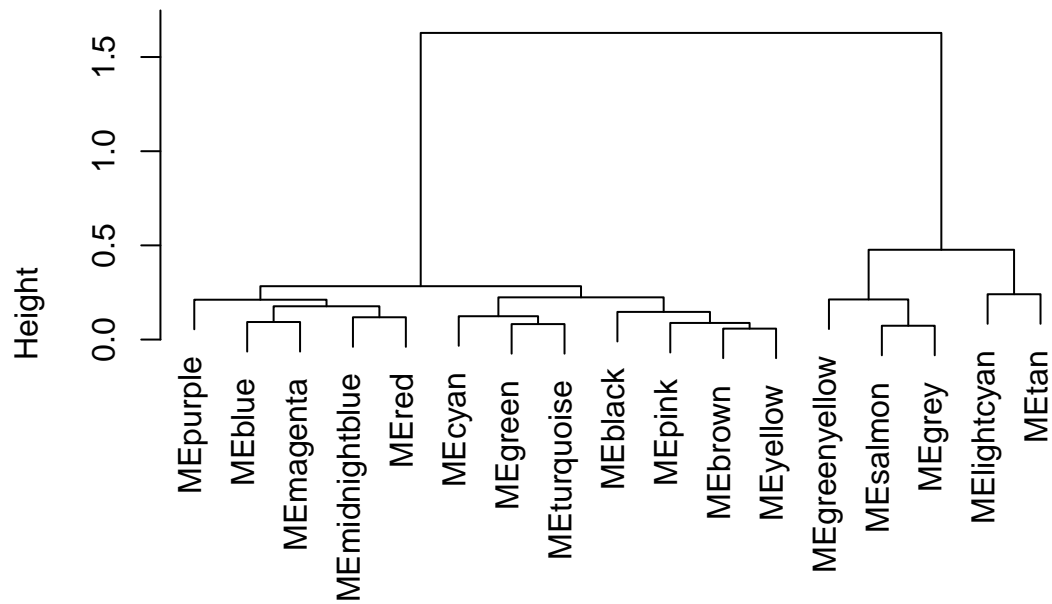

Supplement: Supplementary file 7 [file DataSheet6.ZIP › Figure 5-6--raw data/WGCNA/figure/MECombined.pdf]

# Gene Dendrogram and module colors

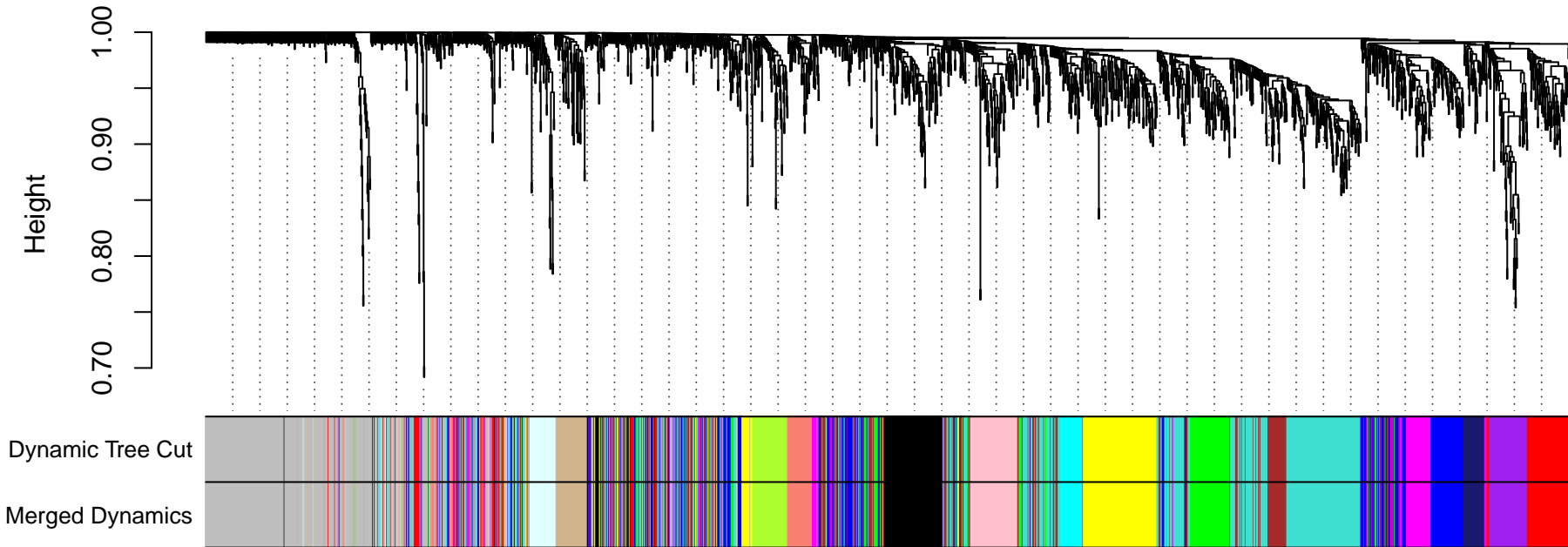

Supplement: Supplementary file 7 [file DataSheet6.ZIP › Figure 5-6--raw data/WGCNA/figure/MergedDynamics.pdf]

# Cluster Dendrogram

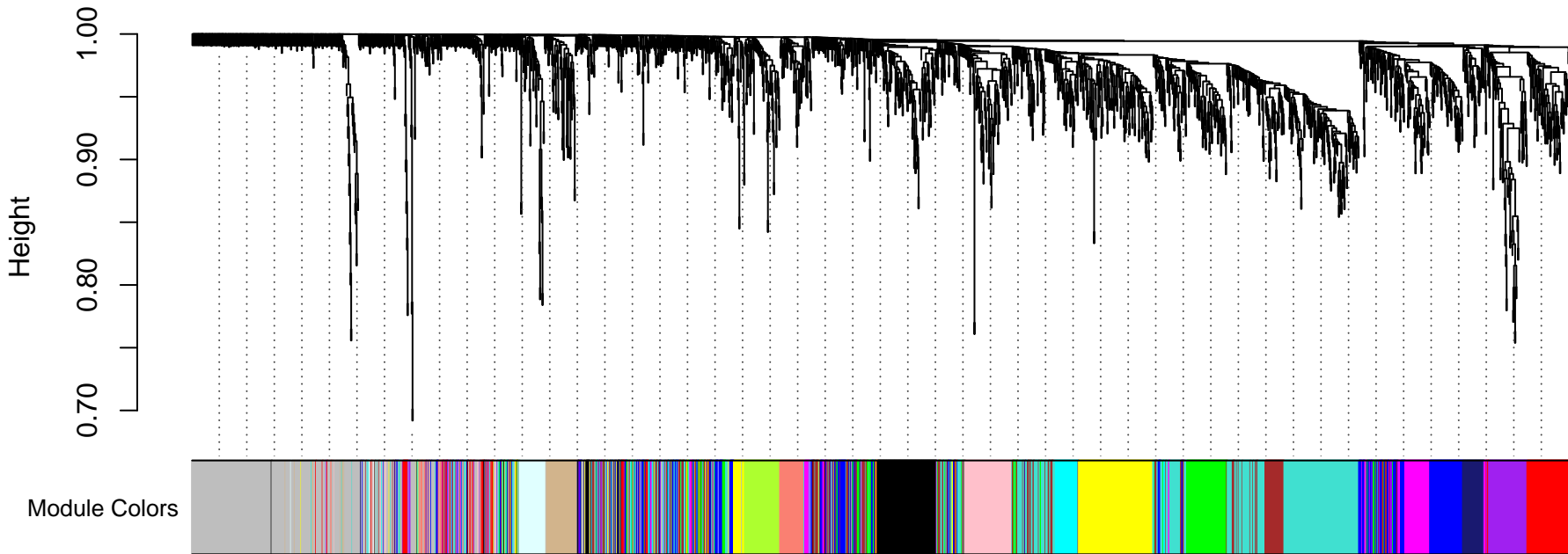

Supplement: Supplementary file 7 [file DataSheet6.ZIP › Figure 5-6--raw data/WGCNA/figure/MergedDynamics_final.pdf]

**Module membership vs. gene significance**  
**cor=0.84, p=9.7e-59**

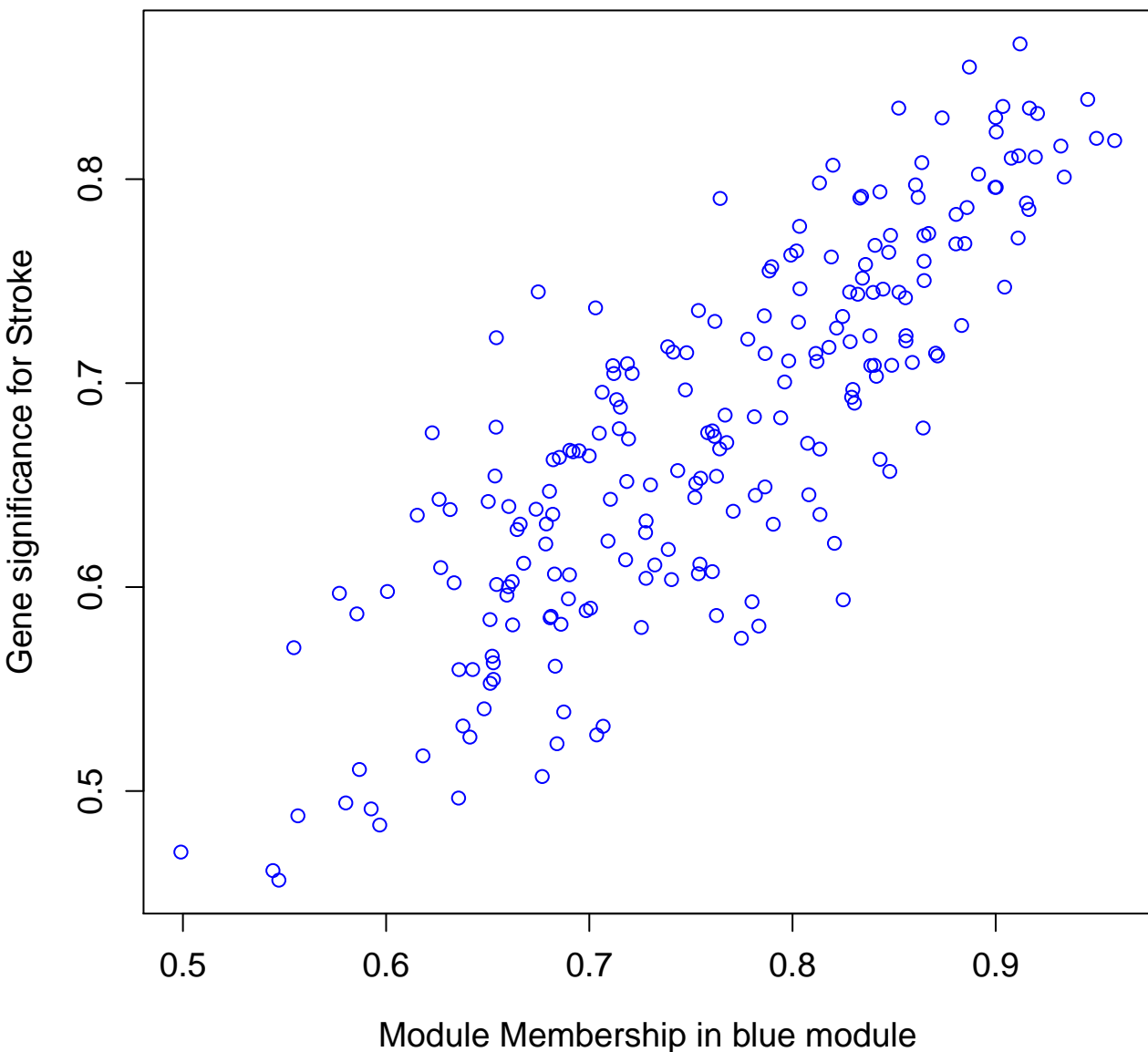

Supplement: Supplementary file 7 [file DataSheet6.ZIP › Figure 5-6--raw data/WGCNA/figure/Module_membership_vs_gene_significance.pdf]

# Module–trait relationships

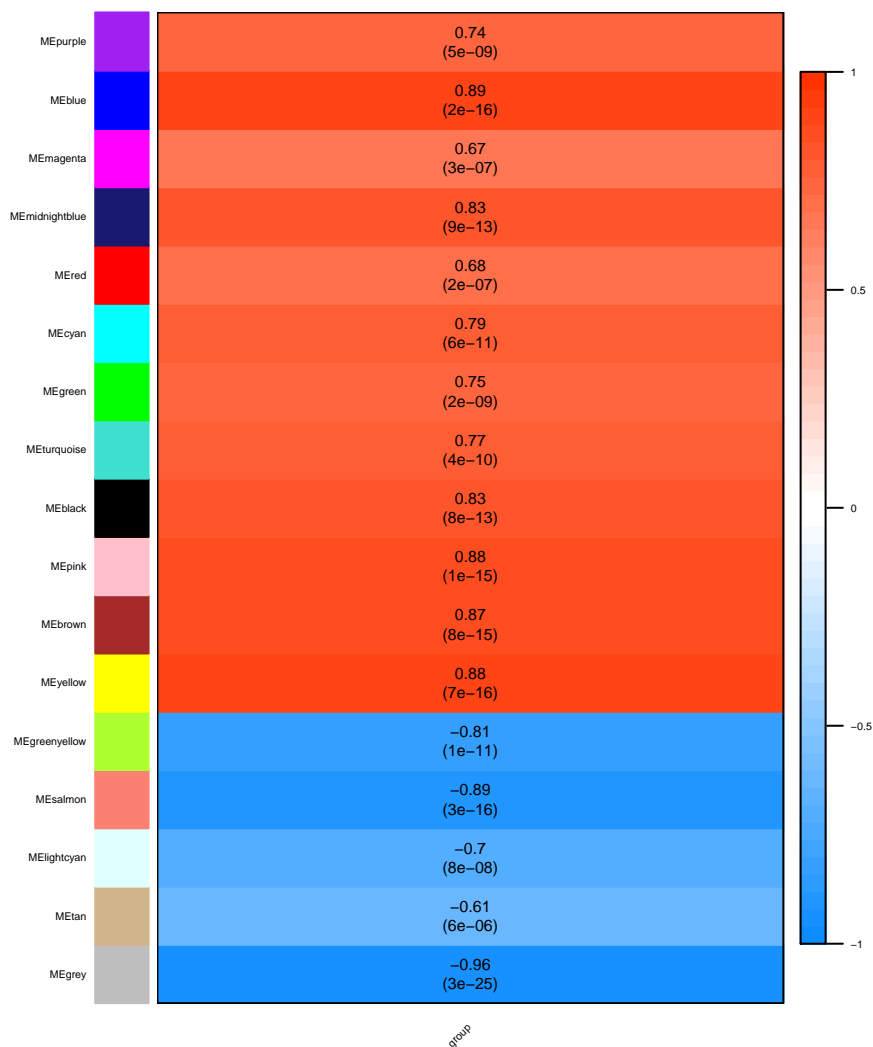

Supplement: Supplementary file 7 [file DataSheet6.ZIP › Figure 5-6--raw data/WGCNA/figure/Module_trait_relationships.pdf]

Sample dendrogram and trait heatmap

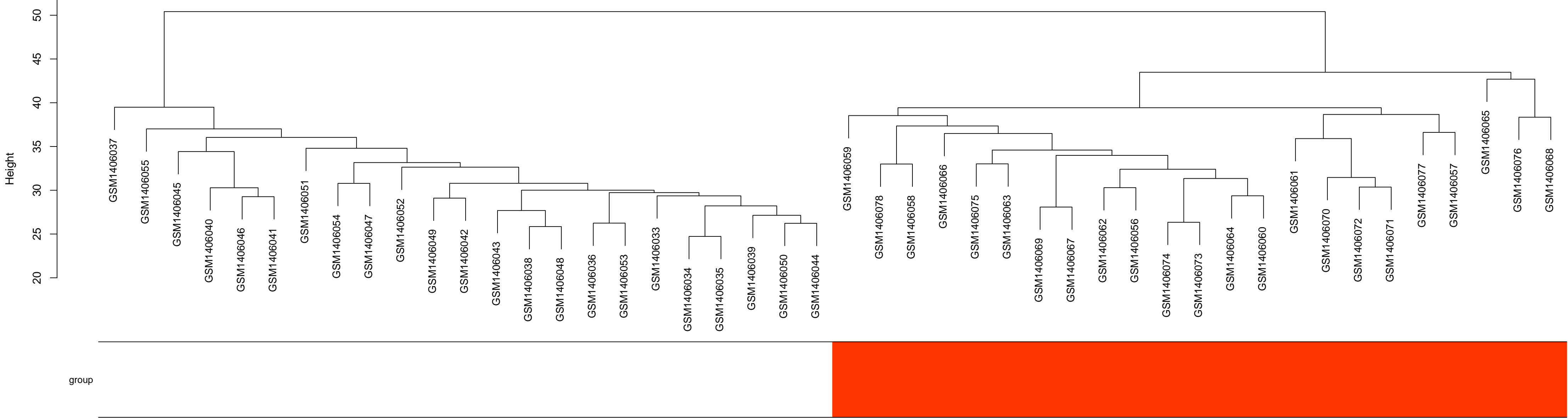

Supplement: Supplementary file 7 [file DataSheet6.ZIP › Figure 5-6--raw data/WGCNA/figure/Sample_dendrogram_and_trait_heatmap.pdf]

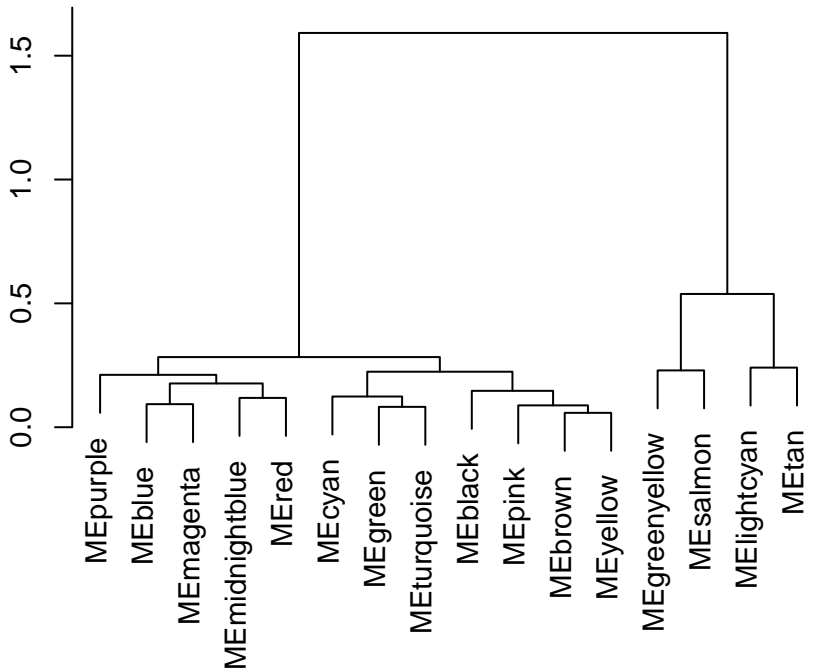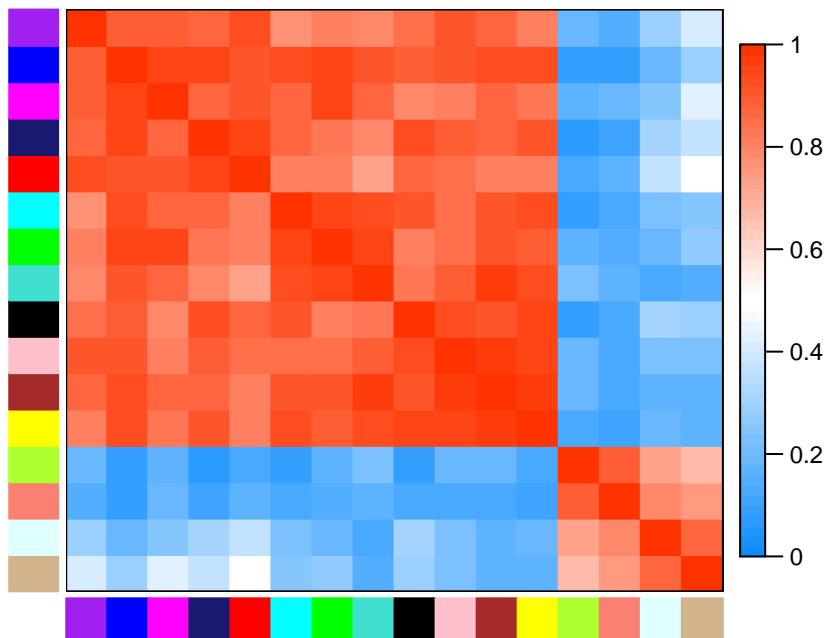

Supplement: Supplementary file 7 [file DataSheet6.ZIP › Figure 5-6--raw data/WGCNA/figure/moduleCor.pdf]

Histogram of k

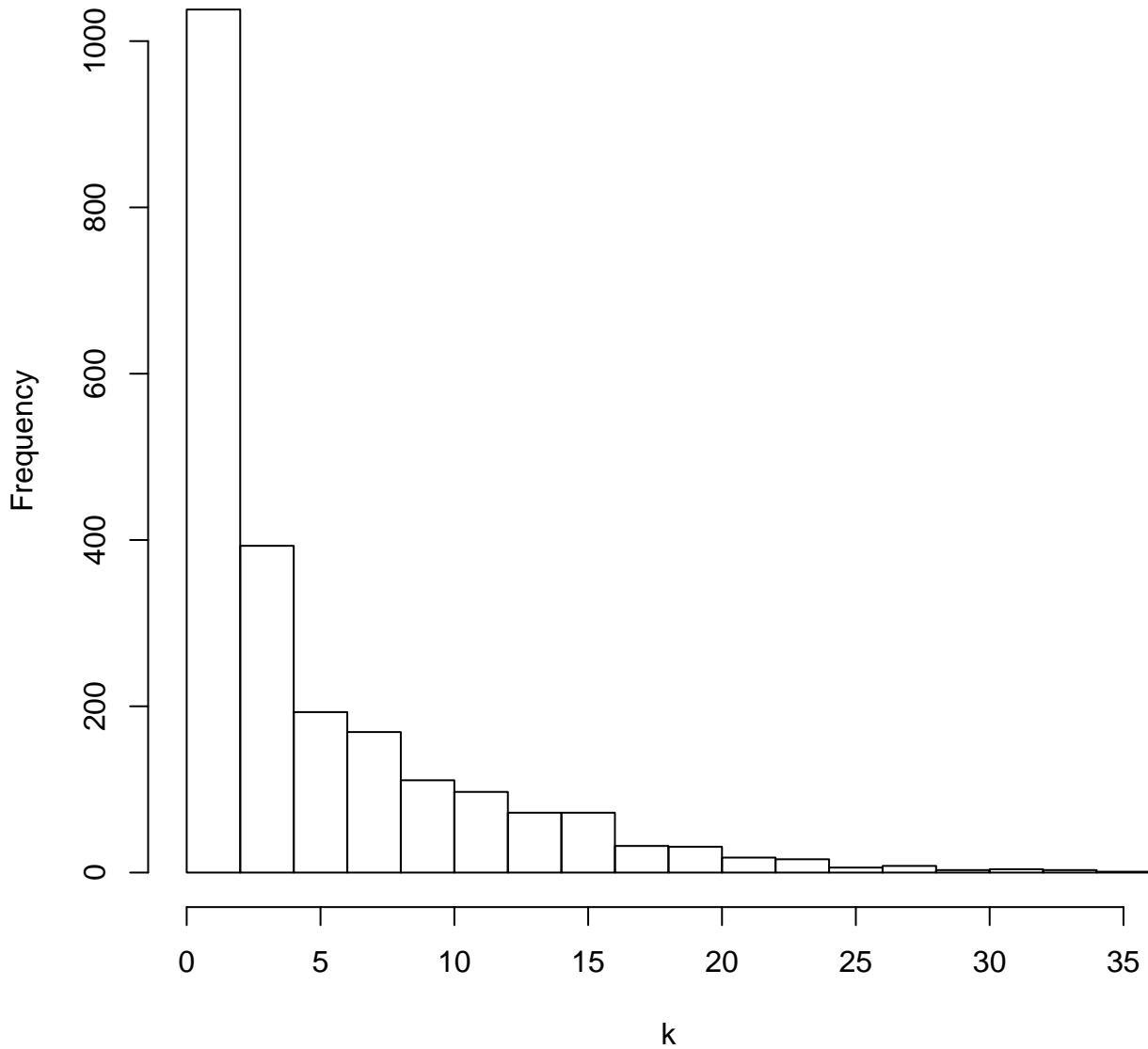

Check scale free topology scale  $R^2= 0.88$  , slope=  $-1.99$

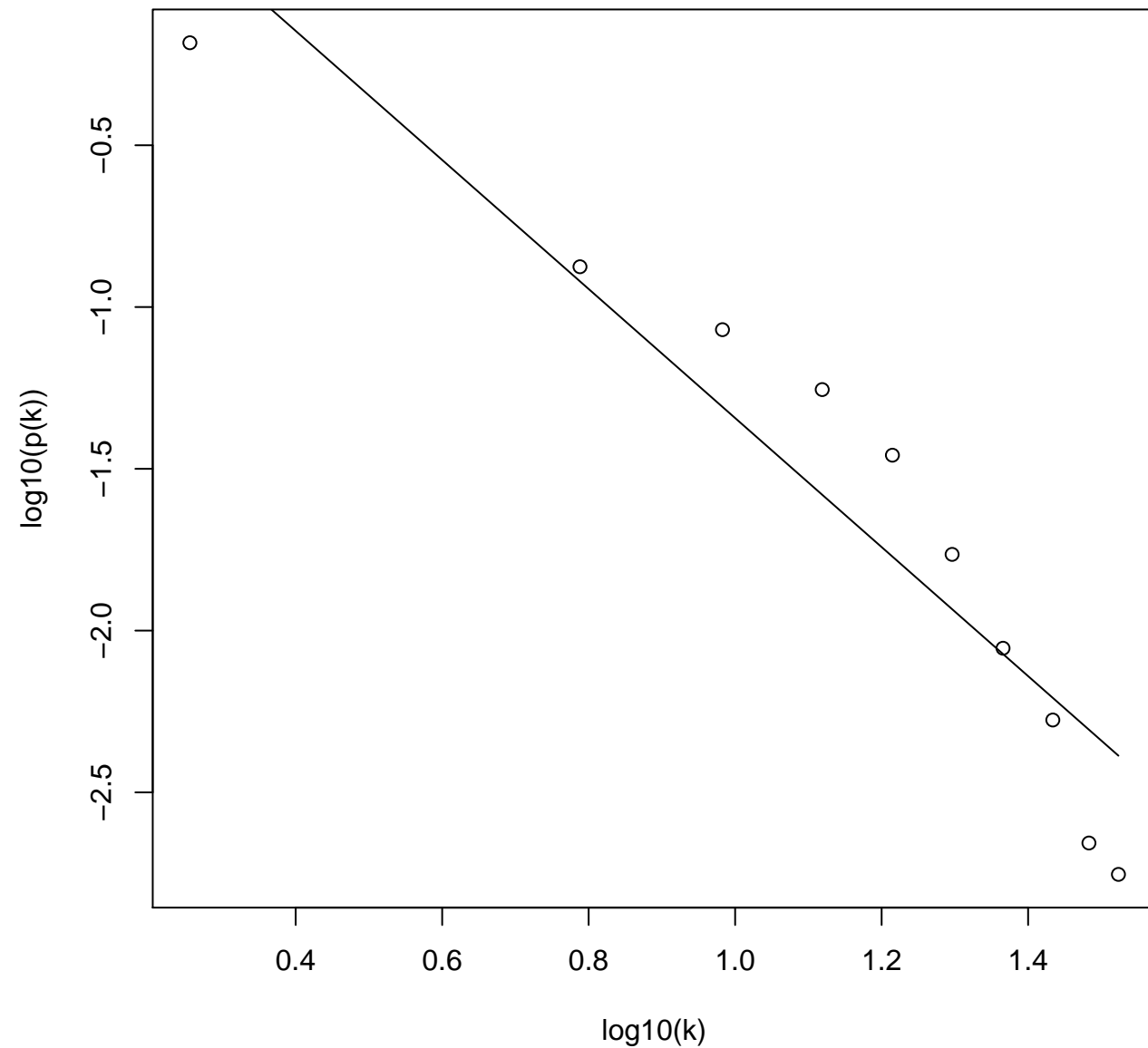

Supplement: Supplementary file 7 [file DataSheet6.ZIP › Figure 5-6--raw data/WGCNA/figure/scaleFree.pdf]

Scale independence

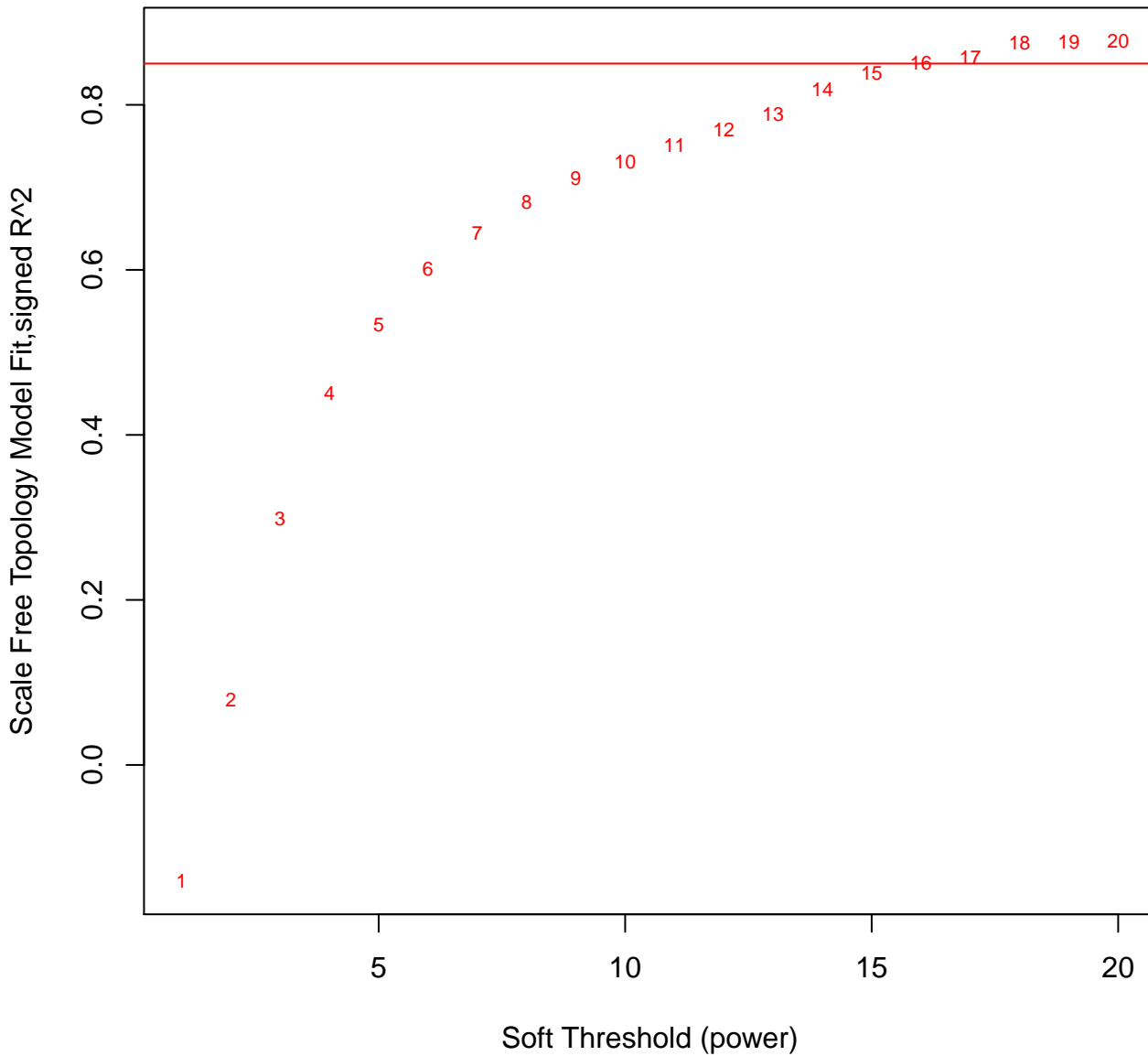

Mean connectivity

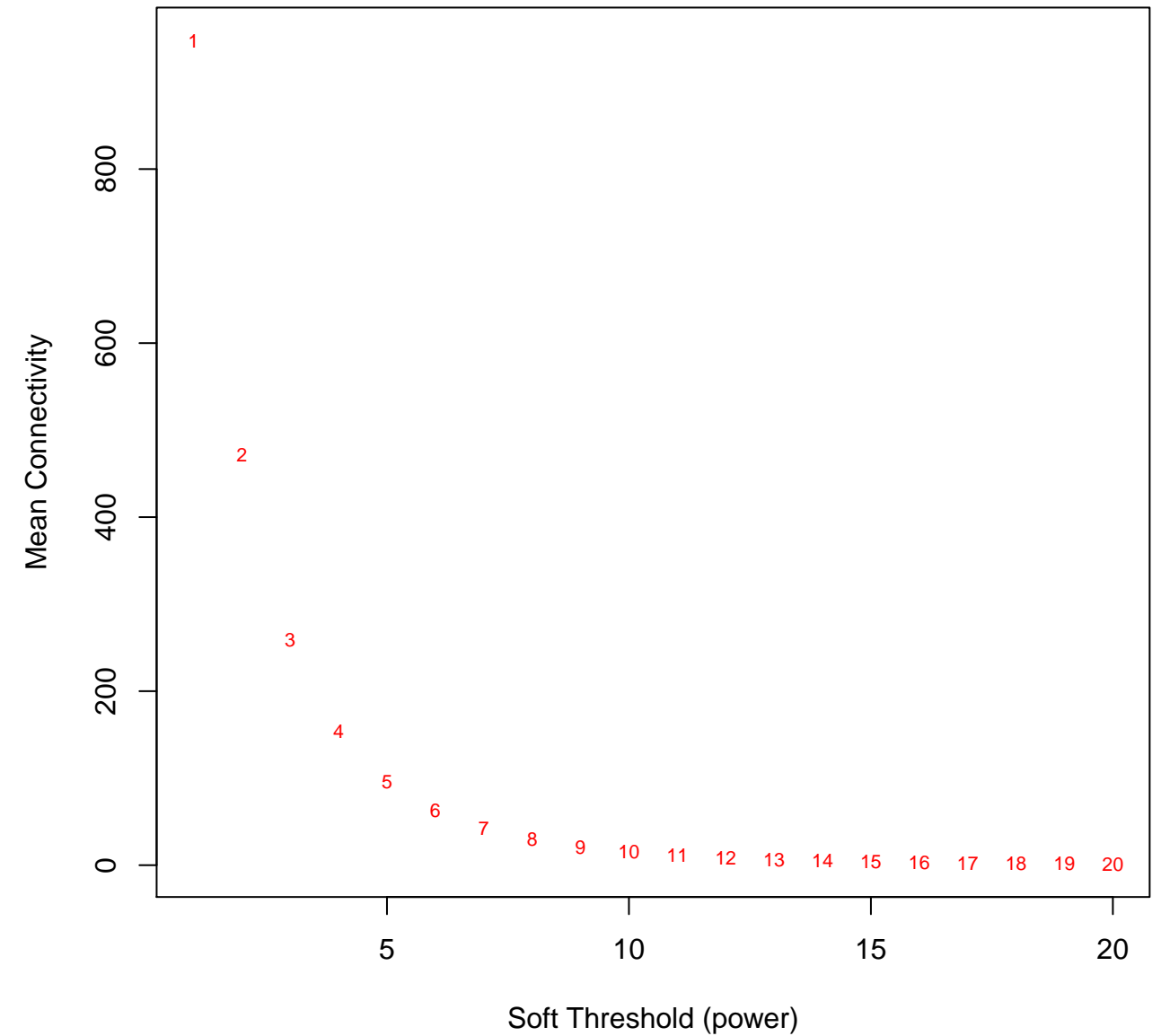

Supplement: Supplementary file 7 [file DataSheet6.ZIP › Figure 5-6--raw data/WGCNA/figure/softThresholding.pdf]

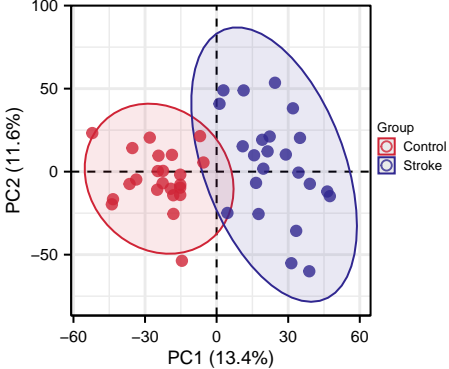

Supplement: Supplementary file 7 [file DataSheet6.ZIP › Figure 5-6--raw data/WGCNA/geo/Affy_mRNA_lncRNA/GSE58294_QC_Nor/PCA_Nor.pdf]

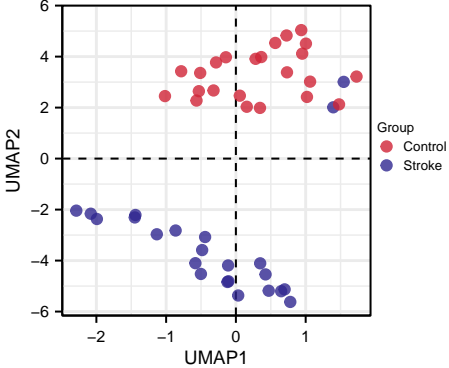

Supplement: Supplementary file 7 [file DataSheet6.ZIP › Figure 5-6--raw data/WGCNA/geo/Affy_mRNA_lncRNA/GSE58294_QC_Nor/UMAP_Nor.pdf]

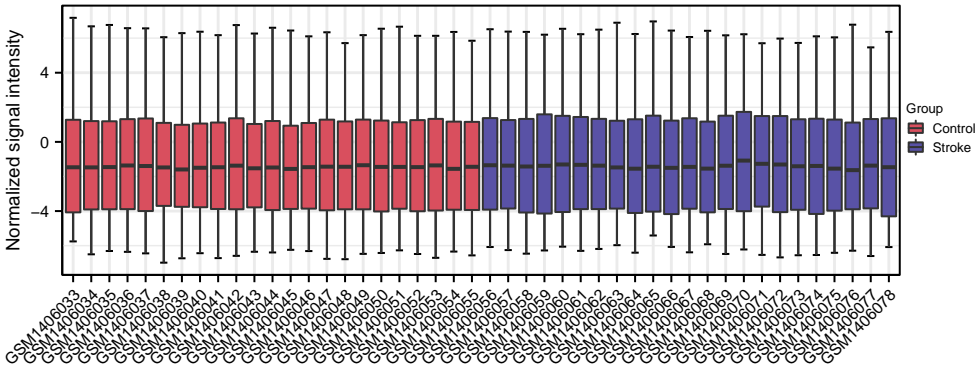

Supplement: Supplementary file 7 [file DataSheet6.ZIP › Figure 5-6--raw data/WGCNA/geo/Affy_mRNA_lncRNA/GSE58294_QC_Nor/boxplot_Nor.pdf]
