# Supplementary material for: Comprehensive Analysis of Hub Genes Associated With Competing Endogenous RNA Networks in Stroke Using Bioinformatics Analysis
Source: Front Genet. 2022 Jan 12;12:779923. doi: 10.3389/fgene.2021.779923 (PMC8790239; doi:10.3389/fgene.2021.779923)
Supplement: Supplementary file 4 [file Table1.DOCX]

| From Node | To Node |
| --- | --- |
| WDR1 | DCTN4 |
| WDR1 | SH3GLB1 |
| WDR1 | ERLIN1 |
| WDR1 | SDF2 |
| WDR1 | ABHD17A |
| WDR1 | MAP3K1 |
| WDR1 | FBXO30 |
| WDR1 | AGFG1 |
| WDR1 | KPNA2 |
| WDR1 | UBE2A |
| WDR1 | GAB1 |
| WDR1 | FAM102A |
| WDR1 | MAPK13 |
| WDR1 | MSL3 |
| WDR1 | ATF6 |
| WDR1 | ETF1 |
| WDR1 | PPFIA1 |
| WDR1 | PRRG4 |
| WDR1 | JAK1 |
| WDR1 | NSDHL |
| WDR1 | SF3A1 |
| WDR1 | BMP2K |
| WDR1 | SAP30 |
| WDR1 | SLK |
| WDR1 | FAM120AOS |
| WDR1 | GIT2 |
| WDR1 | CUL4B |
| WDR1 | VCPIP1 |
| WDR1 | CRIPT |
| WDR1 | PIK3C3 |
| WDR1 | TNFRSF25 |
| WDR1 | ID3 |
| WDR1 | ST6GALNAC3 |
| WDR1 | DHX40 |
| WDR1 | KDM3B |
| WDR1 | SLC12A6 |
| WDR1 | TANC2 |
| WDR1 | IDH1 |
| WDR1 | PAPOLA |
| WDR1 | ATXN2L |
| WDR1 | FEZ2 |
| WDR1 | EAPP |
| WDR1 | HOOK3 |
| WDR1 | CD163 |
| WDR1 | SOAT1 |
| WDR1 | MED31 |
| WDR1 | SENP1 |
| WDR1 | NSMAF |
| WDR1 | PPP5C |
| WDR1 | ATN1 |
| WDR1 | CREBRF |
| WDR1 | VPS41 |
| WDR1 | PSMD12 |
| WDR1 | SHC4 |
| WDR1 | KIAA2013 |
| WDR1 | SAP30L |
| WDR1 | NAGA |
| WDR1 | CLEC5A |
| WDR1 | TSHZ3 |
| WDR1 | ITFG2 |
| WDR1 | STAM2 |
| WDR1 | SPRED2 |
| WDR1 | PSMC3 |
| WDR1 | SCARB2 |
| WDR1 | IPCEF1 |
| WDR1 | TMEM260 |
| WDR1 | TMED8 |
| WDR1 | LPCAT2 |
| WDR1 | WSB1 |
| WDR1 | SCCPDH |
| WDR1 | C7orf73 |
| WDR1 | BAZ2B |
| WDR1 | NDST3 |
| WDR1 | GORASP2 |
| WDR1 | EIF2AK2 |
| WDR1 | TBCC |
| WDR1 | GNAI3 |
| WDR1 | MPPED2 |
| WDR1 | NEURL1 |
| WDR1 | SFN |
| WDR1 | INTS6 |
| WDR1 | TFG |
| WDR1 | MEF2A |
| WDR1 | ASCC1 |
| WDR1 | PRMT1 |
| WDR1 | GOLPH3L |
| WDR1 | CLIC3 |
| WDR1 | UBE2E3 |
| WDR1 | MSX1 |
| WDR1 | AREL1 |
| WDR1 | S1PR3 |
| WDR1 | SCYL2 |
| WDR1 | SIX3 |
| WDR1 | SLX4IP |
| WDR1 | RNF165 |
| WDR1 | NBN |
| WDR1 | PLD4 |
| WDR1 | OGFOD3 |
| WDR1 | CYBB |
| WDR1 | C8orf88 |
| WDR1 | AK2 |
| WDR1 | GNB4 |
| WDR1 | SELT |
| WDR1 | DCUN1D1 |
| WDR1 | ASPH |
| WDR1 | SETD7 |
| WDR1 | ARL15 |
| WDR1 | MMP19 |
| WDR1 | CEP164 |
| WDR1 | PLIN2 |
| WDR1 | GPER1 |
| WDR1 | BROX |
| WDR1 | C5orf45 |
| WDR1 | SUMF1 |
| WDR1 | FBN2 |
| WDR1 | ARRDC4 |
| WDR1 | DNAJA2 |
| WDR1 | DMRT2 |
| WDR1 | ZNF287 |
| WDR1 | CYFIP1 |
| WDR1 | TPM2 |
| WDR1 | BCAT1 |
| WDR1 | FAM199X |
| WDR1 | ADRBK2 |
| WDR1 | LRTOMT |
| WDR1 | GOLPH3 |
| WDR1 | ARF3 |
| WDR1 | TTC28 |
| WDR1 | SCPEP1 |
| WDR1 | CMTM4 |
| WDR1 | ZNF483 |
| WDR1 | ALKBH2 |
| WDR1 | F8 |
| WDR1 | FLVCR2 |
| WDR1 | MS4A4A |
| WDR1 | CHMP7 |
| WDR1 | ACP6 |
| WDR1 | BRCA1 |
| WDR1 | TRAPPC6A |
| WDR1 | EPB41L3 |
| WDR1 | NPEPL1 |
| WDR1 | PAPSS1 |
| WDR1 | DDIT3 |
| WDR1 | NOS3 |
| WDR1 | LDLRAD3 |
| WDR1 | FAM110B |
| WDR1 | RRM2B |
| WDR1 | PHTF1 |
| WDR1 | CLIC4 |
| WDR1 | SLC1A3 |
| WDR1 | DSE |
| WDR1 | SGMS2 |
| WDR1 | PRMT5 |
| WDR1 | MTL5 |
| WDR1 | KNOP1 |
| WDR1 | VAV3 |
| WDR1 | ERI3 |
| WDR1 | CREB1 |
| WDR1 | GAPT |
| WDR1 | ATP10D |
| WDR1 | CC2D2B |
| WDR1 | ENOX1 |
| WDR1 | RLTPR |
| WDR1 | MPZL2 |
| WDR1 | RNASE4 |
| WDR1 | PHACTR1 |
| WDR1 | FLT3 |
| WDR1 | NKAP |
| WDR1 | CD27 |
| WDR1 | DLGAP1-AS2 |
| WDR1 | RP1-193H18.2 |
| WDR1 | LINC00550 |
| WDR1 | LINC00282 |
| WDR1 | DKFZP434L187 |
| WDR1 | RP1-30M3.5 |
| WDR1 | RP11-111K18.2 |
| WDR1 | RP11-2E11.9 |
| WDR1 | RP11-749H17.2 |
| WDR1 | LINC00032 |
| WDR1 | RP11-399O19.9 |
| WDR1 | KB-431C1.4 |
| WDR1 | RP11-1024P17.1 |
| WDR1 | SNRK-AS1 |
| WDR1 | RP11-476D10.1 |
| DCTN4 | SH3GLB1 |
| DCTN4 | ERLIN1 |
| DCTN4 | SDF2 |
| DCTN4 | ABHD17A |
| DCTN4 | MAP3K1 |
| DCTN4 | FBXO30 |
| DCTN4 | AGFG1 |
| DCTN4 | KPNA2 |
| DCTN4 | UBE2A |
| DCTN4 | GAB1 |
| DCTN4 | FAM102A |
| DCTN4 | MAPK13 |
| DCTN4 | MSL3 |
| DCTN4 | ATF6 |
| DCTN4 | ETF1 |
| DCTN4 | PPFIA1 |
| DCTN4 | PRRG4 |
| DCTN4 | JAK1 |
| DCTN4 | NSDHL |
| DCTN4 | SF3A1 |
| DCTN4 | BMP2K |
| DCTN4 | SAP30 |
| DCTN4 | SLK |
| DCTN4 | FAM120AOS |
| DCTN4 | GIT2 |
| DCTN4 | CUL4B |
| DCTN4 | VCPIP1 |
| DCTN4 | CRIPT |
| DCTN4 | PIK3C3 |
| DCTN4 | TNFRSF25 |
| DCTN4 | ID3 |
| DCTN4 | ST6GALNAC3 |
| DCTN4 | DHX40 |
| DCTN4 | KDM3B |
| DCTN4 | SLC12A6 |
| DCTN4 | TANC2 |
| DCTN4 | IDH1 |
| DCTN4 | PAPOLA |
| DCTN4 | ATXN2L |
| DCTN4 | FEZ2 |
| DCTN4 | EAPP |
| DCTN4 | HOOK3 |
| DCTN4 | CD163 |
| DCTN4 | SOAT1 |
| DCTN4 | MED31 |
| DCTN4 | SENP1 |
| DCTN4 | NSMAF |
| DCTN4 | PPP5C |
| DCTN4 | ATN1 |
| DCTN4 | CREBRF |
| DCTN4 | VPS41 |
| DCTN4 | PSMD12 |
| DCTN4 | KIAA2013 |
| DCTN4 | SAP30L |
| DCTN4 | NAGA |
| DCTN4 | CLEC5A |
| DCTN4 | TSHZ3 |
| DCTN4 | ITFG2 |
| DCTN4 | STAM2 |
| DCTN4 | SPRED2 |
| DCTN4 | PSMC3 |
| DCTN4 | SCARB2 |
| DCTN4 | IPCEF1 |
| DCTN4 | TMEM260 |
| DCTN4 | TMED8 |
| DCTN4 | LPCAT2 |
| DCTN4 | WSB1 |
| DCTN4 | SCCPDH |
| DCTN4 | C7orf73 |
| DCTN4 | BAZ2B |
| DCTN4 | NDST3 |
| DCTN4 | GORASP2 |
| DCTN4 | EIF2AK2 |
| DCTN4 | TBCC |
| DCTN4 | GNAI3 |
| DCTN4 | MPPED2 |
| DCTN4 | NEURL1 |
| DCTN4 | SFN |
| DCTN4 | INTS6 |
| DCTN4 | TFG |
| DCTN4 | MEF2A |
| DCTN4 | ASCC1 |
| DCTN4 | PRMT1 |
| DCTN4 | GOLPH3L |
| DCTN4 | CLIC3 |
| DCTN4 | UBE2E3 |
| DCTN4 | MSX1 |
| DCTN4 | AREL1 |
| DCTN4 | S1PR3 |
| DCTN4 | SCYL2 |
| DCTN4 | SIX3 |
| DCTN4 | SLX4IP |
| DCTN4 | RNF165 |
| DCTN4 | NBN |
| DCTN4 | PLD4 |
| DCTN4 | OGFOD3 |
| DCTN4 | CYBB |
| DCTN4 | C8orf88 |
| DCTN4 | AK2 |
| DCTN4 | BIRC2 |
| DCTN4 | GNB4 |
| DCTN4 | SELT |
| DCTN4 | DCUN1D1 |
| DCTN4 | ASPH |
| DCTN4 | SETD7 |
| DCTN4 | ARL15 |
| DCTN4 | MMP19 |
| DCTN4 | CEP164 |
| DCTN4 | PLIN2 |
| DCTN4 | GPER1 |
| DCTN4 | BROX |
| DCTN4 | C5orf45 |
| DCTN4 | SUMF1 |
| DCTN4 | FBN2 |
| DCTN4 | ARRDC4 |
| DCTN4 | DNAJA2 |
| DCTN4 | DMRT2 |
| DCTN4 | CYFIP1 |
| DCTN4 | TPM2 |
| DCTN4 | BCAT1 |
| DCTN4 | FAM199X |
| DCTN4 | ADRBK2 |
| DCTN4 | PNPLA3 |
| DCTN4 | LRTOMT |
| DCTN4 | GOLPH3 |
| DCTN4 | ARF3 |
| DCTN4 | TTC28 |
| DCTN4 | SCPEP1 |
| DCTN4 | CMTM4 |
| DCTN4 | ZNF483 |
| DCTN4 | ALKBH2 |
| DCTN4 | F8 |
| DCTN4 | FLVCR2 |
| DCTN4 | PIEZO1 |
| DCTN4 | MS4A4A |
| DCTN4 | CHMP7 |
| DCTN4 | ACP6 |
| DCTN4 | BRCA1 |
| DCTN4 | TRAPPC6A |
| DCTN4 | EPB41L3 |
| DCTN4 | NPEPL1 |
| DCTN4 | PAPSS1 |
| DCTN4 | DDIT3 |
| DCTN4 | NOS3 |
| DCTN4 | MLC1 |
| DCTN4 | LDLRAD3 |
| DCTN4 | FAM110B |
| DCTN4 | RRM2B |
| DCTN4 | PHTF1 |
| DCTN4 | CLIC4 |
| DCTN4 | SLC1A3 |
| DCTN4 | TLR10 |
| DCTN4 | DSE |
| DCTN4 | SGMS2 |
| DCTN4 | PRMT5 |
| DCTN4 | MTL5 |
| DCTN4 | KNOP1 |
| DCTN4 | VAV3 |
| DCTN4 | ERI3 |
| DCTN4 | CREB1 |
| DCTN4 | GAPT |
| DCTN4 | ATP10D |
| DCTN4 | CC2D2B |
| DCTN4 | ENOX1 |
| DCTN4 | TACR1 |
| DCTN4 | MATK |
| DCTN4 | MPZL2 |
| DCTN4 | SALL3 |
| DCTN4 | RNASE4 |
| DCTN4 | PHACTR1 |
| DCTN4 | NETO2 |
| DCTN4 | FLT3 |
| DCTN4 | NKAP |
| DCTN4 | MOSPD1 |
| DCTN4 | CD27 |
| DCTN4 | PAPSS2 |
| DCTN4 | IFIT5 |
| DCTN4 | DLGAP1-AS2 |
| DCTN4 | RP1-193H18.2 |
| DCTN4 | LINC00282 |
| DCTN4 | DKFZP434L187 |
| DCTN4 | RP1-30M3.5 |
| DCTN4 | RP11-111K18.2 |
| DCTN4 | RP11-2E11.9 |
| DCTN4 | RP11-749H17.2 |
| DCTN4 | RP11-399O19.9 |
| DCTN4 | KB-431C1.4 |
| DCTN4 | RP11-1024P17.1 |
| DCTN4 | SNRK-AS1 |
| DCTN4 | AC005785.2 |
| DCTN4 | RP11-476D10.1 |
| SH3GLB1 | ERLIN1 |
| SH3GLB1 | SDF2 |
| SH3GLB1 | ABHD17A |
| SH3GLB1 | MAP3K1 |
| SH3GLB1 | FBXO30 |
| SH3GLB1 | AGFG1 |
| SH3GLB1 | KPNA2 |
| SH3GLB1 | UBE2A |
| SH3GLB1 | GAB1 |
| SH3GLB1 | FAM102A |
| SH3GLB1 | MAPK13 |
| SH3GLB1 | MSL3 |
| SH3GLB1 | ATF6 |
| SH3GLB1 | ETF1 |
| SH3GLB1 | PPFIA1 |
| SH3GLB1 | PRRG4 |
| SH3GLB1 | JAK1 |
| SH3GLB1 | NSDHL |
| SH3GLB1 | SF3A1 |
| SH3GLB1 | BMP2K |
| SH3GLB1 | SAP30 |
| SH3GLB1 | SLK |
| SH3GLB1 | FAM120AOS |
| SH3GLB1 | GIT2 |
| SH3GLB1 | CUL4B |
| SH3GLB1 | VCPIP1 |
| SH3GLB1 | CRIPT |
| SH3GLB1 | PIK3C3 |
| SH3GLB1 | TNFRSF25 |
| SH3GLB1 | ID3 |
| SH3GLB1 | ST6GALNAC3 |
| SH3GLB1 | DHX40 |
| SH3GLB1 | KDM3B |
| SH3GLB1 | SLC12A6 |
| SH3GLB1 | TANC2 |
| SH3GLB1 | IDH1 |
| SH3GLB1 | PAPOLA |
| SH3GLB1 | ATXN2L |
| SH3GLB1 | FEZ2 |
| SH3GLB1 | EAPP |
| SH3GLB1 | HOOK3 |
| SH3GLB1 | CD163 |
| SH3GLB1 | SOAT1 |
| SH3GLB1 | MED31 |
| SH3GLB1 | SENP1 |
| SH3GLB1 | NSMAF |
| SH3GLB1 | PPP5C |
| SH3GLB1 | ATN1 |
| SH3GLB1 | CREBRF |
| SH3GLB1 | VPS41 |
| SH3GLB1 | PSMD12 |
| SH3GLB1 | SHC4 |
| SH3GLB1 | KIAA2013 |
| SH3GLB1 | SAP30L |
| SH3GLB1 | NAGA |
| SH3GLB1 | CLEC5A |
| SH3GLB1 | TSHZ3 |
| SH3GLB1 | ITFG2 |
| SH3GLB1 | STAM2 |
| SH3GLB1 | SPRED2 |
| SH3GLB1 | PSMC3 |
| SH3GLB1 | SCARB2 |
| SH3GLB1 | IPCEF1 |
| SH3GLB1 | TMEM260 |
| SH3GLB1 | TMED8 |
| SH3GLB1 | LPCAT2 |
| SH3GLB1 | WSB1 |
| SH3GLB1 | SCCPDH |
| SH3GLB1 | C7orf73 |
| SH3GLB1 | BAZ2B |
| SH3GLB1 | NDST3 |
| SH3GLB1 | GORASP2 |
| SH3GLB1 | EIF2AK2 |
| SH3GLB1 | TBCC |
| SH3GLB1 | GNAI3 |
| SH3GLB1 | MPPED2 |
| SH3GLB1 | NEURL1 |
| SH3GLB1 | SFN |
| SH3GLB1 | INTS6 |
| SH3GLB1 | TFG |
| SH3GLB1 | MEF2A |
| SH3GLB1 | ASCC1 |
| SH3GLB1 | PRMT1 |
| SH3GLB1 | GOLPH3L |
| SH3GLB1 | CLIC3 |
| SH3GLB1 | UBE2E3 |
| SH3GLB1 | MSX1 |
| SH3GLB1 | AREL1 |
| SH3GLB1 | S1PR3 |
| SH3GLB1 | SCYL2 |
| SH3GLB1 | SIX3 |
| SH3GLB1 | SLX4IP |
| SH3GLB1 | RNF165 |
| SH3GLB1 | NBN |
| SH3GLB1 | PLD4 |
| SH3GLB1 | OGFOD3 |
| SH3GLB1 | CYBB |
| SH3GLB1 | C8orf88 |
| SH3GLB1 | AK2 |
| SH3GLB1 | BIRC2 |
| SH3GLB1 | GNB4 |
| SH3GLB1 | SELT |
| SH3GLB1 | DCUN1D1 |
| SH3GLB1 | ASPH |
| SH3GLB1 | SETD7 |
| SH3GLB1 | ARL15 |
| SH3GLB1 | MMP19 |
| SH3GLB1 | CEP164 |
| SH3GLB1 | PLIN2 |
| SH3GLB1 | GPER1 |
| SH3GLB1 | BROX |
| SH3GLB1 | SUMF1 |
| SH3GLB1 | FBN2 |
| SH3GLB1 | ARRDC4 |
| SH3GLB1 | DNAJA2 |
| SH3GLB1 | DMRT2 |
| SH3GLB1 | CYFIP1 |
| SH3GLB1 | TPM2 |
| SH3GLB1 | BCAT1 |
| SH3GLB1 | FAM199X |
| SH3GLB1 | ADRBK2 |
| SH3GLB1 | LRTOMT |
| SH3GLB1 | GOLPH3 |
| SH3GLB1 | ARF3 |
| SH3GLB1 | TTC28 |
| SH3GLB1 | SCPEP1 |
| SH3GLB1 | CMTM4 |
| SH3GLB1 | ZNF483 |
| SH3GLB1 | ALKBH2 |
| SH3GLB1 | F8 |
| SH3GLB1 | FLVCR2 |
| SH3GLB1 | MS4A4A |
| SH3GLB1 | CHMP7 |
| SH3GLB1 | ACP6 |
| SH3GLB1 | BRCA1 |
| SH3GLB1 | TRAPPC6A |
| SH3GLB1 | NPEPL1 |
| SH3GLB1 | PAPSS1 |
| SH3GLB1 | DDIT3 |
| SH3GLB1 | NOS3 |
| SH3GLB1 | FAM110B |
| SH3GLB1 | RRM2B |
| SH3GLB1 | PHTF1 |
| SH3GLB1 | CLIC4 |
| SH3GLB1 | SLC1A3 |
| SH3GLB1 | TLR10 |
| SH3GLB1 | DSE |
| SH3GLB1 | SGMS2 |
| SH3GLB1 | PRMT5 |
| SH3GLB1 | MTL5 |
| SH3GLB1 | VAV3 |
| SH3GLB1 | ERI3 |
| SH3GLB1 | CREB1 |
| SH3GLB1 | GAPT |
| SH3GLB1 | ATP10D |
| SH3GLB1 | CC2D2B |
| SH3GLB1 | ENOX1 |
| SH3GLB1 | MATK |
| SH3GLB1 | MPZL2 |
| SH3GLB1 | RNASE4 |
| SH3GLB1 | PHACTR1 |
| SH3GLB1 | FLT3 |
| SH3GLB1 | NKAP |
| SH3GLB1 | SEMG1 |
| SH3GLB1 | IFIT5 |
| SH3GLB1 | DLGAP1-AS2 |
| SH3GLB1 | RP1-193H18.2 |
| SH3GLB1 | LINC00282 |
| SH3GLB1 | DKFZP434L187 |
| SH3GLB1 | RP1-30M3.5 |
| SH3GLB1 | RP11-111K18.2 |
| SH3GLB1 | RP11-2E11.9 |
| SH3GLB1 | RP11-399O19.9 |
| SH3GLB1 | KB-431C1.4 |
| SH3GLB1 | RP11-1024P17.1 |
| SH3GLB1 | SNRK-AS1 |
| SH3GLB1 | AC005785.2 |
| SH3GLB1 | RP11-476D10.1 |
| ERLIN1 | SDF2 |
| ERLIN1 | ABHD17A |
| ERLIN1 | MAP3K1 |
| ERLIN1 | FBXO30 |
| ERLIN1 | AGFG1 |
| ERLIN1 | KPNA2 |
| ERLIN1 | UBE2A |
| ERLIN1 | GAB1 |
| ERLIN1 | FAM102A |
| ERLIN1 | MAPK13 |
| ERLIN1 | MSL3 |
| ERLIN1 | ATF6 |
| ERLIN1 | ETF1 |
| ERLIN1 | PPFIA1 |
| ERLIN1 | PRRG4 |
| ERLIN1 | JAK1 |
| ERLIN1 | NSDHL |
| ERLIN1 | SF3A1 |
| ERLIN1 | BMP2K |
| ERLIN1 | SAP30 |
| ERLIN1 | SLK |
| ERLIN1 | FAM120AOS |
| ERLIN1 | GIT2 |
| ERLIN1 | CUL4B |
| ERLIN1 | VCPIP1 |
| ERLIN1 | CRIPT |
| ERLIN1 | PIK3C3 |
| ERLIN1 | TNFRSF25 |
| ERLIN1 | ID3 |
| ERLIN1 | ST6GALNAC3 |
| ERLIN1 | DHX40 |
| ERLIN1 | KDM3B |
| ERLIN1 | SLC12A6 |
| ERLIN1 | TANC2 |
| ERLIN1 | IDH1 |
| ERLIN1 | PAPOLA |
| ERLIN1 | ATXN2L |
| ERLIN1 | FEZ2 |
| ERLIN1 | EAPP |
| ERLIN1 | HOOK3 |
| ERLIN1 | CD163 |
| ERLIN1 | SOAT1 |
| ERLIN1 | MED31 |
| ERLIN1 | SENP1 |
| ERLIN1 | NSMAF |
| ERLIN1 | PPP5C |
| ERLIN1 | ATN1 |
| ERLIN1 | CREBRF |
| ERLIN1 | VPS41 |
| ERLIN1 | PSMD12 |
| ERLIN1 | SHC4 |
| ERLIN1 | KIAA2013 |
| ERLIN1 | SAP30L |
| ERLIN1 | NAGA |
| ERLIN1 | CLEC5A |
| ERLIN1 | TSHZ3 |
| ERLIN1 | ITFG2 |
| ERLIN1 | STAM2 |
| ERLIN1 | SPRED2 |
| ERLIN1 | PSMC3 |
| ERLIN1 | SCARB2 |
| ERLIN1 | IPCEF1 |
| ERLIN1 | TMEM260 |
| ERLIN1 | TMED8 |
| ERLIN1 | LPCAT2 |
| ERLIN1 | WSB1 |
| ERLIN1 | SCCPDH |
| ERLIN1 | BAZ2B |
| ERLIN1 | NDST3 |
| ERLIN1 | GORASP2 |
| ERLIN1 | EIF2AK2 |
| ERLIN1 | TBCC |
| ERLIN1 | GNAI3 |
| ERLIN1 | MPPED2 |
| ERLIN1 | NEURL1 |
| ERLIN1 | SFN |
| ERLIN1 | INTS6 |
| ERLIN1 | TFG |
| ERLIN1 | MEF2A |
| ERLIN1 | ASCC1 |
| ERLIN1 | PRMT1 |
| ERLIN1 | GOLPH3L |
| ERLIN1 | CLIC3 |
| ERLIN1 | UBE2E3 |
| ERLIN1 | MSX1 |
| ERLIN1 | AREL1 |
| ERLIN1 | S1PR3 |
| ERLIN1 | SCYL2 |
| ERLIN1 | SIX3 |
| ERLIN1 | SLX4IP |
| ERLIN1 | RNF165 |
| ERLIN1 | NBN |
| ERLIN1 | PLD4 |
| ERLIN1 | OGFOD3 |
| ERLIN1 | CYBB |
| ERLIN1 | C8orf88 |
| ERLIN1 | AK2 |
| ERLIN1 | BIRC2 |
| ERLIN1 | GNB4 |
| ERLIN1 | SELT |
| ERLIN1 | DCUN1D1 |
| ERLIN1 | ASPH |
| ERLIN1 | SETD7 |
| ERLIN1 | ARL15 |
| ERLIN1 | MMP19 |
| ERLIN1 | CEP164 |
| ERLIN1 | PLIN2 |
| ERLIN1 | GPER1 |
| ERLIN1 | BROX |
| ERLIN1 | SUMF1 |
| ERLIN1 | FBN2 |
| ERLIN1 | ARRDC4 |
| ERLIN1 | DNAJA2 |
| ERLIN1 | DMRT2 |
| ERLIN1 | CYFIP1 |
| ERLIN1 | TPM2 |
| ERLIN1 | BCAT1 |
| ERLIN1 | FAM199X |
| ERLIN1 | ADRBK2 |
| ERLIN1 | LRTOMT |
| ERLIN1 | GOLPH3 |
| ERLIN1 | ARF3 |
| ERLIN1 | TTC28 |
| ERLIN1 | SCPEP1 |
| ERLIN1 | CMTM4 |
| ERLIN1 | ZNF483 |
| ERLIN1 | ALKBH2 |
| ERLIN1 | F8 |
| ERLIN1 | FLVCR2 |
| ERLIN1 | MS4A4A |
| ERLIN1 | CHMP7 |
| ERLIN1 | ACP6 |
| ERLIN1 | BRCA1 |
| ERLIN1 | TRAPPC6A |
| ERLIN1 | NPEPL1 |
| ERLIN1 | PAPSS1 |
| ERLIN1 | DDIT3 |
| ERLIN1 | NOS3 |
| ERLIN1 | FAM110B |
| ERLIN1 | RRM2B |
| ERLIN1 | PHTF1 |
| ERLIN1 | CLIC4 |
| ERLIN1 | SLC1A3 |
| ERLIN1 | TLR10 |
| ERLIN1 | DSE |
| ERLIN1 | SGMS2 |
| ERLIN1 | PRMT5 |
| ERLIN1 | MTL5 |
| ERLIN1 | VAV3 |
| ERLIN1 | CREB1 |
| ERLIN1 | GAPT |
| ERLIN1 | ATP10D |
| ERLIN1 | CC2D2B |
| ERLIN1 | ENOX1 |
| ERLIN1 | TACR1 |
| ERLIN1 | MPZL2 |
| ERLIN1 | RNASE4 |
| ERLIN1 | PHACTR1 |
| ERLIN1 | FLT3 |
| ERLIN1 | NKAP |
| ERLIN1 | DLGAP1-AS2 |
| ERLIN1 | RP1-193H18.2 |
| ERLIN1 | LINC00282 |
| ERLIN1 | DKFZP434L187 |
| ERLIN1 | RP1-30M3.5 |
| ERLIN1 | RP11-111K18.2 |
| ERLIN1 | RP11-2E11.9 |
| ERLIN1 | RP11-399O19.9 |
| ERLIN1 | KB-431C1.4 |
| ERLIN1 | RP11-1024P17.1 |
| ERLIN1 | SNRK-AS1 |
| ERLIN1 | AC005785.2 |
| ERLIN1 | RP11-476D10.1 |
| SDF2 | ABHD17A |
| SDF2 | MAP3K1 |
| SDF2 | FBXO30 |
| SDF2 | AGFG1 |
| SDF2 | KPNA2 |
| SDF2 | UBE2A |
| SDF2 | GAB1 |
| SDF2 | FAM102A |
| SDF2 | MAPK13 |
| SDF2 | MSL3 |
| SDF2 | ATF6 |
| SDF2 | ETF1 |
| SDF2 | PPFIA1 |
| SDF2 | PRRG4 |
| SDF2 | JAK1 |
| SDF2 | NSDHL |
| SDF2 | SF3A1 |
| SDF2 | BMP2K |
| SDF2 | SAP30 |
| SDF2 | SLK |
| SDF2 | FAM120AOS |
| SDF2 | GIT2 |
| SDF2 | CUL4B |
| SDF2 | VCPIP1 |
| SDF2 | CRIPT |
| SDF2 | PIK3C3 |
| SDF2 | TNFRSF25 |
| SDF2 | ID3 |
| SDF2 | ST6GALNAC3 |
| SDF2 | DHX40 |
| SDF2 | KDM3B |
| SDF2 | SLC12A6 |
| SDF2 | TANC2 |
| SDF2 | IDH1 |
| SDF2 | PAPOLA |
| SDF2 | ATXN2L |
| SDF2 | FEZ2 |
| SDF2 | EAPP |
| SDF2 | HOOK3 |
| SDF2 | CD163 |
| SDF2 | SOAT1 |
| SDF2 | MED31 |
| SDF2 | SENP1 |
| SDF2 | NSMAF |
| SDF2 | PPP5C |
| SDF2 | ATN1 |
| SDF2 | CREBRF |
| SDF2 | VPS41 |
| SDF2 | PSMD12 |
| SDF2 | SHC4 |
| SDF2 | KIAA2013 |
| SDF2 | SAP30L |
| SDF2 | NAGA |
| SDF2 | CLEC5A |
| SDF2 | TSHZ3 |
| SDF2 | ITFG2 |
| SDF2 | STAM2 |
| SDF2 | SPRED2 |
| SDF2 | PSMC3 |
| SDF2 | SCARB2 |
| SDF2 | IPCEF1 |
| SDF2 | TMEM260 |
| SDF2 | TMED8 |
| SDF2 | LPCAT2 |
| SDF2 | WSB1 |
| SDF2 | SCCPDH |
| SDF2 | C7orf73 |
| SDF2 | BAZ2B |
| SDF2 | NDST3 |
| SDF2 | GORASP2 |
| SDF2 | EIF2AK2 |
| SDF2 | TBCC |
| SDF2 | GNAI3 |
| SDF2 | MPPED2 |
| SDF2 | NEURL1 |
| SDF2 | SFN |
| SDF2 | INTS6 |
| SDF2 | TFG |
| SDF2 | MEF2A |
| SDF2 | ASCC1 |
| SDF2 | PRMT1 |
| SDF2 | GOLPH3L |
| SDF2 | CLIC3 |
| SDF2 | UBE2E3 |
| SDF2 | MSX1 |
| SDF2 | AREL1 |
| SDF2 | S1PR3 |
| SDF2 | SCYL2 |
| SDF2 | SIX3 |
| SDF2 | ZNF521 |
| SDF2 | SLX4IP |
| SDF2 | RNF165 |
| SDF2 | NBN |
| SDF2 | PLD4 |
| SDF2 | OGFOD3 |
| SDF2 | CYBB |
| SDF2 | C8orf88 |
| SDF2 | AK2 |
| SDF2 | BIRC2 |
| SDF2 | GNB4 |
| SDF2 | SELT |
| SDF2 | DCUN1D1 |
| SDF2 | ASPH |
| SDF2 | SETD7 |
| SDF2 | ARL15 |
| SDF2 | MMP19 |
| SDF2 | CEP164 |
| SDF2 | PLIN2 |
| SDF2 | GPER1 |
| SDF2 | BROX |
| SDF2 | SUMF1 |
| SDF2 | FBN2 |
| SDF2 | ARRDC4 |
| SDF2 | DNAJA2 |
| SDF2 | DMRT2 |
| SDF2 | ZNF287 |
| SDF2 | CYFIP1 |
| SDF2 | TPM2 |
| SDF2 | BCAT1 |
| SDF2 | FAM199X |
| SDF2 | ADRBK2 |
| SDF2 | LRTOMT |
| SDF2 | GOLPH3 |
| SDF2 | ARF3 |
| SDF2 | TTC28 |
| SDF2 | SCPEP1 |
| SDF2 | CMTM4 |
| SDF2 | ZNF483 |
| SDF2 | ALKBH2 |
| SDF2 | F8 |
| SDF2 | FLVCR2 |
| SDF2 | MS4A4A |
| SDF2 | CHMP7 |
| SDF2 | ACP6 |
| SDF2 | BRCA1 |
| SDF2 | TRAPPC6A |
| SDF2 | NPEPL1 |
| SDF2 | PAPSS1 |
| SDF2 | PDE1C |
| SDF2 | DDIT3 |
| SDF2 | NOS3 |
| SDF2 | MLC1 |
| SDF2 | FAM110B |
| SDF2 | GOLGA6L2 |
| SDF2 | RRM2B |
| SDF2 | PHTF1 |
| SDF2 | CLIC4 |
| SDF2 | SLC1A3 |
| SDF2 | TLR10 |
| SDF2 | DSE |
| SDF2 | SGMS2 |
| SDF2 | PRMT5 |
| SDF2 | MTL5 |
| SDF2 | KNOP1 |
| SDF2 | VAV3 |
| SDF2 | ERI3 |
| SDF2 | CREB1 |
| SDF2 | GAPT |
| SDF2 | ATP10D |
| SDF2 | CC2D2B |
| SDF2 | ENOX1 |
| SDF2 | TACR1 |
| SDF2 | MPZL2 |
| SDF2 | SALL3 |
| SDF2 | RNASE4 |
| SDF2 | PHACTR1 |
| SDF2 | FLT3 |
| SDF2 | NKAP |
| SDF2 | DLGAP1-AS2 |
| SDF2 | RP1-193H18.2 |
| SDF2 | LINC00282 |
| SDF2 | DKFZP434L187 |
| SDF2 | RP1-30M3.5 |
| SDF2 | RP11-111K18.2 |
| SDF2 | RP11-2E11.9 |
| SDF2 | RP11-399O19.9 |
| SDF2 | KB-431C1.4 |
| SDF2 | RP11-1024P17.1 |
| SDF2 | SNRK-AS1 |
| SDF2 | AC005785.2 |
| SDF2 | RP11-476D10.1 |
| ABHD17A | MAP3K1 |
| ABHD17A | FBXO30 |
| ABHD17A | AGFG1 |
| ABHD17A | KPNA2 |
| ABHD17A | UBE2A |
| ABHD17A | GAB1 |
| ABHD17A | MAPK13 |
| ABHD17A | MSL3 |
| ABHD17A | ATF6 |
| ABHD17A | ETF1 |
| ABHD17A | PPFIA1 |
| ABHD17A | PRRG4 |
| ABHD17A | JAK1 |
| ABHD17A | BMP2K |
| ABHD17A | SAP30 |
| ABHD17A | SLK |
| ABHD17A | FAM120AOS |
| ABHD17A | GIT2 |
| ABHD17A | CUL4B |
| ABHD17A | VCPIP1 |
| ABHD17A | CRIPT |
| ABHD17A | PIK3C3 |
| ABHD17A | DHX40 |
| ABHD17A | KDM3B |
| ABHD17A | TANC2 |
| ABHD17A | IDH1 |
| ABHD17A | PAPOLA |
| ABHD17A | EAPP |
| ABHD17A | HOOK3 |
| ABHD17A | CD163 |
| ABHD17A | SOAT1 |
| ABHD17A | SENP1 |
| ABHD17A | NSMAF |
| ABHD17A | CREBRF |
| ABHD17A | VPS41 |
| ABHD17A | PSMD12 |
| ABHD17A | KIAA2013 |
| ABHD17A | TSHZ3 |
| ABHD17A | STAM2 |
| ABHD17A | PSMC3 |
| ABHD17A | IPCEF1 |
| ABHD17A | TMEM260 |
| ABHD17A | TMED8 |
| ABHD17A | LPCAT2 |
| ABHD17A | BAZ2B |
| ABHD17A | GNAI3 |
| ABHD17A | NEURL1 |
| ABHD17A | INTS6 |
| ABHD17A | MEF2A |
| ABHD17A | PRMT1 |
| ABHD17A | GOLPH3L |
| ABHD17A | CLIC3 |
| ABHD17A | SCYL2 |
| ABHD17A | SLX4IP |
| ABHD17A | NBN |
| ABHD17A | GNB4 |
| ABHD17A | SELT |
| ABHD17A | ASPH |
| ABHD17A | BROX |
| ABHD17A | KLHDC3 |
| ABHD17A | SLC1A3 |
| ABHD17A | DSE |
| ABHD17A | MATK |
| ABHD17A | DLGAP1-AS2 |
| ABHD17A | RP1-193H18.2 |
| ABHD17A | RP1-30M3.5 |
| MAP3K1 | FBXO30 |
| MAP3K1 | AGFG1 |
| MAP3K1 | KPNA2 |
| MAP3K1 | UBE2A |
| MAP3K1 | GAB1 |
| MAP3K1 | FAM102A |
| MAP3K1 | MAPK13 |
| MAP3K1 | MSL3 |
| MAP3K1 | ATF6 |
| MAP3K1 | ETF1 |
| MAP3K1 | PPFIA1 |
| MAP3K1 | PRRG4 |
| MAP3K1 | JAK1 |
| MAP3K1 | NSDHL |
| MAP3K1 | SF3A1 |
| MAP3K1 | BMP2K |
| MAP3K1 | SAP30 |
| MAP3K1 | SLK |
| MAP3K1 | FAM120AOS |
| MAP3K1 | GIT2 |
| MAP3K1 | CUL4B |
| MAP3K1 | VCPIP1 |
| MAP3K1 | CRIPT |
| MAP3K1 | PIK3C3 |
| MAP3K1 | TNFRSF25 |
| MAP3K1 | ID3 |
| MAP3K1 | ST6GALNAC3 |
| MAP3K1 | DHX40 |
| MAP3K1 | KDM3B |
| MAP3K1 | SLC12A6 |
| MAP3K1 | TANC2 |
| MAP3K1 | IDH1 |
| MAP3K1 | PAPOLA |
| MAP3K1 | ATXN2L |
| MAP3K1 | FEZ2 |
| MAP3K1 | EAPP |
| MAP3K1 | HOOK3 |
| MAP3K1 | CD163 |
| MAP3K1 | SOAT1 |
| MAP3K1 | MED31 |
| MAP3K1 | SENP1 |
| MAP3K1 | NSMAF |
| MAP3K1 | PPP5C |
| MAP3K1 | ATN1 |
| MAP3K1 | CREBRF |
| MAP3K1 | VPS41 |
| MAP3K1 | PSMD12 |
| MAP3K1 | KIAA2013 |
| MAP3K1 | SAP30L |
| MAP3K1 | NAGA |
| MAP3K1 | CLEC5A |
| MAP3K1 | TSHZ3 |
| MAP3K1 | ITFG2 |
| MAP3K1 | STAM2 |
| MAP3K1 | SPRED2 |
| MAP3K1 | PSMC3 |
| MAP3K1 | SCARB2 |
| MAP3K1 | IPCEF1 |
| MAP3K1 | TMEM260 |
| MAP3K1 | TMED8 |
| MAP3K1 | LPCAT2 |
| MAP3K1 | WSB1 |
| MAP3K1 | SCCPDH |
| MAP3K1 | C7orf73 |
| MAP3K1 | BAZ2B |
| MAP3K1 | NDST3 |
| MAP3K1 | GORASP2 |
| MAP3K1 | EIF2AK2 |
| MAP3K1 | TBCC |
| MAP3K1 | GNAI3 |
| MAP3K1 | MPPED2 |
| MAP3K1 | NEURL1 |
| MAP3K1 | SFN |
| MAP3K1 | INTS6 |
| MAP3K1 | TFG |
| MAP3K1 | MEF2A |
| MAP3K1 | ASCC1 |
| MAP3K1 | PRMT1 |
| MAP3K1 | GOLPH3L |
| MAP3K1 | CLIC3 |
| MAP3K1 | UBE2E3 |
| MAP3K1 | MSX1 |
| MAP3K1 | AREL1 |
| MAP3K1 | S1PR3 |
| MAP3K1 | SCYL2 |
| MAP3K1 | SIX3 |
| MAP3K1 | SLX4IP |
| MAP3K1 | RNF165 |
| MAP3K1 | NBN |
| MAP3K1 | PLD4 |
| MAP3K1 | CYBB |
| MAP3K1 | C8orf88 |
| MAP3K1 | AK2 |
| MAP3K1 | BIRC2 |
| MAP3K1 | GNB4 |
| MAP3K1 | SELT |
| MAP3K1 | DCUN1D1 |
| MAP3K1 | ASPH |
| MAP3K1 | SETD7 |
| MAP3K1 | ARL15 |
| MAP3K1 | CEP164 |
| MAP3K1 | PLIN2 |
| MAP3K1 | GPER1 |
| MAP3K1 | BROX |
| MAP3K1 | SUMF1 |
| MAP3K1 | FBN2 |
| MAP3K1 | ARRDC4 |
| MAP3K1 | DNAJA2 |
| MAP3K1 | CYFIP1 |
| MAP3K1 | TPM2 |
| MAP3K1 | BCAT1 |
| MAP3K1 | FAM199X |
| MAP3K1 | ADRBK2 |
| MAP3K1 | LRTOMT |
| MAP3K1 | GOLPH3 |
| MAP3K1 | ARF3 |
| MAP3K1 | TTC28 |
| MAP3K1 | SCPEP1 |
| MAP3K1 | CMTM4 |
| MAP3K1 | ALKBH2 |
| MAP3K1 | F8 |
| MAP3K1 | FLVCR2 |
| MAP3K1 | MS4A4A |
| MAP3K1 | BRCA1 |
| MAP3K1 | TRAPPC6A |
| MAP3K1 | NPEPL1 |
| MAP3K1 | PAPSS1 |
| MAP3K1 | DDIT3 |
| MAP3K1 | NOS3 |
| MAP3K1 | RRM2B |
| MAP3K1 | PHTF1 |
| MAP3K1 | CLIC4 |
| MAP3K1 | SLC1A3 |
| MAP3K1 | TLR10 |
| MAP3K1 | DSE |
| MAP3K1 | SGMS2 |
| MAP3K1 | PRMT5 |
| MAP3K1 | MTL5 |
| MAP3K1 | VAV3 |
| MAP3K1 | CREB1 |
| MAP3K1 | GAPT |
| MAP3K1 | ATP10D |
| MAP3K1 | CC2D2B |
| MAP3K1 | PHACTR1 |
| MAP3K1 | FLT3 |
| MAP3K1 | NKAP |
| MAP3K1 | IFIT5 |
| MAP3K1 | DLGAP1-AS2 |
| MAP3K1 | RP1-193H18.2 |
| MAP3K1 | LINC00282 |
| MAP3K1 | DKFZP434L187 |
| MAP3K1 | RP1-30M3.5 |
| MAP3K1 | RP11-111K18.2 |
| MAP3K1 | RP11-2E11.9 |
| MAP3K1 | RP11-399O19.9 |
| MAP3K1 | KB-431C1.4 |
| MAP3K1 | RP11-1024P17.1 |
| MAP3K1 | SNRK-AS1 |
| MAP3K1 | RP11-476D10.1 |
| FBXO30 | AGFG1 |
| FBXO30 | KPNA2 |
| FBXO30 | UBE2A |
| FBXO30 | GAB1 |
| FBXO30 | FAM102A |
| FBXO30 | MAPK13 |
| FBXO30 | MSL3 |
| FBXO30 | ATF6 |
| FBXO30 | ETF1 |
| FBXO30 | PPFIA1 |
| FBXO30 | PRRG4 |
| FBXO30 | JAK1 |
| FBXO30 | NSDHL |
| FBXO30 | SF3A1 |
| FBXO30 | BMP2K |
| FBXO30 | SAP30 |
| FBXO30 | SLK |
| FBXO30 | FAM120AOS |
| FBXO30 | GIT2 |
| FBXO30 | CUL4B |
| FBXO30 | VCPIP1 |
| FBXO30 | CRIPT |
| FBXO30 | PIK3C3 |
| FBXO30 | TNFRSF25 |
| FBXO30 | ID3 |
| FBXO30 | ST6GALNAC3 |
| FBXO30 | DHX40 |
| FBXO30 | KDM3B |
| FBXO30 | SLC12A6 |
| FBXO30 | TANC2 |
| FBXO30 | IDH1 |
| FBXO30 | PAPOLA |
| FBXO30 | ATXN2L |
| FBXO30 | FEZ2 |
| FBXO30 | EAPP |
| FBXO30 | HOOK3 |
| FBXO30 | CD163 |
| FBXO30 | SOAT1 |
| FBXO30 | MED31 |
| FBXO30 | SENP1 |
| FBXO30 | NSMAF |
| FBXO30 | PPP5C |
| FBXO30 | ATN1 |
| FBXO30 | CREBRF |
| FBXO30 | VPS41 |
| FBXO30 | PSMD12 |
| FBXO30 | SHC4 |
| FBXO30 | KIAA2013 |
| FBXO30 | SAP30L |
| FBXO30 | NAGA |
| FBXO30 | CLEC5A |
| FBXO30 | TSHZ3 |
| FBXO30 | ITFG2 |
| FBXO30 | STAM2 |
| FBXO30 | SPRED2 |
| FBXO30 | PSMC3 |
| FBXO30 | SCARB2 |
| FBXO30 | IPCEF1 |
| FBXO30 | TMEM260 |
| FBXO30 | TMED8 |
| FBXO30 | LPCAT2 |
| FBXO30 | WSB1 |
| FBXO30 | SCCPDH |
| FBXO30 | C7orf73 |
| FBXO30 | BAZ2B |
| FBXO30 | NDST3 |
| FBXO30 | GORASP2 |
| FBXO30 | EIF2AK2 |
| FBXO30 | TBCC |
| FBXO30 | GNAI3 |
| FBXO30 | MPPED2 |
| FBXO30 | NEURL1 |
| FBXO30 | SFN |
| FBXO30 | INTS6 |
| FBXO30 | TFG |
| FBXO30 | MEF2A |
| FBXO30 | ASCC1 |
| FBXO30 | PRMT1 |
| FBXO30 | GOLPH3L |
| FBXO30 | CLIC3 |
| FBXO30 | UBE2E3 |
| FBXO30 | MSX1 |
| FBXO30 | AREL1 |
| FBXO30 | S1PR3 |
| FBXO30 | SCYL2 |
| FBXO30 | SIX3 |
| FBXO30 | SLX4IP |
| FBXO30 | RNF165 |
| FBXO30 | NBN |
| FBXO30 | PLD4 |
| FBXO30 | OGFOD3 |
| FBXO30 | CYBB |
| FBXO30 | C8orf88 |
| FBXO30 | AK2 |
| FBXO30 | BIRC2 |
| FBXO30 | GNB4 |
| FBXO30 | SELT |
| FBXO30 | DCUN1D1 |
| FBXO30 | ASPH |
| FBXO30 | SETD7 |
| FBXO30 | ARL15 |
| FBXO30 | CEP164 |
| FBXO30 | PLIN2 |
| FBXO30 | GPER1 |
| FBXO30 | BROX |
| FBXO30 | SUMF1 |
| FBXO30 | FBN2 |
| FBXO30 | ARRDC4 |
| FBXO30 | DNAJA2 |
| FBXO30 | CYFIP1 |
| FBXO30 | TPM2 |
| FBXO30 | BCAT1 |
| FBXO30 | FAM199X |
| FBXO30 | ADRBK2 |
| FBXO30 | LRTOMT |
| FBXO30 | GOLPH3 |
| FBXO30 | ARF3 |
| FBXO30 | TTC28 |
| FBXO30 | SCPEP1 |
| FBXO30 | CMTM4 |
| FBXO30 | F8 |
| FBXO30 | FLVCR2 |
| FBXO30 | MS4A4A |
| FBXO30 | CHMP7 |
| FBXO30 | ACP6 |
| FBXO30 | BRCA1 |
| FBXO30 | TRAPPC6A |
| FBXO30 | NPEPL1 |
| FBXO30 | PAPSS1 |
| FBXO30 | DDIT3 |
| FBXO30 | MLC1 |
| FBXO30 | LDLRAD3 |
| FBXO30 | FAM110B |
| FBXO30 | RRM2B |
| FBXO30 | PHTF1 |
| FBXO30 | CLIC4 |
| FBXO30 | SLC1A3 |
| FBXO30 | TLR10 |
| FBXO30 | DSE |
| FBXO30 | SGMS2 |
| FBXO30 | PRMT5 |
| FBXO30 | MTL5 |
| FBXO30 | VAV3 |
| FBXO30 | CREB1 |
| FBXO30 | GAPT |
| FBXO30 | ATP10D |
| FBXO30 | CC2D2B |
| FBXO30 | ENOX1 |
| FBXO30 | MATK |
| FBXO30 | MPZL2 |
| FBXO30 | SALL3 |
| FBXO30 | RNASE4 |
| FBXO30 | PHACTR1 |
| FBXO30 | FLT3 |
| FBXO30 | NKAP |
| FBXO30 | PAPSS2 |
| FBXO30 | SEMG1 |
| FBXO30 | OAS1 |
| FBXO30 | IFIT5 |
| FBXO30 | DLGAP1-AS2 |
| FBXO30 | RP1-193H18.2 |
| FBXO30 | LINC00282 |
| FBXO30 | DKFZP434L187 |
| FBXO30 | RP1-30M3.5 |
| FBXO30 | RP11-111K18.2 |
| FBXO30 | RP11-2E11.9 |
| FBXO30 | RP11-399O19.9 |
| FBXO30 | KB-431C1.4 |
| FBXO30 | RP11-1024P17.1 |
| FBXO30 | SNRK-AS1 |
| FBXO30 | AC005785.2 |
| FBXO30 | RP11-476D10.1 |
| AGFG1 | KPNA2 |
| AGFG1 | UBE2A |
| AGFG1 | GAB1 |
| AGFG1 | FAM102A |
| AGFG1 | MAPK13 |
| AGFG1 | MSL3 |
| AGFG1 | ATF6 |
| AGFG1 | ETF1 |
| AGFG1 | PPFIA1 |
| AGFG1 | PRRG4 |
| AGFG1 | JAK1 |
| AGFG1 | NSDHL |
| AGFG1 | SF3A1 |
| AGFG1 | BMP2K |
| AGFG1 | SAP30 |
| AGFG1 | SLK |
| AGFG1 | FAM120AOS |
| AGFG1 | GIT2 |
| AGFG1 | CUL4B |
| AGFG1 | VCPIP1 |
| AGFG1 | CRIPT |
| AGFG1 | PIK3C3 |
| AGFG1 | TNFRSF25 |
| AGFG1 | ID3 |
| AGFG1 | ST6GALNAC3 |
| AGFG1 | DHX40 |
| AGFG1 | KDM3B |
| AGFG1 | SLC12A6 |
| AGFG1 | TANC2 |
| AGFG1 | IDH1 |
| AGFG1 | PAPOLA |
| AGFG1 | ATXN2L |
| AGFG1 | FEZ2 |
| AGFG1 | EAPP |
| AGFG1 | HOOK3 |
| AGFG1 | CD163 |
| AGFG1 | SOAT1 |
| AGFG1 | MED31 |
| AGFG1 | SENP1 |
| AGFG1 | NSMAF |
| AGFG1 | PPP5C |
| AGFG1 | ATN1 |
| AGFG1 | CREBRF |
| AGFG1 | VPS41 |
| AGFG1 | PSMD12 |
| AGFG1 | SHC4 |
| AGFG1 | KIAA2013 |
| AGFG1 | SAP30L |
| AGFG1 | NAGA |
| AGFG1 | CLEC5A |
| AGFG1 | TSHZ3 |
| AGFG1 | ITFG2 |
| AGFG1 | STAM2 |
| AGFG1 | SPRED2 |
| AGFG1 | PSMC3 |
| AGFG1 | SCARB2 |
| AGFG1 | IPCEF1 |
| AGFG1 | TMEM260 |
| AGFG1 | TMED8 |
| AGFG1 | LPCAT2 |
| AGFG1 | WSB1 |
| AGFG1 | SCCPDH |
| AGFG1 | C7orf73 |
| AGFG1 | BAZ2B |
| AGFG1 | NDST3 |
| AGFG1 | GORASP2 |
| AGFG1 | EIF2AK2 |
| AGFG1 | TBCC |
| AGFG1 | GNAI3 |
| AGFG1 | MPPED2 |
| AGFG1 | NEURL1 |
| AGFG1 | SFN |
| AGFG1 | INTS6 |
| AGFG1 | TFG |
| AGFG1 | MEF2A |
| AGFG1 | ASCC1 |
| AGFG1 | PRMT1 |
| AGFG1 | GOLPH3L |
| AGFG1 | CLIC3 |
| AGFG1 | UBE2E3 |
| AGFG1 | MSX1 |
| AGFG1 | AREL1 |
| AGFG1 | S1PR3 |
| AGFG1 | SCYL2 |
| AGFG1 | SIX3 |
| AGFG1 | SLX4IP |
| AGFG1 | RNF165 |
| AGFG1 | NBN |
| AGFG1 | PLD4 |
| AGFG1 | OGFOD3 |
| AGFG1 | CYBB |
| AGFG1 | C8orf88 |
| AGFG1 | AK2 |
| AGFG1 | BIRC2 |
| AGFG1 | GNB4 |
| AGFG1 | SELT |
| AGFG1 | DCUN1D1 |
| AGFG1 | ASPH |
| AGFG1 | SETD7 |
| AGFG1 | ARL15 |
| AGFG1 | MMP19 |
| AGFG1 | CEP164 |
| AGFG1 | PLIN2 |
| AGFG1 | GPER1 |
| AGFG1 | BROX |
| AGFG1 | C5orf45 |
| AGFG1 | SUMF1 |
| AGFG1 | FBN2 |
| AGFG1 | ARRDC4 |
| AGFG1 | DNAJA2 |
| AGFG1 | DMRT2 |
| AGFG1 | ZNF287 |
| AGFG1 | CYFIP1 |
| AGFG1 | TPM2 |
| AGFG1 | BCAT1 |
| AGFG1 | FAM199X |
| AGFG1 | ADRBK2 |
| AGFG1 | PNPLA3 |
| AGFG1 | LRTOMT |
| AGFG1 | GOLPH3 |
| AGFG1 | ARF3 |
| AGFG1 | TTC28 |
| AGFG1 | SCPEP1 |
| AGFG1 | CMTM4 |
| AGFG1 | ZNF483 |
| AGFG1 | ALKBH2 |
| AGFG1 | F8 |
| AGFG1 | KLHDC3 |
| AGFG1 | FLVCR2 |
| AGFG1 | PIEZO1 |
| AGFG1 | MS4A4A |
| AGFG1 | COL5A3 |
| AGFG1 | CHMP7 |
| AGFG1 | ACP6 |
| AGFG1 | BRCA1 |
| AGFG1 | TRAPPC6A |
| AGFG1 | EPB41L3 |
| AGFG1 | NPEPL1 |
| AGFG1 | PAPSS1 |
| AGFG1 | PDE1C |
| AGFG1 | DDIT3 |
| AGFG1 | MLC1 |
| AGFG1 | LDLRAD3 |
| AGFG1 | FAM110B |
| AGFG1 | RRM2B |
| AGFG1 | PHTF1 |
| AGFG1 | CLIC4 |
| AGFG1 | SLC1A3 |
| AGFG1 | TLR10 |
| AGFG1 | DSE |
| AGFG1 | SGMS2 |
| AGFG1 | PRMT5 |
| AGFG1 | MTL5 |
| AGFG1 | KNOP1 |
| AGFG1 | VAV3 |
| AGFG1 | ERI3 |
| AGFG1 | CREB1 |
| AGFG1 | GAPT |
| AGFG1 | ATP10D |
| AGFG1 | LIN54 |
| AGFG1 | CC2D2B |
| AGFG1 | ENOX1 |
| AGFG1 | TACR1 |
| AGFG1 | MATK |
| AGFG1 | MPZL2 |
| AGFG1 | SALL3 |
| AGFG1 | RNASE4 |
| AGFG1 | PHACTR1 |
| AGFG1 | NETO2 |
| AGFG1 | FLT3 |
| AGFG1 | NKAP |
| AGFG1 | MOSPD1 |
| AGFG1 | CD27 |
| AGFG1 | PAPSS2 |
| AGFG1 | IFIT5 |
| AGFG1 | DLGAP1-AS2 |
| AGFG1 | RP1-193H18.2 |
| AGFG1 | LINC00550 |
| AGFG1 | LINC00282 |
| AGFG1 | DKFZP434L187 |
| AGFG1 | RP1-30M3.5 |
| AGFG1 | RP11-111K18.2 |
| AGFG1 | RP11-2E11.9 |
| AGFG1 | RP11-749H17.2 |
| AGFG1 | RP11-399O19.9 |
| AGFG1 | KB-431C1.4 |
| AGFG1 | RP11-1024P17.1 |
| AGFG1 | SNRK-AS1 |
| AGFG1 | AC005785.2 |
| AGFG1 | RP11-476D10.1 |
| KPNA2 | UBE2A |
| KPNA2 | GAB1 |
| KPNA2 | FAM102A |
| KPNA2 | MAPK13 |
| KPNA2 | MSL3 |
| KPNA2 | ATF6 |
| KPNA2 | ETF1 |
| KPNA2 | PPFIA1 |
| KPNA2 | PRRG4 |
| KPNA2 | JAK1 |
| KPNA2 | NSDHL |
| KPNA2 | SF3A1 |
| KPNA2 | BMP2K |
| KPNA2 | SAP30 |
| KPNA2 | SLK |
| KPNA2 | FAM120AOS |
| KPNA2 | GIT2 |
| KPNA2 | CUL4B |
| KPNA2 | VCPIP1 |
| KPNA2 | CRIPT |
| KPNA2 | PIK3C3 |
| KPNA2 | TNFRSF25 |
| KPNA2 | ID3 |
| KPNA2 | ST6GALNAC3 |
| KPNA2 | DHX40 |
| KPNA2 | KDM3B |
| KPNA2 | SLC12A6 |
| KPNA2 | TANC2 |
| KPNA2 | IDH1 |
| KPNA2 | PAPOLA |
| KPNA2 | ATXN2L |
| KPNA2 | FEZ2 |
| KPNA2 | EAPP |
| KPNA2 | HOOK3 |
| KPNA2 | CD163 |
| KPNA2 | SOAT1 |
| KPNA2 | MED31 |
| KPNA2 | SENP1 |
| KPNA2 | NSMAF |
| KPNA2 | PPP5C |
| KPNA2 | ATN1 |
| KPNA2 | CREBRF |
| KPNA2 | VPS41 |
| KPNA2 | PSMD12 |
| KPNA2 | SHC4 |
| KPNA2 | KIAA2013 |
| KPNA2 | SAP30L |
| KPNA2 | NAGA |
| KPNA2 | CLEC5A |
| KPNA2 | TSHZ3 |
| KPNA2 | ITFG2 |
| KPNA2 | STAM2 |
| KPNA2 | SPRED2 |
| KPNA2 | PSMC3 |
| KPNA2 | SCARB2 |
| KPNA2 | IPCEF1 |
| KPNA2 | TMEM260 |
| KPNA2 | TMED8 |
| KPNA2 | LPCAT2 |
| KPNA2 | WSB1 |
| KPNA2 | SCCPDH |
| KPNA2 | C7orf73 |
| KPNA2 | BAZ2B |
| KPNA2 | NDST3 |
| KPNA2 | GORASP2 |
| KPNA2 | EIF2AK2 |
| KPNA2 | TBCC |
| KPNA2 | GNAI3 |
| KPNA2 | MPPED2 |
| KPNA2 | NEURL1 |
| KPNA2 | SFN |
| KPNA2 | INTS6 |
| KPNA2 | TFG |
| KPNA2 | MEF2A |
| KPNA2 | ASCC1 |
| KPNA2 | PRMT1 |
| KPNA2 | GOLPH3L |
| KPNA2 | CLIC3 |
| KPNA2 | UBE2E3 |
| KPNA2 | MSX1 |
| KPNA2 | AREL1 |
| KPNA2 | S1PR3 |
| KPNA2 | SCYL2 |
| KPNA2 | SIX3 |
| KPNA2 | SLX4IP |
| KPNA2 | RNF165 |
| KPNA2 | NBN |
| KPNA2 | PLD4 |
| KPNA2 | OGFOD3 |
| KPNA2 | CYBB |
| KPNA2 | C8orf88 |
| KPNA2 | AK2 |
| KPNA2 | BIRC2 |
| KPNA2 | GNB4 |
| KPNA2 | SELT |
| KPNA2 | DCUN1D1 |
| KPNA2 | ASPH |
| KPNA2 | SETD7 |
| KPNA2 | ARL15 |
| KPNA2 | MMP19 |
| KPNA2 | CEP164 |
| KPNA2 | PLIN2 |
| KPNA2 | GPER1 |
| KPNA2 | BROX |
| KPNA2 | C5orf45 |
| KPNA2 | SUMF1 |
| KPNA2 | FBN2 |
| KPNA2 | ARRDC4 |
| KPNA2 | DNAJA2 |
| KPNA2 | DMRT2 |
| KPNA2 | ZNF287 |
| KPNA2 | CYFIP1 |
| KPNA2 | TPM2 |
| KPNA2 | BCAT1 |
| KPNA2 | FAM199X |
| KPNA2 | ADRBK2 |
| KPNA2 | PNPLA3 |
| KPNA2 | LRTOMT |
| KPNA2 | GOLPH3 |
| KPNA2 | ARF3 |
| KPNA2 | TTC28 |
| KPNA2 | SCPEP1 |
| KPNA2 | CMTM4 |
| KPNA2 | ZNF483 |
| KPNA2 | ALKBH2 |
| KPNA2 | F8 |
| KPNA2 | KLHDC3 |
| KPNA2 | FLVCR2 |
| KPNA2 | MS4A4A |
| KPNA2 | CHMP7 |
| KPNA2 | ACP6 |
| KPNA2 | BRCA1 |
| KPNA2 | TRAPPC6A |
| KPNA2 | EPB41L3 |
| KPNA2 | NPEPL1 |
| KPNA2 | PAPSS1 |
| KPNA2 | PDE1C |
| KPNA2 | DDIT3 |
| KPNA2 | NOS3 |
| KPNA2 | MLC1 |
| KPNA2 | LDLRAD3 |
| KPNA2 | FAM110B |
| KPNA2 | RRM2B |
| KPNA2 | PHTF1 |
| KPNA2 | CLIC4 |
| KPNA2 | SLC1A3 |
| KPNA2 | TLR10 |
| KPNA2 | DSE |
| KPNA2 | SGMS2 |
| KPNA2 | PRMT5 |
| KPNA2 | MTL5 |
| KPNA2 | KNOP1 |
| KPNA2 | VAV3 |
| KPNA2 | ERI3 |
| KPNA2 | CREB1 |
| KPNA2 | GAPT |
| KPNA2 | ATP10D |
| KPNA2 | CC2D2B |
| KPNA2 | ENOX1 |
| KPNA2 | TACR1 |
| KPNA2 | MATK |
| KPNA2 | RLTPR |
| KPNA2 | MPZL2 |
| KPNA2 | SALL3 |
| KPNA2 | RNASE4 |
| KPNA2 | PHACTR1 |
| KPNA2 | NETO2 |
| KPNA2 | FLT3 |
| KPNA2 | NKAP |
| KPNA2 | MOSPD1 |
| KPNA2 | CD27 |
| KPNA2 | PAPSS2 |
| KPNA2 | IFIT5 |
| KPNA2 | DLGAP1-AS2 |
| KPNA2 | RP1-193H18.2 |
| KPNA2 | LINC00550 |
| KPNA2 | LINC00282 |
| KPNA2 | DKFZP434L187 |
| KPNA2 | RP1-30M3.5 |
| KPNA2 | RP11-111K18.2 |
| KPNA2 | RP11-2E11.9 |
| KPNA2 | RP11-749H17.2 |
| KPNA2 | RP11-399O19.9 |
| KPNA2 | KB-431C1.4 |
| KPNA2 | RP11-1024P17.1 |
| KPNA2 | SNRK-AS1 |
| KPNA2 | AC005785.2 |
| KPNA2 | RP11-476D10.1 |
| UBE2A | GAB1 |
| UBE2A | FAM102A |
| UBE2A | MAPK13 |
| UBE2A | MSL3 |
| UBE2A | ATF6 |
| UBE2A | ETF1 |
| UBE2A | PPFIA1 |
| UBE2A | PRRG4 |
| UBE2A | JAK1 |
| UBE2A | NSDHL |
| UBE2A | SF3A1 |
| UBE2A | BMP2K |
| UBE2A | SAP30 |
| UBE2A | SLK |
| UBE2A | FAM120AOS |
| UBE2A | GIT2 |
| UBE2A | CUL4B |
| UBE2A | VCPIP1 |
| UBE2A | CRIPT |
| UBE2A | PIK3C3 |
| UBE2A | TNFRSF25 |
| UBE2A | ID3 |
| UBE2A | ST6GALNAC3 |
| UBE2A | DHX40 |
| UBE2A | KDM3B |
| UBE2A | SLC12A6 |
| UBE2A | TANC2 |
| UBE2A | IDH1 |
| UBE2A | PAPOLA |
| UBE2A | ATXN2L |
| UBE2A | FEZ2 |
| UBE2A | EAPP |
| UBE2A | HOOK3 |
| UBE2A | CD163 |
| UBE2A | SOAT1 |
| UBE2A | MED31 |
| UBE2A | SENP1 |
| UBE2A | NSMAF |
| UBE2A | PPP5C |
| UBE2A | ATN1 |
| UBE2A | CREBRF |
| UBE2A | VPS41 |
| UBE2A | PSMD12 |
| UBE2A | KIAA2013 |
| UBE2A | SAP30L |
| UBE2A | NAGA |
| UBE2A | CLEC5A |
| UBE2A | TSHZ3 |
| UBE2A | ITFG2 |
| UBE2A | STAM2 |
| UBE2A | SPRED2 |
| UBE2A | PSMC3 |
| UBE2A | SCARB2 |
| UBE2A | IPCEF1 |
| UBE2A | TMEM260 |
| UBE2A | TMED8 |
| UBE2A | LPCAT2 |
| UBE2A | WSB1 |
| UBE2A | SCCPDH |
| UBE2A | C7orf73 |
| UBE2A | BAZ2B |
| UBE2A | NDST3 |
| UBE2A | GORASP2 |
| UBE2A | EIF2AK2 |
| UBE2A | TBCC |
| UBE2A | GNAI3 |
| UBE2A | MPPED2 |
| UBE2A | NEURL1 |
| UBE2A | SFN |
| UBE2A | INTS6 |
| UBE2A | TFG |
| UBE2A | MEF2A |
| UBE2A | ASCC1 |
| UBE2A | PRMT1 |
| UBE2A | GOLPH3L |
| UBE2A | CLIC3 |
| UBE2A | UBE2E3 |
| UBE2A | MSX1 |
| UBE2A | AREL1 |
| UBE2A | S1PR3 |
| UBE2A | SCYL2 |
| UBE2A | SLX4IP |
| UBE2A | RNF165 |
| UBE2A | NBN |
| UBE2A | PLD4 |
| UBE2A | OGFOD3 |
| UBE2A | CYBB |
| UBE2A | AK2 |
| UBE2A | BIRC2 |
| UBE2A | GNB4 |
| UBE2A | SELT |
| UBE2A | DCUN1D1 |
| UBE2A | ASPH |
| UBE2A | SETD7 |
| UBE2A | ARL15 |
| UBE2A | MMP19 |
| UBE2A | CEP164 |
| UBE2A | PLIN2 |
| UBE2A | GPER1 |
| UBE2A | BROX |
| UBE2A | SUMF1 |
| UBE2A | FBN2 |
| UBE2A | ARRDC4 |
| UBE2A | DNAJA2 |
| UBE2A | ZNF287 |
| UBE2A | CYFIP1 |
| UBE2A | TPM2 |
| UBE2A | BCAT1 |
| UBE2A | FAM199X |
| UBE2A | ADRBK2 |
| UBE2A | PNPLA3 |
| UBE2A | LRTOMT |
| UBE2A | GOLPH3 |
| UBE2A | TTC28 |
| UBE2A | SCPEP1 |
| UBE2A | CMTM4 |
| UBE2A | ALKBH2 |
| UBE2A | F8 |
| UBE2A | KLHDC3 |
| UBE2A | FLVCR2 |
| UBE2A | MS4A4A |
| UBE2A | CHMP7 |
| UBE2A | ACP6 |
| UBE2A | BRCA1 |
| UBE2A | TRAPPC6A |
| UBE2A | NPEPL1 |
| UBE2A | PAPSS1 |
| UBE2A | DDIT3 |
| UBE2A | MLC1 |
| UBE2A | FAM110B |
| UBE2A | RRM2B |
| UBE2A | PHTF1 |
| UBE2A | CLIC4 |
| UBE2A | SLC1A3 |
| UBE2A | TLR10 |
| UBE2A | DSE |
| UBE2A | PRMT5 |
| UBE2A | MTL5 |
| UBE2A | KNOP1 |
| UBE2A | VAV3 |
| UBE2A | CREB1 |
| UBE2A | GAPT |
| UBE2A | ATP10D |
| UBE2A | CC2D2B |
| UBE2A | ENOX1 |
| UBE2A | MATK |
| UBE2A | MPZL2 |
| UBE2A | RNASE4 |
| UBE2A | PHACTR1 |
| UBE2A | NETO2 |
| UBE2A | FLT3 |
| UBE2A | NKAP |
| UBE2A | MOSPD1 |
| UBE2A | PAPSS2 |
| UBE2A | IFIT5 |
| UBE2A | DLGAP1-AS2 |
| UBE2A | RP1-193H18.2 |
| UBE2A | LINC00550 |
| UBE2A | LINC00282 |
| UBE2A | DKFZP434L187 |
| UBE2A | RP1-30M3.5 |
| UBE2A | RP11-111K18.2 |
| UBE2A | RP11-2E11.9 |
| UBE2A | RP11-399O19.9 |
| UBE2A | KB-431C1.4 |
| UBE2A | RP11-1024P17.1 |
| UBE2A | SNRK-AS1 |
| UBE2A | AC005785.2 |
| UBE2A | RP11-476D10.1 |
| GAB1 | FAM102A |
| GAB1 | MAPK13 |
| GAB1 | MSL3 |
| GAB1 | ATF6 |
| GAB1 | ETF1 |
| GAB1 | PPFIA1 |
| GAB1 | PRRG4 |
| GAB1 | JAK1 |
| GAB1 | NSDHL |
| GAB1 | SF3A1 |
| GAB1 | BMP2K |
| GAB1 | SAP30 |
| GAB1 | SLK |
| GAB1 | FAM120AOS |
| GAB1 | GIT2 |
| GAB1 | CUL4B |
| GAB1 | VCPIP1 |
| GAB1 | CRIPT |
| GAB1 | PIK3C3 |
| GAB1 | TNFRSF25 |
| GAB1 | ID3 |
| GAB1 | ST6GALNAC3 |
| GAB1 | DHX40 |
| GAB1 | KDM3B |
| GAB1 | SLC12A6 |
| GAB1 | TANC2 |
| GAB1 | IDH1 |
| GAB1 | PAPOLA |
| GAB1 | ATXN2L |
| GAB1 | FEZ2 |
| GAB1 | EAPP |
| GAB1 | HOOK3 |
| GAB1 | CD163 |
| GAB1 | SOAT1 |
| GAB1 | MED31 |
| GAB1 | SENP1 |
| GAB1 | NSMAF |
| GAB1 | PPP5C |
| GAB1 | ATN1 |
| GAB1 | CREBRF |
| GAB1 | VPS41 |
| GAB1 | PSMD12 |
| GAB1 | KIAA2013 |
| GAB1 | SAP30L |
| GAB1 | NAGA |
| GAB1 | CLEC5A |
| GAB1 | TSHZ3 |
| GAB1 | ITFG2 |
| GAB1 | STAM2 |
| GAB1 | SPRED2 |
| GAB1 | PSMC3 |
| GAB1 | SCARB2 |
| GAB1 | IPCEF1 |
| GAB1 | TMEM260 |
| GAB1 | TMED8 |
| GAB1 | LPCAT2 |
| GAB1 | WSB1 |
| GAB1 | SCCPDH |
| GAB1 | C7orf73 |
| GAB1 | BAZ2B |
| GAB1 | GORASP2 |
| GAB1 | EIF2AK2 |
| GAB1 | TBCC |
| GAB1 | GNAI3 |
| GAB1 | MPPED2 |
| GAB1 | NEURL1 |
| GAB1 | SFN |
| GAB1 | INTS6 |
| GAB1 | TFG |
| GAB1 | MEF2A |
| GAB1 | ASCC1 |
| GAB1 | PRMT1 |
| GAB1 | GOLPH3L |
| GAB1 | CLIC3 |
| GAB1 | UBE2E3 |
| GAB1 | MSX1 |
| GAB1 | AREL1 |
| GAB1 | S1PR3 |
| GAB1 | SCYL2 |
| GAB1 | SLX4IP |
| GAB1 | RNF165 |
| GAB1 | NBN |
| GAB1 | PLD4 |
| GAB1 | OGFOD3 |
| GAB1 | CYBB |
| GAB1 | C8orf88 |
| GAB1 | AK2 |
| GAB1 | BIRC2 |
| GAB1 | GNB4 |
| GAB1 | SELT |
| GAB1 | DCUN1D1 |
| GAB1 | ASPH |
| GAB1 | SETD7 |
| GAB1 | ARL15 |
| GAB1 | MMP19 |
| GAB1 | CEP164 |
| GAB1 | PLIN2 |
| GAB1 | GPER1 |
| GAB1 | BROX |
| GAB1 | SUMF1 |
| GAB1 | FBN2 |
| GAB1 | ARRDC4 |
| GAB1 | DNAJA2 |
| GAB1 | ZNF287 |
| GAB1 | CYFIP1 |
| GAB1 | TPM2 |
| GAB1 | BCAT1 |
| GAB1 | FAM199X |
| GAB1 | ADRBK2 |
| GAB1 | LRTOMT |
| GAB1 | GOLPH3 |
| GAB1 | ARF3 |
| GAB1 | TTC28 |
| GAB1 | SCPEP1 |
| GAB1 | CMTM4 |
| GAB1 | ALKBH2 |
| GAB1 | F8 |
| GAB1 | FLVCR2 |
| GAB1 | MS4A4A |
| GAB1 | CHMP7 |
| GAB1 | ACP6 |
| GAB1 | BRCA1 |
| GAB1 | TRAPPC6A |
| GAB1 | NPEPL1 |
| GAB1 | PAPSS1 |
| GAB1 | DDIT3 |
| GAB1 | NOS3 |
| GAB1 | MLC1 |
| GAB1 | FAM110B |
| GAB1 | RRM2B |
| GAB1 | PHTF1 |
| GAB1 | CLIC4 |
| GAB1 | TLR10 |
| GAB1 | DSE |
| GAB1 | SGMS2 |
| GAB1 | PRMT5 |
| GAB1 | MTL5 |
| GAB1 | KNOP1 |
| GAB1 | VAV3 |
| GAB1 | CREB1 |
| GAB1 | GAPT |
| GAB1 | ATP10D |
| GAB1 | CC2D2B |
| GAB1 | ENOX1 |
| GAB1 | MATK |
| GAB1 | MPZL2 |
| GAB1 | RNASE4 |
| GAB1 | PHACTR1 |
| GAB1 | FLT3 |
| GAB1 | NKAP |
| GAB1 | CD27 |
| GAB1 | DLGAP1-AS2 |
| GAB1 | RP1-193H18.2 |
| GAB1 | LINC00282 |
| GAB1 | DKFZP434L187 |
| GAB1 | RP1-30M3.5 |
| GAB1 | RP11-111K18.2 |
| GAB1 | RP11-2E11.9 |
| GAB1 | RP11-399O19.9 |
| GAB1 | KB-431C1.4 |
| GAB1 | RP11-1024P17.1 |
| GAB1 | SNRK-AS1 |
| GAB1 | AC005785.2 |
| GAB1 | RP11-476D10.1 |
| FAM102A | MAPK13 |
| FAM102A | ATF6 |
| FAM102A | ETF1 |
| FAM102A | PPFIA1 |
| FAM102A | PRRG4 |
| FAM102A | JAK1 |
| FAM102A | SF3A1 |
| FAM102A | BMP2K |
| FAM102A | SAP30 |
| FAM102A | FAM120AOS |
| FAM102A | GIT2 |
| FAM102A | CUL4B |
| FAM102A | VCPIP1 |
| FAM102A | PIK3C3 |
| FAM102A | TNFRSF25 |
| FAM102A | ID3 |
| FAM102A | DHX40 |
| FAM102A | SLC12A6 |
| FAM102A | TANC2 |
| FAM102A | IDH1 |
| FAM102A | PAPOLA |
| FAM102A | ATXN2L |
| FAM102A | FEZ2 |
| FAM102A | SOAT1 |
| FAM102A | NSMAF |
| FAM102A | ATN1 |
| FAM102A | VPS41 |
| FAM102A | KIAA2013 |
| FAM102A | NAGA |
| FAM102A | TSHZ3 |
| FAM102A | ITFG2 |
| FAM102A | LPCAT2 |
| FAM102A | BAZ2B |
| FAM102A | GNAI3 |
| FAM102A | SFN |
| FAM102A | PRMT1 |
| FAM102A | AREL1 |
| FAM102A | MMP19 |
| FAM102A | C5orf45 |
| FAM102A | CYFIP1 |
| FAM102A | TPM2 |
| FAM102A | CMTM4 |
| FAM102A | ALKBH2 |
| FAM102A | FLVCR2 |
| FAM102A | CHMP7 |
| FAM102A | TRAPPC6A |
| FAM102A | NOS3 |
| FAM102A | ERI3 |
| FAM102A | RLTPR |
| FAM102A | CD27 |
| FAM102A | CCR7 |
| FAM102A | DLGAP1-AS2 |
| FAM102A | RP1-193H18.2 |
| FAM102A | RP11-2E11.9 |
| MAPK13 | MSL3 |
| MAPK13 | ATF6 |
| MAPK13 | ETF1 |
| MAPK13 | PPFIA1 |
| MAPK13 | PRRG4 |
| MAPK13 | JAK1 |
| MAPK13 | NSDHL |
| MAPK13 | SF3A1 |
| MAPK13 | BMP2K |
| MAPK13 | SAP30 |
| MAPK13 | SLK |
| MAPK13 | FAM120AOS |
| MAPK13 | GIT2 |
| MAPK13 | CUL4B |
| MAPK13 | VCPIP1 |
| MAPK13 | CRIPT |
| MAPK13 | PIK3C3 |
| MAPK13 | TNFRSF25 |
| MAPK13 | ID3 |
| MAPK13 | ST6GALNAC3 |
| MAPK13 | DHX40 |
| MAPK13 | KDM3B |
| MAPK13 | SLC12A6 |
| MAPK13 | TANC2 |
| MAPK13 | IDH1 |
| MAPK13 | PAPOLA |
| MAPK13 | ATXN2L |
| MAPK13 | FEZ2 |
| MAPK13 | EAPP |
| MAPK13 | HOOK3 |
| MAPK13 | CD163 |
| MAPK13 | SOAT1 |
| MAPK13 | MED31 |
| MAPK13 | SENP1 |
| MAPK13 | NSMAF |
| MAPK13 | PPP5C |
| MAPK13 | ATN1 |
| MAPK13 | CREBRF |
| MAPK13 | VPS41 |
| MAPK13 | PSMD12 |
| MAPK13 | SHC4 |
| MAPK13 | KIAA2013 |
| MAPK13 | SAP30L |
| MAPK13 | NAGA |
| MAPK13 | CLEC5A |
| MAPK13 | TSHZ3 |
| MAPK13 | ITFG2 |
| MAPK13 | STAM2 |
| MAPK13 | SPRED2 |
| MAPK13 | PSMC3 |
| MAPK13 | SCARB2 |
| MAPK13 | IPCEF1 |
| MAPK13 | TMEM260 |
| MAPK13 | TMED8 |
| MAPK13 | LPCAT2 |
| MAPK13 | WSB1 |
| MAPK13 | SCCPDH |
| MAPK13 | BAZ2B |
| MAPK13 | NDST3 |
| MAPK13 | EIF2AK2 |
| MAPK13 | TBCC |
| MAPK13 | GNAI3 |
| MAPK13 | MPPED2 |
| MAPK13 | NEURL1 |
| MAPK13 | SFN |
| MAPK13 | INTS6 |
| MAPK13 | TFG |
| MAPK13 | MEF2A |
| MAPK13 | ASCC1 |
| MAPK13 | PRMT1 |
| MAPK13 | GOLPH3L |
| MAPK13 | CLIC3 |
| MAPK13 | UBE2E3 |
| MAPK13 | AREL1 |
| MAPK13 | S1PR3 |
| MAPK13 | SCYL2 |
| MAPK13 | SLX4IP |
| MAPK13 | RNF165 |
| MAPK13 | NBN |
| MAPK13 | PLD4 |
| MAPK13 | OGFOD3 |
| MAPK13 | CYBB |
| MAPK13 | C8orf88 |
| MAPK13 | AK2 |
| MAPK13 | BIRC2 |
| MAPK13 | GNB4 |
| MAPK13 | SELT |
| MAPK13 | DCUN1D1 |
| MAPK13 | ASPH |
| MAPK13 | ARL15 |
| MAPK13 | MMP19 |
| MAPK13 | CEP164 |
| MAPK13 | PLIN2 |
| MAPK13 | BROX |
| MAPK13 | SUMF1 |
| MAPK13 | ARRDC4 |
| MAPK13 | DNAJA2 |
| MAPK13 | CYFIP1 |
| MAPK13 | TPM2 |
| MAPK13 | FAM199X |
| MAPK13 | ADRBK2 |
| MAPK13 | LRTOMT |
| MAPK13 | GOLPH3 |
| MAPK13 | TTC28 |
| MAPK13 | SCPEP1 |
| MAPK13 | CMTM4 |
| MAPK13 | F8 |
| MAPK13 | FLVCR2 |
| MAPK13 | MS4A4A |
| MAPK13 | ACP6 |
| MAPK13 | BRCA1 |
| MAPK13 | NPEPL1 |
| MAPK13 | PAPSS1 |
| MAPK13 | DDIT3 |
| MAPK13 | RRM2B |
| MAPK13 | PHTF1 |
| MAPK13 | TLR10 |
| MAPK13 | PRMT5 |
| MAPK13 | MTL5 |
| MAPK13 | VAV3 |
| MAPK13 | CREB1 |
| MAPK13 | GAPT |
| MAPK13 | ATP10D |
| MAPK13 | ENOX1 |
| MAPK13 | PHACTR1 |
| MAPK13 | NKAP |
| MAPK13 | DLGAP1-AS2 |
| MAPK13 | RP1-193H18.2 |
| MAPK13 | LINC00282 |
| MAPK13 | DKFZP434L187 |
| MAPK13 | RP1-30M3.5 |
| MAPK13 | RP11-111K18.2 |
| MAPK13 | RP11-2E11.9 |
| MAPK13 | RP11-399O19.9 |
| MAPK13 | KB-431C1.4 |
| MAPK13 | RP11-1024P17.1 |
| MAPK13 | SNRK-AS1 |
| MAPK13 | RP11-476D10.1 |
| MSL3 | ATF6 |
| MSL3 | ETF1 |
| MSL3 | PPFIA1 |
| MSL3 | PRRG4 |
| MSL3 | JAK1 |
| MSL3 | NSDHL |
| MSL3 | BMP2K |
| MSL3 | SAP30 |
| MSL3 | SLK |
| MSL3 | FAM120AOS |
| MSL3 | GIT2 |
| MSL3 | CUL4B |
| MSL3 | VCPIP1 |
| MSL3 | CRIPT |
| MSL3 | PIK3C3 |
| MSL3 | ID3 |
| MSL3 | ST6GALNAC3 |
| MSL3 | DHX40 |
| MSL3 | KDM3B |
| MSL3 | SLC12A6 |
| MSL3 | TANC2 |
| MSL3 | IDH1 |
| MSL3 | PAPOLA |
| MSL3 | ATXN2L |
| MSL3 | FEZ2 |
| MSL3 | EAPP |
| MSL3 | HOOK3 |
| MSL3 | CD163 |
| MSL3 | SOAT1 |
| MSL3 | MED31 |
| MSL3 | SENP1 |
| MSL3 | NSMAF |
| MSL3 | PPP5C |
| MSL3 | ATN1 |
| MSL3 | CREBRF |
| MSL3 | VPS41 |
| MSL3 | PSMD12 |
| MSL3 | KIAA2013 |
| MSL3 | SAP30L |
| MSL3 | NAGA |
| MSL3 | CLEC5A |
| MSL3 | TSHZ3 |
| MSL3 | ITFG2 |
| MSL3 | STAM2 |
| MSL3 | SPRED2 |
| MSL3 | PSMC3 |
| MSL3 | SCARB2 |
| MSL3 | IPCEF1 |
| MSL3 | TMEM260 |
| MSL3 | TMED8 |
| MSL3 | LPCAT2 |
| MSL3 | WSB1 |
| MSL3 | SCCPDH |
| MSL3 | C7orf73 |
| MSL3 | BAZ2B |
| MSL3 | NDST3 |
| MSL3 | GORASP2 |
| MSL3 | EIF2AK2 |
| MSL3 | TBCC |
| MSL3 | GNAI3 |
| MSL3 | MPPED2 |
| MSL3 | NEURL1 |
| MSL3 | SFN |
| MSL3 | INTS6 |
| MSL3 | TFG |
| MSL3 | MEF2A |
| MSL3 | ASCC1 |
| MSL3 | PRMT1 |
| MSL3 | GOLPH3L |
| MSL3 | CLIC3 |
| MSL3 | UBE2E3 |
| MSL3 | MSX1 |
| MSL3 | AREL1 |
| MSL3 | S1PR3 |
| MSL3 | SCYL2 |
| MSL3 | SIX3 |
| MSL3 | SLX4IP |
| MSL3 | RNF165 |
| MSL3 | NBN |
| MSL3 | PLD4 |
| MSL3 | OGFOD3 |
| MSL3 | CYBB |
| MSL3 | C8orf88 |
| MSL3 | BIRC2 |
| MSL3 | GNB4 |
| MSL3 | SELT |
| MSL3 | DCUN1D1 |
| MSL3 | ASPH |
| MSL3 | SETD7 |
| MSL3 | CEP164 |
| MSL3 | GPER1 |
| MSL3 | BROX |
| MSL3 | SUMF1 |
| MSL3 | FBN2 |
| MSL3 | ARRDC4 |
| MSL3 | DNAJA2 |
| MSL3 | CYFIP1 |
| MSL3 | BCAT1 |
| MSL3 | FAM199X |
| MSL3 | ADRBK2 |
| MSL3 | LRTOMT |
| MSL3 | GOLPH3 |
| MSL3 | TTC28 |
| MSL3 | SCPEP1 |
| MSL3 | CMTM4 |
| MSL3 | F8 |
| MSL3 | FLVCR2 |
| MSL3 | MS4A4A |
| MSL3 | ACP6 |
| MSL3 | BRCA1 |
| MSL3 | TRAPPC6A |
| MSL3 | NPEPL1 |
| MSL3 | PAPSS1 |
| MSL3 | DDIT3 |
| MSL3 | MLC1 |
| MSL3 | RRM2B |
| MSL3 | PHTF1 |
| MSL3 | CLIC4 |
| MSL3 | SLC1A3 |
| MSL3 | TLR10 |
| MSL3 | DSE |
| MSL3 | PRMT5 |
| MSL3 | MTL5 |
| MSL3 | VAV3 |
| MSL3 | CREB1 |
| MSL3 | GAPT |
| MSL3 | ATP10D |
| MSL3 | CC2D2B |
| MSL3 | MATK |
| MSL3 | MPZL2 |
| MSL3 | SALL3 |
| MSL3 | RNASE4 |
| MSL3 | PHACTR1 |
| MSL3 | FLT3 |
| MSL3 | NKAP |
| MSL3 | PAPSS2 |
| MSL3 | IFIT5 |
| MSL3 | DLGAP1-AS2 |
| MSL3 | RP1-193H18.2 |
| MSL3 | LINC00282 |
| MSL3 | DKFZP434L187 |
| MSL3 | RP1-30M3.5 |
| MSL3 | RP11-111K18.2 |
| MSL3 | RP11-2E11.9 |
| MSL3 | RP11-399O19.9 |
| MSL3 | KB-431C1.4 |
| MSL3 | RP11-1024P17.1 |
| MSL3 | SNRK-AS1 |
| MSL3 | AC005785.2 |
| MSL3 | RP11-476D10.1 |
| ATF6 | ETF1 |
| ATF6 | PPFIA1 |
| ATF6 | PRRG4 |
| ATF6 | JAK1 |
| ATF6 | NSDHL |
| ATF6 | SF3A1 |
| ATF6 | BMP2K |
| ATF6 | SAP30 |
| ATF6 | SLK |
| ATF6 | FAM120AOS |
| ATF6 | GIT2 |
| ATF6 | CUL4B |
| ATF6 | VCPIP1 |
| ATF6 | CRIPT |
| ATF6 | PIK3C3 |
| ATF6 | TNFRSF25 |
| ATF6 | ID3 |
| ATF6 | ST6GALNAC3 |
| ATF6 | DHX40 |
| ATF6 | KDM3B |
| ATF6 | SLC12A6 |
| ATF6 | TANC2 |
| ATF6 | IDH1 |
| ATF6 | PAPOLA |
| ATF6 | ATXN2L |
| ATF6 | FEZ2 |
| ATF6 | EAPP |
| ATF6 | HOOK3 |
| ATF6 | CD163 |
| ATF6 | SOAT1 |
| ATF6 | MED31 |
| ATF6 | SENP1 |
| ATF6 | NSMAF |
| ATF6 | PPP5C |
| ATF6 | ATN1 |
| ATF6 | CREBRF |
| ATF6 | VPS41 |
| ATF6 | PSMD12 |
| ATF6 | SHC4 |
| ATF6 | KIAA2013 |
| ATF6 | SAP30L |
| ATF6 | NAGA |
| ATF6 | CLEC5A |
| ATF6 | TSHZ3 |
| ATF6 | ITFG2 |
| ATF6 | STAM2 |
| ATF6 | SPRED2 |
| ATF6 | PSMC3 |
| ATF6 | SCARB2 |
| ATF6 | IPCEF1 |
| ATF6 | TMEM260 |
| ATF6 | TMED8 |
| ATF6 | LPCAT2 |
| ATF6 | WSB1 |
| ATF6 | SCCPDH |
| ATF6 | C7orf73 |
| ATF6 | BAZ2B |
| ATF6 | NDST3 |
| ATF6 | GORASP2 |
| ATF6 | EIF2AK2 |
| ATF6 | TBCC |
| ATF6 | GNAI3 |
| ATF6 | MPPED2 |
| ATF6 | NEURL1 |
| ATF6 | SFN |
| ATF6 | INTS6 |
| ATF6 | TFG |
| ATF6 | MEF2A |
| ATF6 | ASCC1 |
| ATF6 | PRMT1 |
| ATF6 | GOLPH3L |
| ATF6 | CLIC3 |
| ATF6 | UBE2E3 |
| ATF6 | MSX1 |
| ATF6 | AREL1 |
| ATF6 | S1PR3 |
| ATF6 | SCYL2 |
| ATF6 | SIX3 |
| ATF6 | SLX4IP |
| ATF6 | RNF165 |
| ATF6 | NBN |
| ATF6 | PLD4 |
| ATF6 | OGFOD3 |
| ATF6 | CYBB |
| ATF6 | C8orf88 |
| ATF6 | AK2 |
| ATF6 | BIRC2 |
| ATF6 | GNB4 |
| ATF6 | SELT |
| ATF6 | DCUN1D1 |
| ATF6 | ASPH |
| ATF6 | SETD7 |
| ATF6 | ARL15 |
| ATF6 | MMP19 |
| ATF6 | CEP164 |
| ATF6 | PLIN2 |
| ATF6 | GPER1 |
| ATF6 | BROX |
| ATF6 | SUMF1 |
| ATF6 | FBN2 |
| ATF6 | ARRDC4 |
| ATF6 | DNAJA2 |
| ATF6 | DMRT2 |
| ATF6 | ZNF287 |
| ATF6 | CYFIP1 |
| ATF6 | TPM2 |
| ATF6 | BCAT1 |
| ATF6 | FAM199X |
| ATF6 | ADRBK2 |
| ATF6 | LRTOMT |
| ATF6 | GOLPH3 |
| ATF6 | ARF3 |
| ATF6 | TTC28 |
| ATF6 | SCPEP1 |
| ATF6 | CMTM4 |
| ATF6 | ZNF483 |
| ATF6 | ALKBH2 |
| ATF6 | F8 |
| ATF6 | FLVCR2 |
| ATF6 | MS4A4A |
| ATF6 | CHMP7 |
| ATF6 | ACP6 |
| ATF6 | BRCA1 |
| ATF6 | TRAPPC6A |
| ATF6 | NPEPL1 |
| ATF6 | PAPSS1 |
| ATF6 | DDIT3 |
| ATF6 | NOS3 |
| ATF6 | MLC1 |
| ATF6 | FAM110B |
| ATF6 | RRM2B |
| ATF6 | PHTF1 |
| ATF6 | CLIC4 |
| ATF6 | SLC1A3 |
| ATF6 | TLR10 |
| ATF6 | DSE |
| ATF6 | SGMS2 |
| ATF6 | PRMT5 |
| ATF6 | MTL5 |
| ATF6 | KNOP1 |
| ATF6 | VAV3 |
| ATF6 | ERI3 |
| ATF6 | CREB1 |
| ATF6 | GAPT |
| ATF6 | ATP10D |
| ATF6 | CC2D2B |
| ATF6 | ENOX1 |
| ATF6 | TACR1 |
| ATF6 | MATK |
| ATF6 | MPZL2 |
| ATF6 | SALL3 |
| ATF6 | RNASE4 |
| ATF6 | PHACTR1 |
| ATF6 | NETO2 |
| ATF6 | FLT3 |
| ATF6 | NKAP |
| ATF6 | SEMG1 |
| ATF6 | IFIT5 |
| ATF6 | DLGAP1-AS2 |
| ATF6 | RP1-193H18.2 |
| ATF6 | LINC00282 |
| ATF6 | DKFZP434L187 |
| ATF6 | RP1-30M3.5 |
| ATF6 | RP11-111K18.2 |
| ATF6 | RP11-2E11.9 |
| ATF6 | RP11-399O19.9 |
| ATF6 | KB-431C1.4 |
| ATF6 | RP11-1024P17.1 |
| ATF6 | SNRK-AS1 |
| ATF6 | AC005785.2 |
| ATF6 | RP11-476D10.1 |
| ETF1 | PPFIA1 |
| ETF1 | PRRG4 |
| ETF1 | JAK1 |
| ETF1 | NSDHL |
| ETF1 | SF3A1 |
| ETF1 | BMP2K |
| ETF1 | SAP30 |
| ETF1 | SLK |
| ETF1 | FAM120AOS |
| ETF1 | GIT2 |
| ETF1 | CUL4B |
| ETF1 | VCPIP1 |
| ETF1 | CRIPT |
| ETF1 | PIK3C3 |
| ETF1 | TNFRSF25 |
| ETF1 | ID3 |
| ETF1 | ST6GALNAC3 |
| ETF1 | DHX40 |
| ETF1 | KDM3B |
| ETF1 | SLC12A6 |
| ETF1 | TANC2 |
| ETF1 | IDH1 |
| ETF1 | PAPOLA |
| ETF1 | ATXN2L |
| ETF1 | FEZ2 |
| ETF1 | EAPP |
| ETF1 | HOOK3 |
| ETF1 | CD163 |
| ETF1 | SOAT1 |
| ETF1 | MED31 |
| ETF1 | SENP1 |
| ETF1 | NSMAF |
| ETF1 | PPP5C |
| ETF1 | ATN1 |
| ETF1 | CREBRF |
| ETF1 | VPS41 |
| ETF1 | PSMD12 |
| ETF1 | SHC4 |
| ETF1 | KIAA2013 |
| ETF1 | SAP30L |
| ETF1 | NAGA |
| ETF1 | CLEC5A |
| ETF1 | TSHZ3 |
| ETF1 | ITFG2 |
| ETF1 | STAM2 |
| ETF1 | SPRED2 |
| ETF1 | PSMC3 |
| ETF1 | SCARB2 |
| ETF1 | IPCEF1 |
| ETF1 | TMEM260 |
| ETF1 | TMED8 |
| ETF1 | LPCAT2 |
| ETF1 | WSB1 |
| ETF1 | SCCPDH |
| ETF1 | C7orf73 |
| ETF1 | BAZ2B |
| ETF1 | NDST3 |
| ETF1 | GORASP2 |
| ETF1 | EIF2AK2 |
| ETF1 | TBCC |
| ETF1 | GNAI3 |
| ETF1 | MPPED2 |
| ETF1 | NEURL1 |
| ETF1 | SFN |
| ETF1 | INTS6 |
| ETF1 | TFG |
| ETF1 | MEF2A |
| ETF1 | ASCC1 |
| ETF1 | PRMT1 |
| ETF1 | GOLPH3L |
| ETF1 | CLIC3 |
| ETF1 | UBE2E3 |
| ETF1 | MSX1 |
| ETF1 | AREL1 |
| ETF1 | S1PR3 |
| ETF1 | SCYL2 |
| ETF1 | SIX3 |
| ETF1 | SLX4IP |
| ETF1 | RNF165 |
| ETF1 | NBN |
| ETF1 | PLD4 |
| ETF1 | OGFOD3 |
| ETF1 | CYBB |
| ETF1 | C8orf88 |
| ETF1 | AK2 |
| ETF1 | BIRC2 |
| ETF1 | GNB4 |
| ETF1 | SELT |
| ETF1 | DCUN1D1 |
| ETF1 | ASPH |
| ETF1 | SETD7 |
| ETF1 | ARL15 |
| ETF1 | MMP19 |
| ETF1 | CEP164 |
| ETF1 | PLIN2 |
| ETF1 | GPER1 |
| ETF1 | BROX |
| ETF1 | C5orf45 |
| ETF1 | SUMF1 |
| ETF1 | FBN2 |
| ETF1 | ARRDC4 |
| ETF1 | DNAJA2 |
| ETF1 | DMRT2 |
| ETF1 | ZNF287 |
| ETF1 | CYFIP1 |
| ETF1 | TPM2 |
| ETF1 | BCAT1 |
| ETF1 | FAM199X |
| ETF1 | ADRBK2 |
| ETF1 | LRTOMT |
| ETF1 | GOLPH3 |
| ETF1 | ARF3 |
| ETF1 | TTC28 |
| ETF1 | SCPEP1 |
| ETF1 | CMTM4 |
| ETF1 | ZNF483 |
| ETF1 | ALKBH2 |
| ETF1 | F8 |
| ETF1 | FLVCR2 |
| ETF1 | MS4A4A |
| ETF1 | COL5A3 |
| ETF1 | CHMP7 |
| ETF1 | ACP6 |
| ETF1 | BRCA1 |
| ETF1 | TRAPPC6A |
| ETF1 | EPB41L3 |
| ETF1 | NPEPL1 |
| ETF1 | PAPSS1 |
| ETF1 | DDIT3 |
| ETF1 | NOS3 |
| ETF1 | MLC1 |
| ETF1 | LDLRAD3 |
| ETF1 | FAM110B |
| ETF1 | RRM2B |
| ETF1 | PHTF1 |
| ETF1 | CLIC4 |
| ETF1 | SLC1A3 |
| ETF1 | TLR10 |
| ETF1 | DSE |
| ETF1 | SGMS2 |
| ETF1 | PRMT5 |
| ETF1 | MTL5 |
| ETF1 | KNOP1 |
| ETF1 | VAV3 |
| ETF1 | ERI3 |
| ETF1 | CREB1 |
| ETF1 | GAPT |
| ETF1 | ATP10D |
| ETF1 | CC2D2B |
| ETF1 | ENOX1 |
| ETF1 | TACR1 |
| ETF1 | MATK |
| ETF1 | MPZL2 |
| ETF1 | SALL3 |
| ETF1 | RNASE4 |
| ETF1 | PHACTR1 |
| ETF1 | NETO2 |
| ETF1 | FLT3 |
| ETF1 | NKAP |
| ETF1 | CD27 |
| ETF1 | PAPSS2 |
| ETF1 | IFIT5 |
| ETF1 | DLGAP1-AS2 |
| ETF1 | RP1-193H18.2 |
| ETF1 | LINC00550 |
| ETF1 | LINC00282 |
| ETF1 | DKFZP434L187 |
| ETF1 | RP1-30M3.5 |
| ETF1 | RP11-111K18.2 |
| ETF1 | RP11-2E11.9 |
| ETF1 | RP11-749H17.2 |
| ETF1 | LINC00032 |
| ETF1 | RP11-399O19.9 |
| ETF1 | KB-431C1.4 |
| ETF1 | RP11-1024P17.1 |
| ETF1 | SNRK-AS1 |
| ETF1 | AC005785.2 |
| ETF1 | RP11-476D10.1 |
| PPFIA1 | PRRG4 |
| PPFIA1 | JAK1 |
| PPFIA1 | NSDHL |
| PPFIA1 | SF3A1 |
| PPFIA1 | BMP2K |
| PPFIA1 | SAP30 |
| PPFIA1 | SLK |
| PPFIA1 | FAM120AOS |
| PPFIA1 | GIT2 |
| PPFIA1 | CUL4B |
| PPFIA1 | VCPIP1 |
| PPFIA1 | CRIPT |
| PPFIA1 | PIK3C3 |
| PPFIA1 | TNFRSF25 |
| PPFIA1 | ID3 |
| PPFIA1 | ST6GALNAC3 |
| PPFIA1 | DHX40 |
| PPFIA1 | KDM3B |
| PPFIA1 | SLC12A6 |
| PPFIA1 | TANC2 |
| PPFIA1 | IDH1 |
| PPFIA1 | PAPOLA |
| PPFIA1 | ATXN2L |
| PPFIA1 | FEZ2 |
| PPFIA1 | EAPP |
| PPFIA1 | HOOK3 |
| PPFIA1 | CD163 |
| PPFIA1 | SOAT1 |
| PPFIA1 | MED31 |
| PPFIA1 | SENP1 |
| PPFIA1 | NSMAF |
| PPFIA1 | PPP5C |
| PPFIA1 | ATN1 |
| PPFIA1 | CREBRF |
| PPFIA1 | VPS41 |
| PPFIA1 | PSMD12 |
| PPFIA1 | KIAA2013 |
| PPFIA1 | SAP30L |
| PPFIA1 | NAGA |
| PPFIA1 | CLEC5A |
| PPFIA1 | TSHZ3 |
| PPFIA1 | ITFG2 |
| PPFIA1 | STAM2 |
| PPFIA1 | SPRED2 |
| PPFIA1 | PSMC3 |
| PPFIA1 | SCARB2 |
| PPFIA1 | IPCEF1 |
| PPFIA1 | TMEM260 |
| PPFIA1 | TMED8 |
| PPFIA1 | LPCAT2 |
| PPFIA1 | WSB1 |
| PPFIA1 | SCCPDH |
| PPFIA1 | C7orf73 |
| PPFIA1 | BAZ2B |
| PPFIA1 | NDST3 |
| PPFIA1 | GORASP2 |
| PPFIA1 | EIF2AK2 |
| PPFIA1 | TBCC |
| PPFIA1 | GNAI3 |
| PPFIA1 | MPPED2 |
| PPFIA1 | NEURL1 |
| PPFIA1 | SFN |
| PPFIA1 | INTS6 |
| PPFIA1 | TFG |
| PPFIA1 | MEF2A |
| PPFIA1 | PRMT1 |
| PPFIA1 | GOLPH3L |
| PPFIA1 | CLIC3 |
| PPFIA1 | UBE2E3 |
| PPFIA1 | MSX1 |
| PPFIA1 | AREL1 |
| PPFIA1 | S1PR3 |
| PPFIA1 | SCYL2 |
| PPFIA1 | SLX4IP |
| PPFIA1 | RNF165 |
| PPFIA1 | NBN |
| PPFIA1 | PLD4 |
| PPFIA1 | OGFOD3 |
| PPFIA1 | CYBB |
| PPFIA1 | C8orf88 |
| PPFIA1 | AK2 |
| PPFIA1 | BIRC2 |
| PPFIA1 | GNB4 |
| PPFIA1 | SELT |
| PPFIA1 | DCUN1D1 |
| PPFIA1 | ASPH |
| PPFIA1 | SETD7 |
| PPFIA1 | ARL15 |
| PPFIA1 | MMP19 |
| PPFIA1 | CEP164 |
| PPFIA1 | GPER1 |
| PPFIA1 | BROX |
| PPFIA1 | C5orf45 |
| PPFIA1 | SUMF1 |
| PPFIA1 | FBN2 |
| PPFIA1 | ARRDC4 |
| PPFIA1 | DNAJA2 |
| PPFIA1 | CYFIP1 |
| PPFIA1 | TPM2 |
| PPFIA1 | BCAT1 |
| PPFIA1 | FAM199X |
| PPFIA1 | ADRBK2 |
| PPFIA1 | LRTOMT |
| PPFIA1 | GOLPH3 |
| PPFIA1 | ARF3 |
| PPFIA1 | TTC28 |
| PPFIA1 | SCPEP1 |
| PPFIA1 | CMTM4 |
| PPFIA1 | ALKBH2 |
| PPFIA1 | F8 |
| PPFIA1 | FLVCR2 |
| PPFIA1 | PIEZO1 |
| PPFIA1 | MS4A4A |
| PPFIA1 | CHMP7 |
| PPFIA1 | ACP6 |
| PPFIA1 | BRCA1 |
| PPFIA1 | TRAPPC6A |
| PPFIA1 | NPEPL1 |
| PPFIA1 | PAPSS1 |
| PPFIA1 | DDIT3 |
| PPFIA1 | NOS3 |
| PPFIA1 | MLC1 |
| PPFIA1 | FAM110B |
| PPFIA1 | RRM2B |
| PPFIA1 | PHTF1 |
| PPFIA1 | CLIC4 |
| PPFIA1 | SLC1A3 |
| PPFIA1 | TLR10 |
| PPFIA1 | DSE |
| PPFIA1 | SGMS2 |
| PPFIA1 | PRMT5 |
| PPFIA1 | MTL5 |
| PPFIA1 | KNOP1 |
| PPFIA1 | VAV3 |
| PPFIA1 | CREB1 |
| PPFIA1 | GAPT |
| PPFIA1 | ATP10D |
| PPFIA1 | CC2D2B |
| PPFIA1 | ENOX1 |
| PPFIA1 | MATK |
| PPFIA1 | MPZL2 |
| PPFIA1 | SALL3 |
| PPFIA1 | RNASE4 |
| PPFIA1 | PHACTR1 |
| PPFIA1 | FLT3 |
| PPFIA1 | NKAP |
| PPFIA1 | CD27 |
| PPFIA1 | DLGAP1-AS2 |
| PPFIA1 | RP1-193H18.2 |
| PPFIA1 | LINC00282 |
| PPFIA1 | DKFZP434L187 |
| PPFIA1 | RP1-30M3.5 |
| PPFIA1 | RP11-111K18.2 |
| PPFIA1 | RP11-2E11.9 |
| PPFIA1 | RP11-399O19.9 |
| PPFIA1 | KB-431C1.4 |
| PPFIA1 | RP11-1024P17.1 |
| PPFIA1 | SNRK-AS1 |
| PPFIA1 | AC005785.2 |
| PPFIA1 | RP11-476D10.1 |
| PRRG4 | JAK1 |
| PRRG4 | NSDHL |
| PRRG4 | SF3A1 |
| PRRG4 | BMP2K |
| PRRG4 | SAP30 |
| PRRG4 | SLK |
| PRRG4 | FAM120AOS |
| PRRG4 | GIT2 |
| PRRG4 | CUL4B |
| PRRG4 | VCPIP1 |
| PRRG4 | CRIPT |
| PRRG4 | PIK3C3 |
| PRRG4 | TNFRSF25 |
| PRRG4 | ID3 |
| PRRG4 | ST6GALNAC3 |
| PRRG4 | DHX40 |
| PRRG4 | KDM3B |
| PRRG4 | SLC12A6 |
| PRRG4 | TANC2 |
| PRRG4 | IDH1 |
| PRRG4 | PAPOLA |
| PRRG4 | ATXN2L |
| PRRG4 | FEZ2 |
| PRRG4 | EAPP |
| PRRG4 | HOOK3 |
| PRRG4 | CD163 |
| PRRG4 | SOAT1 |
| PRRG4 | MED31 |
| PRRG4 | SENP1 |
| PRRG4 | NSMAF |
| PRRG4 | PPP5C |
| PRRG4 | ATN1 |
| PRRG4 | CREBRF |
| PRRG4 | VPS41 |
| PRRG4 | PSMD12 |
| PRRG4 | KIAA2013 |
| PRRG4 | SAP30L |
| PRRG4 | NAGA |
| PRRG4 | CLEC5A |
| PRRG4 | TSHZ3 |
| PRRG4 | ITFG2 |
| PRRG4 | STAM2 |
| PRRG4 | SPRED2 |
| PRRG4 | PSMC3 |
| PRRG4 | SCARB2 |
| PRRG4 | IPCEF1 |
| PRRG4 | TMEM260 |
| PRRG4 | TMED8 |
| PRRG4 | LPCAT2 |
| PRRG4 | WSB1 |
| PRRG4 | SCCPDH |
| PRRG4 | BAZ2B |
| PRRG4 | EIF2AK2 |
| PRRG4 | TBCC |
| PRRG4 | GNAI3 |
| PRRG4 | MPPED2 |
| PRRG4 | NEURL1 |
| PRRG4 | SFN |
| PRRG4 | INTS6 |
| PRRG4 | TFG |
| PRRG4 | MEF2A |
| PRRG4 | ASCC1 |
| PRRG4 | PRMT1 |
| PRRG4 | GOLPH3L |
| PRRG4 | CLIC3 |
| PRRG4 | UBE2E3 |
| PRRG4 | MSX1 |
| PRRG4 | AREL1 |
| PRRG4 | S1PR3 |
| PRRG4 | SCYL2 |
| PRRG4 | SLX4IP |
| PRRG4 | RNF165 |
| PRRG4 | NBN |
| PRRG4 | PLD4 |
| PRRG4 | CYBB |
| PRRG4 | GNB4 |
| PRRG4 | SELT |
| PRRG4 | DCUN1D1 |
| PRRG4 | ASPH |
| PRRG4 | SETD7 |
| PRRG4 | GPER1 |
| PRRG4 | BROX |
| PRRG4 | SUMF1 |
| PRRG4 | FBN2 |
| PRRG4 | ARRDC4 |
| PRRG4 | DNAJA2 |
| PRRG4 | CYFIP1 |
| PRRG4 | TPM2 |
| PRRG4 | BCAT1 |
| PRRG4 | FAM199X |
| PRRG4 | ADRBK2 |
| PRRG4 | LRTOMT |
| PRRG4 | GOLPH3 |
| PRRG4 | ARF3 |
| PRRG4 | TTC28 |
| PRRG4 | SCPEP1 |
| PRRG4 | CMTM4 |
| PRRG4 | ALKBH2 |
| PRRG4 | F8 |
| PRRG4 | FLVCR2 |
| PRRG4 | MS4A4A |
| PRRG4 | CHMP7 |
| PRRG4 | BRCA1 |
| PRRG4 | TRAPPC6A |
| PRRG4 | PAPSS1 |
| PRRG4 | DDIT3 |
| PRRG4 | RRM2B |
| PRRG4 | PHTF1 |
| PRRG4 | CLIC4 |
| PRRG4 | SLC1A3 |
| PRRG4 | DSE |
| PRRG4 | PRMT5 |
| PRRG4 | VAV3 |
| PRRG4 | CREB1 |
| PRRG4 | GAPT |
| PRRG4 | ATP10D |
| PRRG4 | CC2D2B |
| PRRG4 | DLGAP1-AS2 |
| PRRG4 | RP1-193H18.2 |
| PRRG4 | LINC00282 |
| PRRG4 | DKFZP434L187 |
| PRRG4 | RP1-30M3.5 |
| PRRG4 | RP11-111K18.2 |
| PRRG4 | RP11-2E11.9 |
| PRRG4 | RP11-399O19.9 |
| PRRG4 | KB-431C1.4 |
| PRRG4 | SNRK-AS1 |
| PRRG4 | RP11-476D10.1 |
| JAK1 | NSDHL |
| JAK1 | SF3A1 |
| JAK1 | BMP2K |
| JAK1 | SAP30 |
| JAK1 | SLK |
| JAK1 | FAM120AOS |
| JAK1 | GIT2 |
| JAK1 | CUL4B |
| JAK1 | VCPIP1 |
| JAK1 | CRIPT |
| JAK1 | PIK3C3 |
| JAK1 | TNFRSF25 |
| JAK1 | ID3 |
| JAK1 | ST6GALNAC3 |
| JAK1 | DHX40 |
| JAK1 | KDM3B |
| JAK1 | SLC12A6 |
| JAK1 | TANC2 |
| JAK1 | IDH1 |
| JAK1 | PAPOLA |
| JAK1 | ATXN2L |
| JAK1 | FEZ2 |
| JAK1 | EAPP |
| JAK1 | HOOK3 |
| JAK1 | CD163 |
| JAK1 | SOAT1 |
| JAK1 | MED31 |
| JAK1 | SENP1 |
| JAK1 | NSMAF |
| JAK1 | PPP5C |
| JAK1 | ATN1 |
| JAK1 | CREBRF |
| JAK1 | VPS41 |
| JAK1 | PSMD12 |
| JAK1 | KIAA2013 |
| JAK1 | SAP30L |
| JAK1 | NAGA |
| JAK1 | CLEC5A |
| JAK1 | TSHZ3 |
| JAK1 | ITFG2 |
| JAK1 | STAM2 |
| JAK1 | SPRED2 |
| JAK1 | PSMC3 |
| JAK1 | SCARB2 |
| JAK1 | IPCEF1 |
| JAK1 | TMEM260 |
| JAK1 | TMED8 |
| JAK1 | LPCAT2 |
| JAK1 | WSB1 |
| JAK1 | SCCPDH |
| JAK1 | C7orf73 |
| JAK1 | BAZ2B |
| JAK1 | NDST3 |
| JAK1 | EIF2AK2 |
| JAK1 | TBCC |
| JAK1 | GNAI3 |
| JAK1 | MPPED2 |
| JAK1 | NEURL1 |
| JAK1 | SFN |
| JAK1 | INTS6 |
| JAK1 | TFG |
| JAK1 | MEF2A |
| JAK1 | ASCC1 |
| JAK1 | PRMT1 |
| JAK1 | CLIC3 |
| JAK1 | UBE2E3 |
| JAK1 | AREL1 |
| JAK1 | S1PR3 |
| JAK1 | SCYL2 |
| JAK1 | SLX4IP |
| JAK1 | RNF165 |
| JAK1 | NBN |
| JAK1 | PLD4 |
| JAK1 | CYBB |
| JAK1 | C8orf88 |
| JAK1 | GNB4 |
| JAK1 | SELT |
| JAK1 | DCUN1D1 |
| JAK1 | ASPH |
| JAK1 | SETD7 |
| JAK1 | ARL15 |
| JAK1 | MMP19 |
| JAK1 | CEP164 |
| JAK1 | PLIN2 |
| JAK1 | GPER1 |
| JAK1 | BROX |
| JAK1 | SUMF1 |
| JAK1 | FBN2 |
| JAK1 | ARRDC4 |
| JAK1 | DNAJA2 |
| JAK1 | CYFIP1 |
| JAK1 | TPM2 |
| JAK1 | BCAT1 |
| JAK1 | ADRBK2 |
| JAK1 | LRTOMT |
| JAK1 | GOLPH3 |
| JAK1 | ARF3 |
| JAK1 | TTC28 |
| JAK1 | SCPEP1 |
| JAK1 | CMTM4 |
| JAK1 | ALKBH2 |
| JAK1 | F8 |
| JAK1 | FLVCR2 |
| JAK1 | MS4A4A |
| JAK1 | CHMP7 |
| JAK1 | BRCA1 |
| JAK1 | TRAPPC6A |
| JAK1 | NPEPL1 |
| JAK1 | PAPSS1 |
| JAK1 | DDIT3 |
| JAK1 | NOS3 |
| JAK1 | RRM2B |
| JAK1 | PHTF1 |
| JAK1 | CLIC4 |
| JAK1 | DSE |
| JAK1 | SGMS2 |
| JAK1 | PRMT5 |
| JAK1 | MTL5 |
| JAK1 | KNOP1 |
| JAK1 | VAV3 |
| JAK1 | CREB1 |
| JAK1 | GAPT |
| JAK1 | MPZL2 |
| JAK1 | PHACTR1 |
| JAK1 | FLT3 |
| JAK1 | NKAP |
| JAK1 | DLGAP1-AS2 |
| JAK1 | RP1-193H18.2 |
| JAK1 | LINC00282 |
| JAK1 | DKFZP434L187 |
| JAK1 | RP1-30M3.5 |
| JAK1 | RP11-111K18.2 |
| JAK1 | RP11-2E11.9 |
| JAK1 | RP11-399O19.9 |
| JAK1 | KB-431C1.4 |
| JAK1 | RP11-1024P17.1 |
| JAK1 | SNRK-AS1 |
| JAK1 | RP11-476D10.1 |
| NSDHL | BMP2K |
| NSDHL | SAP30 |
| NSDHL | SLK |
| NSDHL | FAM120AOS |
| NSDHL | GIT2 |
| NSDHL | CUL4B |
| NSDHL | VCPIP1 |
| NSDHL | PIK3C3 |
| NSDHL | DHX40 |
| NSDHL | KDM3B |
| NSDHL | TANC2 |
| NSDHL | IDH1 |
| NSDHL | PAPOLA |
| NSDHL | EAPP |
| NSDHL | SOAT1 |
| NSDHL | NSMAF |
| NSDHL | CREBRF |
| NSDHL | VPS41 |
| NSDHL | KIAA2013 |
| NSDHL | STAM2 |
| NSDHL | SCARB2 |
| NSDHL | TMEM260 |
| NSDHL | LPCAT2 |
| NSDHL | BAZ2B |
| NSDHL | TBCC |
| NSDHL | INTS6 |
| NSDHL | MEF2A |
| NSDHL | GNB4 |
| NSDHL | SELT |
| NSDHL | BROX |
| NSDHL | DLGAP1-AS2 |
| NSDHL | RP1-193H18.2 |
| NSDHL | RP1-30M3.5 |
| NSDHL | RP11-111K18.2 |
| NSDHL | RP11-749H17.2 |
| SF3A1 | FAM120AOS |
| SF3A1 | GIT2 |
| SF3A1 | CUL4B |
| SF3A1 | VCPIP1 |
| SF3A1 | PIK3C3 |
| SF3A1 | TNFRSF25 |
| SF3A1 | ID3 |
| SF3A1 | DHX40 |
| SF3A1 | KDM3B |
| SF3A1 | SLC12A6 |
| SF3A1 | TANC2 |
| SF3A1 | FEZ2 |
| SF3A1 | CD163 |
| SF3A1 | VPS41 |
| SF3A1 | KIAA2013 |
| SF3A1 | NAGA |
| SF3A1 | ITFG2 |
| SF3A1 | LPCAT2 |
| SF3A1 | GNAI3 |
| SF3A1 | SFN |
| SF3A1 | AREL1 |
| SF3A1 | TPM2 |
| SF3A1 | ARF3 |
| SF3A1 | CHMP7 |
| SF3A1 | NOS3 |
| SF3A1 | DLGAP1-AS2 |
| SF3A1 | RP1-193H18.2 |
| BMP2K | SAP30 |
| BMP2K | SLK |
| BMP2K | FAM120AOS |
| BMP2K | GIT2 |
| BMP2K | CUL4B |
| BMP2K | VCPIP1 |
| BMP2K | CRIPT |
| BMP2K | PIK3C3 |
| BMP2K | TNFRSF25 |
| BMP2K | ID3 |
| BMP2K | ST6GALNAC3 |
| BMP2K | DHX40 |
| BMP2K | KDM3B |
| BMP2K | SLC12A6 |
| BMP2K | TANC2 |
| BMP2K | IDH1 |
| BMP2K | PAPOLA |
| BMP2K | ATXN2L |
| BMP2K | FEZ2 |
| BMP2K | EAPP |
| BMP2K | HOOK3 |
| BMP2K | CD163 |
| BMP2K | SOAT1 |
| BMP2K | MED31 |
| BMP2K | SENP1 |
| BMP2K | NSMAF |
| BMP2K | PPP5C |
| BMP2K | ATN1 |
| BMP2K | CREBRF |
| BMP2K | VPS41 |
| BMP2K | PSMD12 |
| BMP2K | KIAA2013 |
| BMP2K | SAP30L |
| BMP2K | NAGA |
| BMP2K | CLEC5A |
| BMP2K | TSHZ3 |
| BMP2K | ITFG2 |
| BMP2K | STAM2 |
| BMP2K | SPRED2 |
| BMP2K | PSMC3 |
| BMP2K | SCARB2 |
| BMP2K | IPCEF1 |
| BMP2K | TMEM260 |
| BMP2K | TMED8 |
| BMP2K | LPCAT2 |
| BMP2K | WSB1 |
| BMP2K | SCCPDH |
| BMP2K | C7orf73 |
| BMP2K | BAZ2B |
| BMP2K | EIF2AK2 |
| BMP2K | TBCC |
| BMP2K | GNAI3 |
| BMP2K | MPPED2 |
| BMP2K | NEURL1 |
| BMP2K | SFN |
| BMP2K | INTS6 |
| BMP2K | TFG |
| BMP2K | MEF2A |
| BMP2K | PRMT1 |
| BMP2K | GOLPH3L |
| BMP2K | CLIC3 |
| BMP2K | UBE2E3 |
| BMP2K | MSX1 |
| BMP2K | AREL1 |
| BMP2K | S1PR3 |
| BMP2K | SCYL2 |
| BMP2K | SLX4IP |
| BMP2K | RNF165 |
| BMP2K | NBN |
| BMP2K | OGFOD3 |
| BMP2K | CYBB |
| BMP2K | BIRC2 |
| BMP2K | GNB4 |
| BMP2K | SELT |
| BMP2K | DCUN1D1 |
| BMP2K | ASPH |
| BMP2K | SETD7 |
| BMP2K | ARL15 |
| BMP2K | GPER1 |
| BMP2K | BROX |
| BMP2K | C5orf45 |
| BMP2K | SUMF1 |
| BMP2K | FBN2 |
| BMP2K | ARRDC4 |
| BMP2K | DNAJA2 |
| BMP2K | CYFIP1 |
| BMP2K | TPM2 |
| BMP2K | BCAT1 |
| BMP2K | FAM199X |
| BMP2K | ADRBK2 |
| BMP2K | LRTOMT |
| BMP2K | GOLPH3 |
| BMP2K | ARF3 |
| BMP2K | TTC28 |
| BMP2K | SCPEP1 |
| BMP2K | CMTM4 |
| BMP2K | ALKBH2 |
| BMP2K | F8 |
| BMP2K | FLVCR2 |
| BMP2K | MS4A4A |
| BMP2K | CHMP7 |
| BMP2K | BRCA1 |
| BMP2K | TRAPPC6A |
| BMP2K | EPB41L3 |
| BMP2K | NPEPL1 |
| BMP2K | PAPSS1 |
| BMP2K | DDIT3 |
| BMP2K | MLC1 |
| BMP2K | LDLRAD3 |
| BMP2K | FAM110B |
| BMP2K | RRM2B |
| BMP2K | PHTF1 |
| BMP2K | CLIC4 |
| BMP2K | SLC1A3 |
| BMP2K | TLR10 |
| BMP2K | DSE |
| BMP2K | SGMS2 |
| BMP2K | PRMT5 |
| BMP2K | KNOP1 |
| BMP2K | VAV3 |
| BMP2K | CREB1 |
| BMP2K | GAPT |
| BMP2K | ATP10D |
| BMP2K | CC2D2B |
| BMP2K | MATK |
| BMP2K | MPZL2 |
| BMP2K | RNASE4 |
| BMP2K | PHACTR1 |
| BMP2K | FLT3 |
| BMP2K | NKAP |
| BMP2K | PAPSS2 |
| BMP2K | DLGAP1-AS2 |
| BMP2K | RP1-193H18.2 |
| BMP2K | LINC00282 |
| BMP2K | RP1-30M3.5 |
| BMP2K | RP11-111K18.2 |
| BMP2K | RP11-2E11.9 |
| BMP2K | RP11-399O19.9 |
| BMP2K | KB-431C1.4 |
| BMP2K | RP11-1024P17.1 |
| BMP2K | SNRK-AS1 |
| SAP30 | SLK |
| SAP30 | FAM120AOS |
| SAP30 | GIT2 |
| SAP30 | CUL4B |
| SAP30 | VCPIP1 |
| SAP30 | CRIPT |
| SAP30 | PIK3C3 |
| SAP30 | TNFRSF25 |
| SAP30 | ID3 |
| SAP30 | ST6GALNAC3 |
| SAP30 | DHX40 |
| SAP30 | KDM3B |
| SAP30 | TANC2 |
| SAP30 | IDH1 |
| SAP30 | PAPOLA |
| SAP30 | ATXN2L |
| SAP30 | FEZ2 |
| SAP30 | EAPP |
| SAP30 | HOOK3 |
| SAP30 | CD163 |
| SAP30 | SOAT1 |
| SAP30 | MED31 |
| SAP30 | SENP1 |
| SAP30 | NSMAF |
| SAP30 | PPP5C |
| SAP30 | ATN1 |
| SAP30 | CREBRF |
| SAP30 | VPS41 |
| SAP30 | PSMD12 |
| SAP30 | KIAA2013 |
| SAP30 | SAP30L |
| SAP30 | NAGA |
| SAP30 | CLEC5A |
| SAP30 | STAM2 |
| SAP30 | SPRED2 |
| SAP30 | PSMC3 |
| SAP30 | SCARB2 |
| SAP30 | IPCEF1 |
| SAP30 | TMEM260 |
| SAP30 | TMED8 |
| SAP30 | LPCAT2 |
| SAP30 | WSB1 |
| SAP30 | SCCPDH |
| SAP30 | BAZ2B |
| SAP30 | EIF2AK2 |
| SAP30 | TBCC |
| SAP30 | GNAI3 |
| SAP30 | INTS6 |
| SAP30 | TFG |
| SAP30 | MEF2A |
| SAP30 | CLIC3 |
| SAP30 | MSX1 |
| SAP30 | SCYL2 |
| SAP30 | SLX4IP |
| SAP30 | NBN |
| SAP30 | OGFOD3 |
| SAP30 | GNB4 |
| SAP30 | SELT |
| SAP30 | ASPH |
| SAP30 | GPER1 |
| SAP30 | BROX |
| SAP30 | SUMF1 |
| SAP30 | FBN2 |
| SAP30 | ARRDC4 |
| SAP30 | DNAJA2 |
| SAP30 | GOLPH3 |
| SAP30 | SCPEP1 |
| SAP30 | CMTM4 |
| SAP30 | F8 |
| SAP30 | FLVCR2 |
| SAP30 | ACP6 |
| SAP30 | NPEPL1 |
| SAP30 | RRM2B |
| SAP30 | VAV3 |
| SAP30 | CREB1 |
| SAP30 | GAPT |
| SAP30 | RNASE4 |
| SAP30 | PHACTR1 |
| SAP30 | FLT3 |
| SAP30 | DLGAP1-AS2 |
| SAP30 | RP1-193H18.2 |
| SAP30 | LINC00282 |
| SAP30 | DKFZP434L187 |
| SAP30 | RP1-30M3.5 |
| SAP30 | RP11-111K18.2 |
| SAP30 | KB-431C1.4 |
| SAP30 | SNRK-AS1 |
| SLK | FAM120AOS |
| SLK | GIT2 |
| SLK | CUL4B |
| SLK | VCPIP1 |
| SLK | CRIPT |
| SLK | PIK3C3 |
| SLK | ST6GALNAC3 |
| SLK | DHX40 |
| SLK | TANC2 |
| SLK | IDH1 |
| SLK | PAPOLA |
| SLK | FEZ2 |
| SLK | EAPP |
| SLK | HOOK3 |
| SLK | SOAT1 |
| SLK | MED31 |
| SLK | SENP1 |
| SLK | NSMAF |
| SLK | PPP5C |
| SLK | CREBRF |
| SLK | VPS41 |
| SLK | PSMD12 |
| SLK | SAP30L |
| SLK | STAM2 |
| SLK | PSMC3 |
| SLK | SCARB2 |
| SLK | IPCEF1 |
| SLK | TMEM260 |
| SLK | TMED8 |
| SLK | LPCAT2 |
| SLK | WSB1 |
| SLK | SCCPDH |
| SLK | BAZ2B |
| SLK | GORASP2 |
| SLK | EIF2AK2 |
| SLK | TBCC |
| SLK | GNAI3 |
| SLK | NEURL1 |
| SLK | INTS6 |
| SLK | TFG |
| SLK | MEF2A |
| SLK | ASCC1 |
| SLK | GOLPH3L |
| SLK | CLIC3 |
| SLK | SCYL2 |
| SLK | SLX4IP |
| SLK | NBN |
| SLK | PLD4 |
| SLK | BIRC2 |
| SLK | GNB4 |
| SLK | SELT |
| SLK | DCUN1D1 |
| SLK | ASPH |
| SLK | BROX |
| SLK | ARRDC4 |
| SLK | DNAJA2 |
| SLK | FAM199X |
| SLK | ADRBK2 |
| SLK | LRTOMT |
| SLK | CMTM4 |
| SLK | ACP6 |
| SLK | BRCA1 |
| SLK | NPEPL1 |
| SLK | PAPSS1 |
| SLK | DDIT3 |
| SLK | RRM2B |
| SLK | PHTF1 |
| SLK | TLR10 |
| SLK | PRMT5 |
| SLK | MTL5 |
| SLK | CREB1 |
| SLK | GAPT |
| SLK | ATP10D |
| SLK | DLGAP1-AS2 |
| SLK | RP1-193H18.2 |
| SLK | LINC00282 |
| SLK | RP1-30M3.5 |
| SLK | RP11-111K18.2 |
| SLK | RP11-399O19.9 |
| SLK | KB-431C1.4 |
| SLK | SNRK-AS1 |
| SLK | RP11-476D10.1 |
| FAM120AOS | GIT2 |
| FAM120AOS | CUL4B |
| FAM120AOS | VCPIP1 |
| FAM120AOS | CRIPT |
| FAM120AOS | PIK3C3 |
| FAM120AOS | TNFRSF25 |
| FAM120AOS | ID3 |
| FAM120AOS | ST6GALNAC3 |
| FAM120AOS | DHX40 |
| FAM120AOS | KDM3B |
| FAM120AOS | SLC12A6 |
| FAM120AOS | TANC2 |
| FAM120AOS | IDH1 |
| FAM120AOS | PAPOLA |
| FAM120AOS | ATXN2L |
| FAM120AOS | FEZ2 |
| FAM120AOS | EAPP |
| FAM120AOS | HOOK3 |
| FAM120AOS | CD163 |
| FAM120AOS | SOAT1 |
| FAM120AOS | MED31 |
| FAM120AOS | SENP1 |
| FAM120AOS | NSMAF |
| FAM120AOS | PPP5C |
| FAM120AOS | ATN1 |
| FAM120AOS | CREBRF |
| FAM120AOS | VPS41 |
| FAM120AOS | PSMD12 |
| FAM120AOS | KIAA2013 |
| FAM120AOS | SAP30L |
| FAM120AOS | NAGA |
| FAM120AOS | CLEC5A |
| FAM120AOS | TSHZ3 |
| FAM120AOS | ITFG2 |
| FAM120AOS | STAM2 |
| FAM120AOS | SPRED2 |
| FAM120AOS | IPCEF1 |
| FAM120AOS | TMEM260 |
| FAM120AOS | TMED8 |
| FAM120AOS | LPCAT2 |
| FAM120AOS | WSB1 |
| FAM120AOS | SCCPDH |
| FAM120AOS | BAZ2B |
| FAM120AOS | GORASP2 |
| FAM120AOS | EIF2AK2 |
| FAM120AOS | TBCC |
| FAM120AOS | GNAI3 |
| FAM120AOS | MPPED2 |
| FAM120AOS | NEURL1 |
| FAM120AOS | SFN |
| FAM120AOS | INTS6 |
| FAM120AOS | TFG |
| FAM120AOS | MEF2A |
| FAM120AOS | ASCC1 |
| FAM120AOS | UBE2E3 |
| FAM120AOS | AREL1 |
| FAM120AOS | SCYL2 |
| FAM120AOS | SLX4IP |
| FAM120AOS | NBN |
| FAM120AOS | PLD4 |
| FAM120AOS | C8orf88 |
| FAM120AOS | GNB4 |
| FAM120AOS | SELT |
| FAM120AOS | DCUN1D1 |
| FAM120AOS | ASPH |
| FAM120AOS | PLIN2 |
| FAM120AOS | GPER1 |
| FAM120AOS | BROX |
| FAM120AOS | SUMF1 |
| FAM120AOS | ARRDC4 |
| FAM120AOS | DNAJA2 |
| FAM120AOS | CYFIP1 |
| FAM120AOS | TPM2 |
| FAM120AOS | ARF3 |
| FAM120AOS | TTC28 |
| FAM120AOS | SCPEP1 |
| FAM120AOS | CMTM4 |
| FAM120AOS | F8 |
| FAM120AOS | FLVCR2 |
| FAM120AOS | MS4A4A |
| FAM120AOS | ACP6 |
| FAM120AOS | BRCA1 |
| FAM120AOS | NPEPL1 |
| FAM120AOS | PAPSS1 |
| FAM120AOS | DDIT3 |
| FAM120AOS | RRM2B |
| FAM120AOS | PHTF1 |
| FAM120AOS | VAV3 |
| FAM120AOS | ATP10D |
| FAM120AOS | PHACTR1 |
| FAM120AOS | FLT3 |
| FAM120AOS | DLGAP1-AS2 |
| FAM120AOS | RP1-193H18.2 |
| FAM120AOS | LINC00282 |
| FAM120AOS | DKFZP434L187 |
| FAM120AOS | RP1-30M3.5 |
| FAM120AOS | RP11-111K18.2 |
| FAM120AOS | RP11-2E11.9 |
| FAM120AOS | RP11-399O19.9 |
| FAM120AOS | KB-431C1.4 |
| FAM120AOS | SNRK-AS1 |
| FAM120AOS | RP11-476D10.1 |
| GIT2 | CUL4B |
| GIT2 | VCPIP1 |
| GIT2 | CRIPT |
| GIT2 | PIK3C3 |
| GIT2 | TNFRSF25 |
| GIT2 | ID3 |
| GIT2 | ST6GALNAC3 |
| GIT2 | DHX40 |
| GIT2 | KDM3B |
| GIT2 | SLC12A6 |
| GIT2 | TANC2 |
| GIT2 | IDH1 |
| GIT2 | PAPOLA |
| GIT2 | ATXN2L |
| GIT2 | FEZ2 |
| GIT2 | EAPP |
| GIT2 | HOOK3 |
| GIT2 | CD163 |
| GIT2 | SOAT1 |
| GIT2 | MED31 |
| GIT2 | SENP1 |
| GIT2 | NSMAF |
| GIT2 | PPP5C |
| GIT2 | ATN1 |
| GIT2 | CREBRF |
| GIT2 | VPS41 |
| GIT2 | PSMD12 |
| GIT2 | SHC4 |
| GIT2 | KIAA2013 |
| GIT2 | SAP30L |
| GIT2 | NAGA |
| GIT2 | CLEC5A |
| GIT2 | TSHZ3 |
| GIT2 | ITFG2 |
| GIT2 | STAM2 |
| GIT2 | SPRED2 |
| GIT2 | PSMC3 |
| GIT2 | SCARB2 |
| GIT2 | IPCEF1 |
| GIT2 | TMEM260 |
| GIT2 | TMED8 |
| GIT2 | LPCAT2 |
| GIT2 | WSB1 |
| GIT2 | SCCPDH |
| GIT2 | C7orf73 |
| GIT2 | BAZ2B |
| GIT2 | NDST3 |
| GIT2 | GORASP2 |
| GIT2 | EIF2AK2 |
| GIT2 | TBCC |
| GIT2 | GNAI3 |
| GIT2 | MPPED2 |
| GIT2 | NEURL1 |
| GIT2 | SFN |
| GIT2 | INTS6 |
| GIT2 | TFG |
| GIT2 | MEF2A |
| GIT2 | ASCC1 |
| GIT2 | PRMT1 |
| GIT2 | GOLPH3L |
| GIT2 | CLIC3 |
| GIT2 | UBE2E3 |
| GIT2 | MSX1 |
| GIT2 | AREL1 |
| GIT2 | S1PR3 |
| GIT2 | SCYL2 |
| GIT2 | SLX4IP |
| GIT2 | RNF165 |
| GIT2 | NBN |
| GIT2 | PLD4 |
| GIT2 | OGFOD3 |
| GIT2 | CYBB |
| GIT2 | C8orf88 |
| GIT2 | AK2 |
| GIT2 | BIRC2 |
| GIT2 | GNB4 |
| GIT2 | SELT |
| GIT2 | DCUN1D1 |
| GIT2 | ASPH |
| GIT2 | SETD7 |
| GIT2 | ARL15 |
| GIT2 | MMP19 |
| GIT2 | CEP164 |
| GIT2 | PLIN2 |
| GIT2 | GPER1 |
| GIT2 | BROX |
| GIT2 | C5orf45 |
| GIT2 | SUMF1 |
| GIT2 | FBN2 |
| GIT2 | ARRDC4 |
| GIT2 | DNAJA2 |
| GIT2 | ZNF287 |
| GIT2 | CYFIP1 |
| GIT2 | TPM2 |
| GIT2 | BCAT1 |
| GIT2 | FAM199X |
| GIT2 | ADRBK2 |
| GIT2 | LRTOMT |
| GIT2 | GOLPH3 |
| GIT2 | ARF3 |
| GIT2 | TTC28 |
| GIT2 | SCPEP1 |
| GIT2 | CMTM4 |
| GIT2 | ALKBH2 |
| GIT2 | F8 |
| GIT2 | FLVCR2 |
| GIT2 | MS4A4A |
| GIT2 | CHMP7 |
| GIT2 | ACP6 |
| GIT2 | BRCA1 |
| GIT2 | TRAPPC6A |
| GIT2 | NPEPL1 |
| GIT2 | PAPSS1 |
| GIT2 | DDIT3 |
| GIT2 | NOS3 |
| GIT2 | FAM110B |
| GIT2 | RRM2B |
| GIT2 | PHTF1 |
| GIT2 | CLIC4 |
| GIT2 | SLC1A3 |
| GIT2 | TLR10 |
| GIT2 | DSE |
| GIT2 | SGMS2 |
| GIT2 | PRMT5 |
| GIT2 | MTL5 |
| GIT2 | KNOP1 |
| GIT2 | VAV3 |
| GIT2 | ERI3 |
| GIT2 | CREB1 |
| GIT2 | GAPT |
| GIT2 | ATP10D |
| GIT2 | CC2D2B |
| GIT2 | ENOX1 |
| GIT2 | MPZL2 |
| GIT2 | PHACTR1 |
| GIT2 | FLT3 |
| GIT2 | NKAP |
| GIT2 | CD27 |
| GIT2 | DLGAP1-AS2 |
| GIT2 | RP1-193H18.2 |
| GIT2 | LINC00282 |
| GIT2 | DKFZP434L187 |
| GIT2 | RP1-30M3.5 |
| GIT2 | RP11-111K18.2 |
| GIT2 | RP11-2E11.9 |
| GIT2 | RP11-399O19.9 |
| GIT2 | KB-431C1.4 |
| GIT2 | RP11-1024P17.1 |
| GIT2 | SNRK-AS1 |
| GIT2 | AC005785.2 |
| GIT2 | RP11-476D10.1 |
| CUL4B | VCPIP1 |
| CUL4B | CRIPT |
| CUL4B | PIK3C3 |
| CUL4B | TNFRSF25 |
| CUL4B | ID3 |
| CUL4B | ST6GALNAC3 |
| CUL4B | DHX40 |
| CUL4B | KDM3B |
| CUL4B | SLC12A6 |
| CUL4B | TANC2 |
| CUL4B | IDH1 |
| CUL4B | PAPOLA |
| CUL4B | ATXN2L |
| CUL4B | FEZ2 |
| CUL4B | EAPP |
| CUL4B | HOOK3 |
| CUL4B | CD163 |
| CUL4B | SOAT1 |
| CUL4B | MED31 |
| CUL4B | SENP1 |
| CUL4B | NSMAF |
| CUL4B | PPP5C |
| CUL4B | ATN1 |
| CUL4B | CREBRF |
| CUL4B | VPS41 |
| CUL4B | PSMD12 |
| CUL4B | SHC4 |
| CUL4B | KIAA2013 |
| CUL4B | SAP30L |
| CUL4B | NAGA |
| CUL4B | CLEC5A |
| CUL4B | TSHZ3 |
| CUL4B | ITFG2 |
| CUL4B | STAM2 |
| CUL4B | SPRED2 |
| CUL4B | PSMC3 |
| CUL4B | SCARB2 |
| CUL4B | IPCEF1 |
| CUL4B | TMEM260 |
| CUL4B | TMED8 |
| CUL4B | LPCAT2 |
| CUL4B | WSB1 |
| CUL4B | SCCPDH |
| CUL4B | C7orf73 |
| CUL4B | BAZ2B |
| CUL4B | NDST3 |
| CUL4B | GORASP2 |
| CUL4B | EIF2AK2 |
| CUL4B | TBCC |
| CUL4B | GNAI3 |
| CUL4B | MPPED2 |
| CUL4B | NEURL1 |
| CUL4B | SFN |
| CUL4B | INTS6 |
| CUL4B | TFG |
| CUL4B | MEF2A |
| CUL4B | ASCC1 |
| CUL4B | PRMT1 |
| CUL4B | GOLPH3L |
| CUL4B | CLIC3 |
| CUL4B | UBE2E3 |
| CUL4B | MSX1 |
| CUL4B | AREL1 |
| CUL4B | S1PR3 |
| CUL4B | SCYL2 |
| CUL4B | SIX3 |
| CUL4B | SLX4IP |
| CUL4B | RNF165 |
| CUL4B | NBN |
| CUL4B | PLD4 |
| CUL4B | OGFOD3 |
| CUL4B | CYBB |
| CUL4B | C8orf88 |
| CUL4B | AK2 |
| CUL4B | BIRC2 |
| CUL4B | GNB4 |
| CUL4B | SELT |
| CUL4B | DCUN1D1 |
| CUL4B | ASPH |
| CUL4B | SETD7 |
| CUL4B | ARL15 |
| CUL4B | MMP19 |
| CUL4B | CEP164 |
| CUL4B | PLIN2 |
| CUL4B | GPER1 |
| CUL4B | BROX |
| CUL4B | C5orf45 |
| CUL4B | SUMF1 |
| CUL4B | FBN2 |
| CUL4B | ARRDC4 |
| CUL4B | DNAJA2 |
| CUL4B | DMRT2 |
| CUL4B | ZNF287 |
| CUL4B | CYFIP1 |
| CUL4B | TPM2 |
| CUL4B | BCAT1 |
| CUL4B | FAM199X |
| CUL4B | ADRBK2 |
| CUL4B | PNPLA3 |
| CUL4B | LRTOMT |
| CUL4B | GOLPH3 |
| CUL4B | ARF3 |
| CUL4B | TTC28 |
| CUL4B | SCPEP1 |
| CUL4B | CMTM4 |
| CUL4B | ALKBH2 |
| CUL4B | F8 |
| CUL4B | FLVCR2 |
| CUL4B | PIEZO1 |
| CUL4B | MS4A4A |
| CUL4B | COL5A3 |
| CUL4B | CHMP7 |
| CUL4B | ACP6 |
| CUL4B | BRCA1 |
| CUL4B | TRAPPC6A |
| CUL4B | NPEPL1 |
| CUL4B | PAPSS1 |
| CUL4B | PDE1C |
| CUL4B | DDIT3 |
| CUL4B | NOS3 |
| CUL4B | MLC1 |
| CUL4B | LDLRAD3 |
| CUL4B | FAM110B |
| CUL4B | GOLGA6L2 |
| CUL4B | RRM2B |
| CUL4B | PHTF1 |
| CUL4B | CLIC4 |
| CUL4B | SLC1A3 |
| CUL4B | TLR10 |
| CUL4B | DSE |
| CUL4B | GALNT15 |
| CUL4B | SGMS2 |
| CUL4B | PRMT5 |
| CUL4B | MTL5 |
| CUL4B | KNOP1 |
| CUL4B | VAV3 |
| CUL4B | ERI3 |
| CUL4B | CREB1 |
| CUL4B | GAPT |
| CUL4B | ATP10D |
| CUL4B | LIN54 |
| CUL4B | CC2D2B |
| CUL4B | ENOX1 |
| CUL4B | TACR1 |
| CUL4B | MATK |
| CUL4B | MPZL2 |
| CUL4B | SALL3 |
| CUL4B | RNASE4 |
| CUL4B | PHACTR1 |
| CUL4B | NETO2 |
| CUL4B | FLT3 |
| CUL4B | NKAP |
| CUL4B | CD27 |
| CUL4B | PAPSS2 |
| CUL4B | SEMG1 |
| CUL4B | IFIT5 |
| CUL4B | ZNF608 |
| CUL4B | DLGAP1-AS2 |
| CUL4B | RP1-193H18.2 |
| CUL4B | LINC00550 |
| CUL4B | LINC00282 |
| CUL4B | DKFZP434L187 |
| CUL4B | RP1-30M3.5 |
| CUL4B | RP11-111K18.2 |
| CUL4B | RP11-2E11.9 |
| CUL4B | RP11-399O19.9 |
| CUL4B | KB-431C1.4 |
| CUL4B | RP11-1024P17.1 |
| CUL4B | SNRK-AS1 |
| CUL4B | AC005785.2 |
| CUL4B | RP11-476D10.1 |
| VCPIP1 | CRIPT |
| VCPIP1 | PIK3C3 |
| VCPIP1 | ID3 |
| VCPIP1 | ST6GALNAC3 |
| VCPIP1 | DHX40 |
| VCPIP1 | KDM3B |
| VCPIP1 | SLC12A6 |
| VCPIP1 | TANC2 |
| VCPIP1 | IDH1 |
| VCPIP1 | PAPOLA |
| VCPIP1 | ATXN2L |
| VCPIP1 | FEZ2 |
| VCPIP1 | EAPP |
| VCPIP1 | HOOK3 |
| VCPIP1 | CD163 |
| VCPIP1 | SOAT1 |
| VCPIP1 | MED31 |
| VCPIP1 | SENP1 |
| VCPIP1 | NSMAF |
| VCPIP1 | PPP5C |
| VCPIP1 | ATN1 |
| VCPIP1 | CREBRF |
| VCPIP1 | VPS41 |
| VCPIP1 | PSMD12 |
| VCPIP1 | KIAA2013 |
| VCPIP1 | SAP30L |
| VCPIP1 | NAGA |
| VCPIP1 | CLEC5A |
| VCPIP1 | TSHZ3 |
| VCPIP1 | ITFG2 |
| VCPIP1 | STAM2 |
| VCPIP1 | SCARB2 |
| VCPIP1 | IPCEF1 |
| VCPIP1 | TMEM260 |
| VCPIP1 | TMED8 |
| VCPIP1 | LPCAT2 |
| VCPIP1 | WSB1 |
| VCPIP1 | SCCPDH |
| VCPIP1 | BAZ2B |
| VCPIP1 | EIF2AK2 |
| VCPIP1 | TBCC |
| VCPIP1 | GNAI3 |
| VCPIP1 | MPPED2 |
| VCPIP1 | NEURL1 |
| VCPIP1 | SFN |
| VCPIP1 | INTS6 |
| VCPIP1 | TFG |
| VCPIP1 | MEF2A |
| VCPIP1 | ASCC1 |
| VCPIP1 | PRMT1 |
| VCPIP1 | GOLPH3L |
| VCPIP1 | CLIC3 |
| VCPIP1 | UBE2E3 |
| VCPIP1 | AREL1 |
| VCPIP1 | S1PR3 |
| VCPIP1 | SCYL2 |
| VCPIP1 | SLX4IP |
| VCPIP1 | NBN |
| VCPIP1 | PLD4 |
| VCPIP1 | CYBB |
| VCPIP1 | BIRC2 |
| VCPIP1 | GNB4 |
| VCPIP1 | SELT |
| VCPIP1 | DCUN1D1 |
| VCPIP1 | ASPH |
| VCPIP1 | SETD7 |
| VCPIP1 | MMP19 |
| VCPIP1 | CEP164 |
| VCPIP1 | PLIN2 |
| VCPIP1 | BROX |
| VCPIP1 | SUMF1 |
| VCPIP1 | ARRDC4 |
| VCPIP1 | DNAJA2 |
| VCPIP1 | CYFIP1 |
| VCPIP1 | TPM2 |
| VCPIP1 | TTC28 |
| VCPIP1 | SCPEP1 |
| VCPIP1 | CMTM4 |
| VCPIP1 | ALKBH2 |
| VCPIP1 | F8 |
| VCPIP1 | FLVCR2 |
| VCPIP1 | MS4A4A |
| VCPIP1 | ACP6 |
| VCPIP1 | BRCA1 |
| VCPIP1 | TRAPPC6A |
| VCPIP1 | NPEPL1 |
| VCPIP1 | PAPSS1 |
| VCPIP1 | DDIT3 |
| VCPIP1 | RRM2B |
| VCPIP1 | PHTF1 |
| VCPIP1 | PRMT5 |
| VCPIP1 | MTL5 |
| VCPIP1 | VAV3 |
| VCPIP1 | GAPT |
| VCPIP1 | ATP10D |
| VCPIP1 | IFIT5 |
| VCPIP1 | DLGAP1-AS2 |
| VCPIP1 | RP1-193H18.2 |
| VCPIP1 | LINC00282 |
| VCPIP1 | RP1-30M3.5 |
| VCPIP1 | RP11-111K18.2 |
| VCPIP1 | RP11-2E11.9 |
| VCPIP1 | RP11-399O19.9 |
| VCPIP1 | KB-431C1.4 |
| VCPIP1 | SNRK-AS1 |
| VCPIP1 | RP11-476D10.1 |
| CRIPT | PIK3C3 |
| CRIPT | ST6GALNAC3 |
| CRIPT | DHX40 |
| CRIPT | KDM3B |
| CRIPT | SLC12A6 |
| CRIPT | TANC2 |
| CRIPT | IDH1 |
| CRIPT | PAPOLA |
| CRIPT | ATXN2L |
| CRIPT | FEZ2 |
| CRIPT | EAPP |
| CRIPT | HOOK3 |
| CRIPT | SOAT1 |
| CRIPT | MED31 |
| CRIPT | SENP1 |
| CRIPT | NSMAF |
| CRIPT | PPP5C |
| CRIPT | ATN1 |
| CRIPT | CREBRF |
| CRIPT | VPS41 |
| CRIPT | PSMD12 |
| CRIPT | SHC4 |
| CRIPT | KIAA2013 |
| CRIPT | SAP30L |
| CRIPT | ITFG2 |
| CRIPT | STAM2 |
| CRIPT | PSMC3 |
| CRIPT | SCARB2 |
| CRIPT | TMEM260 |
| CRIPT | TMED8 |
| CRIPT | LPCAT2 |
| CRIPT | SCCPDH |
| CRIPT | BAZ2B |
| CRIPT | EIF2AK2 |
| CRIPT | TBCC |
| CRIPT | GNAI3 |
| CRIPT | NEURL1 |
| CRIPT | INTS6 |
| CRIPT | TFG |
| CRIPT | MEF2A |
| CRIPT | ASCC1 |
| CRIPT | PRMT1 |
| CRIPT | CLIC3 |
| CRIPT | UBE2E3 |
| CRIPT | MSX1 |
| CRIPT | AREL1 |
| CRIPT | SCYL2 |
| CRIPT | SLX4IP |
| CRIPT | NBN |
| CRIPT | OGFOD3 |
| CRIPT | AK2 |
| CRIPT | BIRC2 |
| CRIPT | GNB4 |
| CRIPT | DCUN1D1 |
| CRIPT | ASPH |
| CRIPT | BROX |
| CRIPT | ARRDC4 |
| CRIPT | DNAJA2 |
| CRIPT | ADRBK2 |
| CRIPT | GOLPH3 |
| CRIPT | TTC28 |
| CRIPT | CMTM4 |
| CRIPT | F8 |
| CRIPT | FLVCR2 |
| CRIPT | ACP6 |
| CRIPT | BRCA1 |
| CRIPT | PAPSS1 |
| CRIPT | DDIT3 |
| CRIPT | RRM2B |
| CRIPT | MTL5 |
| CRIPT | CREB1 |
| CRIPT | GAPT |
| CRIPT | ATP10D |
| CRIPT | DLGAP1-AS2 |
| CRIPT | RP1-193H18.2 |
| CRIPT | LINC00282 |
| CRIPT | RP1-30M3.5 |
| CRIPT | RP11-111K18.2 |
| CRIPT | KB-431C1.4 |
| CRIPT | SNRK-AS1 |
| PIK3C3 | TNFRSF25 |
| PIK3C3 | ID3 |
| PIK3C3 | ST6GALNAC3 |
| PIK3C3 | DHX40 |
| PIK3C3 | KDM3B |
| PIK3C3 | SLC12A6 |
| PIK3C3 | TANC2 |
| PIK3C3 | IDH1 |
| PIK3C3 | PAPOLA |
| PIK3C3 | ATXN2L |
| PIK3C3 | FEZ2 |
| PIK3C3 | EAPP |
| PIK3C3 | HOOK3 |
| PIK3C3 | CD163 |
| PIK3C3 | SOAT1 |
| PIK3C3 | MED31 |
| PIK3C3 | SENP1 |
| PIK3C3 | NSMAF |
| PIK3C3 | PPP5C |
| PIK3C3 | ATN1 |
| PIK3C3 | CREBRF |
| PIK3C3 | VPS41 |
| PIK3C3 | PSMD12 |
| PIK3C3 | KIAA2013 |
| PIK3C3 | SAP30L |
| PIK3C3 | NAGA |
| PIK3C3 | CLEC5A |
| PIK3C3 | TSHZ3 |
| PIK3C3 | ITFG2 |
| PIK3C3 | STAM2 |
| PIK3C3 | SPRED2 |
| PIK3C3 | PSMC3 |
| PIK3C3 | SCARB2 |
| PIK3C3 | IPCEF1 |
| PIK3C3 | TMEM260 |
| PIK3C3 | TMED8 |
| PIK3C3 | LPCAT2 |
| PIK3C3 | WSB1 |
| PIK3C3 | SCCPDH |
| PIK3C3 | C7orf73 |
| PIK3C3 | BAZ2B |
| PIK3C3 | NDST3 |
| PIK3C3 | GORASP2 |
| PIK3C3 | EIF2AK2 |
| PIK3C3 | TBCC |
| PIK3C3 | GNAI3 |
| PIK3C3 | MPPED2 |
| PIK3C3 | NEURL1 |
| PIK3C3 | SFN |
| PIK3C3 | INTS6 |
| PIK3C3 | TFG |
| PIK3C3 | MEF2A |
| PIK3C3 | ASCC1 |
| PIK3C3 | PRMT1 |
| PIK3C3 | GOLPH3L |
| PIK3C3 | CLIC3 |
| PIK3C3 | UBE2E3 |
| PIK3C3 | MSX1 |
| PIK3C3 | AREL1 |
| PIK3C3 | S1PR3 |
| PIK3C3 | SCYL2 |
| PIK3C3 | SIX3 |
| PIK3C3 | SLX4IP |
| PIK3C3 | RNF165 |
| PIK3C3 | NBN |
| PIK3C3 | PLD4 |
| PIK3C3 | OGFOD3 |
| PIK3C3 | CYBB |
| PIK3C3 | C8orf88 |
| PIK3C3 | AK2 |
| PIK3C3 | BIRC2 |
| PIK3C3 | GNB4 |
| PIK3C3 | SELT |
| PIK3C3 | DCUN1D1 |
| PIK3C3 | ASPH |
| PIK3C3 | SETD7 |
| PIK3C3 | ARL15 |
| PIK3C3 | MMP19 |
| PIK3C3 | CEP164 |
| PIK3C3 | PLIN2 |
| PIK3C3 | GPER1 |
| PIK3C3 | BROX |
| PIK3C3 | SUMF1 |
| PIK3C3 | FBN2 |
| PIK3C3 | ARRDC4 |
| PIK3C3 | DNAJA2 |
| PIK3C3 | DMRT2 |
| PIK3C3 | ZNF287 |
| PIK3C3 | CYFIP1 |
| PIK3C3 | TPM2 |
| PIK3C3 | BCAT1 |
| PIK3C3 | FAM199X |
| PIK3C3 | ADRBK2 |
| PIK3C3 | LRTOMT |
| PIK3C3 | GOLPH3 |
| PIK3C3 | ARF3 |
| PIK3C3 | TTC28 |
| PIK3C3 | SCPEP1 |
| PIK3C3 | CMTM4 |
| PIK3C3 | ZNF483 |
| PIK3C3 | ALKBH2 |
| PIK3C3 | F8 |
| PIK3C3 | FLVCR2 |
| PIK3C3 | MS4A4A |
| PIK3C3 | CHMP7 |
| PIK3C3 | ACP6 |
| PIK3C3 | BRCA1 |
| PIK3C3 | TRAPPC6A |
| PIK3C3 | NPEPL1 |
| PIK3C3 | PAPSS1 |
| PIK3C3 | DDIT3 |
| PIK3C3 | FAM110B |
| PIK3C3 | RRM2B |
| PIK3C3 | PHTF1 |
| PIK3C3 | CLIC4 |
| PIK3C3 | SLC1A3 |
| PIK3C3 | TLR10 |
| PIK3C3 | DSE |
| PIK3C3 | PRMT5 |
| PIK3C3 | MTL5 |
| PIK3C3 | KNOP1 |
| PIK3C3 | VAV3 |
| PIK3C3 | ERI3 |
| PIK3C3 | CREB1 |
| PIK3C3 | GAPT |
| PIK3C3 | ATP10D |
| PIK3C3 | CC2D2B |
| PIK3C3 | ENOX1 |
| PIK3C3 | TACR1 |
| PIK3C3 | MATK |
| PIK3C3 | MPZL2 |
| PIK3C3 | RNASE4 |
| PIK3C3 | PHACTR1 |
| PIK3C3 | NETO2 |
| PIK3C3 | FLT3 |
| PIK3C3 | NKAP |
| PIK3C3 | DLGAP1-AS2 |
| PIK3C3 | RP1-193H18.2 |
| PIK3C3 | LINC00282 |
| PIK3C3 | DKFZP434L187 |
| PIK3C3 | RP1-30M3.5 |
| PIK3C3 | RP11-111K18.2 |
| PIK3C3 | RP11-2E11.9 |
| PIK3C3 | RP11-399O19.9 |
| PIK3C3 | KB-431C1.4 |
| PIK3C3 | RP11-1024P17.1 |
| PIK3C3 | SNRK-AS1 |
| PIK3C3 | AC005785.2 |
| PIK3C3 | RP11-476D10.1 |
| TNFRSF25 | ID3 |
| TNFRSF25 | DHX40 |
| TNFRSF25 | SLC12A6 |
| TNFRSF25 | TANC2 |
| TNFRSF25 | IDH1 |
| TNFRSF25 | FEZ2 |
| TNFRSF25 | CD163 |
| TNFRSF25 | SOAT1 |
| TNFRSF25 | NSMAF |
| TNFRSF25 | KIAA2013 |
| TNFRSF25 | NAGA |
| TNFRSF25 | TSHZ3 |
| TNFRSF25 | ITFG2 |
| TNFRSF25 | SPRED2 |
| TNFRSF25 | LPCAT2 |
| TNFRSF25 | GNAI3 |
| TNFRSF25 | PRMT1 |
| TNFRSF25 | AREL1 |
| TNFRSF25 | MMP19 |
| TNFRSF25 | C5orf45 |
| TNFRSF25 | ARF3 |
| TNFRSF25 | CHMP7 |
| TNFRSF25 | TRAPPC6A |
| TNFRSF25 | EPB41L3 |
| TNFRSF25 | NOS3 |
| TNFRSF25 | ERI3 |
| TNFRSF25 | RLTPR |
| TNFRSF25 | CD27 |
| TNFRSF25 | DLGAP1-AS2 |
| TNFRSF25 | RP1-193H18.2 |
| ID3 | DHX40 |
| ID3 | KDM3B |
| ID3 | SLC12A6 |
| ID3 | TANC2 |
| ID3 | PAPOLA |
| ID3 | FEZ2 |
| ID3 | SOAT1 |
| ID3 | NSMAF |
| ID3 | VPS41 |
| ID3 | KIAA2013 |
| ID3 | NAGA |
| ID3 | LPCAT2 |
| ID3 | BAZ2B |
| ID3 | GNAI3 |
| ID3 | SFN |
| ID3 | PRMT1 |
| ID3 | MMP19 |
| ID3 | CHMP7 |
| ID3 | NOS3 |
| ID3 | RLTPR |
| ID3 | CD27 |
| ID3 | DLGAP1-AS2 |
| ID3 | RP1-193H18.2 |
| ST6GALNAC3 | DHX40 |
| ST6GALNAC3 | SLC12A6 |
| ST6GALNAC3 | TANC2 |
| ST6GALNAC3 | IDH1 |
| ST6GALNAC3 | PAPOLA |
| ST6GALNAC3 | ATXN2L |
| ST6GALNAC3 | FEZ2 |
| ST6GALNAC3 | EAPP |
| ST6GALNAC3 | HOOK3 |
| ST6GALNAC3 | SOAT1 |
| ST6GALNAC3 | MED31 |
| ST6GALNAC3 | SENP1 |
| ST6GALNAC3 | NSMAF |
| ST6GALNAC3 | PPP5C |
| ST6GALNAC3 | ATN1 |
| ST6GALNAC3 | CREBRF |
| ST6GALNAC3 | VPS41 |
| ST6GALNAC3 | PSMD12 |
| ST6GALNAC3 | KIAA2013 |
| ST6GALNAC3 | ITFG2 |
| ST6GALNAC3 | STAM2 |
| ST6GALNAC3 | TMEM260 |
| ST6GALNAC3 | TMED8 |
| ST6GALNAC3 | LPCAT2 |
| ST6GALNAC3 | SCCPDH |
| ST6GALNAC3 | BAZ2B |
| ST6GALNAC3 | EIF2AK2 |
| ST6GALNAC3 | TBCC |
| ST6GALNAC3 | GNAI3 |
| ST6GALNAC3 | NEURL1 |
| ST6GALNAC3 | TFG |
| ST6GALNAC3 | MEF2A |
| ST6GALNAC3 | UBE2E3 |
| ST6GALNAC3 | SCYL2 |
| ST6GALNAC3 | NBN |
| ST6GALNAC3 | PLD4 |
| ST6GALNAC3 | BIRC2 |
| ST6GALNAC3 | GNB4 |
| ST6GALNAC3 | DCUN1D1 |
| ST6GALNAC3 | BROX |
| ST6GALNAC3 | DNAJA2 |
| ST6GALNAC3 | GOLPH3 |
| ST6GALNAC3 | TTC28 |
| ST6GALNAC3 | CMTM4 |
| ST6GALNAC3 | DDIT3 |
| ST6GALNAC3 | RRM2B |
| ST6GALNAC3 | DLGAP1-AS2 |
| ST6GALNAC3 | RP1-193H18.2 |
| ST6GALNAC3 | LINC00282 |
| ST6GALNAC3 | RP1-30M3.5 |
| ST6GALNAC3 | RP11-2E11.9 |
| ST6GALNAC3 | KB-431C1.4 |
| DHX40 | KDM3B |
| DHX40 | SLC12A6 |
| DHX40 | TANC2 |
| DHX40 | IDH1 |
| DHX40 | PAPOLA |
| DHX40 | ATXN2L |
| DHX40 | FEZ2 |
| DHX40 | EAPP |
| DHX40 | HOOK3 |
| DHX40 | CD163 |
| DHX40 | SOAT1 |
| DHX40 | MED31 |
| DHX40 | SENP1 |
| DHX40 | NSMAF |
| DHX40 | PPP5C |
| DHX40 | ATN1 |
| DHX40 | CREBRF |
| DHX40 | VPS41 |
| DHX40 | PSMD12 |
| DHX40 | KIAA2013 |
| DHX40 | SAP30L |
| DHX40 | NAGA |
| DHX40 | CLEC5A |
| DHX40 | TSHZ3 |
| DHX40 | ITFG2 |
| DHX40 | STAM2 |
| DHX40 | SPRED2 |
| DHX40 | PSMC3 |
| DHX40 | SCARB2 |
| DHX40 | IPCEF1 |
| DHX40 | TMEM260 |
| DHX40 | TMED8 |
| DHX40 | LPCAT2 |
| DHX40 | WSB1 |
| DHX40 | SCCPDH |
| DHX40 | C7orf73 |
| DHX40 | BAZ2B |
| DHX40 | EIF2AK2 |
| DHX40 | TBCC |
| DHX40 | GNAI3 |
| DHX40 | MPPED2 |
| DHX40 | NEURL1 |
| DHX40 | SFN |
| DHX40 | INTS6 |
| DHX40 | TFG |
| DHX40 | MEF2A |
| DHX40 | PRMT1 |
| DHX40 | GOLPH3L |
| DHX40 | CLIC3 |
| DHX40 | UBE2E3 |
| DHX40 | MSX1 |
| DHX40 | AREL1 |
| DHX40 | S1PR3 |
| DHX40 | SCYL2 |
| DHX40 | SLX4IP |
| DHX40 | RNF165 |
| DHX40 | NBN |
| DHX40 | PLD4 |
| DHX40 | OGFOD3 |
| DHX40 | CYBB |
| DHX40 | AK2 |
| DHX40 | BIRC2 |
| DHX40 | GNB4 |
| DHX40 | SELT |
| DHX40 | DCUN1D1 |
| DHX40 | ASPH |
| DHX40 | SETD7 |
| DHX40 | ARL15 |
| DHX40 | MMP19 |
| DHX40 | CEP164 |
| DHX40 | GPER1 |
| DHX40 | BROX |
| DHX40 | C5orf45 |
| DHX40 | SUMF1 |
| DHX40 | FBN2 |
| DHX40 | ARRDC4 |
| DHX40 | DNAJA2 |
| DHX40 | CYFIP1 |
| DHX40 | TPM2 |
| DHX40 | BCAT1 |
| DHX40 | FAM199X |
| DHX40 | ADRBK2 |
| DHX40 | LRTOMT |
| DHX40 | GOLPH3 |
| DHX40 | ARF3 |
| DHX40 | TTC28 |
| DHX40 | SCPEP1 |
| DHX40 | CMTM4 |
| DHX40 | F8 |
| DHX40 | FLVCR2 |
| DHX40 | MS4A4A |
| DHX40 | CHMP7 |
| DHX40 | ACP6 |
| DHX40 | BRCA1 |
| DHX40 | TRAPPC6A |
| DHX40 | NPEPL1 |
| DHX40 | PAPSS1 |
| DHX40 | DDIT3 |
| DHX40 | MLC1 |
| DHX40 | FAM110B |
| DHX40 | RRM2B |
| DHX40 | PHTF1 |
| DHX40 | CLIC4 |
| DHX40 | SLC1A3 |
| DHX40 | TLR10 |
| DHX40 | DSE |
| DHX40 | SGMS2 |
| DHX40 | PRMT5 |
| DHX40 | MTL5 |
| DHX40 | KNOP1 |
| DHX40 | VAV3 |
| DHX40 | CREB1 |
| DHX40 | GAPT |
| DHX40 | ATP10D |
| DHX40 | CC2D2B |
| DHX40 | MATK |
| DHX40 | MPZL2 |
| DHX40 | RNASE4 |
| DHX40 | PHACTR1 |
| DHX40 | FLT3 |
| DHX40 | NKAP |
| DHX40 | DLGAP1-AS2 |
| DHX40 | RP1-193H18.2 |
| DHX40 | LINC00282 |
| DHX40 | DKFZP434L187 |
| DHX40 | RP1-30M3.5 |
| DHX40 | RP11-111K18.2 |
| DHX40 | RP11-2E11.9 |
| DHX40 | RP11-399O19.9 |
| DHX40 | KB-431C1.4 |
| DHX40 | RP11-1024P17.1 |
| DHX40 | SNRK-AS1 |
| DHX40 | RP11-476D10.1 |
| KDM3B | SLC12A6 |
| KDM3B | TANC2 |
| KDM3B | IDH1 |
| KDM3B | PAPOLA |
| KDM3B | FEZ2 |
| KDM3B | EAPP |
| KDM3B | HOOK3 |
| KDM3B | CD163 |
| KDM3B | SOAT1 |
| KDM3B | MED31 |
| KDM3B | SENP1 |
| KDM3B | NSMAF |
| KDM3B | PPP5C |
| KDM3B | ATN1 |
| KDM3B | CREBRF |
| KDM3B | VPS41 |
| KDM3B | KIAA2013 |
| KDM3B | SAP30L |
| KDM3B | NAGA |
| KDM3B | TSHZ3 |
| KDM3B | ITFG2 |
| KDM3B | STAM2 |
| KDM3B | SPRED2 |
| KDM3B | PSMC3 |
| KDM3B | IPCEF1 |
| KDM3B | TMEM260 |
| KDM3B | TMED8 |
| KDM3B | LPCAT2 |
| KDM3B | BAZ2B |
| KDM3B | EIF2AK2 |
| KDM3B | TBCC |
| KDM3B | GNAI3 |
| KDM3B | SFN |
| KDM3B | INTS6 |
| KDM3B | TFG |
| KDM3B | MEF2A |
| KDM3B | PRMT1 |
| KDM3B | AREL1 |
| KDM3B | SLX4IP |
| KDM3B | NBN |
| KDM3B | CYBB |
| KDM3B | SELT |
| KDM3B | ASPH |
| KDM3B | BROX |
| KDM3B | FBN2 |
| KDM3B | ARRDC4 |
| KDM3B | TPM2 |
| KDM3B | FAM199X |
| KDM3B | GOLPH3 |
| KDM3B | ARF3 |
| KDM3B | SCPEP1 |
| KDM3B | CMTM4 |
| KDM3B | F8 |
| KDM3B | FLVCR2 |
| KDM3B | CHMP7 |
| KDM3B | BRCA1 |
| KDM3B | PAPSS1 |
| KDM3B | RRM2B |
| KDM3B | PHTF1 |
| KDM3B | DSE |
| KDM3B | VAV3 |
| KDM3B | DLGAP1-AS2 |
| KDM3B | RP1-193H18.2 |
| KDM3B | RP1-30M3.5 |
| KDM3B | RP11-111K18.2 |
| KDM3B | RP11-399O19.9 |
| KDM3B | KB-431C1.4 |
| SLC12A6 | TANC2 |
| SLC12A6 | IDH1 |
| SLC12A6 | PAPOLA |
| SLC12A6 | FEZ2 |
| SLC12A6 | HOOK3 |
| SLC12A6 | SOAT1 |
| SLC12A6 | SENP1 |
| SLC12A6 | NSMAF |
| SLC12A6 | ATN1 |
| SLC12A6 | VPS41 |
| SLC12A6 | KIAA2013 |
| SLC12A6 | TSHZ3 |
| SLC12A6 | ITFG2 |
| SLC12A6 | STAM2 |
| SLC12A6 | TMED8 |
| SLC12A6 | LPCAT2 |
| SLC12A6 | BAZ2B |
| SLC12A6 | TBCC |
| SLC12A6 | GNAI3 |
| SLC12A6 | SFN |
| SLC12A6 | MEF2A |
| SLC12A6 | PRMT1 |
| SLC12A6 | AREL1 |
| SLC12A6 | NBN |
| SLC12A6 | GNB4 |
| SLC12A6 | MMP19 |
| SLC12A6 | TPM2 |
| SLC12A6 | ARF3 |
| SLC12A6 | CHMP7 |
| SLC12A6 | TRAPPC6A |
| SLC12A6 | NOS3 |
| SLC12A6 | KNOP1 |
| SLC12A6 | RLTPR |
| SLC12A6 | DLGAP1-AS2 |
| SLC12A6 | RP1-193H18.2 |
| SLC12A6 | RP1-30M3.5 |
| TANC2 | IDH1 |
| TANC2 | PAPOLA |
| TANC2 | ATXN2L |
| TANC2 | FEZ2 |
| TANC2 | EAPP |
| TANC2 | HOOK3 |
| TANC2 | CD163 |
| TANC2 | SOAT1 |
| TANC2 | MED31 |
| TANC2 | SENP1 |
| TANC2 | NSMAF |
| TANC2 | PPP5C |
| TANC2 | ATN1 |
| TANC2 | CREBRF |
| TANC2 | VPS41 |
| TANC2 | PSMD12 |
| TANC2 | KIAA2013 |
| TANC2 | SAP30L |
| TANC2 | NAGA |
| TANC2 | CLEC5A |
| TANC2 | TSHZ3 |
| TANC2 | ITFG2 |
| TANC2 | STAM2 |
| TANC2 | SPRED2 |
| TANC2 | PSMC3 |
| TANC2 | IPCEF1 |
| TANC2 | TMEM260 |
| TANC2 | TMED8 |
| TANC2 | LPCAT2 |
| TANC2 | WSB1 |
| TANC2 | SCCPDH |
| TANC2 | BAZ2B |
| TANC2 | EIF2AK2 |
| TANC2 | TBCC |
| TANC2 | GNAI3 |
| TANC2 | NEURL1 |
| TANC2 | SFN |
| TANC2 | INTS6 |
| TANC2 | TFG |
| TANC2 | MEF2A |
| TANC2 | ASCC1 |
| TANC2 | PRMT1 |
| TANC2 | CLIC3 |
| TANC2 | UBE2E3 |
| TANC2 | AREL1 |
| TANC2 | S1PR3 |
| TANC2 | SCYL2 |
| TANC2 | SLX4IP |
| TANC2 | NBN |
| TANC2 | PLD4 |
| TANC2 | OGFOD3 |
| TANC2 | CYBB |
| TANC2 | GNB4 |
| TANC2 | SELT |
| TANC2 | ASPH |
| TANC2 | ARL15 |
| TANC2 | MMP19 |
| TANC2 | GPER1 |
| TANC2 | BROX |
| TANC2 | SUMF1 |
| TANC2 | FBN2 |
| TANC2 | ARRDC4 |
| TANC2 | DNAJA2 |
| TANC2 | CYFIP1 |
| TANC2 | TPM2 |
| TANC2 | GOLPH3 |
| TANC2 | ARF3 |
| TANC2 | SCPEP1 |
| TANC2 | CMTM4 |
| TANC2 | ALKBH2 |
| TANC2 | F8 |
| TANC2 | FLVCR2 |
| TANC2 | CHMP7 |
| TANC2 | ACP6 |
| TANC2 | BRCA1 |
| TANC2 | TRAPPC6A |
| TANC2 | PAPSS1 |
| TANC2 | DDIT3 |
| TANC2 | NOS3 |
| TANC2 | RRM2B |
| TANC2 | PHTF1 |
| TANC2 | VAV3 |
| TANC2 | CREB1 |
| TANC2 | GAPT |
| TANC2 | MPZL2 |
| TANC2 | PHACTR1 |
| TANC2 | FLT3 |
| TANC2 | DLGAP1-AS2 |
| TANC2 | RP1-193H18.2 |
| TANC2 | LINC00282 |
| TANC2 | DKFZP434L187 |
| TANC2 | RP1-30M3.5 |
| TANC2 | RP11-111K18.2 |
| TANC2 | RP11-2E11.9 |
| TANC2 | KB-431C1.4 |
| TANC2 | SNRK-AS1 |
| TANC2 | RP11-476D10.1 |
| IDH1 | PAPOLA |
| IDH1 | ATXN2L |
| IDH1 | FEZ2 |
| IDH1 | EAPP |
| IDH1 | HOOK3 |
| IDH1 | CD163 |
| IDH1 | SOAT1 |
| IDH1 | MED31 |
| IDH1 | SENP1 |
| IDH1 | NSMAF |
| IDH1 | PPP5C |
| IDH1 | ATN1 |
| IDH1 | CREBRF |
| IDH1 | VPS41 |
| IDH1 | PSMD12 |
| IDH1 | KIAA2013 |
| IDH1 | SAP30L |
| IDH1 | NAGA |
| IDH1 | CLEC5A |
| IDH1 | TSHZ3 |
| IDH1 | ITFG2 |
| IDH1 | STAM2 |
| IDH1 | SPRED2 |
| IDH1 | SCARB2 |
| IDH1 | IPCEF1 |
| IDH1 | TMEM260 |
| IDH1 | TMED8 |
| IDH1 | LPCAT2 |
| IDH1 | WSB1 |
| IDH1 | SCCPDH |
| IDH1 | BAZ2B |
| IDH1 | EIF2AK2 |
| IDH1 | GNAI3 |
| IDH1 | INTS6 |
| IDH1 | TFG |
| IDH1 | MEF2A |
| IDH1 | PRMT1 |
| IDH1 | GOLPH3L |
| IDH1 | CLIC3 |
| IDH1 | MSX1 |
| IDH1 | AREL1 |
| IDH1 | S1PR3 |
| IDH1 | SCYL2 |
| IDH1 | SLX4IP |
| IDH1 | NBN |
| IDH1 | PLD4 |
| IDH1 | CYBB |
| IDH1 | GNB4 |
| IDH1 | SELT |
| IDH1 | DCUN1D1 |
| IDH1 | ASPH |
| IDH1 | SETD7 |
| IDH1 | CEP164 |
| IDH1 | GPER1 |
| IDH1 | BROX |
| IDH1 | SUMF1 |
| IDH1 | FBN2 |
| IDH1 | ARRDC4 |
| IDH1 | DNAJA2 |
| IDH1 | CYFIP1 |
| IDH1 | ADRBK2 |
| IDH1 | GOLPH3 |
| IDH1 | TTC28 |
| IDH1 | SCPEP1 |
| IDH1 | CMTM4 |
| IDH1 | ALKBH2 |
| IDH1 | F8 |
| IDH1 | FLVCR2 |
| IDH1 | MS4A4A |
| IDH1 | TRAPPC6A |
| IDH1 | EPB41L3 |
| IDH1 | PAPSS1 |
| IDH1 | RRM2B |
| IDH1 | PHTF1 |
| IDH1 | CLIC4 |
| IDH1 | SLC1A3 |
| IDH1 | DSE |
| IDH1 | PRMT5 |
| IDH1 | VAV3 |
| IDH1 | GAPT |
| IDH1 | MATK |
| IDH1 | RNASE4 |
| IDH1 | DLGAP1-AS2 |
| IDH1 | RP1-193H18.2 |
| IDH1 | LINC00282 |
| IDH1 | RP1-30M3.5 |
| IDH1 | RP11-111K18.2 |
| IDH1 | RP11-2E11.9 |
| IDH1 | RP11-399O19.9 |
| IDH1 | KB-431C1.4 |
| PAPOLA | ATXN2L |
| PAPOLA | FEZ2 |
| PAPOLA | EAPP |
| PAPOLA | HOOK3 |
| PAPOLA | CD163 |
| PAPOLA | SOAT1 |
| PAPOLA | MED31 |
| PAPOLA | SENP1 |
| PAPOLA | NSMAF |
| PAPOLA | PPP5C |
| PAPOLA | ATN1 |
| PAPOLA | CREBRF |
| PAPOLA | VPS41 |
| PAPOLA | PSMD12 |
| PAPOLA | KIAA2013 |
| PAPOLA | SAP30L |
| PAPOLA | NAGA |
| PAPOLA | CLEC5A |
| PAPOLA | TSHZ3 |
| PAPOLA | ITFG2 |
| PAPOLA | STAM2 |
| PAPOLA | PSMC3 |
| PAPOLA | SCARB2 |
| PAPOLA | IPCEF1 |
| PAPOLA | TMEM260 |
| PAPOLA | TMED8 |
| PAPOLA | LPCAT2 |
| PAPOLA | WSB1 |
| PAPOLA | SCCPDH |
| PAPOLA | BAZ2B |
| PAPOLA | EIF2AK2 |
| PAPOLA | TBCC |
| PAPOLA | GNAI3 |
| PAPOLA | NEURL1 |
| PAPOLA | INTS6 |
| PAPOLA | TFG |
| PAPOLA | MEF2A |
| PAPOLA | PRMT1 |
| PAPOLA | GOLPH3L |
| PAPOLA | CLIC3 |
| PAPOLA | UBE2E3 |
| PAPOLA | MSX1 |
| PAPOLA | AREL1 |
| PAPOLA | S1PR3 |
| PAPOLA | SCYL2 |
| PAPOLA | SLX4IP |
| PAPOLA | NBN |
| PAPOLA | PLD4 |
| PAPOLA | CYBB |
| PAPOLA | BIRC2 |
| PAPOLA | GNB4 |
| PAPOLA | SELT |
| PAPOLA | DCUN1D1 |
| PAPOLA | ASPH |
| PAPOLA | ARL15 |
| PAPOLA | MMP19 |
| PAPOLA | CEP164 |
| PAPOLA | BROX |
| PAPOLA | SUMF1 |
| PAPOLA | FBN2 |
| PAPOLA | ARRDC4 |
| PAPOLA | DNAJA2 |
| PAPOLA | CYFIP1 |
| PAPOLA | BCAT1 |
| PAPOLA | ADRBK2 |
| PAPOLA | LRTOMT |
| PAPOLA | GOLPH3 |
| PAPOLA | TTC28 |
| PAPOLA | SCPEP1 |
| PAPOLA | CMTM4 |
| PAPOLA | ALKBH2 |
| PAPOLA | FLVCR2 |
| PAPOLA | MS4A4A |
| PAPOLA | ACP6 |
| PAPOLA | BRCA1 |
| PAPOLA | TRAPPC6A |
| PAPOLA | NPEPL1 |
| PAPOLA | PAPSS1 |
| PAPOLA | DDIT3 |
| PAPOLA | RRM2B |
| PAPOLA | PHTF1 |
| PAPOLA | CLIC4 |
| PAPOLA | DSE |
| PAPOLA | PRMT5 |
| PAPOLA | MTL5 |
| PAPOLA | VAV3 |
| PAPOLA | CREB1 |
| PAPOLA | GAPT |
| PAPOLA | NKAP |
| PAPOLA | DLGAP1-AS2 |
| PAPOLA | RP1-193H18.2 |
| PAPOLA | LINC00282 |
| PAPOLA | RP1-30M3.5 |
| PAPOLA | RP11-111K18.2 |
| PAPOLA | RP11-2E11.9 |
| PAPOLA | KB-431C1.4 |
| PAPOLA | SNRK-AS1 |
| PAPOLA | RP11-476D10.1 |
| ATXN2L | FEZ2 |
| ATXN2L | HOOK3 |
| ATXN2L | SOAT1 |
| ATXN2L | SENP1 |
| ATXN2L | NSMAF |
| ATXN2L | ATN1 |
| ATXN2L | VPS41 |
| ATXN2L | KIAA2013 |
| ATXN2L | NAGA |
| ATXN2L | STAM2 |
| ATXN2L | LPCAT2 |
| ATXN2L | SCCPDH |
| ATXN2L | BAZ2B |
| ATXN2L | TBCC |
| ATXN2L | GNAI3 |
| ATXN2L | MEF2A |
| ATXN2L | GNB4 |
| ATXN2L | DNAJA2 |
| ATXN2L | DLGAP1-AS2 |
| ATXN2L | RP1-193H18.2 |
| ATXN2L | RP1-30M3.5 |
| FEZ2 | EAPP |
| FEZ2 | HOOK3 |
| FEZ2 | CD163 |
| FEZ2 | SOAT1 |
| FEZ2 | SENP1 |
| FEZ2 | NSMAF |
| FEZ2 | ATN1 |
| FEZ2 | VPS41 |
| FEZ2 | KIAA2013 |
| FEZ2 | NAGA |
| FEZ2 | CLEC5A |
| FEZ2 | TSHZ3 |
| FEZ2 | ITFG2 |
| FEZ2 | STAM2 |
| FEZ2 | TMEM260 |
| FEZ2 | TMED8 |
| FEZ2 | LPCAT2 |
| FEZ2 | SCCPDH |
| FEZ2 | BAZ2B |
| FEZ2 | TBCC |
| FEZ2 | GNAI3 |
| FEZ2 | TFG |
| FEZ2 | MEF2A |
| FEZ2 | UBE2E3 |
| FEZ2 | AREL1 |
| FEZ2 | S1PR3 |
| FEZ2 | OGFOD3 |
| FEZ2 | CYBB |
| FEZ2 | AK2 |
| FEZ2 | GNB4 |
| FEZ2 | SETD7 |
| FEZ2 | MMP19 |
| FEZ2 | PLIN2 |
| FEZ2 | GPER1 |
| FEZ2 | BROX |
| FEZ2 | SUMF1 |
| FEZ2 | ARRDC4 |
| FEZ2 | CYFIP1 |
| FEZ2 | ARF3 |
| FEZ2 | CMTM4 |
| FEZ2 | FLVCR2 |
| FEZ2 | EPB41L3 |
| FEZ2 | VAV3 |
| FEZ2 | DLGAP1-AS2 |
| FEZ2 | RP1-193H18.2 |
| FEZ2 | RP1-30M3.5 |
| EAPP | HOOK3 |
| EAPP | CD163 |
| EAPP | SOAT1 |
| EAPP | MED31 |
| EAPP | SENP1 |
| EAPP | NSMAF |
| EAPP | PPP5C |
| EAPP | ATN1 |
| EAPP | CREBRF |
| EAPP | VPS41 |
| EAPP | PSMD12 |
| EAPP | KIAA2013 |
| EAPP | SAP30L |
| EAPP | CLEC5A |
| EAPP | TSHZ3 |
| EAPP | ITFG2 |
| EAPP | STAM2 |
| EAPP | SPRED2 |
| EAPP | PSMC3 |
| EAPP | SCARB2 |
| EAPP | IPCEF1 |
| EAPP | TMEM260 |
| EAPP | TMED8 |
| EAPP | LPCAT2 |
| EAPP | WSB1 |
| EAPP | SCCPDH |
| EAPP | BAZ2B |
| EAPP | NDST3 |
| EAPP | GORASP2 |
| EAPP | EIF2AK2 |
| EAPP | TBCC |
| EAPP | GNAI3 |
| EAPP | MPPED2 |
| EAPP | NEURL1 |
| EAPP | INTS6 |
| EAPP | TFG |
| EAPP | MEF2A |
| EAPP | GOLPH3L |
| EAPP | CLIC3 |
| EAPP | SCYL2 |
| EAPP | SLX4IP |
| EAPP | RNF165 |
| EAPP | NBN |
| EAPP | PLD4 |
| EAPP | BIRC2 |
| EAPP | GNB4 |
| EAPP | SELT |
| EAPP | DCUN1D1 |
| EAPP | ASPH |
| EAPP | CEP164 |
| EAPP | BROX |
| EAPP | SUMF1 |
| EAPP | ARRDC4 |
| EAPP | DNAJA2 |
| EAPP | FAM199X |
| EAPP | LRTOMT |
| EAPP | GOLPH3 |
| EAPP | TTC28 |
| EAPP | SCPEP1 |
| EAPP | CMTM4 |
| EAPP | F8 |
| EAPP | MS4A4A |
| EAPP | ACP6 |
| EAPP | BRCA1 |
| EAPP | NPEPL1 |
| EAPP | PAPSS1 |
| EAPP | DDIT3 |
| EAPP | RRM2B |
| EAPP | PHTF1 |
| EAPP | TLR10 |
| EAPP | DSE |
| EAPP | PRMT5 |
| EAPP | MTL5 |
| EAPP | VAV3 |
| EAPP | CREB1 |
| EAPP | GAPT |
| EAPP | ATP10D |
| EAPP | CC2D2B |
| EAPP | NKAP |
| EAPP | IFIT5 |
| EAPP | DLGAP1-AS2 |
| EAPP | RP1-193H18.2 |
| EAPP | LINC00282 |
| EAPP | DKFZP434L187 |
| EAPP | RP1-30M3.5 |
| EAPP | RP11-111K18.2 |
| EAPP | RP11-399O19.9 |
| EAPP | KB-431C1.4 |
| EAPP | RP11-1024P17.1 |
| EAPP | SNRK-AS1 |
| EAPP | RP11-476D10.1 |
| HOOK3 | CD163 |
| HOOK3 | SOAT1 |
| HOOK3 | MED31 |
| HOOK3 | SENP1 |
| HOOK3 | NSMAF |
| HOOK3 | PPP5C |
| HOOK3 | ATN1 |
| HOOK3 | CREBRF |
| HOOK3 | VPS41 |
| HOOK3 | PSMD12 |
| HOOK3 | SAP30L |
| HOOK3 | NAGA |
| HOOK3 | CLEC5A |
| HOOK3 | TSHZ3 |
| HOOK3 | ITFG2 |
| HOOK3 | STAM2 |
| HOOK3 | PSMC3 |
| HOOK3 | SCARB2 |
| HOOK3 | IPCEF1 |
| HOOK3 | TMEM260 |
| HOOK3 | TMED8 |
| HOOK3 | LPCAT2 |
| HOOK3 | WSB1 |
| HOOK3 | SCCPDH |
| HOOK3 | BAZ2B |
| HOOK3 | GORASP2 |
| HOOK3 | EIF2AK2 |
| HOOK3 | TBCC |
| HOOK3 | GNAI3 |
| HOOK3 | NEURL1 |
| HOOK3 | INTS6 |
| HOOK3 | TFG |
| HOOK3 | MEF2A |
| HOOK3 | ASCC1 |
| HOOK3 | PRMT1 |
| HOOK3 | GOLPH3L |
| HOOK3 | CLIC3 |
| HOOK3 | UBE2E3 |
| HOOK3 | MSX1 |
| HOOK3 | S1PR3 |
| HOOK3 | SCYL2 |
| HOOK3 | SLX4IP |
| HOOK3 | NBN |
| HOOK3 | OGFOD3 |
| HOOK3 | CYBB |
| HOOK3 | BIRC2 |
| HOOK3 | GNB4 |
| HOOK3 | SELT |
| HOOK3 | DCUN1D1 |
| HOOK3 | ASPH |
| HOOK3 | SETD7 |
| HOOK3 | ARL15 |
| HOOK3 | CEP164 |
| HOOK3 | PLIN2 |
| HOOK3 | GPER1 |
| HOOK3 | BROX |
| HOOK3 | SUMF1 |
| HOOK3 | ARRDC4 |
| HOOK3 | DNAJA2 |
| HOOK3 | CYFIP1 |
| HOOK3 | FAM199X |
| HOOK3 | ADRBK2 |
| HOOK3 | LRTOMT |
| HOOK3 | GOLPH3 |
| HOOK3 | SCPEP1 |
| HOOK3 | CMTM4 |
| HOOK3 | F8 |
| HOOK3 | FLVCR2 |
| HOOK3 | ACP6 |
| HOOK3 | NPEPL1 |
| HOOK3 | PAPSS1 |
| HOOK3 | DDIT3 |
| HOOK3 | RRM2B |
| HOOK3 | PHTF1 |
| HOOK3 | CLIC4 |
| HOOK3 | SLC1A3 |
| HOOK3 | TLR10 |
| HOOK3 | DSE |
| HOOK3 | PRMT5 |
| HOOK3 | MTL5 |
| HOOK3 | VAV3 |
| HOOK3 | CREB1 |
| HOOK3 | GAPT |
| HOOK3 | ATP10D |
| HOOK3 | CC2D2B |
| HOOK3 | RNASE4 |
| HOOK3 | FLT3 |
| HOOK3 | DLGAP1-AS2 |
| HOOK3 | RP1-193H18.2 |
| HOOK3 | LINC00282 |
| HOOK3 | RP1-30M3.5 |
| HOOK3 | RP11-111K18.2 |
| HOOK3 | KB-431C1.4 |
| HOOK3 | SNRK-AS1 |
| CD163 | SOAT1 |
| CD163 | NSMAF |
| CD163 | VPS41 |
| CD163 | KIAA2013 |
| CD163 | NAGA |
| CD163 | CLEC5A |
| CD163 | TSHZ3 |
| CD163 | STAM2 |
| CD163 | SPRED2 |
| CD163 | LPCAT2 |
| CD163 | BAZ2B |
| CD163 | GNAI3 |
| CD163 | MEF2A |
| CD163 | AREL1 |
| CD163 | S1PR3 |
| CD163 | CYBB |
| CD163 | GNB4 |
| CD163 | SETD7 |
| CD163 | GPER1 |
| CD163 | BROX |
| CD163 | FBN2 |
| CD163 | CYFIP1 |
| CD163 | ARF3 |
| CD163 | SCPEP1 |
| CD163 | FLVCR2 |
| CD163 | EPB41L3 |
| CD163 | LDLRAD3 |
| CD163 | SLC1A3 |
| CD163 | SGMS2 |
| CD163 | RNASE4 |
| CD163 | FLT3 |
| CD163 | DLGAP1-AS2 |
| CD163 | RP1-193H18.2 |
| SOAT1 | MED31 |
| SOAT1 | SENP1 |
| SOAT1 | NSMAF |
| SOAT1 | PPP5C |
| SOAT1 | ATN1 |
| SOAT1 | CREBRF |
| SOAT1 | VPS41 |
| SOAT1 | PSMD12 |
| SOAT1 | KIAA2013 |
| SOAT1 | SAP30L |
| SOAT1 | NAGA |
| SOAT1 | CLEC5A |
| SOAT1 | TSHZ3 |
| SOAT1 | ITFG2 |
| SOAT1 | STAM2 |
| SOAT1 | SCARB2 |
| SOAT1 | IPCEF1 |
| SOAT1 | TMEM260 |
| SOAT1 | TMED8 |
| SOAT1 | LPCAT2 |
| SOAT1 | WSB1 |
| SOAT1 | SCCPDH |
| SOAT1 | BAZ2B |
| SOAT1 | EIF2AK2 |
| SOAT1 | TBCC |
| SOAT1 | GNAI3 |
| SOAT1 | MPPED2 |
| SOAT1 | INTS6 |
| SOAT1 | TFG |
| SOAT1 | MEF2A |
| SOAT1 | PRMT1 |
| SOAT1 | GOLPH3L |
| SOAT1 | CLIC3 |
| SOAT1 | UBE2E3 |
| SOAT1 | AREL1 |
| SOAT1 | S1PR3 |
| SOAT1 | SCYL2 |
| SOAT1 | SLX4IP |
| SOAT1 | NBN |
| SOAT1 | PLD4 |
| SOAT1 | CYBB |
| SOAT1 | GNB4 |
| SOAT1 | SELT |
| SOAT1 | DCUN1D1 |
| SOAT1 | ASPH |
| SOAT1 | SETD7 |
| SOAT1 | CEP164 |
| SOAT1 | GPER1 |
| SOAT1 | BROX |
| SOAT1 | SUMF1 |
| SOAT1 | FBN2 |
| SOAT1 | ARRDC4 |
| SOAT1 | DNAJA2 |
| SOAT1 | CYFIP1 |
| SOAT1 | LRTOMT |
| SOAT1 | GOLPH3 |
| SOAT1 | TTC28 |
| SOAT1 | SCPEP1 |
| SOAT1 | CMTM4 |
| SOAT1 | FLVCR2 |
| SOAT1 | MS4A4A |
| SOAT1 | TRAPPC6A |
| SOAT1 | DDIT3 |
| SOAT1 | RRM2B |
| SOAT1 | PHTF1 |
| SOAT1 | SLC1A3 |
| SOAT1 | PRMT5 |
| SOAT1 | MTL5 |
| SOAT1 | VAV3 |
| SOAT1 | DLGAP1-AS2 |
| SOAT1 | RP1-193H18.2 |
| SOAT1 | LINC00282 |
| SOAT1 | RP1-30M3.5 |
| SOAT1 | RP11-111K18.2 |
| SOAT1 | RP11-2E11.9 |
| SOAT1 | KB-431C1.4 |
| SOAT1 | SNRK-AS1 |
| MED31 | SENP1 |
| MED31 | NSMAF |
| MED31 | PPP5C |
| MED31 | CREBRF |
| MED31 | VPS41 |
| MED31 | PSMD12 |
| MED31 | KIAA2013 |
| MED31 | STAM2 |
| MED31 | TMEM260 |
| MED31 | TMED8 |
| MED31 | LPCAT2 |
| MED31 | SCCPDH |
| MED31 | BAZ2B |
| MED31 | TBCC |
| MED31 | GNAI3 |
| MED31 | MEF2A |
| MED31 | GNB4 |
| MED31 | SELT |
| MED31 | BROX |
| MED31 | DNAJA2 |
| MED31 | PFN4 |
| MED31 | DLGAP1-AS2 |
| MED31 | RP1-193H18.2 |
| MED31 | RP1-30M3.5 |
| MED31 | RP11-111K18.2 |
| MED31 | KB-431C1.4 |
| SENP1 | NSMAF |
| SENP1 | PPP5C |
| SENP1 | ATN1 |
| SENP1 | CREBRF |
| SENP1 | VPS41 |
| SENP1 | PSMD12 |
| SENP1 | KIAA2013 |
| SENP1 | SAP30L |
| SENP1 | ITFG2 |
| SENP1 | STAM2 |
| SENP1 | SCARB2 |
| SENP1 | TMEM260 |
| SENP1 | TMED8 |
| SENP1 | LPCAT2 |
| SENP1 | SCCPDH |
| SENP1 | BAZ2B |
| SENP1 | EIF2AK2 |
| SENP1 | TBCC |
| SENP1 | GNAI3 |
| SENP1 | NEURL1 |
| SENP1 | INTS6 |
| SENP1 | TFG |
| SENP1 | MEF2A |
| SENP1 | ASCC1 |
| SENP1 | GOLPH3L |
| SENP1 | CLIC3 |
| SENP1 | UBE2E3 |
| SENP1 | SCYL2 |
| SENP1 | SLX4IP |
| SENP1 | NBN |
| SENP1 | OGFOD3 |
| SENP1 | AK2 |
| SENP1 | BIRC2 |
| SENP1 | GNB4 |
| SENP1 | SELT |
| SENP1 | DCUN1D1 |
| SENP1 | ASPH |
| SENP1 | BROX |
| SENP1 | ARRDC4 |
| SENP1 | DNAJA2 |
| SENP1 | ADRBK2 |
| SENP1 | GOLPH3 |
| SENP1 | CMTM4 |
| SENP1 | FLVCR2 |
| SENP1 | PAPSS1 |
| SENP1 | RRM2B |
| SENP1 | CLIC4 |
| SENP1 | MTL5 |
| SENP1 | CREB1 |
| SENP1 | GAPT |
| SENP1 | ATP10D |
| SENP1 | DLGAP1-AS2 |
| SENP1 | RP1-193H18.2 |
| SENP1 | LINC00282 |
| SENP1 | RP1-30M3.5 |
| SENP1 | RP11-111K18.2 |
| SENP1 | KB-431C1.4 |
| NSMAF | PPP5C |
| NSMAF | ATN1 |
| NSMAF | CREBRF |
| NSMAF | VPS41 |
| NSMAF | PSMD12 |
| NSMAF | KIAA2013 |
| NSMAF | SAP30L |
| NSMAF | NAGA |
| NSMAF | CLEC5A |
| NSMAF | TSHZ3 |
| NSMAF | ITFG2 |
| NSMAF | STAM2 |
| NSMAF | SPRED2 |
| NSMAF | PSMC3 |
| NSMAF | SCARB2 |
| NSMAF | IPCEF1 |
| NSMAF | TMEM260 |
| NSMAF | TMED8 |
| NSMAF | LPCAT2 |
| NSMAF | WSB1 |
| NSMAF | SCCPDH |
| NSMAF | C7orf73 |
| NSMAF | BAZ2B |
| NSMAF | NDST3 |
| NSMAF | GORASP2 |
| NSMAF | EIF2AK2 |
| NSMAF | TBCC |
| NSMAF | GNAI3 |
| NSMAF | MPPED2 |
| NSMAF | NEURL1 |
| NSMAF | SFN |
| NSMAF | INTS6 |
| NSMAF | TFG |
| NSMAF | MEF2A |
| NSMAF | ASCC1 |
| NSMAF | PRMT1 |
| NSMAF | GOLPH3L |
| NSMAF | CLIC3 |
| NSMAF | UBE2E3 |
| NSMAF | MSX1 |
| NSMAF | AREL1 |
| NSMAF | S1PR3 |
| NSMAF | SCYL2 |
| NSMAF | SLX4IP |
| NSMAF | RNF165 |
| NSMAF | NBN |
| NSMAF | PLD4 |
| NSMAF | OGFOD3 |
| NSMAF | CYBB |
| NSMAF | BIRC2 |
| NSMAF | GNB4 |
| NSMAF | SELT |
| NSMAF | DCUN1D1 |
| NSMAF | ASPH |
| NSMAF | SETD7 |
| NSMAF | ARL15 |
| NSMAF | MMP19 |
| NSMAF | CEP164 |
| NSMAF | GPER1 |
| NSMAF | BROX |
| NSMAF | SUMF1 |
| NSMAF | FBN2 |
| NSMAF | ARRDC4 |
| NSMAF | DNAJA2 |
| NSMAF | CYFIP1 |
| NSMAF | TPM2 |
| NSMAF | BCAT1 |
| NSMAF | FAM199X |
| NSMAF | ADRBK2 |
| NSMAF | LRTOMT |
| NSMAF | GOLPH3 |
| NSMAF | TTC28 |
| NSMAF | SCPEP1 |
| NSMAF | CMTM4 |
| NSMAF | ALKBH2 |
| NSMAF | F8 |
| NSMAF | FLVCR2 |
| NSMAF | MS4A4A |
| NSMAF | CHMP7 |
| NSMAF | ACP6 |
| NSMAF | BRCA1 |
| NSMAF | TRAPPC6A |
| NSMAF | NPEPL1 |
| NSMAF | PAPSS1 |
| NSMAF | DDIT3 |
| NSMAF | MLC1 |
| NSMAF | RRM2B |
| NSMAF | PHTF1 |
| NSMAF | CLIC4 |
| NSMAF | SLC1A3 |
| NSMAF | TLR10 |
| NSMAF | DSE |
| NSMAF | PRMT5 |
| NSMAF | MTL5 |
| NSMAF | KNOP1 |
| NSMAF | VAV3 |
| NSMAF | CREB1 |
| NSMAF | GAPT |
| NSMAF | ATP10D |
| NSMAF | CC2D2B |
| NSMAF | MATK |
| NSMAF | MPZL2 |
| NSMAF | RNASE4 |
| NSMAF | PHACTR1 |
| NSMAF | NETO2 |
| NSMAF | FLT3 |
| NSMAF | NKAP |
| NSMAF | PAPSS2 |
| NSMAF | DLGAP1-AS2 |
| NSMAF | RP1-193H18.2 |
| NSMAF | LINC00282 |
| NSMAF | DKFZP434L187 |
| NSMAF | RP1-30M3.5 |
| NSMAF | RP11-111K18.2 |
| NSMAF | RP11-2E11.9 |
| NSMAF | RP11-399O19.9 |
| NSMAF | KB-431C1.4 |
| NSMAF | RP11-1024P17.1 |
| NSMAF | SNRK-AS1 |
| NSMAF | AC005785.2 |
| NSMAF | RP11-476D10.1 |
| PPP5C | CREBRF |
| PPP5C | VPS41 |
| PPP5C | PSMD12 |
| PPP5C | KIAA2013 |
| PPP5C | TSHZ3 |
| PPP5C | STAM2 |
| PPP5C | TMEM260 |
| PPP5C | TMED8 |
| PPP5C | LPCAT2 |
| PPP5C | SCCPDH |
| PPP5C | BAZ2B |
| PPP5C | TBCC |
| PPP5C | GNAI3 |
| PPP5C | MEF2A |
| PPP5C | GNB4 |
| PPP5C | BROX |
| PPP5C | DNAJA2 |
| PPP5C | DLGAP1-AS2 |
| PPP5C | RP1-193H18.2 |
| PPP5C | RP1-30M3.5 |
| PPP5C | KB-431C1.4 |
| ATN1 | CREBRF |
| ATN1 | VPS41 |
| ATN1 | KIAA2013 |
| ATN1 | TSHZ3 |
| ATN1 | STAM2 |
| ATN1 | TMEM260 |
| ATN1 | TMED8 |
| ATN1 | LPCAT2 |
| ATN1 | BAZ2B |
| ATN1 | TBCC |
| ATN1 | GNAI3 |
| ATN1 | MEF2A |
| ATN1 | PRMT1 |
| ATN1 | NBN |
| ATN1 | GNB4 |
| ATN1 | SELT |
| ATN1 | BROX |
| ATN1 | C5orf45 |
| ATN1 | DNAJA2 |
| ATN1 | LRTOMT |
| ATN1 | GOLPH3 |
| ATN1 | PIEZO1 |
| ATN1 | CHMP7 |
| ATN1 | RRM2B |
| ATN1 | RLTPR |
| ATN1 | DLGAP1-AS2 |
| ATN1 | RP1-193H18.2 |
| ATN1 | RP1-30M3.5 |
| ATN1 | KB-431C1.4 |
| CREBRF | VPS41 |
| CREBRF | PSMD12 |
| CREBRF | SAP30L |
| CREBRF | CLEC5A |
| CREBRF | TSHZ3 |
| CREBRF | STAM2 |
| CREBRF | PSMC3 |
| CREBRF | SCARB2 |
| CREBRF | IPCEF1 |
| CREBRF | TMEM260 |
| CREBRF | TMED8 |
| CREBRF | LPCAT2 |
| CREBRF | WSB1 |
| CREBRF | SCCPDH |
| CREBRF | BAZ2B |
| CREBRF | EIF2AK2 |
| CREBRF | TBCC |
| CREBRF | GNAI3 |
| CREBRF | MPPED2 |
| CREBRF | NEURL1 |
| CREBRF | INTS6 |
| CREBRF | TFG |
| CREBRF | MEF2A |
| CREBRF | GOLPH3L |
| CREBRF | CLIC3 |
| CREBRF | MSX1 |
| CREBRF | SCYL2 |
| CREBRF | SLX4IP |
| CREBRF | RNF165 |
| CREBRF | NBN |
| CREBRF | PLD4 |
| CREBRF | BIRC2 |
| CREBRF | GNB4 |
| CREBRF | SELT |
| CREBRF | DCUN1D1 |
| CREBRF | ASPH |
| CREBRF | CEP164 |
| CREBRF | BROX |
| CREBRF | SUMF1 |
| CREBRF | ARRDC4 |
| CREBRF | DNAJA2 |
| CREBRF | FAM199X |
| CREBRF | LRTOMT |
| CREBRF | GOLPH3 |
| CREBRF | TTC28 |
| CREBRF | SCPEP1 |
| CREBRF | CMTM4 |
| CREBRF | F8 |
| CREBRF | ACP6 |
| CREBRF | BRCA1 |
| CREBRF | NPEPL1 |
| CREBRF | PAPSS1 |
| CREBRF | DDIT3 |
| CREBRF | RRM2B |
| CREBRF | PHTF1 |
| CREBRF | CLIC4 |
| CREBRF | TLR10 |
| CREBRF | DSE |
| CREBRF | PRMT5 |
| CREBRF | MTL5 |
| CREBRF | VAV3 |
| CREBRF | CREB1 |
| CREBRF | GAPT |
| CREBRF | ATP10D |
| CREBRF | CC2D2B |
| CREBRF | NKAP |
| CREBRF | IFIT5 |
| CREBRF | DLGAP1-AS2 |
| CREBRF | RP1-193H18.2 |
| CREBRF | LINC00282 |
| CREBRF | DKFZP434L187 |
| CREBRF | RP1-30M3.5 |
| CREBRF | RP11-111K18.2 |
| CREBRF | RP11-399O19.9 |
| CREBRF | KB-431C1.4 |
| CREBRF | RP11-1024P17.1 |
| CREBRF | SNRK-AS1 |
| CREBRF | RP11-476D10.1 |
| VPS41 | PSMD12 |
| VPS41 | KIAA2013 |
| VPS41 | SAP30L |
| VPS41 | NAGA |
| VPS41 | CLEC5A |
| VPS41 | TSHZ3 |
| VPS41 | ITFG2 |
| VPS41 | STAM2 |
| VPS41 | SCARB2 |
| VPS41 | IPCEF1 |
| VPS41 | TMEM260 |
| VPS41 | TMED8 |
| VPS41 | LPCAT2 |
| VPS41 | WSB1 |
| VPS41 | SCCPDH |
| VPS41 | BAZ2B |
| VPS41 | EIF2AK2 |
| VPS41 | TBCC |
| VPS41 | GNAI3 |
| VPS41 | NEURL1 |
| VPS41 | SFN |
| VPS41 | INTS6 |
| VPS41 | TFG |
| VPS41 | MEF2A |
| VPS41 | PRMT1 |
| VPS41 | UBE2E3 |
| VPS41 | AREL1 |
| VPS41 | S1PR3 |
| VPS41 | SCYL2 |
| VPS41 | SLX4IP |
| VPS41 | NBN |
| VPS41 | PLD4 |
| VPS41 | OGFOD3 |
| VPS41 | CYBB |
| VPS41 | AK2 |
| VPS41 | GNB4 |
| VPS41 | SELT |
| VPS41 | ASPH |
| VPS41 | SETD7 |
| VPS41 | ARL15 |
| VPS41 | PLIN2 |
| VPS41 | GPER1 |
| VPS41 | BROX |
| VPS41 | SUMF1 |
| VPS41 | ARRDC4 |
| VPS41 | DNAJA2 |
| VPS41 | CYFIP1 |
| VPS41 | ADRBK2 |
| VPS41 | LRTOMT |
| VPS41 | GOLPH3 |
| VPS41 | SCPEP1 |
| VPS41 | CMTM4 |
| VPS41 | F8 |
| VPS41 | FLVCR2 |
| VPS41 | ACP6 |
| VPS41 | BRCA1 |
| VPS41 | PAPSS1 |
| VPS41 | DDIT3 |
| VPS41 | RRM2B |
| VPS41 | PHTF1 |
| VPS41 | MTL5 |
| VPS41 | VAV3 |
| VPS41 | CREB1 |
| VPS41 | GAPT |
| VPS41 | ATP10D |
| VPS41 | ENOX1 |
| VPS41 | MPZL2 |
| VPS41 | RNASE4 |
| VPS41 | FLT3 |
| VPS41 | DLGAP1-AS2 |
| VPS41 | RP1-193H18.2 |
| VPS41 | LINC00282 |
| VPS41 | DKFZP434L187 |
| VPS41 | RP1-30M3.5 |
| VPS41 | RP11-111K18.2 |
| VPS41 | RP11-2E11.9 |
| VPS41 | KB-431C1.4 |
| VPS41 | SNRK-AS1 |
| PSMD12 | KIAA2013 |
| PSMD12 | SAP30L |
| PSMD12 | CLEC5A |
| PSMD12 | STAM2 |
| PSMD12 | PSMC3 |
| PSMD12 | SCARB2 |
| PSMD12 | IPCEF1 |
| PSMD12 | TMEM260 |
| PSMD12 | TMED8 |
| PSMD12 | LPCAT2 |
| PSMD12 | WSB1 |
| PSMD12 | SCCPDH |
| PSMD12 | BAZ2B |
| PSMD12 | GORASP2 |
| PSMD12 | EIF2AK2 |
| PSMD12 | TBCC |
| PSMD12 | MPPED2 |
| PSMD12 | NEURL1 |
| PSMD12 | INTS6 |
| PSMD12 | TFG |
| PSMD12 | MEF2A |
| PSMD12 | GOLPH3L |
| PSMD12 | CLIC3 |
| PSMD12 | MSX1 |
| PSMD12 | SCYL2 |
| PSMD12 | SLX4IP |
| PSMD12 | NBN |
| PSMD12 | BIRC2 |
| PSMD12 | GNB4 |
| PSMD12 | SELT |
| PSMD12 | DCUN1D1 |
| PSMD12 | ASPH |
| PSMD12 | BROX |
| PSMD12 | SUMF1 |
| PSMD12 | ARRDC4 |
| PSMD12 | DNAJA2 |
| PSMD12 | FAM199X |
| PSMD12 | LRTOMT |
| PSMD12 | GOLPH3 |
| PSMD12 | SCPEP1 |
| PSMD12 | CMTM4 |
| PSMD12 | F8 |
| PSMD12 | NPEPL1 |
| PSMD12 | PAPSS1 |
| PSMD12 | DDIT3 |
| PSMD12 | RRM2B |
| PSMD12 | CLIC4 |
| PSMD12 | DSE |
| PSMD12 | PRMT5 |
| PSMD12 | CREB1 |
| PSMD12 | GAPT |
| PSMD12 | DLGAP1-AS2 |
| PSMD12 | RP1-193H18.2 |
| PSMD12 | LINC00282 |
| PSMD12 | DKFZP434L187 |
| PSMD12 | RP1-30M3.5 |
| PSMD12 | RP11-111K18.2 |
| PSMD12 | KB-431C1.4 |
| SHC4 | DLGAP1-AS2 |
| SHC4 | RP1-193H18.2 |
| KIAA2013 | SAP30L |
| KIAA2013 | NAGA |
| KIAA2013 | CLEC5A |
| KIAA2013 | TSHZ3 |
| KIAA2013 | ITFG2 |
| KIAA2013 | STAM2 |
| KIAA2013 | SPRED2 |
| KIAA2013 | IPCEF1 |
| KIAA2013 | TMED8 |
| KIAA2013 | LPCAT2 |
| KIAA2013 | WSB1 |
| KIAA2013 | SCCPDH |
| KIAA2013 | BAZ2B |
| KIAA2013 | TBCC |
| KIAA2013 | GNAI3 |
| KIAA2013 | SFN |
| KIAA2013 | INTS6 |
| KIAA2013 | TFG |
| KIAA2013 | PRMT1 |
| KIAA2013 | UBE2E3 |
| KIAA2013 | AREL1 |
| KIAA2013 | NBN |
| KIAA2013 | CYBB |
| KIAA2013 | GNB4 |
| KIAA2013 | SELT |
| KIAA2013 | ASPH |
| KIAA2013 | MMP19 |
| KIAA2013 | GPER1 |
| KIAA2013 | BROX |
| KIAA2013 | SUMF1 |
| KIAA2013 | FBN2 |
| KIAA2013 | ARRDC4 |
| KIAA2013 | DNAJA2 |
| KIAA2013 | CYFIP1 |
| KIAA2013 | TPM2 |
| KIAA2013 | GOLPH3 |
| KIAA2013 | ARF3 |
| KIAA2013 | SCPEP1 |
| KIAA2013 | CMTM4 |
| KIAA2013 | F8 |
| KIAA2013 | FLVCR2 |
| KIAA2013 | CHMP7 |
| KIAA2013 | BRCA1 |
| KIAA2013 | TRAPPC6A |
| KIAA2013 | PAPSS1 |
| KIAA2013 | NOS3 |
| KIAA2013 | FAM110B |
| KIAA2013 | RRM2B |
| KIAA2013 | PHTF1 |
| KIAA2013 | VAV3 |
| KIAA2013 | MPZL2 |
| KIAA2013 | PHACTR1 |
| KIAA2013 | DLGAP1-AS2 |
| KIAA2013 | RP1-193H18.2 |
| KIAA2013 | LINC00282 |
| KIAA2013 | RP1-30M3.5 |
| KIAA2013 | RP11-111K18.2 |
| KIAA2013 | RP11-2E11.9 |
| KIAA2013 | KB-431C1.4 |
| KIAA2013 | RP11-476D10.1 |
| SAP30L | TSHZ3 |
| SAP30L | STAM2 |
| SAP30L | TMEM260 |
| SAP30L | TMED8 |
| SAP30L | LPCAT2 |
| SAP30L | BAZ2B |
| SAP30L | TBCC |
| SAP30L | GNAI3 |
| SAP30L | MEF2A |
| SAP30L | GNB4 |
| SAP30L | BROX |
| SAP30L | DLGAP1-AS2 |
| SAP30L | RP1-193H18.2 |
| SAP30L | RP1-30M3.5 |
| SAP30L | KB-431C1.4 |
| SAP30L | RP11-476D10.1 |
| NAGA | TSHZ3 |
| NAGA | STAM2 |
| NAGA | LPCAT2 |
| NAGA | BAZ2B |
| NAGA | GNAI3 |
| NAGA | AREL1 |
| NAGA | S1PR3 |
| NAGA | CYBB |
| NAGA | GNB4 |
| NAGA | SETD7 |
| NAGA | GPER1 |
| NAGA | SUMF1 |
| NAGA | FBN2 |
| NAGA | CYFIP1 |
| NAGA | ARF3 |
| NAGA | SCPEP1 |
| NAGA | CMTM4 |
| NAGA | FLVCR2 |
| NAGA | EPB41L3 |
| NAGA | LDLRAD3 |
| NAGA | SGMS2 |
| NAGA | RLTPR |
| NAGA | RNASE4 |
| NAGA | DLGAP1-AS2 |
| NAGA | RP1-193H18.2 |
| CLEC5A | STAM2 |
| CLEC5A | TMEM260 |
| CLEC5A | TMED8 |
| CLEC5A | LPCAT2 |
| CLEC5A | BAZ2B |
| CLEC5A | TBCC |
| CLEC5A | GNAI3 |
| CLEC5A | MEF2A |
| CLEC5A | GNB4 |
| CLEC5A | SELT |
| CLEC5A | ASPH |
| CLEC5A | BROX |
| CLEC5A | DLGAP1-AS2 |
| CLEC5A | RP1-193H18.2 |
| CLEC5A | RP1-30M3.5 |
| TSHZ3 | STAM2 |
| TSHZ3 | TMED8 |
| TSHZ3 | LPCAT2 |
| TSHZ3 | WSB1 |
| TSHZ3 | BAZ2B |
| TSHZ3 | GNAI3 |
| TSHZ3 | MEF2A |
| TSHZ3 | AREL1 |
| TSHZ3 | CYBB |
| TSHZ3 | GNB4 |
| TSHZ3 | BROX |
| TSHZ3 | FBN2 |
| TSHZ3 | ARF3 |
| TSHZ3 | SCPEP1 |
| TSHZ3 | VAV3 |
| TSHZ3 | DLGAP1-AS2 |
| TSHZ3 | RP1-193H18.2 |
| ITFG2 | STAM2 |
| ITFG2 | LPCAT2 |
| ITFG2 | BAZ2B |
| ITFG2 | TBCC |
| ITFG2 | GNAI3 |
| ITFG2 | MEF2A |
| ITFG2 | GNB4 |
| ITFG2 | DLGAP1-AS2 |
| ITFG2 | RP1-193H18.2 |
| ITFG2 | RP1-30M3.5 |
| STAM2 | PSMC3 |
| STAM2 | SCARB2 |
| STAM2 | IPCEF1 |
| STAM2 | TMEM260 |
| STAM2 | TMED8 |
| STAM2 | LPCAT2 |
| STAM2 | WSB1 |
| STAM2 | SCCPDH |
| STAM2 | C7orf73 |
| STAM2 | BAZ2B |
| STAM2 | EIF2AK2 |
| STAM2 | TBCC |
| STAM2 | GNAI3 |
| STAM2 | MPPED2 |
| STAM2 | NEURL1 |
| STAM2 | INTS6 |
| STAM2 | TFG |
| STAM2 | MEF2A |
| STAM2 | PRMT1 |
| STAM2 | GOLPH3L |
| STAM2 | CLIC3 |
| STAM2 | UBE2E3 |
| STAM2 | MSX1 |
| STAM2 | AREL1 |
| STAM2 | SCYL2 |
| STAM2 | SLX4IP |
| STAM2 | NBN |
| STAM2 | OGFOD3 |
| STAM2 | CYBB |
| STAM2 | BIRC2 |
| STAM2 | GNB4 |
| STAM2 | SELT |
| STAM2 | DCUN1D1 |
| STAM2 | ASPH |
| STAM2 | SETD7 |
| STAM2 | BROX |
| STAM2 | SUMF1 |
| STAM2 | FBN2 |
| STAM2 | ARRDC4 |
| STAM2 | DNAJA2 |
| STAM2 | BCAT1 |
| STAM2 | FAM199X |
| STAM2 | ADRBK2 |
| STAM2 | LRTOMT |
| STAM2 | GOLPH3 |
| STAM2 | TTC28 |
| STAM2 | SCPEP1 |
| STAM2 | CMTM4 |
| STAM2 | F8 |
| STAM2 | FLVCR2 |
| STAM2 | BRCA1 |
| STAM2 | TRAPPC6A |
| STAM2 | NPEPL1 |
| STAM2 | PAPSS1 |
| STAM2 | DDIT3 |
| STAM2 | FAM110B |
| STAM2 | RRM2B |
| STAM2 | PHTF1 |
| STAM2 | CLIC4 |
| STAM2 | DSE |
| STAM2 | PRMT5 |
| STAM2 | MTL5 |
| STAM2 | VAV3 |
| STAM2 | CREB1 |
| STAM2 | GAPT |
| STAM2 | ATP10D |
| STAM2 | CC2D2B |
| STAM2 | MPZL2 |
| STAM2 | PHACTR1 |
| STAM2 | NKAP |
| STAM2 | DLGAP1-AS2 |
| STAM2 | RP1-193H18.2 |
| STAM2 | LINC00282 |
| STAM2 | RP1-30M3.5 |
| STAM2 | RP11-111K18.2 |
| STAM2 | RP11-399O19.9 |
| STAM2 | KB-431C1.4 |
| STAM2 | RP11-1024P17.1 |
| STAM2 | SNRK-AS1 |
| STAM2 | RP11-476D10.1 |
| SPRED2 | LPCAT2 |
| SPRED2 | BROX |
| SPRED2 | DLGAP1-AS2 |
| SPRED2 | RP1-193H18.2 |
| PSMC3 | TMEM260 |
| PSMC3 | LPCAT2 |
| PSMC3 | NEURL1 |
| PSMC3 | MEF2A |
| PSMC3 | PRMT1 |
| PSMC3 | KLHDC3 |
| PSMC3 | DLGAP1-AS2 |
| PSMC3 | RP1-193H18.2 |
| PSMC3 | RP1-30M3.5 |
| PSMC3 | RP11-111K18.2 |
| SCARB2 | TMEM260 |
| SCARB2 | TMED8 |
| SCARB2 | LPCAT2 |
| SCARB2 | BAZ2B |
| SCARB2 | TBCC |
| SCARB2 | INTS6 |
| SCARB2 | MEF2A |
| SCARB2 | GNB4 |
| SCARB2 | SELT |
| SCARB2 | SETD7 |
| SCARB2 | BROX |
| SCARB2 | DNAJA2 |
| SCARB2 | ADRBK2 |
| SCARB2 | CLIC4 |
| SCARB2 | DLGAP1-AS2 |
| SCARB2 | RP1-193H18.2 |
| SCARB2 | RP1-30M3.5 |
| SCARB2 | RP11-111K18.2 |
| IPCEF1 | TMEM260 |
| IPCEF1 | TMED8 |
| IPCEF1 | LPCAT2 |
| IPCEF1 | BAZ2B |
| IPCEF1 | MEF2A |
| IPCEF1 | SLX4IP |
| IPCEF1 | SELT |
| IPCEF1 | BROX |
| IPCEF1 | FAM199X |
| IPCEF1 | DLGAP1-AS2 |
| IPCEF1 | RP1-193H18.2 |
| IPCEF1 | RP11-111K18.2 |
| TMEM260 | TMED8 |
| TMEM260 | LPCAT2 |
| TMEM260 | WSB1 |
| TMEM260 | SCCPDH |
| TMEM260 | BAZ2B |
| TMEM260 | GORASP2 |
| TMEM260 | EIF2AK2 |
| TMEM260 | TBCC |
| TMEM260 | GNAI3 |
| TMEM260 | MPPED2 |
| TMEM260 | NEURL1 |
| TMEM260 | INTS6 |
| TMEM260 | TFG |
| TMEM260 | MEF2A |
| TMEM260 | GOLPH3L |
| TMEM260 | CLIC3 |
| TMEM260 | MSX1 |
| TMEM260 | SCYL2 |
| TMEM260 | SLX4IP |
| TMEM260 | NBN |
| TMEM260 | PLD4 |
| TMEM260 | BIRC2 |
| TMEM260 | GNB4 |
| TMEM260 | SELT |
| TMEM260 | DCUN1D1 |
| TMEM260 | ASPH |
| TMEM260 | CEP164 |
| TMEM260 | BROX |
| TMEM260 | SUMF1 |
| TMEM260 | ARRDC4 |
| TMEM260 | DNAJA2 |
| TMEM260 | FAM199X |
| TMEM260 | ADRBK2 |
| TMEM260 | LRTOMT |
| TMEM260 | GOLPH3 |
| TMEM260 | TTC28 |
| TMEM260 | SCPEP1 |
| TMEM260 | CMTM4 |
| TMEM260 | F8 |
| TMEM260 | FLVCR2 |
| TMEM260 | MS4A4A |
| TMEM260 | ACP6 |
| TMEM260 | BRCA1 |
| TMEM260 | NPEPL1 |
| TMEM260 | PAPSS1 |
| TMEM260 | DDIT3 |
| TMEM260 | RRM2B |
| TMEM260 | PHTF1 |
| TMEM260 | CLIC4 |
| TMEM260 | SLC1A3 |
| TMEM260 | TLR10 |
| TMEM260 | DSE |
| TMEM260 | PRMT5 |
| TMEM260 | MTL5 |
| TMEM260 | VAV3 |
| TMEM260 | CREB1 |
| TMEM260 | GAPT |
| TMEM260 | ATP10D |
| TMEM260 | CC2D2B |
| TMEM260 | NKAP |
| TMEM260 | IFIT5 |
| TMEM260 | DLGAP1-AS2 |
| TMEM260 | RP1-193H18.2 |
| TMEM260 | LINC00282 |
| TMEM260 | DKFZP434L187 |
| TMEM260 | RP1-30M3.5 |
| TMEM260 | RP11-111K18.2 |
| TMEM260 | RP11-399O19.9 |
| TMEM260 | KB-431C1.4 |
| TMEM260 | RP11-1024P17.1 |
| TMEM260 | SNRK-AS1 |
| TMEM260 | RP11-476D10.1 |
| TMED8 | LPCAT2 |
| TMED8 | WSB1 |
| TMED8 | SCCPDH |
| TMED8 | BAZ2B |
| TMED8 | EIF2AK2 |
| TMED8 | TBCC |
| TMED8 | GNAI3 |
| TMED8 | NEURL1 |
| TMED8 | INTS6 |
| TMED8 | TFG |
| TMED8 | MEF2A |
| TMED8 | PRMT1 |
| TMED8 | GOLPH3L |
| TMED8 | SCYL2 |
| TMED8 | SLX4IP |
| TMED8 | NBN |
| TMED8 | BIRC2 |
| TMED8 | GNB4 |
| TMED8 | SELT |
| TMED8 | DCUN1D1 |
| TMED8 | ASPH |
| TMED8 | BROX |
| TMED8 | ARRDC4 |
| TMED8 | DNAJA2 |
| TMED8 | FAM199X |
| TMED8 | ADRBK2 |
| TMED8 | LRTOMT |
| TMED8 | GOLPH3 |
| TMED8 | SCPEP1 |
| TMED8 | CMTM4 |
| TMED8 | F8 |
| TMED8 | FLVCR2 |
| TMED8 | MS4A4A |
| TMED8 | ACP6 |
| TMED8 | BRCA1 |
| TMED8 | PAPSS1 |
| TMED8 | DDIT3 |
| TMED8 | RRM2B |
| TMED8 | PHTF1 |
| TMED8 | SLC1A3 |
| TMED8 | TLR10 |
| TMED8 | DSE |
| TMED8 | MTL5 |
| TMED8 | VAV3 |
| TMED8 | CREB1 |
| TMED8 | GAPT |
| TMED8 | ATP10D |
| TMED8 | DLGAP1-AS2 |
| TMED8 | RP1-193H18.2 |
| TMED8 | LINC00282 |
| TMED8 | RP1-30M3.5 |
| TMED8 | RP11-111K18.2 |
| TMED8 | KB-431C1.4 |
| TMED8 | SNRK-AS1 |
| LPCAT2 | WSB1 |
| LPCAT2 | SCCPDH |
| LPCAT2 | BAZ2B |
| LPCAT2 | EIF2AK2 |
| LPCAT2 | TBCC |
| LPCAT2 | GNAI3 |
| LPCAT2 | MPPED2 |
| LPCAT2 | NEURL1 |
| LPCAT2 | SFN |
| LPCAT2 | INTS6 |
| LPCAT2 | TFG |
| LPCAT2 | MEF2A |
| LPCAT2 | PRMT1 |
| LPCAT2 | GOLPH3L |
| LPCAT2 | CLIC3 |
| LPCAT2 | UBE2E3 |
| LPCAT2 | MSX1 |
| LPCAT2 | AREL1 |
| LPCAT2 | S1PR3 |
| LPCAT2 | SCYL2 |
| LPCAT2 | SLX4IP |
| LPCAT2 | RNF165 |
| LPCAT2 | NBN |
| LPCAT2 | PLD4 |
| LPCAT2 | CYBB |
| LPCAT2 | C8orf88 |
| LPCAT2 | BIRC2 |
| LPCAT2 | GNB4 |
| LPCAT2 | SELT |
| LPCAT2 | DCUN1D1 |
| LPCAT2 | ASPH |
| LPCAT2 | SETD7 |
| LPCAT2 | MMP19 |
| LPCAT2 | CEP164 |
| LPCAT2 | GPER1 |
| LPCAT2 | BROX |
| LPCAT2 | SUMF1 |
| LPCAT2 | FBN2 |
| LPCAT2 | ARRDC4 |
| LPCAT2 | DNAJA2 |
| LPCAT2 | CYFIP1 |
| LPCAT2 | TPM2 |
| LPCAT2 | BCAT1 |
| LPCAT2 | FAM199X |
| LPCAT2 | ADRBK2 |
| LPCAT2 | LRTOMT |
| LPCAT2 | GOLPH3 |
| LPCAT2 | ARF3 |
| LPCAT2 | TTC28 |
| LPCAT2 | SCPEP1 |
| LPCAT2 | CMTM4 |
| LPCAT2 | ALKBH2 |
| LPCAT2 | F8 |
| LPCAT2 | FLVCR2 |
| LPCAT2 | MS4A4A |
| LPCAT2 | ACP6 |
| LPCAT2 | BRCA1 |
| LPCAT2 | TRAPPC6A |
| LPCAT2 | NPEPL1 |
| LPCAT2 | PAPSS1 |
| LPCAT2 | DDIT3 |
| LPCAT2 | RRM2B |
| LPCAT2 | PHTF1 |
| LPCAT2 | CLIC4 |
| LPCAT2 | TLR10 |
| LPCAT2 | DSE |
| LPCAT2 | PRMT5 |
| LPCAT2 | MTL5 |
| LPCAT2 | VAV3 |
| LPCAT2 | CREB1 |
| LPCAT2 | GAPT |
| LPCAT2 | ATP10D |
| LPCAT2 | CC2D2B |
| LPCAT2 | MATK |
| LPCAT2 | MPZL2 |
| LPCAT2 | PHACTR1 |
| LPCAT2 | NKAP |
| LPCAT2 | SEMG1 |
| LPCAT2 | IFIT5 |
| LPCAT2 | DLGAP1-AS2 |
| LPCAT2 | RP1-193H18.2 |
| LPCAT2 | LINC00282 |
| LPCAT2 | DKFZP434L187 |
| LPCAT2 | RP1-30M3.5 |
| LPCAT2 | RP11-111K18.2 |
| LPCAT2 | RP11-2E11.9 |
| LPCAT2 | RP11-399O19.9 |
| LPCAT2 | KB-431C1.4 |
| LPCAT2 | RP11-1024P17.1 |
| LPCAT2 | SNRK-AS1 |
| LPCAT2 | RP11-476D10.1 |
| WSB1 | BAZ2B |
| WSB1 | INTS6 |
| WSB1 | MEF2A |
| WSB1 | SCYL2 |
| WSB1 | GNB4 |
| WSB1 | SELT |
| WSB1 | ASPH |
| WSB1 | BROX |
| WSB1 | DNAJA2 |
| WSB1 | DDIT3 |
| WSB1 | DLGAP1-AS2 |
| WSB1 | RP1-193H18.2 |
| WSB1 | RP1-30M3.5 |
| WSB1 | RP11-111K18.2 |
| WSB1 | KB-431C1.4 |
| SCCPDH | BAZ2B |
| SCCPDH | TBCC |
| SCCPDH | GNAI3 |
| SCCPDH | MEF2A |
| SCCPDH | GNB4 |
| SCCPDH | BROX |
| SCCPDH | SUMF1 |
| SCCPDH | DNAJA2 |
| SCCPDH | CMTM4 |
| SCCPDH | DLGAP1-AS2 |
| SCCPDH | RP1-193H18.2 |
| SCCPDH | LINC00282 |
| SCCPDH | RP1-30M3.5 |
| C7orf73 | DLGAP1-AS2 |
| BAZ2B | EIF2AK2 |
| BAZ2B | TBCC |
| BAZ2B | GNAI3 |
| BAZ2B | NEURL1 |
| BAZ2B | INTS6 |
| BAZ2B | TFG |
| BAZ2B | MEF2A |
| BAZ2B | PRMT1 |
| BAZ2B | GOLPH3L |
| BAZ2B | CLIC3 |
| BAZ2B | UBE2E3 |
| BAZ2B | MSX1 |
| BAZ2B | AREL1 |
| BAZ2B | S1PR3 |
| BAZ2B | SCYL2 |
| BAZ2B | SLX4IP |
| BAZ2B | NBN |
| BAZ2B | PLD4 |
| BAZ2B | CYBB |
| BAZ2B | BIRC2 |
| BAZ2B | GNB4 |
| BAZ2B | SELT |
| BAZ2B | DCUN1D1 |
| BAZ2B | ASPH |
| BAZ2B | MMP19 |
| BAZ2B | CEP164 |
| BAZ2B | GPER1 |
| BAZ2B | BROX |
| BAZ2B | SUMF1 |
| BAZ2B | FBN2 |
| BAZ2B | ARRDC4 |
| BAZ2B | DNAJA2 |
| BAZ2B | CYFIP1 |
| BAZ2B | BCAT1 |
| BAZ2B | LRTOMT |
| BAZ2B | GOLPH3 |
| BAZ2B | TTC28 |
| BAZ2B | SCPEP1 |
| BAZ2B | CMTM4 |
| BAZ2B | F8 |
| BAZ2B | FLVCR2 |
| BAZ2B | MS4A4A |
| BAZ2B | BRCA1 |
| BAZ2B | TRAPPC6A |
| BAZ2B | NPEPL1 |
| BAZ2B | PAPSS1 |
| BAZ2B | DDIT3 |
| BAZ2B | RRM2B |
| BAZ2B | PHTF1 |
| BAZ2B | CLIC4 |
| BAZ2B | DSE |
| BAZ2B | PRMT5 |
| BAZ2B | MTL5 |
| BAZ2B | VAV3 |
| BAZ2B | CREB1 |
| BAZ2B | GAPT |
| BAZ2B | ATP10D |
| BAZ2B | MPZL2 |
| BAZ2B | IFIT5 |
| BAZ2B | DLGAP1-AS2 |
| BAZ2B | RP1-193H18.2 |
| BAZ2B | LINC00282 |
| BAZ2B | RP1-30M3.5 |
| BAZ2B | RP11-111K18.2 |
| BAZ2B | RP11-2E11.9 |
| BAZ2B | RP11-399O19.9 |
| BAZ2B | KB-431C1.4 |
| BAZ2B | SNRK-AS1 |
| BAZ2B | RP11-476D10.1 |
| NDST3 | DLGAP1-AS2 |
| NDST3 | RP1-193H18.2 |
| GORASP2 | MEF2A |
| GORASP2 | SCYL2 |
| GORASP2 | DLGAP1-AS2 |
| GORASP2 | RP1-193H18.2 |
| EIF2AK2 | GNAI3 |
| EIF2AK2 | INTS6 |
| EIF2AK2 | MEF2A |
| EIF2AK2 | GNB4 |
| EIF2AK2 | SELT |
| EIF2AK2 | BROX |
| EIF2AK2 | DNAJA2 |
| EIF2AK2 | DLGAP1-AS2 |
| EIF2AK2 | RP1-193H18.2 |
| EIF2AK2 | RP1-30M3.5 |
| EIF2AK2 | KB-431C1.4 |
| TBCC | GNAI3 |
| TBCC | NEURL1 |
| TBCC | INTS6 |
| TBCC | TFG |
| TBCC | MEF2A |
| TBCC | UBE2E3 |
| TBCC | AREL1 |
| TBCC | SLX4IP |
| TBCC | NBN |
| TBCC | PLD4 |
| TBCC | OGFOD3 |
| TBCC | AK2 |
| TBCC | GNB4 |
| TBCC | SELT |
| TBCC | DCUN1D1 |
| TBCC | ASPH |
| TBCC | BROX |
| TBCC | SUMF1 |
| TBCC | ARRDC4 |
| TBCC | DNAJA2 |
| TBCC | FAM199X |
| TBCC | LRTOMT |
| TBCC | GOLPH3 |
| TBCC | TTC28 |
| TBCC | SCPEP1 |
| TBCC | CMTM4 |
| TBCC | F8 |
| TBCC | FLVCR2 |
| TBCC | ACP6 |
| TBCC | BRCA1 |
| TBCC | PAPSS1 |
| TBCC | DDIT3 |
| TBCC | RRM2B |
| TBCC | MTL5 |
| TBCC | VAV3 |
| TBCC | CREB1 |
| TBCC | GAPT |
| TBCC | ATP10D |
| TBCC | DLGAP1-AS2 |
| TBCC | RP1-193H18.2 |
| TBCC | LINC00282 |
| TBCC | DKFZP434L187 |
| TBCC | RP1-30M3.5 |
| TBCC | RP11-111K18.2 |
| TBCC | KB-431C1.4 |
| TBCC | SNRK-AS1 |
| GNAI3 | SFN |
| GNAI3 | INTS6 |
| GNAI3 | TFG |
| GNAI3 | MEF2A |
| GNAI3 | PRMT1 |
| GNAI3 | UBE2E3 |
| GNAI3 | AREL1 |
| GNAI3 | S1PR3 |
| GNAI3 | NBN |
| GNAI3 | CYBB |
| GNAI3 | AK2 |
| GNAI3 | GNB4 |
| GNAI3 | ASPH |
| GNAI3 | SETD7 |
| GNAI3 | ARL15 |
| GNAI3 | MMP19 |
| GNAI3 | PLIN2 |
| GNAI3 | BROX |
| GNAI3 | SUMF1 |
| GNAI3 | ARRDC4 |
| GNAI3 | DNAJA2 |
| GNAI3 | CYFIP1 |
| GNAI3 | TPM2 |
| GNAI3 | GOLPH3 |
| GNAI3 | ARF3 |
| GNAI3 | TTC28 |
| GNAI3 | SCPEP1 |
| GNAI3 | CMTM4 |
| GNAI3 | FLVCR2 |
| GNAI3 | TRAPPC6A |
| GNAI3 | RRM2B |
| GNAI3 | PHTF1 |
| GNAI3 | VAV3 |
| GNAI3 | GAPT |
| GNAI3 | RLTPR |
| GNAI3 | DLGAP1-AS2 |
| GNAI3 | RP1-193H18.2 |
| GNAI3 | LINC00282 |
| GNAI3 | RP1-30M3.5 |
| GNAI3 | RP11-2E11.9 |
| GNAI3 | KB-431C1.4 |
| MPPED2 | MEF2A |
| MPPED2 | SELT |
| MPPED2 | BROX |
| MPPED2 | DLGAP1-AS2 |
| MPPED2 | RP1-193H18.2 |
| NEURL1 | MEF2A |
| NEURL1 | SCYL2 |
| NEURL1 | DNAJA2 |
| NEURL1 | KLHDC3 |
| NEURL1 | DLGAP1-AS2 |
| NEURL1 | RP1-193H18.2 |
| NEURL1 | RP1-30M3.5 |
| SFN | PRMT1 |
| SFN | TPM2 |
| SFN | CHMP7 |
| SFN | DLGAP1-AS2 |
| SFN | RP1-193H18.2 |
| INTS6 | TFG |
| INTS6 | MEF2A |
| INTS6 | GOLPH3L |
| INTS6 | CLIC3 |
| INTS6 | MSX1 |
| INTS6 | SCYL2 |
| INTS6 | SLX4IP |
| INTS6 | NBN |
| INTS6 | CYBB |
| INTS6 | BIRC2 |
| INTS6 | GNB4 |
| INTS6 | SELT |
| INTS6 | DCUN1D1 |
| INTS6 | ASPH |
| INTS6 | BROX |
| INTS6 | FBN2 |
| INTS6 | ARRDC4 |
| INTS6 | DNAJA2 |
| INTS6 | FAM199X |
| INTS6 | LRTOMT |
| INTS6 | GOLPH3 |
| INTS6 | SCPEP1 |
| INTS6 | CMTM4 |
| INTS6 | F8 |
| INTS6 | BRCA1 |
| INTS6 | PAPSS1 |
| INTS6 | RRM2B |
| INTS6 | CLIC4 |
| INTS6 | DSE |
| INTS6 | CREB1 |
| INTS6 | GAPT |
| INTS6 | DLGAP1-AS2 |
| INTS6 | RP1-193H18.2 |
| INTS6 | RP1-30M3.5 |
| INTS6 | RP11-111K18.2 |
| INTS6 | KB-431C1.4 |
| INTS6 | RP11-1024P17.1 |
| TFG | MEF2A |
| TFG | CLIC3 |
| TFG | SCYL2 |
| TFG | SLX4IP |
| TFG | NBN |
| TFG | GNB4 |
| TFG | SELT |
| TFG | ASPH |
| TFG | BROX |
| TFG | DNAJA2 |
| TFG | GOLPH3 |
| TFG | CMTM4 |
| TFG | PAPSS1 |
| TFG | RRM2B |
| TFG | CREB1 |
| TFG | DLGAP1-AS2 |
| TFG | RP1-193H18.2 |
| TFG | LINC00282 |
| TFG | RP1-30M3.5 |
| TFG | RP11-111K18.2 |
| TFG | KB-431C1.4 |
| MEF2A | ASCC1 |
| MEF2A | PRMT1 |
| MEF2A | GOLPH3L |
| MEF2A | CLIC3 |
| MEF2A | UBE2E3 |
| MEF2A | MSX1 |
| MEF2A | S1PR3 |
| MEF2A | SCYL2 |
| MEF2A | SLX4IP |
| MEF2A | NBN |
| MEF2A | CYBB |
| MEF2A | BIRC2 |
| MEF2A | GNB4 |
| MEF2A | SELT |
| MEF2A | DCUN1D1 |
| MEF2A | ASPH |
| MEF2A | SETD7 |
| MEF2A | CEP164 |
| MEF2A | GPER1 |
| MEF2A | BROX |
| MEF2A | SUMF1 |
| MEF2A | ARRDC4 |
| MEF2A | DNAJA2 |
| MEF2A | CYFIP1 |
| MEF2A | FAM199X |
| MEF2A | ADRBK2 |
| MEF2A | LRTOMT |
| MEF2A | GOLPH3 |
| MEF2A | TTC28 |
| MEF2A | SCPEP1 |
| MEF2A | CMTM4 |
| MEF2A | F8 |
| MEF2A | FLVCR2 |
| MEF2A | MS4A4A |
| MEF2A | ACP6 |
| MEF2A | BRCA1 |
| MEF2A | NPEPL1 |
| MEF2A | PAPSS1 |
| MEF2A | DDIT3 |
| MEF2A | RRM2B |
| MEF2A | PHTF1 |
| MEF2A | CLIC4 |
| MEF2A | SLC1A3 |
| MEF2A | TLR10 |
| MEF2A | DSE |
| MEF2A | PRMT5 |
| MEF2A | MTL5 |
| MEF2A | VAV3 |
| MEF2A | CREB1 |
| MEF2A | GAPT |
| MEF2A | ATP10D |
| MEF2A | CC2D2B |
| MEF2A | RNASE4 |
| MEF2A | FLT3 |
| MEF2A | NKAP |
| MEF2A | IFIT5 |
| MEF2A | DLGAP1-AS2 |
| MEF2A | RP1-193H18.2 |
| MEF2A | LINC00282 |
| MEF2A | DKFZP434L187 |
| MEF2A | RP1-30M3.5 |
| MEF2A | RP11-111K18.2 |
| MEF2A | RP11-399O19.9 |
| MEF2A | KB-431C1.4 |
| MEF2A | RP11-1024P17.1 |
| MEF2A | SNRK-AS1 |
| MEF2A | RP11-476D10.1 |
| ASCC1 | DLGAP1-AS2 |
| ASCC1 | RP1-193H18.2 |
| PRMT1 | AREL1 |
| PRMT1 | NBN |
| PRMT1 | GNB4 |
| PRMT1 | CHMP7 |
| PRMT1 | TRAPPC6A |
| PRMT1 | CD27 |
| PRMT1 | DLGAP1-AS2 |
| PRMT1 | RP1-193H18.2 |
| PRMT1 | RP1-30M3.5 |
| PRMT1 | RP11-111K18.2 |
| GOLPH3L | SCYL2 |
| GOLPH3L | SLX4IP |
| GOLPH3L | BIRC2 |
| GOLPH3L | DCUN1D1 |
| GOLPH3L | BROX |
| GOLPH3L | DNAJA2 |
| GOLPH3L | DLGAP1-AS2 |
| GOLPH3L | RP1-193H18.2 |
| GOLPH3L | RP1-30M3.5 |
| CLIC3 | SCYL2 |
| CLIC3 | SLX4IP |
| CLIC3 | RNF165 |
| CLIC3 | GNB4 |
| CLIC3 | BROX |
| CLIC3 | DNAJA2 |
| CLIC3 | MLC1 |
| CLIC3 | RRM2B |
| CLIC3 | MATK |
| CLIC3 | DLGAP1-AS2 |
| CLIC3 | RP1-193H18.2 |
| CLIC3 | RP1-30M3.5 |
| CLIC3 | KB-431C1.4 |
| UBE2E3 | DNAJA2 |
| UBE2E3 | DLGAP1-AS2 |
| UBE2E3 | RP1-193H18.2 |
| UBE2E3 | RP1-30M3.5 |
| MSX1 | GNB4 |
| MSX1 | BROX |
| MSX1 | DNAJA2 |
| MSX1 | RRM2B |
| MSX1 | DLGAP1-AS2 |
| MSX1 | RP1-193H18.2 |
| MSX1 | RP1-30M3.5 |
| MSX1 | RP11-111K18.2 |
| MSX1 | KB-431C1.4 |
| AREL1 | NBN |
| AREL1 | TPM2 |
| AREL1 | ARF3 |
| AREL1 | VAV3 |
| AREL1 | DLGAP1-AS2 |
| AREL1 | RP1-193H18.2 |
| AREL1 | RP1-30M3.5 |
| S1PR3 | GNB4 |
| S1PR3 | CYFIP1 |
| S1PR3 | CMTM4 |
| S1PR3 | DLGAP1-AS2 |
| S1PR3 | RP1-193H18.2 |
| SCYL2 | SLX4IP |
| SCYL2 | BIRC2 |
| SCYL2 | GNB4 |
| SCYL2 | SELT |
| SCYL2 | DCUN1D1 |
| SCYL2 | ASPH |
| SCYL2 | BROX |
| SCYL2 | DNAJA2 |
| SCYL2 | CMTM4 |
| SCYL2 | PAPSS1 |
| SCYL2 | RRM2B |
| SCYL2 | CLIC4 |
| SCYL2 | DSE |
| SCYL2 | CREB1 |
| SCYL2 | GAPT |
| SCYL2 | MOSPD1 |
| SCYL2 | DLGAP1-AS2 |
| SCYL2 | RP1-193H18.2 |
| SCYL2 | LINC00282 |
| SCYL2 | RP1-30M3.5 |
| SCYL2 | KB-431C1.4 |
| SCYL2 | RP11-1024P17.1 |
| SIX3 | DLGAP1-AS2 |
| SLX4IP | NBN |
| SLX4IP | GNB4 |
| SLX4IP | SELT |
| SLX4IP | ASPH |
| SLX4IP | SETD7 |
| SLX4IP | BROX |
| SLX4IP | DNAJA2 |
| SLX4IP | FAM199X |
| SLX4IP | GOLPH3 |
| SLX4IP | PAPSS1 |
| SLX4IP | RRM2B |
| SLX4IP | CLIC4 |
| SLX4IP | DSE |
| SLX4IP | CREB1 |
| SLX4IP | GAPT |
| SLX4IP | DLGAP1-AS2 |
| SLX4IP | RP1-193H18.2 |
| SLX4IP | RP1-30M3.5 |
| SLX4IP | RP11-111K18.2 |
| SLX4IP | KB-431C1.4 |
| SLX4IP | RP11-1024P17.1 |
| RNF165 | MLC1 |
| RNF165 | DLGAP1-AS2 |
| RNF165 | RP1-193H18.2 |
| NBN | GNB4 |
| NBN | SELT |
| NBN | ASPH |
| NBN | BROX |
| NBN | DNAJA2 |
| NBN | GOLPH3 |
| NBN | PAPSS1 |
| NBN | RRM2B |
| NBN | CREB1 |
| NBN | DLGAP1-AS2 |
| NBN | RP1-193H18.2 |
| NBN | RP1-30M3.5 |
| NBN | RP11-111K18.2 |
| NBN | KB-431C1.4 |
| PLD4 | DLGAP1-AS2 |
| PLD4 | RP1-193H18.2 |
| PLD4 | RP1-30M3.5 |
| OGFOD3 | AK2 |
| OGFOD3 | DLGAP1-AS2 |
| OGFOD3 | RP1-193H18.2 |
| OGFOD3 | RP1-30M3.5 |
| CYBB | GNB4 |
| CYBB | FBN2 |
| CYBB | ADRBK2 |
| CYBB | EPB41L3 |
| CYBB | SGMS2 |
| CYBB | DLGAP1-AS2 |
| CYBB | RP1-193H18.2 |
| C8orf88 | DLGAP1-AS2 |
| C8orf88 | RP1-193H18.2 |
| AK2 | DLGAP1-AS2 |
| AK2 | RP1-193H18.2 |
| AK2 | RP1-30M3.5 |
| BIRC2 | GNB4 |
| BIRC2 | DCUN1D1 |
| BIRC2 | BROX |
| BIRC2 | DNAJA2 |
| BIRC2 | MOSPD1 |
| BIRC2 | DLGAP1-AS2 |
| BIRC2 | RP1-193H18.2 |
| BIRC2 | LINC00282 |
| BIRC2 | RP1-30M3.5 |
| BIRC2 | KB-431C1.4 |
| GNB4 | SELT |
| GNB4 | DCUN1D1 |
| GNB4 | ASPH |
| GNB4 | CEP164 |
| GNB4 | BROX |
| GNB4 | SUMF1 |
| GNB4 | FBN2 |
| GNB4 | ARRDC4 |
| GNB4 | DNAJA2 |
| GNB4 | CYFIP1 |
| GNB4 | ADRBK2 |
| GNB4 | LRTOMT |
| GNB4 | GOLPH3 |
| GNB4 | TTC28 |
| GNB4 | SCPEP1 |
| GNB4 | CMTM4 |
| GNB4 | F8 |
| GNB4 | MS4A4A |
| GNB4 | BRCA1 |
| GNB4 | TRAPPC6A |
| GNB4 | NPEPL1 |
| GNB4 | PAPSS1 |
| GNB4 | DDIT3 |
| GNB4 | RRM2B |
| GNB4 | PHTF1 |
| GNB4 | CLIC4 |
| GNB4 | DSE |
| GNB4 | PRMT5 |
| GNB4 | VAV3 |
| GNB4 | CREB1 |
| GNB4 | GAPT |
| GNB4 | RNASE4 |
| GNB4 | DLGAP1-AS2 |
| GNB4 | RP1-193H18.2 |
| GNB4 | LINC00282 |
| GNB4 | RP1-30M3.5 |
| GNB4 | RP11-111K18.2 |
| GNB4 | KB-431C1.4 |
| GNB4 | RP11-476D10.1 |
| SELT | DCUN1D1 |
| SELT | ASPH |
| SELT | BROX |
| SELT | ARRDC4 |
| SELT | DNAJA2 |
| SELT | FAM199X |
| SELT | LRTOMT |
| SELT | GOLPH3 |
| SELT | SCPEP1 |
| SELT | F8 |
| SELT | MS4A4A |
| SELT | BRCA1 |
| SELT | NPEPL1 |
| SELT | PAPSS1 |
| SELT | DDIT3 |
| SELT | RRM2B |
| SELT | PHTF1 |
| SELT | DSE |
| SELT | VAV3 |
| SELT | CREB1 |
| SELT | CC2D2B |
| SELT | DLGAP1-AS2 |
| SELT | RP1-193H18.2 |
| SELT | RP1-30M3.5 |
| SELT | RP11-111K18.2 |
| SELT | RP11-399O19.9 |
| SELT | KB-431C1.4 |
| DCUN1D1 | ASPH |
| DCUN1D1 | BROX |
| DCUN1D1 | DNAJA2 |
| DCUN1D1 | MOSPD1 |
| DCUN1D1 | DLGAP1-AS2 |
| DCUN1D1 | RP1-193H18.2 |
| DCUN1D1 | LINC00282 |
| DCUN1D1 | RP1-30M3.5 |
| DCUN1D1 | KB-431C1.4 |
| ASPH | BROX |
| ASPH | ARRDC4 |
| ASPH | DNAJA2 |
| ASPH | FAM199X |
| ASPH | LRTOMT |
| ASPH | GOLPH3 |
| ASPH | SCPEP1 |
| ASPH | F8 |
| ASPH | FLVCR2 |
| ASPH | PAPSS1 |
| ASPH | DDIT3 |
| ASPH | RRM2B |
| ASPH | SLC1A3 |
| ASPH | TLR10 |
| ASPH | DSE |
| ASPH | VAV3 |
| ASPH | CREB1 |
| ASPH | GAPT |
| ASPH | CC2D2B |
| ASPH | DLGAP1-AS2 |
| ASPH | RP1-193H18.2 |
| ASPH | RP1-30M3.5 |
| ASPH | RP11-111K18.2 |
| ASPH | KB-431C1.4 |
| SETD7 | CYFIP1 |
| SETD7 | DLGAP1-AS2 |
| SETD7 | RP1-193H18.2 |
| SETD7 | RP1-30M3.5 |
| ARL15 | DLGAP1-AS2 |
| ARL15 | RP1-193H18.2 |
| MMP19 | DLGAP1-AS2 |
| MMP19 | RP1-193H18.2 |
| CEP164 | BROX |
| CEP164 | DLGAP1-AS2 |
| CEP164 | RP1-193H18.2 |
| PLIN2 | DLGAP1-AS2 |
| PLIN2 | RP1-193H18.2 |
| PLIN2 | RP1-30M3.5 |
| GPER1 | DLGAP1-AS2 |
| GPER1 | RP1-193H18.2 |
| BROX | SUMF1 |
| BROX | ARRDC4 |
| BROX | DNAJA2 |
| BROX | FAM199X |
| BROX | LRTOMT |
| BROX | GOLPH3 |
| BROX | SCPEP1 |
| BROX | CMTM4 |
| BROX | F8 |
| BROX | MS4A4A |
| BROX | BRCA1 |
| BROX | NPEPL1 |
| BROX | PAPSS1 |
| BROX | DDIT3 |
| BROX | RRM2B |
| BROX | PHTF1 |
| BROX | CLIC4 |
| BROX | TLR10 |
| BROX | DSE |
| BROX | PRMT5 |
| BROX | MTL5 |
| BROX | VAV3 |
| BROX | CREB1 |
| BROX | GAPT |
| BROX | ATP10D |
| BROX | CC2D2B |
| BROX | FLT3 |
| BROX | NKAP |
| BROX | IFIT5 |
| BROX | DLGAP1-AS2 |
| BROX | RP1-193H18.2 |
| BROX | LINC00282 |
| BROX | DKFZP434L187 |
| BROX | RP1-30M3.5 |
| BROX | RP11-111K18.2 |
| BROX | RP11-399O19.9 |
| BROX | KB-431C1.4 |
| BROX | RP11-1024P17.1 |
| BROX | SNRK-AS1 |
| BROX | RP11-476D10.1 |
| SUMF1 | DLGAP1-AS2 |
| SUMF1 | RP1-193H18.2 |
| SUMF1 | RP1-30M3.5 |
| FBN2 | DLGAP1-AS2 |
| FBN2 | RP1-193H18.2 |
| ARRDC4 | DNAJA2 |
| ARRDC4 | RNASE4 |
| ARRDC4 | DLGAP1-AS2 |
| ARRDC4 | RP1-193H18.2 |
| ARRDC4 | RP1-30M3.5 |
| ARRDC4 | RP11-111K18.2 |
| ARRDC4 | KB-431C1.4 |
| DNAJA2 | FAM199X |
| DNAJA2 | LRTOMT |
| DNAJA2 | GOLPH3 |
| DNAJA2 | SCPEP1 |
| DNAJA2 | CMTM4 |
| DNAJA2 | F8 |
| DNAJA2 | NPEPL1 |
| DNAJA2 | PAPSS1 |
| DNAJA2 | DDIT3 |
| DNAJA2 | RRM2B |
| DNAJA2 | CLIC4 |
| DNAJA2 | DSE |
| DNAJA2 | CREB1 |
| DNAJA2 | GAPT |
| DNAJA2 | NKAP |
| DNAJA2 | DLGAP1-AS2 |
| DNAJA2 | RP1-193H18.2 |
| DNAJA2 | LINC00282 |
| DNAJA2 | RP1-30M3.5 |
| DNAJA2 | RP11-111K18.2 |
| DNAJA2 | KB-431C1.4 |
| DMRT2 | DLGAP1-AS2 |
| ZNF287 | DLGAP1-AS2 |
| CYFIP1 | CMTM4 |
| CYFIP1 | DLGAP1-AS2 |
| CYFIP1 | RP1-193H18.2 |
| TPM2 | DLGAP1-AS2 |
| TPM2 | RP1-193H18.2 |
| BCAT1 | DLGAP1-AS2 |
| BCAT1 | RP1-193H18.2 |
| FAM199X | LRTOMT |
| FAM199X | PAPSS1 |
| FAM199X | DSE |
| FAM199X | CREB1 |
| FAM199X | CC2D2B |
| FAM199X | DLGAP1-AS2 |
| FAM199X | RP1-193H18.2 |
| FAM199X | RP1-30M3.5 |
| FAM199X | RP11-111K18.2 |
| FAM199X | KB-431C1.4 |
| FAM199X | RP11-1024P17.1 |
| ADRBK2 | DLGAP1-AS2 |
| ADRBK2 | RP1-193H18.2 |
| ADRBK2 | RP1-30M3.5 |
| PNPLA3 | GALNT15 |
| LRTOMT | DLGAP1-AS2 |
| LRTOMT | RP1-193H18.2 |
| LRTOMT | RP1-30M3.5 |
| LRTOMT | RP11-111K18.2 |
| LRTOMT | KB-431C1.4 |
| GOLPH3 | RRM2B |
| GOLPH3 | CREB1 |
| GOLPH3 | DLGAP1-AS2 |
| GOLPH3 | RP1-193H18.2 |
| GOLPH3 | RP1-30M3.5 |
| GOLPH3 | RP11-111K18.2 |
| GOLPH3 | KB-431C1.4 |
| ARF3 | DLGAP1-AS2 |
| ARF3 | RP1-193H18.2 |
| TTC28 | TACR1 |
| TTC28 | DLGAP1-AS2 |
| TTC28 | RP1-193H18.2 |
| TTC28 | RP1-30M3.5 |
| SCPEP1 | DLGAP1-AS2 |
| SCPEP1 | RP1-193H18.2 |
| SCPEP1 | RP1-30M3.5 |
| CMTM4 | RNASE4 |
| CMTM4 | DLGAP1-AS2 |
| CMTM4 | RP1-193H18.2 |
| CMTM4 | LINC00282 |
| CMTM4 | RP1-30M3.5 |
| CMTM4 | KB-431C1.4 |
| ALKBH2 | TRAPPC6A |
| ALKBH2 | DLGAP1-AS2 |
| ALKBH2 | RP1-193H18.2 |
| F8 | DLGAP1-AS2 |
| F8 | RP1-193H18.2 |
| F8 | RP1-30M3.5 |
| F8 | RP11-111K18.2 |
| F8 | KB-431C1.4 |
| FLVCR2 | DLGAP1-AS2 |
| FLVCR2 | RP1-193H18.2 |
| FLVCR2 | RP1-30M3.5 |
| MS4A4A | DLGAP1-AS2 |
| MS4A4A | RP1-193H18.2 |
| CHMP7 | CD27 |
| CHMP7 | CCR7 |
| CHMP7 | DLGAP1-AS2 |
| ACP6 | DLGAP1-AS2 |
| ACP6 | RP1-193H18.2 |
| BRCA1 | DLGAP1-AS2 |
| BRCA1 | RP1-193H18.2 |
| BRCA1 | RP1-30M3.5 |
| BRCA1 | RP11-111K18.2 |
| BRCA1 | KB-431C1.4 |
| TRAPPC6A | ERI3 |
| TRAPPC6A | DLGAP1-AS2 |
| TRAPPC6A | RP1-193H18.2 |
| EPB41L3 | LDLRAD3 |
| EPB41L3 | RLTPR |
| NPEPL1 | DLGAP1-AS2 |
| NPEPL1 | RP1-193H18.2 |
| NPEPL1 | KB-431C1.4 |
| PAPSS1 | RRM2B |
| PAPSS1 | DSE |
| PAPSS1 | CREB1 |
| PAPSS1 | DLGAP1-AS2 |
| PAPSS1 | RP1-193H18.2 |
| PAPSS1 | RP1-30M3.5 |
| PAPSS1 | RP11-111K18.2 |
| PAPSS1 | KB-431C1.4 |
| DDIT3 | PRMT5 |
| DDIT3 | MTL5 |
| DDIT3 | DLGAP1-AS2 |
| DDIT3 | RP1-193H18.2 |
| DDIT3 | LINC00282 |
| DDIT3 | RP1-30M3.5 |
| DDIT3 | RP11-111K18.2 |
| DDIT3 | KB-431C1.4 |
| MLC1 | MATK |
| FAM110B | DLGAP1-AS2 |
| RRM2B | CREB1 |
| RRM2B | GAPT |
| RRM2B | DLGAP1-AS2 |
| RRM2B | RP1-193H18.2 |
| RRM2B | RP1-30M3.5 |
| RRM2B | RP11-111K18.2 |
| RRM2B | KB-431C1.4 |
| PHTF1 | DLGAP1-AS2 |
| PHTF1 | RP1-193H18.2 |
| CLIC4 | DLGAP1-AS2 |
| CLIC4 | RP1-193H18.2 |
| CLIC4 | RP1-30M3.5 |
| CLIC4 | KB-431C1.4 |
| SLC1A3 | DLGAP1-AS2 |
| SLC1A3 | RP1-193H18.2 |
| TLR10 | DLGAP1-AS2 |
| TLR10 | RP1-193H18.2 |
| TLR10 | KB-431C1.4 |
| DSE | CREB1 |
| DSE | CC2D2B |
| DSE | PAPSS2 |
| DSE | DLGAP1-AS2 |
| DSE | RP1-193H18.2 |
| DSE | RP1-30M3.5 |
| DSE | RP11-111K18.2 |
| DSE | KB-431C1.4 |
| PRMT5 | DLGAP1-AS2 |
| PRMT5 | RP1-193H18.2 |
| PRMT5 | KB-431C1.4 |
| MTL5 | DLGAP1-AS2 |
| MTL5 | RP1-193H18.2 |
| MTL5 | RP1-30M3.5 |
| MTL5 | RP11-111K18.2 |
| KNOP1 | ERI3 |
| KNOP1 | DLGAP1-AS2 |
| VAV3 | DLGAP1-AS2 |
| VAV3 | RP1-193H18.2 |
| VAV3 | RP1-30M3.5 |
| VAV3 | KB-431C1.4 |
| ERI3 | RLTPR |
| CREB1 | DLGAP1-AS2 |
| CREB1 | RP1-193H18.2 |
| CREB1 | RP1-30M3.5 |
| CREB1 | RP11-111K18.2 |
| CREB1 | KB-431C1.4 |
| GAPT | DLGAP1-AS2 |
| GAPT | RP1-193H18.2 |
| GAPT | RP1-30M3.5 |
| GAPT | RP11-111K18.2 |
| GAPT | KB-431C1.4 |
| ATP10D | DLGAP1-AS2 |
| ATP10D | RP1-193H18.2 |
| ATP10D | RP1-30M3.5 |
| ATP10D | RP11-111K18.2 |
| ATP10D | KB-431C1.4 |
| CC2D2B | DLGAP1-AS2 |
| CC2D2B | RP1-193H18.2 |
| ENOX1 | DLGAP1-AS2 |
| ENOX1 | RP1-193H18.2 |
| MPZL2 | DLGAP1-AS2 |
| MPZL2 | RP1-193H18.2 |
| MPZL2 | KB-431C1.4 |
| RNASE4 | DLGAP1-AS2 |
| RNASE4 | RP1-193H18.2 |
| PHACTR1 | DLGAP1-AS2 |
| PHACTR1 | RP1-193H18.2 |
| FLT3 | DLGAP1-AS2 |
| FLT3 | RP1-193H18.2 |
| NKAP | DLGAP1-AS2 |
| NKAP | RP1-193H18.2 |
| CD27 | CCR7 |
| IFIT5 | DLGAP1-AS2 |
| IFIT5 | RP1-193H18.2 |
| NOG | CCR7 |
| DLGAP1-AS2 | RP1-193H18.2 |
| DLGAP1-AS2 | LINC00282 |
| DLGAP1-AS2 | DKFZP434L187 |
| DLGAP1-AS2 | RP1-30M3.5 |
| DLGAP1-AS2 | RP11-111K18.2 |
| DLGAP1-AS2 | RP11-2E11.9 |
| DLGAP1-AS2 | RP11-399O19.9 |
| DLGAP1-AS2 | KB-431C1.4 |
| DLGAP1-AS2 | RP11-1024P17.1 |
| DLGAP1-AS2 | SNRK-AS1 |
| DLGAP1-AS2 | AC005785.2 |
| DLGAP1-AS2 | RP11-476D10.1 |
| RP1-193H18.2 | LINC00282 |
| RP1-193H18.2 | DKFZP434L187 |
| RP1-193H18.2 | RP1-30M3.5 |
| RP1-193H18.2 | RP11-111K18.2 |
| RP1-193H18.2 | RP11-2E11.9 |
| RP1-193H18.2 | RP11-399O19.9 |
| RP1-193H18.2 | KB-431C1.4 |
| RP1-193H18.2 | RP11-1024P17.1 |
| RP1-193H18.2 | SNRK-AS1 |
| RP1-193H18.2 | RP11-476D10.1 |
| LINC00282 | RP1-30M3.5 |
| LINC00282 | KB-431C1.4 |
| RP1-30M3.5 | RP11-111K18.2 |
| RP1-30M3.5 | KB-431C1.4 |
| RP1-30M3.5 | SNRK-AS1 |
| RP11-111K18.2 | KB-431C1.4 |
| RP11-111K18.2 | RP11-1024P17.1 |
| RP11-111K18.2 | SNRK-AS1 |
| KB-431C1.4 | RP11-1024P17.1 |
| KB-431C1.4 | SNRK-AS1 |
