# Supplementary material for: Comprehensive Analysis of Hub Genes Associated With Competing Endogenous RNA Networks in Stroke Using Bioinformatics Analysis
Source: Front Genet. 2022 Jan 12;12:779923. doi: 10.3389/fgene.2021.779923 (PMC8790239; doi:10.3389/fgene.2021.779923)
Supplement: Supplementary file 6 [file DataSheet1.ZIP › Supplementary Material Presentation-figures/Figure 5.pdf]

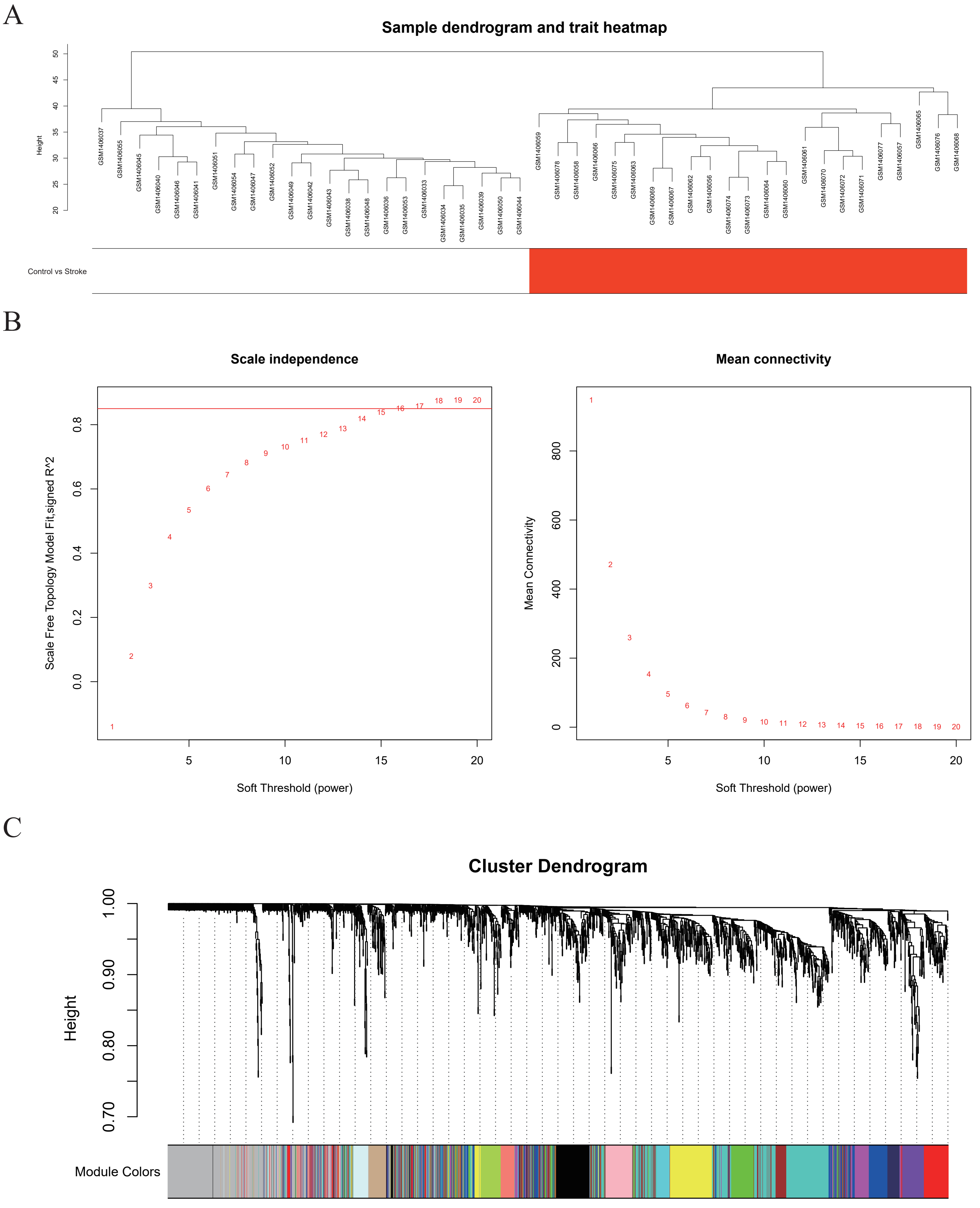
A
Sample dendrogram and trait heatmap
Height
GSM1406037
GSM1406055
GSM1406045
GSM1406040
GSM1406046
GSM1406041
GSM1406051
GSM1406054
GSM1406047
GSM1406052
GSM1406049
GSM1406042
GSM1406043
GSM1406038
GSM1406048
GSM1406036
GSM1406053
GSM1406033
GSM1406034
GSM1406035
GSM1406039
GSM1406050
GSM1406044
GSM1406059
GSM1406078
GSM1406058
GSM1406066
GSM1406075
GSM1406063
GSM1406069
GSM1406067
GSM1406062
GSM1406056
GSM1406074
GSM1406073
GSM1406064
GSM1406060
GSM1406061
GSM1406070
GSM1406072
GSM1406071
GSM1406077
GSM1406057
GSM1406065
GSM1406076
GSM1406068
Control vs Stroke
B
Scale independence
Scale Free Topology Model Fit,signed R^2
Soft Threshold (power)
Mean connectivity
Mean Connectivity
Soft Threshold (power)
C
Cluster Dendrogram
Height
Module Colors
